# Abstracts from the 2nd International Symposium on Phytochemicals in Medicine and Food (2-ISPMF)

**DOI:** 10.1186/s13020-018-0172-2

**Published:** 2018-05-14

## 1 The use of Chinese medicine in the management of psychiatric disorders: from clinical trials to psychopharmacological approaches

### Zhang-Jin Zhang

#### School of Chinese Medicine, The University of Hong Kong, Hong Kong, China

##### **Correspondence:** Zhang-Jin Zhang - zhangzj@hku.hk

*Journal of Chinese Medicine* 2018, **13(Suppl 1):**1

**Background:** There have been numerous psychological and psychiatric terms recorded in traditional Chinese medical bibliographies, developing a TCM specialty called mental-emotional diseases (神志病), in which symptomatology, etiology, psychopathology, and various therapeutic approaches have been well established.

**Results:** Chinese medicine was the mainstay in the management of mental disorders and wellbeing in ancient days. It also has been increasingly used in today’s clinical practice aimed to enhance the clinical efficacy, reduced adverse effects caused by conventional treatment and comorbid symptoms. This fact is reflected in an increasing number of clinical studies, demonstrating the potential benefits of Chinese medicine in the treatment of anxiety, mood disorders, insomnia, schizophrenia, tic disorders, and antipsychotic-induced side effects. There are also numerous studies in identifying psychopharmacological and psychotherapeutic effects of herbal medicine in in vitro and in vivo models.

**Conclusions:** This talk will provide an overview of the empirical use of Chinese medicine in the treatment of mental disorders and related research findings obtained from clinical trials and laboratory-based experiments.

## 2 An innovative platform for Traditional Medicine R&D at the University of Macau: Enhancing international collaboration in the New Era

### Chunming Wang, Yitao Wang

#### State Key Laboratory of Quality Control in Chinese Medicine, Institute of Chinese Medical Sciences, University of Macau, Macau SAR, China

##### **Correspondence:** Yitao Wang - ytwang@umac.mo

*Journal of Chinese Medicine* 2018, **13(Suppl 1):**2

Since its establishment as the first research-oriented institute in Macau in 2002, the Institute of Chinese Medical Sciences (ICMS) at the University of Macau (UM) has devoted itself to promoting the study of Chinese medical sciences with a global vision and an interdisciplinary approach. It has gained international reputation as an academic hub for research of both traditional medicine and biopharmaceutical sciences. Since December 2010, ICMS has prided itself in hosting the State Key Laboratory of Quality Research in Chinese Medicine—as China’s first ever State Key Laboratory in the field. The Institute, home to 34 principal investigators and over 200 research students, has achieved remarkable progress in terms of funding supports, research publications as well as talent nurturing. Only in the past 6 years, the team has published over 1000 papers in SCI-indexed journals (including the likes of *Nature, Medicine* and *Lancet*) as well as secured grants worth approximately USD 30 million. Nowadays, with its state-of-the-art facilities, internationally-trained researchers and solid research outputs, ICMS/SKL promises to further build up a platform for global collaboration on translational research and product development, and ultimately to position Macau as a dynamic R&D centre of traditional medicine in the region and beyond.

## 3 Alkaloids: Back to basics for drug development

### R. Verpoorte

#### Natural Products Laboratory, Institute of Biology Leiden, Leiden University, PO Box 9505, 2300RA Leiden, The Netherlands

##### **Correspondence:** R. Verpoorte - VERPOORT@chem.LeidenUniv.NL

*Journal of Chinese Medicine* 2018, **13(Suppl 1):**3

Since molecular biology started its advance some 30 years ago, it had a major landmark in obtaining the full sequence of the human genome, followed by that of various other organisms. We are now reaching the phase that the 1000 $ full sequencing of an organism becomes reality. It is almost cheaper to sequence again than to save the full sequence of an organism. At the same time it becomes clear that having a sequence does not help much to really understand a living organism. The high expectations for drug development, for example, have shown to be over optimistic, as so far no novel drugs have resulted from this knowledge.

For natural products based drug development the trend was at-random screening of crude plant extracts. More recently a metabolomics approach came into the picture as it allows fast dereplication and also allows to find possible synergy and pro-drugs, which particularly is of interest in studying medicinal plants. Such a systemic approach means a clear change from the “single compound—single target” paradigm of drug development of the past 40 years. Moreover, plants are no considered to be super organisms in the sense that they are dependent on the collaboration of the plant with all kind of microorganisms, e.g. in the rhizosphere, but also endophytes in the plant itself. That means new organisms as potential sources for drug development.

In the classical way plants were studied for single active compounds, often in a targeted approach. Particularly alkaloids are a valuable resource for drug development, about 80% of all known drugs contain an amine function.

In the past researchers specialized on a specific class of natural products. Nowadays generalists should be able to isolate and structure elucidate any compound from any source. As a result basic knowledge about the different classes is disappearing. Particularly with the open-access publication hype, journals are interested in publishing many papers and not in high quality as authors pay and not the readers, resulting in publication with surprising results.

The rapid erosion of basic knowledge makes it worth to go against this trend, back to a combination of basic knowledge on alkaloids and learning either from nature, i.e. plant environment interactions; or from our ancestors, i.e. traditional medicines. Combining alkaloid phytochemistry, pharmacology, biology and biotechnology will be the key to a better understanding of nature and applying this knowledge for applications like crop protection and novel drugs.

## 4 Protective influence of healthful nutrition on mechanisms of environmental pollutant toxicity and disease risks

### Bernhard Hennig

#### University of Kentucky Superfund Research Center, Lexington, KY 40536, USA

##### **Correspondence:** Bernhard Hennig

*Journal of Chinese Medicine* 2018, **13(Suppl 1):**4

**Background:** Human exposures to environmental contaminants in China and around the world, such as persistent chlorinated organics, heavy metals, pesticides, phthalates, flame retardants, electronic waste, and especially airborne pollutants, are significant and require urgent attention. Given this widespread contamination and abundance of such environmental pollutants in the ecosystem, it is unlikely that remediation alone will be sufficient to address the health impacts associated with these exposures. Furthermore, we must assume that the body burdens of some of these contaminants result in populations with extraordinary vulnerabilities to disease risks. Thus, exploring preventive measures of environmental exposure and disease risk through new paradigms of environmental toxicology, optimal and/or healthful nutrition, and health is essential.

**Results and discussion:** There is a significant need to further understand the relationship between lifestyle modifications and toxicant-induced diseases. Factors which can trigger the pathologies of non-communicable or chronic diseases, including atherosclerosis, diabetes, and obesity, are complex. Complex diseases often do not have a single cause, and it is the interaction between our genes, the environment (e.g., chemical or non-chemical stressors and/or buffers) we are exposed to and our lifestyles which ultimately cause or prevent complex diseases. Relevant environmental and lifestyle factors include the timing, from early development through adulthood, and duration of exposure to environmental toxicants, as well as potential nutritional interventions and the etiology of non-communicable diseases. While a sedentary lifestyle and/or poor dietary habits can exacerbate the deleterious effects resulting from exposure to toxic chemicals, much emerging evidence suggests that positive life-style changes (e.g., healthful nutrition, exercise or increased physical activity) can modulate and/or reduce the toxicity of environmental pollutants. Diet or the types of foods we eat may serve as either an agonist or an antagonist of the health impacts associated with exposure to environmental pollutants. Our work has shown that diets high in anti-inflammatory bioactive nutrients (e.g., phytochemicals or polyphenols) and increasing physical activity are two possible strategies of modulating and reducing the disease risks associated with exposure to toxic pollutants in the environment. For example, we found that animals which consumed a diet enriched with bioactive compounds common in green tea were better prepared to counteract a subsequent exposure to dioxin-like pollutants as evidenced by decreased oxidative stress and increased antioxidant defense proteins. Emerging data now implicate the importance of epigenetic control mechanisms in persistent organic pollutant-induced inflammation and prevention of toxicity by phytochemicals or bioactive nutrients. Understanding the epigenetic control mechanisms of pollutant-induced diseases will allow for more focused therapeutic and preventative measures, which can be implemented during developmental or early phases in life and thus may be highly applicable to the fields of public health and risk assessment.

**Conclusion:** Consuming healthy diets rich in plant-derived bioactive nutrients may reduce the vulnerability to diseases linked to environmental toxic insults. This nutritional paradigm in environmental toxicology requires further study in order to improve our understanding of the relationship between nutrition or other lifestyle modifications and toxicant-induced diseases.

**Acknowledgements:** Supported in part by the NIEHS/NIH grant P42ES007380.

## 5 The healthy effect of strawberry bioactive compounds: is there a possible clue on the molecular mechanisms involved in different chronic disease?

### Francesca Giampieri, Tamara Yuliett Forbes-Hernandez, Sadia Afrin, Danila Cianciosi, Massimiliano Gasparrini, Maurizio Battino

#### Centre for Nutrition & Health, Universidad Europea del Atlantico (UEA), Santander, Spain and Department of Clinical Sciences, Faculty of Medicine, Polytechnic University of Marche, Ancona, Italy

##### **Correspondence:** Maurizio Battino

*Journal of Chinese Medicine* 2018, **13(Suppl 1):**5

**Background:** Nowadays, it is generally accepted that a diet rich in fruit and vegetables is beneficial for human health. The consumption of strawberry has been associated with the maintenance of well-being and the prevention of the most common chronic diseases, thanks to the high contents of antioxidants and phytochemicals present in the fruit [1–3]. We focused especially on human dermal fibroblasts (HDF), human liver hepatocellular cells (HepG2), RAW macrophages, 3T3-L1 adipocytes and breast cancer cell line A17 for the in vitro models and on young and old rats and healthy young humans for the in vivo studies.

**Results:** Strawberry bioactive compounds were able to protect HDF and RAW stressed with different agents, reducing the intracellular ROS and NO production, lipid, protein and DNA damage, pro-inflammatory cytokines level and restoring the antioxidant enzymes reserve and mitochondrial functionality [4, 5]. At the same time, strawberry treatment was also effective in ameliorating the lipid profile by reducing lipid accumulation, low-density lipoprotein cholesterol, triglycerides levels and lipid peroxidation, in HepG2 and 3T3-L1 cells [6]. On A17 cells strawberry treatment was able to decrease the cellular viability in a time- and dose-dependent manner, reduce the number of cells in S phase and inhibit cellular mobility [7]. Strawberry consumption exerted favourable effects on FVB syngeneic mice, constraining the cellular growth of A17 cells orthotopically transplanted [7]. Moreover, in young and old rats stressed with ethanol/Doxorubicin administration strawberry consumption improved antioxidant defences, decreased biomarkers of oxidative damage and inflammation and enhanced mitochondrial functionality [8]. Finally, in human healthy volunteers, acute and medium-term strawberry intake led to significant increase in plasma total antioxidant capacity and in folate and vitamin C serum concentrations, as well as to noteworthy improvements of plasma lipid profile and erythrocyte and lymphocyte resistance to ex vivo induced oxidative damage [9].

All these biological effects have been explained mainly through the total antioxidant capacity exerted by strawberry bioactive compounds, but recently more complex mechanisms have begun to be investigated [2, 3]. We showed that strawberry polyphenols can modulate many genes involved in cellular antioxidant defences, inflammation, metabolism, survival and proliferation, such as AMPK, Nrf2, NF-kB, Mcam, among others [4, 6, 7].

**Conclusions:** Strawberry may represent a promising powerful disease-fighting food, for the prevention of chronic degenerative pathologies or in support to traditional therapies for the best achievement of therapeutic goals. However, further in vivo studies are needed to translate the in vitro evidence into in vivo outcomes and to understand the mechanisms governing the strawberry phytochemicals bioefficacy.

**References**

1. Kresty LA, Mallery SR, Stoner GD. J Berry Res. 2016; 6:251–61.
2. Bach-Faig A, Berry EM, Lairon D, et al. Public Health Nutr. 2011; 14:2274–84.
3. Edirisinghe I, Burton-Freeman B. J Berry Res. 2016; 6:237–50.
4. Afrin S, Giampieri F, Gasparrini M, et al. Molecules. 2016; 21:30.
5. Forbes-Hernandez TY, Gasparrini M, Afrin S, et al. Crit Rev Food Sci Nutr. 2016; 56:S46–59.
6. Afrin S, Gasparrini M, Forbes-Hernandez TY, et al. J Agric Food Chem. 2016; 64:4435–49.
7. Mazzoni L, Perez-Lopez P, Giampieri F, et al. J Sci Food Agric. 2016; 96:365–71.
8. Giampieri F, Forbes-Hernandez TY, Gasparrini M, et al. Food Funct. 2015; 6:1386–98.
9. Giampieri F, Alvarez-Suarez JM, Battino M. J Agric Food Chem. 2014; 62:3867–76.
10. Giampieri F, Tulipani S, Alvarez-Suarez JM et al. Nutrition. 2012; 28:9–19.
11. Giampieri F, Alvarez-Suarez JM, Mazzoni L, et al. Nat Prod Res. 2012; 27:448–55.
12. Gasparrini M, Forbes-Hernandez TY, Afrin S, et al. Int J Mol Sci. 2015; 16:17870–84.
13. Giampieri F, Alvarez-Suarez JM, Tulipani S, et al. J Agric Food Chem. 2012; 60:2322–7.
14. Giampieri F, Alvarez-Suarez JM, Mazzoni L, et al. Food Funct. 2014; 5:1939–48.
15. Giampieri F, Alvarez-Suarez JM, Mazzoni L et al. Molecules. 2014; 19:7798–816.
16. Amatori S, Mazzoni L, Alvarez-Suarez JM et al. Sci Rep. 2016; 6:30917.
17. Giampieri F, Alvarez-Suarez JM, Gasparrini M, et al. Food Chem Toxicol. 2016; 94:128–37.
18. Diamanti J, Mezzetti B, Giampieri F, et al. J Agric. Food Chem. 2014; 62:3935–43.
19. Alvarez-Suarez JM, Dekanski D et al. PLoS ONE. 2011; 6:e25878.
20. Tulipani S, Armeni T, Giampieri F, et al. Food Chem. 2014; 156:87–93.
21. Alvarez-Suarez JM, Giampieri F, Tulipani S, et al. J Nutr Biochem. 2014; 25:289–94.
22. Tulipani S, Alvarez-Suarez JM, Busco F et al. Food Chem. 2011; 128:180–6.
23. Gasparrini M, Giampieri F, et al. Curr Drug Targets. 2016; 17:865–89.
24. Forbes-Hernández TY, Giampieri F, Gasparrini M, et al. Food Chem Toxicol. 2014; 68:154–82.
25. Pistollato F, Giampieri F, Battino M. Food Chem Toxicol. 2015; 75:58–70.

## 6 Anti-inflammatory and proresolving natural products and their therapeutic potential

### Young-Joon Surh

#### Tumor Microenvironment Global Core Research Center, College of Pharmacy, Seoul National University, Seoul 08826, South Korea

##### **Correspondence:** Young-Joon Surh - surh@snu.ac.kr

*Journal of Chinese Medicine* 2018, **13(Suppl 1):**6

**Background:** The implication of inflammatory tissue damage in pathophysiology of human cancer and some metabolic disorders is under intense investigation both at the research level and in clinical practice. Numerous studies have identified a series of critical molecules/changes in the inflammatory signaling. Some of the key molecules involved in mediating proinflammatory signaling include NF-κB, STAT3, AP-1, HIF-1α, and p53 [1]. Nuclear factor E2-related factor-2 (Nrf2) plays a crucial role in regulating stress-responsive gene induction. This transcription factor is sequestered in the cytoplasm as an inactive complex with the inhibitory protein Keap1. Upon activation, Nrf2 binds to antioxidant responsive element (ARE) sites, leading to the coordinated up-regulation of target genes that encode many anti-inflammatory as well as antioxidant and other cytoprotective proteins [2].

**Results:** The proper regulation of aforementioned transcription factors mediating pro- or anti-inflammatory signaling by pharmaceutical or nutritional manipulation hence provides an important strategy for the treatment or prevention of inflammation-associated diseases. While chronic inflammation is detrimental, acute inflammation is a physiologic response to protect cells from microbial infection and other noxious stimuli. However, if timely resolution of inflammation is failed, inflammation persists and can progress to a chronic state which has long been thought as a predisposing factor to many human disorders including cancer [3]. Resolution of inflammation is an active process regulated coordinatedly by distinct anti-inflammatory and pro-resolving lipid mediators some of which are derived from n − 3 polyunsaturated fatty acids.

**Conclusion:** For efficacious prevention of inflammation-associated disorders, identification of natural products with capability to stimulate/potentiate pro-resolving/anti-inflammatory processes as well as to suppress aberrant overactivation of proinflammatory ones merit further investigation.

**References**

1. Surh YJ, Kundu JK, Na HK, Lee JS. Redox-sensitive transcription factors as prime targets for chemoprevention with anti-inflammatory and antioxidative phytochemicals. J Nutr. 2005; 135(12 Suppl):2993S–3001S.
2. Kim J, Cha YN, Surh YJ. A protective role of nuclear factor-erythroid 2-related factor-2 (Nrf2) in inflammatory disorders. Mutat Res. 2010; 690(1–2):12–23.
3. Lee HN, Na HK, Surh YJ. Resolution of inflammation as a novel chemopreventive strategy. Semin Immunopathol. 2013; 35:151–161.

## 7 Discovery of new natural enzyme inhibitors—Combination of ethnomedical data and in vitro, in vivo and in silico approach

### Ilkay Erdogan Orhan

#### Department of Pharmacognosy, Faculty of Pharmacy, Gazi University, 06330 Ankara, Turkey

##### **Correspondence:** Ilkay Erdogan Orhan

*Journal of Chinese Medicine* 2018, **13(Suppl 1):**7

**Background:** Many enzyme inhibitors from plants are currently used in clinic for treatment of various diseases. For instance; galanthamine is an alkaloid-derivative drug with effective inhibition against cholinesterases used for the treatment of Alzheimer’s disease, while the first examples of statins used against hypercholesterolemia were also of natural origin. Taking this information into account, an extensive research is being conducted on screening natural products for their enzyme inhibitory potentials.

**Materials and methods:** Consistently, we have focused on screening natural compounds for their inhibitory capacities against a number of enzymes since long time, e.g. cholinesterases, tyrosinase, lipoxygenase, elastase, collagenase, xanthine oxidase, phosphodiesterase, etc. Starting from the medicinal plants with folkloric use, we have so far obtained some interesting results through enzyme inhibition assays supported by in vivo (in some cases) and in silico experiments.

**Results:** Our new results obtained from in vitro, in vivo, and in silico experiments with natural compounds from different chemical classes such as pteryxin, resveratrol derivatives, phenolic acids, hyperforin, hyuganin C, etc. seems to be promising cholinesterase inhibitors (Fig. 1).

**Fig. 1:**
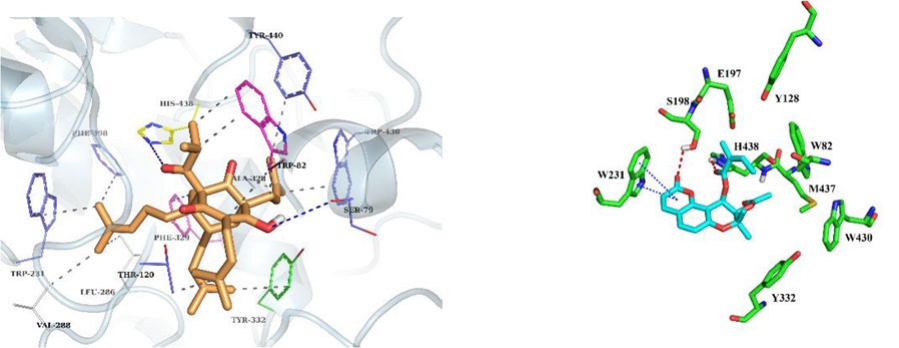
Interactions of hyperforin (orange skeleton, representation in sticks) with residues within binding site of BChE (on the left) and docking solution of natural product pteryxin (in blue) inside the active site of BChE (on the right)

**Conclusion:** Natural products have been proven to be the imperative models for new drug candidates and drug molecules.

## 8 Polyvalent naphthoquinones, a valuable family of natural compounds

### Nuria Chinchilla^1^, Guillermo A. Guerrero-Vásquez^1^, Alexandra G. Durán^1^, Mariola Macías^2^, Juan M. Sánchez-Calvo^3^, Rosa M. Varela^1^, José M. G. Molinillo^1^, Francisco A. Macías^1^

#### ^1^Allelopathy Group, Department of Organic Chemistry, Institute of Biomolecules (INBIO), Campus de Excelencia Internacional Agroalimentario (ceiA3), University of Cádiz, C/Avda. República Saharaui, 7. 11510-Puerto Real, (Cádiz), Spain; ^2^Departamento de Anatomía Patológica, Biología celular, Histología, Historia de la Ciencia, Medicina Legal y Forense y Toxicología, Facultad de Medicina, Universidad de Cádiz, Plaza Fragela, 9. 11003-Cádiz, Spain; ^3^Area de Gestión Sanitaria Norte de Cadiz, Hospital de Jerez, Unidad de Gestión Clínica de Enfermedades Infecciosas y Microbiología, Ronda de circunvalación s/n. 11407-Jerez, (Cadiz), Spain

##### **Correspondence:** Francisco A. Macías - famacias@uca.es

*Journal of Chinese Medicine* 2018, **13(Suppl 1):**8

**Background:** Quinones are organic compounds whose chemical properties allow them to interact with biological targets by forming covalent bonds and by acting as electron transfer agents in oxidation–reduction reactions. Within this class of compound, the naphthoquinones are of particular interest because of their occurrence as natural products [1–3] and as environmental chemicals [4]. The central pharmacophore 1,4-naphthoquinone is responsible for the anticancer activity shown by this type of compound [5,6]. In addition, this class of compound can also have antibacterial properties and they are therefore used as fungicides. Tropical plants such as teak (*Tectona grandis*), which is a natural source of this type of metabolite, have a high resistance to fungi and insects, including xylophagous insects. These kinds of compounds have also shown phytotoxic activity. One of the first examples of an allelochemical to be described was the naphthoquinone juglone (5-hydroxy-1,4-naphthoquinone), that show inhibition of lettuce seed germination.[7] This makes to these compounds an important future targets in the development of drugs and ecoherbicides. This gives sufficient reasons to develop efficient synthetic routes to this kind of compounds (Fig. 1).

**Fig. 1:**
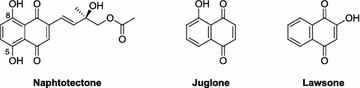
Natural products with naphtoquinones skeleton

**Results:** Naphtotectone is the most abundant and bioactive compound of teak leaves [8]. This compound provokes chromatin condensation and lost of morphology of the cellular membrane and nuclear envelope which drive to necrosis or apoptosis. The enantioselective synthesis of natural product was achieved in 7 steps and 38% overall yield. [9, 10] In addition, the phytotoxicity of this compound and its synthetic derivatives has been evaluated, with the halogen compounds being the most active [11]. The citotoxicity study of naphthoquinone analogues showed that the hydroxyl groups at positions 5 and 8 play a critical role on the activity observed.

The phytotoxicity of the natural products lawsone and juglone has also been evaluated [12]. The most active compounds were acyl derivatives introduced at position 5 and ether derivatives at position 2 into the naphthoquinone backbone (Fig. 2). The effects produced were mostly stunting and necrosis caused by growth inhibition. The parameter most affected was the root length and *Echinochloa cruss*-*galli* L. was the most sensitive weed.

**Fig. 2:**
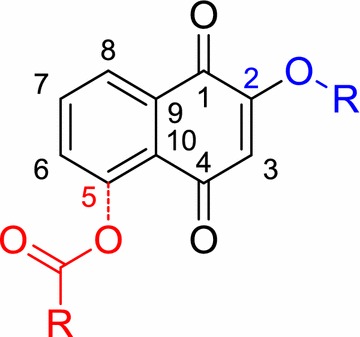
Structural requirements for enhance the bioactivity

**Conclusions:** These kinds of compounds are a clear example of how a polyvalent natural product can play a key role in nature with valuables applications in human health.

**Acknowledgments:** The authors gratefully acknowledge the Spanish Ministry of Economy and Competitivity (MINECO) (project number AGL2013-42238-R).

**References**

1. Pinto AV, de Castro SL. Molecules. 2009; 14:11.
2. Abdelmohsen K, Patak P, Von Montfort C, et al. Methods Enzymol. 2004:378.
3. Shearer MJ, Newman P. Thromb Haemost. 2008; 100:4.
4. Eiguren-Fernandez A, Miguel AH, Di Stefano E, et al. Aerosol Sci Technol. 2008; 42:10.
5. Tandon VK, Chhor RB, Singh RV, et al. Bioorg Med Chem Lett. 2004; 14:5.
6. Klotz LO, Hou X, Jacob C. Molecules. 2014; 19:9.
7. Spencer GF, Tjarks LW, England RE, et al. J Nat Prod; 1986; 49:3.
8. Macias FA, Lacret R., Varela RM, Nogueiras C. Molinillo JMG. J Chem Ecol. 2010; 36(4):396.
9. Guerrero-Vasquez GA, Galarza FAD, Molinillo JMG, et al. Eu J Org Chem. 2016; 1599–605.
10. Guerrero-Vasquez GA, Andrade CKZ, Molinillo JMG, et al. Eu J Org Chem. 2013; 6175–80.
11. Chinchilla N, Guerrero-Vásquez GA, Varela RM, Molinillo JMG, Macías FA. Res Chem Intermed. 2017.
12. Duran AG, Chinchilla N, Molinillo JMG, Macias FA. Pest Manag. Sci. 2017.

## 9 Understanding adaptogens and the specificity of the pharmacological action of phytochemicals: myth or reality?

### Alexander Panossian

#### EuroPharma USA Inc., Green Bay, WI, USA

##### **Correspondence:** Alexander Panossian - apanossian@europharmausa.com

*Journal of Chinese Medicine* 2018, **13(Suppl 1):**9

**Background:** Adaptogens are natural compounds or plant extracts that increase an organism’s non-specific resistance to stress by increasing its adaptability and survival [1]. The term adaptogens is used in alternative and complimentary medicine, pharmacognosy, phytomedicine, phytopharmacology, and phytotherapy research. Initially, the term adaptogen was coined to describe substances that can increase the “state of non-specific resistance to stress”. Originally, the adaptogenic concept was based on Hans Selye’s theory of adaptive stress response of neuroendocrine immune complex and long-term traditional use of some medicinal plants that are believed to promote physical and mental health, improve defense mechanisms of the body, and enhance longevity. It was suggested that certain compounds and herbal extracts, termed adaptogens, could diminish the magnitude of the alarm phase of adaptive stress response and prolong the duration the phase of non-specific resistance to stress. Based on evidences mainly from animal studies, adaptogens were defined as nontoxic “metabolic regulators, which increase the ability of an organism to adapt to environmental factors and to avoid damage from such factors”. It should be emphasized that the term “adaptogen” is associated with a physiological process—adaptation to environmental challenges, which is a multistep process that involves diverse mechanisms of intra- and extracellular interactions. The updated definition of adaptogens is supported by results of recent studies of molecular mechanisms of action of adaptogens on a variety of regulatory systems from the cellular level to whole organism [1].

**Materials and methods:** For assessment of the pharmacological efficacy of adaptogens, their stress-protective (e.g. survival, cell’s degeneration or apoptosis) or stimulating and tonic effects on cognitive functions, mental performance and physical endurance in stressful conditions are normally investigated. Various methods of molecular biology were used in studies of the moleculat mechanisms of action of adaptogens.

The main difference between adaptogens and conventional stimulants, such as caffeine, amphetamine, etc., is that after prolonged use, the later can cause the user to develop both tolerance and addiction. Adaptogens exhibit polyvalent beneficial effects against chronic inflammation, atherosclerosis, neurodegenerative cognitive impairment, metabolic disorders, cancer, and other aging-related diseases. All of them are associated with the metabolic regulation of homeostasis and threatened adaptability of stress system. The metabolic regulation of homeostasis by adaptogens at the cellular and systemic level is associated with multiple targets [1]. Consequently, the pharmacology of adaptogens is a typical example of network pharmacology that can be approached using the systems biology concept. The classic reductionist model that presumes a specific receptor/drug interaction is unsuitable for this scenario and insufficient when attempting to understand the mechanism of action of adaptogens. Molecular targets, signaling pathways, and networks common to adaptogens have been identified [1]. They are associated with stress hormones and key mediators of the regulation of homeostasis (molecular chaperons Hsp70, neuropeptide Y, G protein-coupled receptors, dopamine-cAMP-PKA-CERT, IP3, PLC, DAG, PI3K, NFkB, mediated signaling pathways, stress activated kinase JNK, FOXO3, cortisol, estrogens, nitric oxide, etc.) [1]. Many studies indicate direct interactions between tetracyclic terpenoids and corticosteroid and estrogenic receptors.

A characteristic feature of adaptogens is that they act as eustressors (i.e. “good stressors”), and as mild stress mimetics or ‘stress-vaccines’ that induce a stress-protective response. Adaptogens exhibit multitarget action and the shared use of several different receptors, including receptors for corticosteroid, mineralocorticoid, progestin, estrogen, serotonin, NMDA, and nicotinic acetylcholine, receptor tyrosine kinases, and many G-protein coupled receptors [1]. Therefore, the possibility that numerous molecular network interactions (with feedback regulation of the neuroendocrine and immune systems) contribute to the overall pharmacological response and result in agonist-dependent antagonism is most suitable for understanding the mechanisms of action of adaptogens [1].

The mechanisms of action of adaptogens are “specifically” related to stress-protective activity and increased adaptability of the organism [1]. Molecular targets, signaling pathways, and networks common to adaptogens are associated with chronic inflammation, atherosclerosis, neurodegenerative cognitive impairment, metabolic disorders, and cancer, all of which are more common with age [1]. Current and potential use of adaptogens in pharmacotherapy is related to the treatment of mental diseases and behavioral disorders, such as depression, anxiety, bipolar disorder, stress-induced fatigue, and cognitive function. Their prophylactic use by healthy subjects (pharmacosanation) to reduce the negative effects of stress and for prevention of age-related diseases is justified.

**Conclusion:** The mechanisms of action of adaptogens are “specifically” related to stress-protective activity and increased adaptability of the organism [1]. It is very unlikely that the pharmacological activity of any phytochemical is specific and associated only with one type of receptor, particularly adaptogenic compounds, which affect key mediators of the adaptive stress response at intracellular and extracellular levels of communication [1].

**Reference**

1. Panossian AG, 2017. Understanding adaptogenic activity: specificity of the pharmacological action of adaptogens and other phytochemicals. Ann NY Acad Sci. 22 Jun 2017. http://onlinelibrary.wiley.com/doi/10.1111/nyas.13399/full.

## 10 Tocotrienols in bone protection: from animals to humans

### Chwan-Li (Leslie) Shen

#### School of Medicine, Texas Tech University Health Sciences Center, Lubbock, Texas, USA

##### **Correspondence:** Chwan-Li (Leslie) Shen

*Journal of Chinese Medicine* 2018, **13(Suppl 1):**10

**Background:** Osteoporosis, a degenerative bone disease, is characterized by low bone mass and microstructural deterioration of bone tissue that results in bone fragility and an increased susceptibility to fractures. The trend of increased life expectancy is accompanied with an increase in the prevalence of osteoporosis and concomitant complications in the elderly population.

**Results:** Epidemiological evidence has shown an association between vitamin E consumption and the prevention of age-related bone loss in elderly women and men. Animal studies show that ingestion of vitamin E, especially tocotrienols, may benefit bone health in terms of maintain higher bone mineral density and improving bone microstructure and quality. The beneficial effects of tocotrienols on bone health appear to be mediated via anti-oxidant/anti-inflammatory pathways and/or 3-hydroxy-3-methylglutaryl coenzyme A (HMG CoA) mechanisms. This presentation will cover the following (i) overview of prevalence and etiology of osteoporosis, (ii) types of vitamin E (tocopherols vs. tocotrienols), (iii) findings of tocotrienols and bone health: cellular, animal and human studies, (iv) beyond tocotrienols and bone health, (v) challenges and limitation, and (vi) future translational research direction.

**Conclusion:** Existing preclinical studies provide a certain level of evidence suggesting that tocotrienols-mediated protection mitigates bone loss and improves healing of bone fractures. However, these studies are largely preliminary, suggestive, and far from being clinically relevant or meaningful. Furthermore, there is still limited evidence supporting the association between alleviated decrease or even increase of BMD and anti-osteoporotic effect of tocotrienols in humans.

## 11 Flavonoid action on ion transporters and channels: its physiological roles

### Yoshinori Marunaka

#### Department of Molecular Cell Physiology, Kyoto Prefectural University of Medicine, Kyoto 602-8566, Japan

##### **Correspondence:** Yoshinori Marunaka

*Journal of Chinese Medicine* 2018, **13(Suppl 1):**11

**Background:** Flavonoids have multiple potential to control various cell functions in our body. Recently cytosolic Cl- has been shown to regulate blood pressure, neurite elongation, and epithelial water secretion. Flavonoids have stimulatory actions on Cl- transporter, Na+ –K+ –2Cl– cotransporter 1 (NKCC1; an isoform of NKCC), elevating the cytosolic Cl- concentration. In this talk, based on stimulatory actions of flavonoids on NKCC1, I introduce the molecular mechanism of quercetin on: (1) blood pressure, (2) neurite elongation, and (3) epithelial Cl- secretion associated with water secretion.

**Results and conclusions:** (1) Flavonoids increase the cytosolic Cl– concentration by activating NKCC1, showing an anti-hypertensive action via diminution of epithelial Na+ channel (ENaC) expression. (2) Flavonoids stimulate neurite elongation via elevation of the cytosolic Cl– concentration by activating NKCC1 due to tubulin polymerization through Cl–induced inhibition of GTPase. (3) In lung airway epithelia, via NKCC1 activation, flavonoids increase the driving force for Cl– secretion through elevation of the cytosolic Cl– concentration associated with water secretion, participating in prevention of our body from bacterial and viral infection.

## 12 In vitro and in vivo studies on digestion and fermentation of polysaccharides from seeds of *Plantago asiatica* L. with its beneficial effects on intestinal health

### Shaoping Nie, Jielun Hu, Junyi Yin, Mingyong Xie

#### State Key Laboratory of Food Science and Technology, Nanchang University, Nanchang 330047, China

##### **Correspondence:** Shaoping Nie

*Journal of Chinese Medicine* 2018, **13(Suppl 1):**12

**Background:** Functional mechanism of polysaccharide is still largely unknown and need detailed investigation. Here, a systematic research model was established for in vitro and in vivo studies on the digestion and fermentation of the polysaccharide from seeds of *Plantago asiatica* L. and its beneficial effects on intestinal health.

**Materials and methods:** Saliva, gastric, small intestinal digestion, and large bowel fermentation of polysaccharide from *P. asiatica* L. were analyzed by simulated human digestion and fermentation system in vitro and mouse model in vivo. In vitro physiology effects of this polysaccharide (glucose diffusion, activities of various digestive enzymes and the absorption of cholesterol and bile acids) were also investigated in simulated intestinal tract. In addition, different mouse models were also established for evaluating the effects of this polysacchairde on intestinal functions in vivo. Researches were also carried out on whether oral administration and pre-administration of the polysaccharide could prevent or treat the development and colon microbiota changes in mice with dextran sodium sulfate (DSS)-induced experimental colitis.

**Results:** It was found that salivary amylase had no effect on the polysaccharide; however, the polysaccharide was influenced in later gastrointestinal digestion. A steady decrease in molecular weight was observed as digestion time increased. The polysaccharide was also physiologically active for human large bowel, and its carbohydrate composition determined its SCFA production. It could have notable influence on slowing down glucose diffusion and inhibiting α-amylase activity, pancreatic lipase and protease activities. In addition, the polysaccharide was able to bind bile acids and may reduce cholesterol level. Interestingly, it was found the polysaccharide intake could lower the apparent absorption of lipid in mice. *Bacteroides* sp., *Eubacterium* sp., butyrate-producing bacteria *Butyrivibrio* sp., and probiotics *Bifidobacterium bifidum*, *Lactobacillus fermentum*, and *Lactobacillus reuteriin* in mouse colon were all increased after polysaccharide intake. For mice with experimental colitis, the polysaccharide could prevent and treat the development and colon microbiota changes.

**Conclusions:** Our research could provide some information for the digestion and fermentation of polysaccharide from *P. asiatica* L., and may provide the models for the methods of evaluating the digestion and fermentation of other complex carbohydrates. It could also provide some meaningful information for functional mechanism of this polysaccharide, which makes the polysaccharide a potential ingredient in functional food applications.

## 13 Devising a ‘Sweet’ natural tool for therapeutic applications

### Qiu Li, Yiming Niu, Chunming Wang

#### State Key Laboratory of Quality Control in Chinese Medicine, Institute of Chinese Medical Sciences, University of Macau, Macau SAR, China

##### **Correspondence:** Chunming Wang - cmwang@umac.mo

*Journal of Chinese Medicine* 2018, **13(Suppl 1):**13

**Background:** Carbohydrate molecules, including polysaccharides, glycosaminoglycans and small molecule carbohydrates, play diverse and important roles in a wide range of physiological and pathological processes. Designing carbohydrate-based therapeutic agents to promote the activity of certain growth factors (GFs) may provide a new approach to solve unmet medical challenges.

**Materials and methods:** We obtained an acidic polysaccharide fraction (EUP3) from the bark of *Eucommia ulmoides*, among a series of natural Chinese medicinal herbs [1]. We performed a series of binding affinity assays for growth factors (such as ELISA and SPR) and confirmed the affinity of EUP3 for binding FGF-2 and PDGF-BB, two key cytokines of blood vessel formation and maturation, respectively.

**Results:** EUP3 polysaccharide is a naturally derived acidic polysaccharide with affinity for pro-angiogenic GFs, especially PDGF-BB, which is under investigation as a bioactive component for wound healing.

**Conclusions:** In summary, we demonstrated a class of polysaccharide tool developed in our laboratory with specific focus. This carbohydrate material with specific features exhibit unique and useful functions that may have an impact in tissue regenerative applications (Fig. 1).

**Fig. 1:**
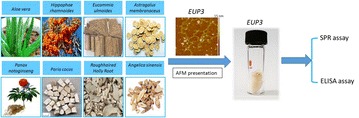
The discovery of EUP3 polysaccharide

**Acknowledgements:** We thank funding grants from the University of Macau (MYRG2015-00160-ICMS-QRCM; MYRG2016-00031-ICMS-QRCM) and the National Natural Science Foundation of China (NSFC 51503232).

**Reference**

1. Li Q, Guo G, Meng F, Wang HH, Niu Y, Zhang Q, Zhang J, Wang Y, Dong L, Wang C. A Naturally Derived, Growth Factor-Binding Polysaccharide for Therapeutic Angiogenesis. ACS Macro Letters. 2016; 5(5):617–21.

## 14 Plant biodiversity: phytochemicals and health

### Pinarosa Avato

#### Dipartimento di Farmacia-Scienze del Farmaco, Università degli Studi di Bari Aldo Moro, via E. Orabona 4, Bari, Italy

##### **Correspondence:** Pinarosa Avato - pinarosa.avato@uniba.it

*Journal of Chinese Medicine* 2018, **13(Suppl 1):**14

**Background:** Plant biological diversity represents a highly valuable heritage to be protected with care and to be appreciated for its numerous ecological implications as well as for its industrial importance. The plant kingdom, due to its biological diversity, constitutes in fact a rich source of high quality molecules which are used in agriculture, veterinary, pharmacy and medicine with various applications.

Biological diversity of plants also relies on chemical diversity mainly deriving from their secondary metabolism. Plants synthesize and accumulate in their organs a large number of highly specialized metabolites, the so called secondary metabolites, which possess a wide range of different chemical structures that result from plant evolution and are the molecular basis of heredity. These secondary metabolites are specific to an individual species thus contributing to define the plant species distinctiveness.

Phytochemicals are responsible for the plant ecological properties and are required for the plant-environment interactions. In addition, many of them display important pharmacological properties.

**Results and conclusions:** In these recent years, the growing interest in using plant metabolites to treat diseases in humans and animals and the high request of health products originating from natural sources rather than synthetic has contributed to revive the research towards the study of plant biodiversity with the aim to identify new bioactive molecules and/or phytochemicals which may serve as leads to develop new health products or innovative drugs.

The present communication will provide a selection of botanical species with phytopharmaceutical importance and highlight the chemical polymorphism deriving from the plant biodiversity along with its implications on bioactivity. Based on our study on the chemical and biological characterization of rare or less studied plant species, discussion will include examples of wild growing species as well as of cultivated plant species. Their importance in the promotion of a good health and in the development of new pharmacological applications will be illustrated and commented.

## 15 The bio-activities of the novel steroids from *Epigynum* Wight

### Jian-Xin Cao, Gui-Guang Cheng

#### Yunnan Institute of Food Safety, Kunming University of Science and Technology, Kunming, 6505000, People’s Republic of China

##### **Correspondence:** Jian-Xin Cao - jxcao321@hotmail.com

*Journal of Chinese Medicine* 2018, **13(Suppl 1):**15

**Background:** An ideal plant system is of great significance to reveal the mystery of the plant kingdom, and drug discovery [1]. Apocynaceae and Asclepiadaceae are two important families in the flora. Apocynaceae directly and progressively changes in the evolution to Asclepiadaceae. Epigyneae is at the highest position in the evolution system of Apocynacea. *Epigynum* Wigth belongs to Epigyneae—the evolutionary highest family, therefore, it is a genus at a junction status evolution to Asclepiadaceae from Apocynaceae. *Epigynum* auritum is the only species distributed in China in the *Epigynum* Wight of Epigyneae [2, 3]. The chemical perspective of *E.* auritum remains largely unknown. We chose the species to research their chemical constituents and try to find some clues in the taxonomy.

As we know, cardiac glycoside is the important bioactive constituent of Apocynaceae, and pregnane is the main constituent of Asclepiadaceae. But in our preliminary studies on *E. auritum*, we discovered three kinds of novel steroid natural products with deformed or degraded skeletons, they neither belong to cardiac glycoside, nor belong to traditional pregnane. And *E. auritum’s* kin—*E.* cochinchinensis, mainly distributed in Laos, was also found including same types of steroid natural products. The structures of compounds from *E. auritum* and *E. cochinchinensis* were elucidated as follow (Fig. 1) [4, 5, 6]:

**Fig. 1:**
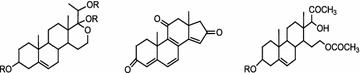
See text for description

**Result:** Our studies also revealed that the novel androstane derivatives from *E.* auritum displayed significant immunosuppressive activities. Remarkably, the activity of androsta-4,6,8(9),14(15)-tetraene-3,11,16-trione was found similar to standard immunosuppresive agents tritolide and dexamethasone [7, 8].

According to the result of the previous research, an autoimmune disease is a multi-system involvement condition arising from an abnormal immune response producing a variety of autoantibodies on the base of the activation of T and B cells. Lymphoma is a malignant lymphocytes tumor, while lymphocytes (e.g., B, T and NK cells) undertake the body’s immune system. The acute lymphoblastic leukemia (ALL) is also a cloned disease involving the malignant proliferation of the precursor of T and B lymphocytes. Therefore, both autoimmune diseases and ALL are closely related to T and B lymphocytes, and the main drugs are the glucocorticoids (GCs) currently used in clinical therapies. ALL and lymphoma cell proliferation inhibition experiment of novel androstane derivatives were evaluated with dexamethasone as a positive control [9, 10, 11, 12]. Our research results showed that androstane derivatives significantly inhibited MOLT4 and SR cell proliferation, which dexamethasone has showed resistance for these two cell lines. Therefore, the androstane derivatives can be considered as a new potential clinical drugs for overcoming the resistance of dexamethasone, and worth to further study.

**Conclusions:** In the future, we will study the structure–activity relationship of the androstane compounds isolated from *Epigynum* plants and its derivatives with the inhibition of lymphatic hyperplastic tumor cell proliferation, further verificate the inhibition on the primary cell proliferation for searching the potential new drugs overcoming GCs resistance in lymphatic hyperplastic tumor treatment.

**References**

1. Zenyi W, et al. Xinhua medica outline, Book Two, Shanghai Scientific and Technical Publishers; 1991. pp. 403–6.
2. Ying J, et al. Flora of China, vol 63, China Science and Technology Press; 1977. pp. 245–6.
3. Bintao L, et al. Guihuaia. 1982; 2(4):165–70.
4. Cao JX, et al. Tetrahedron. 2005; 61(27):6630–3.
5. Gao F, *Cao JX, *Cheng GG. Nat Prod Res. 2016; 31(9):1102–5.
6. Gao F, *Cao JX, *Cheng GG. Fitoterapia. 2017. 10.1016/j.fitote.2017.02.011.
7. Jianxin C, et al. Chinese Patent. 2014.09 (authorization), 201310059903.2
8. Jianxin C, et al. Chinese Patent, 2014.09 (authorization), 201310059966.8.
9. Hulleman E, et al. Blood. 2009; 113(9):2014–21.
10. Hossain T, et al. J Paediatr Surg Bangladesh. 2016; 6(1):3–9.
11. Pépin AJ, et al. Crit Rev Oncol Hematol. 2016; 107:138–48.
12. Holleman A, et al. N Engl J Med. 2004; 351(6):533–542.

## 16 Conserved transcription factors regulating defense chemistry in plants; jasmonate-responsive ERF transcription factors involved in alkaloid regulation in Solanaceae crops

### Tsubasa Shoji

#### Graduate School of Biological Sciences, Nara Institute of Science and Technology, Nara, Japan

##### **Correspondence:** Tsubasa Shoji

*Journal of Chinese Medicine* 2018, **13(Suppl 1):**16

**Background:** To cope with threats imposed by pathogens and pests, plants accumulate toxic substances, including a diverse array of alkaloids, terpenoids, and other metabolites with bioactive properties. Steroidal glycoalkaloids (SGAs) and nicotine are specialized metabolites found in certain species of Solanaceae family. A group of jasmonate-responsive ERF transcription factors, of which genes form tandem clusters in relevant genomes, regulate production of SGAs in tomato and potato [1, 2] and that of nicotine in tobacco [3].

**Results:** Altered expression of ERFs causes the drastic changes of SGA or nicotine accumulation and expression of metabolic and transport genes involved in long multi-step routes leading to the end products, including upstream primary pathways. Promoter-binding studies demonstrate that ERF factors up-regulate the transcription of the downstream structural genes. Such transcriptional regulation is corroborated with frequent occurrence of cognate cis-regulatory elements for the factors in the promoter regions. A loss-of-function mutation of JRE4, one of the clustered ERF genes from tomato, causing a substitution of one amino acid residue critical for DNA-binding activity of the factor, results in marked reduction of SGA accumulation and associated gene expression in the species, supporting a predominant role of JRE4 in the SGA regulation.

**Conclusions:** The functional conservation and diversity of ERF factors and their possible contribution for the evolution of networks with co-regulated metabolic genes will be discussed.

**References**

1. Cárdenas. Nat Comm. 2016; 7:10654.
2. Thagun, et al. Plant Cell Physiol. 2016; 57:961–75.
3. Shoji T, Kajikawa M, Hashimoto T. Plant Cell. 2010; 22:3390–409.

## 17 Anticancer potential of flavones

### Randolph R.J. Arroo^1^, Didem Şöhretoğlu^2^, Demetrios A. Spandidos^3^, Vasilis P. Androutsopoulos^3^

#### ^1^Leicester School of Pharmacy, De Montfort University, Leicester, UK; ^2^ Faculty of Pharmac,y Hacettepe Unıversıty, Ankara, Turkey; ^3^Department of Toxicology, Medical School, University of Crete, Heraklion, Greece

##### **Correspondence:** Randolph R.J. Arroo

*Journal of Chinese Medicine* 2018, **13(Suppl 1):**17

**Background:** Many papers have been written on the anticancer properties of dietary flavonoids, and a range of potential mechanisms of action of flavonoids. However, most dietary flavonoids—notably polyphenolic flavonoids—have very poor ADME properties, and the levels necessary to stop growth of tumour cells cannot be sustained in a human body trough dietary intake alone. At present no flavonoid based drugs are clinically used in cancer therapy. Thus, whereas epidemiological and pre-clinical data seem to indicate a high potential for flavonoids, from the point of view of the pharmaceutical industry and drug developers, they are considered poor candidates.

**Results:** The flavones—which constitute a subgroup of the flavonoids—show some structural analogy with oestrogen (Fig. 1) and are known to interact with human oestrogen receptors [1], either as agonist or as antagonist. They are classed as phytoestrogens, and may play a role in cancer prevention through a mechanism of action [2] possibly similar to that of the clinically used medication tamoxifen.

**Fig. 1:**
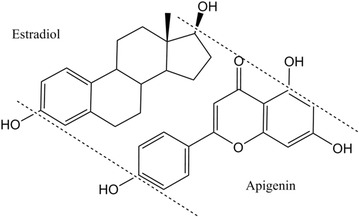
Structural similarity between estradiol and the flavone apigenin

**Conclusion:** Flavones are abundantly present in common fruits and vegetables, many of which have been associated with cancer prevention [3]. Their phytoestrogen activity makes that they can assert their biological action at concentrations that are realistically achievable in the human systemic circulation.

**References**

1. Arroo RRJ, Beresford KJM, Bhambra AS, Boarder MR, Budriesi R, Cheng Z, Micucci M, Ruparelia KC, Surichan S, Androutsopoulos VP. Phytoestrogens as natural prodrugs in cancer prevention:towards a mechanistic model. Phyt. Rev. 2014; 13:853–66
2. Cheng Z, Surichan S, Ruparelia K Arroo R, Boarder M Tangeretin and its metabolite 4´-hydroxytetramethoxyflavone attenuate EGF-stimulated cell cycle progression in hepatocytes; role of inhibition at the level of mTOR/p70S6K. Br J Pharmacol. 2011; 162:1781–1791.
3. Shukla S, Gupta S. Apigenin: a promising molecule for cancer prevention. Pharm Res. 2010; 27:962–78.

## 18 Interaction of medicinally active dietary plant flavonoids and nano-vehicles for drug delivery: insights from “two color” fluorescence and related studies

### Pradeep K. Sengupta

#### Department of Biophysics, Molecular Biology and Bioinformatics, University of Calcutta, 92 Acharya Prafulla Chandra Road, Kolkata 700009, India

##### **Correspondence:** Pradeep K. Sengupta - pradeepsinp@yahoo.co.in

*Journal of Chinese Medicine* 2018, **13(Suppl 1):**18

**Background:** Flavonols and related polyphenolic compounds of the flavonoid group are ubiquitous in higher plants, and abundant in common plant based food and beverages (such as citrus fruits, berries, onion, broccoli, soy products, tea and red wine). These phytochemicals have been the focus of enormous recent attention as nutraceuticals which are active against various free radical mediated and other human diseases, especially cancers, cardiovascular ailments, diabetes, atherosclerosis, and neurodegenerative disorders Their high potency and low cytotoxicity make them viable alternatives to conventional therapeutics [1]. In this context, the question of the intracellular targets of flavonoids, the mode of interaction with such targets, and the quest for suitable ‘nano- vehicles’ for drug delivery (which are capable of enhancing the solubility and bioavailability of these nutraceuticals) have been key areas of explorations [1–2]. On a different scenario, from spectroscopic perspectives, flavonols (which are the most commonly occurring dietary flavonoids) have come into much recent prominence as prototype molecules exhibiting ultrafast proton transfer reactions following light absorption, which leads to exquisitely sensitive ‘two color’ fluorescence (comprising blue–violet and yellow–green emission bands (Fig. 1).

**Fig. 1:**
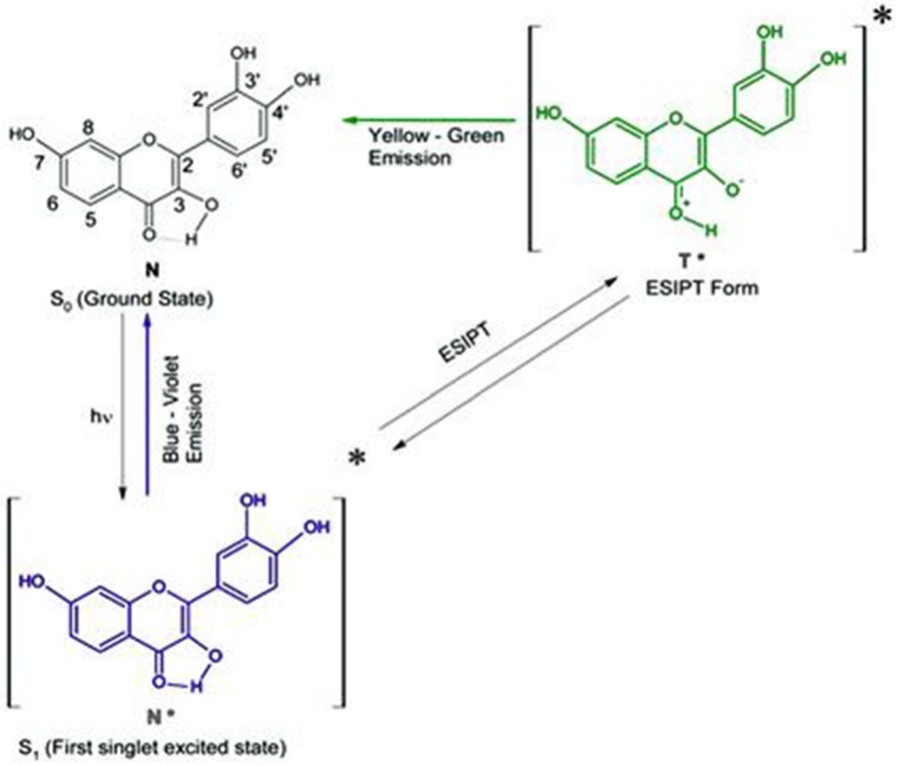
Excited state intramolecular proton transfer (ESIPT) mechanism underlying ‘two color’ fluorescence behavior of flavonols, illustrated for the dietary plant flavonol FISETIN [see Ref. 2]

The relative intensities between the two colors, and other important emission parameters (such as emission maxima, lifetime, and anisotropy) are strongly modulated by the local environment of the target binding site(s). This has opened up interesting opportunities for using flavonols as their ‘own fluorescence reporters’ for noninvasing sensing of their interactions with various intra-cellular targets (encompassing proteins, duplex and quadruplex DNA, and biomembranes) [2–8], as well as ‘nano-vehicles’ for drug delivery [9].

**Results:** Representative and recent studies exemplifying applications of ‘two color’ fluorescence of typical dietary flavonols, such as fisetin and quercetin are highlighted and reviewed in this lecture. This is based on research activities in our laboratory during the past 15 years, from 2001 till date [2–9], and other related work.

Upon binding to various bio-relevant targets (encompassing carrier proteins, biomembranes, duplex and quadruplex DNA), and cyclodextrin based nano-vehicles for drug delivery, dramatic changes are noted in the two color emission profiles of representative flavonols we examined. In particular, we observed spectacular enhancement of the yellow-green ESIPT tautomer emission yield, as well as drastic changes in anisotropy, lifetime, and rotational correlation time upon binding to such broad range of receptors. Simultaneously, for fisetin and other similar structurally related flavonols, for which the normal fluorescence exhibits strong ‘charge transfer’ character, significant blue shifts are noted in the maxima of the normal (non proton transferred)fluorescence band, signifying relatively apolar binding environments. With regard to encapsulation in cyclodextrin nano-cavities, the role of cavity size, and chemical modifications to the parent cyclodextrins, crucially determine encapsulation capabilities [9]. These are clearly evident from differences in the fluorescence emission profiles, tautomer/normal emission intensity ratio, anisotropy and other emission parameters. The occurrence of FRET from trp-214 of human serum albumin (HSA) to flavonol molecules bound to the protein matrix are clearly evident from both fluorescence emission and excitation profiles. This indicates that the flavonol binding sites in HSA are proximal to unique tryptophan moiety of the protein. (trp-214) [3]. Studies on interactions of dietary flavonols with quadruplex DNA reveal that flavonols are useful quadruplex DNA ligands [4–5], and thus can be potentially useful anti-cancer drugs through stabilization of quadruplex DNA structure. The intrinsic fluorescence emission of flavonols also prove to be useful in exploring binding of flavonols to model and natural biomembranes, both in terms of locating the environments of the flavonols in membranes (from the two color fluorescence emission signatures) and obtaining quantitative estimates of partition coefficients, and phase transition temperatures for liposomal membranes [2]. Supporting studies via other relevant spectroscopic techniques, especially induced circular dichroism (ICD) and molecular modeling are also briefly reviewed, and such studies confirm and consolidate the fluorescence based findings. Such complementary studies provide additional insights regarding the binding processes, including thermodynamics and conformational aspects, together with details regarding the dominant noncovalent interactions involved. Implications of such findings are assessed in relation to the promising prospects of flavonoid based drug discovery and development.

**Conclusions:** 1. The exquisitely sensitive intrinsic ‘two color’ fluorescence of medicinally active dietary flavonols offers interesting possibilities for using favonols as their ‘own fluorescence reporters’ for non-invasive sensing of their interactions with various intra-cellular targets relevant to the therapeutic actions of flavonols (encompassing proteins, duplex and quadruplex DNA, and biomembranes), as well as their encapsulation in natural and chemically modified ‘nano-vehicles’ for drug delivery.

2. It is evident from such studies that the structure and substitution patterns of flavonols play a crucial role in determining their binding modes, and relative affinities with their receptors.

3 We can envision that. expanded use of the intrinsic fluorescence of dietary flavonols via spectroscopy and microscopic studies can emerge as a valuable approach in drug discovery and development endeavors aimed towards screening of the most appropriate flavonol derivatives with desired medicinal properties, from among numerous structural variants available in nature.

**References**

1. Havsteen, BH. Pharmacol. Therapeut. 2002; 96:67–202.
2. Sengupta, PK. Pharmacologically active plant flavonols as proton transfer based multiparametric probes targeting biomolecules. reviews in fluorescence 2016. Geddes CD, editor. Chap. 4. Springer internat; 2017, and references cited therein
3. Chaudhuri S, Sengupta B, Taylor J, Pahari B, Sengupta, PK. Curr Drug Metab. 2013; 14:491–503.
4. Sengupta B, Pahari B, Blackmon L, Sengupta, PK. PLoS ONE, 2013; 8:e65383. doi: 10.1371.
5. Bhattacharjee S, Chakraborty S, Sengupta PK, Bhowmik S. J Phys Chem. 2016; 120:8942–52.
6. Pahari B, Chaudhuri S, Chakraborty S, Sengupta PK. J Phys Chem. 2015; 119:2533–45.
7. Pahari B, Chakraborty S, Sengupta B, Chaudhuri S, Martin W, Taylor J, Henley J, Davis B, Biswas PK, Sharma AK, Sengupta PK. Food Nutr Sci. 2013; 83–92.
8. Davis B, Biswas PK, Sharma AK, Sengupta PK. Food Nutr Sci. 2013; 83–92. Pahari
9. Sengupta BB, Chakraborty S, Thomas B, McGowan D, Sengupta PK. J Photochem Photobiol. B Biol. 2013; 118:33–41, and references cited therein.

## 19 Screening for topoisomerases I inhibitors from herbal medicines combining bioaffinity ultrafiltration and LC–MS

### Gui-Lin Chen^1,2^, Ming-Quan Guo^1,2^

#### ^1^Key Laboratory of Plant Germplasm Enhancement and Specialty Agriculture, Wuhan Botanical Garden of Chinese Academy of Sciences, Wuhan, 430074, China; ^2^The Sino-Africa Joint Research Center, Chinese Academy of Sciences, Wuhan 430074, China

##### **Correspondence:** Ming-Quan Guo - guomq@wbgcas.cn

*Journal of Chinese Medicine* 2018, **13(Suppl 1):**19

**Background:** In the early drug discovery stage, the binding affinity between small molecule candidates and its therapeutic bio-molecule targets is considered as the primary determinant of the candidate’s biological activity [1]. Recently, it has been found that almost half of the small-molecule drugs in the market are enzyme inhibitors according to a recent survey [2]. Topoisomerase I (Topo I), a well recognized therapeutic target for a variety of cancers like ovarian, lung, and colorectal cancer, has gained increasing interest since Topo I inhibitors have shown good anticancer activity, and some Topo I inhibitors have already been approved by FDA like camptothecin (CPT) type inhibitors including topotecan and irinotecan. However, these inhibitors suffered from lactone instability and their effects are rapidly reversible. To circumvent these limitations, novel Topo I inhibitors have been actively sought, especially new natural inhibitors with less side-effect [3, 4]. In this context, we strived to combine affinity ultrafiltration with LC/MS (UF-LC/MS) aiming to fish out specific bioactive compounds targeting Top I from some medicinal plants of interest.

**Materials and methods:** DNA topoisomerase I (*E. coli*) was obtained from New England Biolabs (NEB, Ipswich, Massachusetts, USA), and the centrifugal ultrafiltration filters (YM-30, 30 kDa) were purchased from Millipore Co. Ltd (Bedford, MA, USA). The ultrafiltration experiment was conducted according to our previous work [5, 6]. As for the herbal plants of interest in this work, the bulbs of Lycoris radiata were obtained from Wuhan Botanical Garden, and the barks of Rhamnus davurica Pall were from Jikang pharmaceutical Co., Ltd. (Hebei, China). The two human cancer cell lines (Hep G2 and HT-29) were obtained from China Center for Type Culture Collection (CCTCC, Wuhan, China). At last, a Thermo Accela 600 HPLC system coupled with a TSQ Quantum Access MAX mass spectrometer (Thermo Fisher Scientific, San Jose, CA, USA) was used for the analysis of samples before and after ultrafiltration.

**Results:** The application of UF-LC/MS strategy leads to some very interesting findings, and several new types of natural compounds from different medicinal plants were successfully screened out and tested to markedly inhibit Topo I as compared with CPT. In more details, an alkaloid from Lycoris radiata was screened out, and exhibited good dose-dependent inhibition against Topo I with IC50 at 7.25 ± 0.20 μg/mL comparable to CPT at 6.72 ± 0.23 μg/mL; and also strongly inhibited the proliferation of HT-29 and Hep G2 cells in an intuitive dose-dependent manner with the IC50 values at 3.98 ± 0.29 μg/mL and 11.85 ± 0.20 μg/mL, respectively. In another effort, some flavonoids like compounds 3 and 4 against Topo I screened out from Rhamnus davurica using UF-LC/MS showed markedly inhibition to proliferation of Hep G2 cells with IC50 values at 10.20 ± 0.24 and 4.78 ± 0.28 μg/mL, respectively.

**Conclusions:** The UF-LC/MS using Topo I as a target enzyme proved to be an efficient and alternative approach for the rapidly screening and identification of natural-origin Topo I ligands from some medicinal plants of interest, the bioactive compounds against Topo I could be fished out, and identified from complex plant extracts. Thus, The UF-LC/MS method has shown great potential as a powerful tool for the discovery of novel Topo I inhibitors in a high throughput manner.

**Acknowledgements:** This work was jointly supported by the Natural Science Foundation of China (Grant No. 81673580 to M. Guo), “the One Hundred Talents Project” from Chinese Academy of Sciences (Grant No. 29Y429291a0129 to M. Guo), and the Sino-Africa joint research project (Grant No. SAJC20160233 to M. Guo). These funders played no roles in the study design, data collection and analysis, and decision to publish.

**References**

1. Qin SS, Ren YR, Fu X, Shen J, Chen X, Wang Q, Bi X, Liu WJ, Li LX, Liang GX, Yang C, Shui WQ. Multiple ligand detection and affinity measurement by ultrafiltration and mass spectrometry analysis applied to fragment mixture screening. Anal Chim Acta. 2015; 886:98–106.
2. Shu YS, Peng MJ, Zhang YP, Peng S. Combination of preparative HPLC and HSCCC methods to separate phosphodiesterase inhibitors from Eucommia ulmoides bark guided by ultrafiltration-based ligand screening. Anal Bioanal Chem. 2013; 405:4213–4223.
3. Xiao S, Yu RR, Ai N, Fan XH. Rapid screening natural-origin lipase inhibitors from hypolipidemic decoctions by ultrafiltration combined with liquid chromatography-mass spectrometry. J Pharmaceut Biomed Anal. 2015; 104:67–74.
4. Zhu HB, Liu S, Li X, Song FR, Liu ZQ, Liu SY. Rapid screening natural-origin lipase inhibitors from hypolipidemic decoctions by ultrafiltration combined with liquid chromatography-mass spectrometry. Anal Bioanal Chem. 2013; 405:7437–7445.
5. Chen, GL, Tian YQ, Wu JL, Li N, Guo MQ. Antiproliferative activities of Amaryllidaceae alkaloids from Lycoris radiata targeting DNA topoisomerase I. Sci. Rep. 2016; 6:38284.
6. Chen GL, Guo MQ. Screening for natural inhibitors of topoisomerases I from Rhamnus davurica by affinity ultrafiltration and high-performance liquid chromatography–mass spectrometry. Front Plant Sci. 2017; 8:1521–1531

## 20 The function and molecular mechanism of 1-deoxynojirimycin from mulberry leaves

### Kiran Thakur, Zhang Fang, Xue-Qin Hu, Zhao-Jun Wei

#### School of Food Science and Engineering, Hefei University of Technology, Hefei 230009, People’s Republic of China

##### **Correspondence:** Zhao-Jun Wei - zjwei@hfut.edu.cn

*Journal of Chinese Medicine* 2018, **13(Suppl 1):**20

**Background:** Mulberry 1-deoxynojirimycin (DNJ), a imino suagr known as glucose analog is gaining considerable interest due to its multi-faecetd health benefits. Previous studies claimed that DNJ possessed high α-glucosidase inhibition, antioxidant, antimicrobial, anti-inflammatory activity, inhibition of adipogenesis as well as protection from age-related behavioral. Thus, the present work plan was executed to evaluate the anti-diabetic potential of DNJ with a objective of restoring urinary metabolite change in *db/db* mice model.

**Materials and methods:** In this study, urinary metabolomics method of type 2 diabetic *db/db* mice based on LC–MS was employed to represent the metabolic characteristics of T2DM followed by DNJ supplementation to assess the biochemical and histopathological parameters. Metabolomics was applied to LC–MS metabolic urinary of type 2 diabetes (T2DM) mice treated with mulberry 1-deoxynojirimycin (DNJ). Principal component analysis (PCA), partial least squares discriminant analysis (PLS-DA), and one-way analysis of variance (ANOVA) were applied to directly demonstrate the changed profiles of the treated mice groups.

**Results:** After DNJ administration, serum biochemical indicators related to T2DM like blood glucose, triglyceride, total cholesterol, nitrogen, malondialdehyde and creatinine decreased significantly. Histopathological changes in liver cells were marked by deformations and disruptions in central area of nuclei in DM mice. DNJ treatment recovered regular liver cells with normal nuclei. LC–MS data acquistion showed that the metabolites of T2DM were significantly different from healthy controls in the bulk data generated. The level of 16 metabolites showed that the diabetic group was closer to the healthy group as the DNJ treatment time prolonged. DNJ inhibited the activity of glucosidase on glucose metabolism, but also affected lipid and amino acid metabolism.

**Conclusions:** The metabolic profiling in urine of mice treated with DNJ provided essential hints to intervene with pathophysiology of T2DM which can also be useful as a source of novel T2DM-associated biomarkers. Present results also provided a reference for the underlying anti-diabetic mechanism of DNJ which may offer deep insights into the potent metabolite biomarkers of the applied anti-diabetic interventions.

## 21 Network pharmacology studies on the bioactive compounds and action mechanisms of natural products for the treatment of diabetes mellitus

### Weiwei Li, Guoqi Yuan, Yuxiang Pan, Cong Wang, Haixia Chen

#### School of Pharmaceutical Science and Technology, Tianjin University, Tianjin, P. R. China

##### **Correspondence:** Haixia Chen - chenhx@tju.edu.cn

*Journal of Chinese Medicine* 2018, **13(Suppl 1):**21

**Background:** Diabetes mellitus (DM) is a kind of chronic and metabolic disease, which can cause a number of diseases and severe complications. It is caused by either the body can’t produce enough insulin or the body can’t effectively respond to the produced insulin. Natural products have played an important role in DM treatment due to multi-components and multi-targets to produce combined or synergistic effects. Network pharmacology approach, integrating network biology and pharmacology, is introduced to study DM, which can combine the drugs, target proteins and disease and form drug-target-disease networks [1]. It has been widely used in the studies of the bioactive compounds and action mechanisms of natural products for the treatment of DM.

**Results:** Natural extracts, polysaccharides and polyphenols are the main components used to DM therapy. Super Natural II, NAPRALERT, Chemical Entities of Biological Interest and DrugBank are the related databases. The action mechanisms related to type 2 DM (T2DM) include α-amylase and α-glucosidase inhibitory, targeting β cell dysfunction, targeting signal pathways [2–8] (AMPK, PI3K/Akt, mTOR, JAK-STAT, ROS-ERK-NF-κB, Wnt and IGF-1 signal pathways) (Fig. 1) and modulation of gut microbiota. T2D-Db, T2DGADB and T2D@ZJU, the databases related to DM, were developed to integrate these information. The high availability of databases provides new opportunities for data integration and can be used to predict the target network of the bioactive constituents. Network pharmacology has been widely applied on the treatment of DM. Based on network pharmacology, the mechanisms of Ge-Gen-Qin-Lian decoction and Tangminling Pills used to the treatment of T2DM were elucidated.

**Fig. 1:**
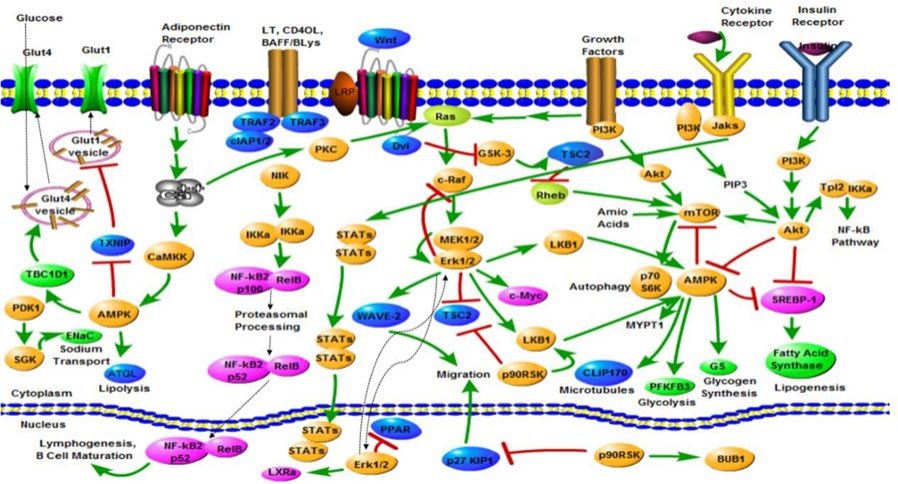
Summary of signal pathways related to diabetes mellitus

**Conclusions:** The appropriate use of network pharmacology may initiate new directions, overcome the disadvantages of current antidiabetic therapies as well as contribute new insights into the discovery of novel antidiabetic drugs.

**Acknowledgements:** Our work was financially supported by the grant from the Natural Science Foundation of China (NSFC 31371879) and National High Technology Research and Development Program (“863” Program) of China (Grant No. SS2013AA100207).

**References**

1. Poornima P, Kumar JD, Zhao Q, Blunder M, Efferth T. Network pharmacology of cancer: from understanding of complex interactomes to the design of multi-target specific therapeutics from nature. Pharmacol Res. 2016; 111:290–302.
2. Kurimoto Y, Shibayama Y, Inoue S, Soga M, Takikawa M, Ito C, et al. Black soybean seed coat extract ameliorates hyperglycemia and insulin sensitivity via the activation of AMP-activated protein kinase in diabetic mice. J Agric Food Chem. 2013; 61:5558–64.
3. Li S, Chen H, Wang J, Wang X, Hu B, Lv F. Involvement of the PI3K/Akt signal pathway in the hypoglycemic effects of tea polysaccharides on diabetic mice. Int J Biol Macromol. 2015; 81:967–74.
4. Siegel N. The mTOR pathway and its role in human genetic diseases. Mutat Res. 2008; 659:284–92.
5. Gurzov EN, Stanley WJ, Pappas EG, Thomas HE, Gough DJ. The JAK-STAT pathway in obesity and diabetes. FEBS J. 2016; 283:3002–15.
6. Liu M, Qin J, Hao Y, Liu M, Luo J, Luo T, et al. Astragalus polysaccharide suppresses skeletal muscle myostatin expression in diabetes:involvement of ROS-ERK and NF-kB pathways. Oxid Med Cell Longev. 2013; 12:782497–782497.
7. Chiang YT, Ip W, Jin T. The role of the Wnt signaling pathway in incretin hormone production and function. Front Physiol. 2012; 3:1–14.
8. Siddle K. Signalling by insulin and IGF receptors: supporting acts and new players. J Mol Endocrinol. 2011; 47:1–10.

## 22 Nutritional interventions to attenuate negative health effects of ambient particulate matter

### Yang Qin, Jingjing Chen, Tao Zhang, Bo Jiang

#### State Key Laboratory of Food Science and Technology, Jiangnan University, Wuxi, Jiangsu, China

##### **Correspondence:** Bo Jiang - bjiang@jiangnan.edu.cn

*Journal of Chinese Medicine* 2018, **13(Suppl 1):**22

**Background:** In recent years, the ambient particulate matter (PM) continues to pose a threat to public health worldwide [1]. Recent research has identified oxidative stress and inflammatory responses as potential features underlying the toxic effects of PM [2]. Sulforaphane (SFN), possessing antioxidant and anti-inflammatory properties, has not been reported in amelioration of oxidative damage induced by PM and warrants further investigations [3, 4]. In this study, protective effects of SFN against oxidative damage incurred by PM2.5 were evaluated.

**Materials and methods:** PM samples were collected by an air sampler in winter in 2016. Samples were then surged with sonication, freeze-dried, weighed and stored at − 20 °C. To obtain PM suspensions, 5 mg PM samples were blended with 1 mL phosphate buffered saline. Adenocarcinomic human alveolar basal epithelial cells (A549) were used as in vitro models. A549 cells were maintained in F-12 K medium supplemented with 10% fetal bovine serum and 1% penicillin/streptomycin at 37 °C in a 5% CO_2_ humidified cell culture incubator.

**Results:** The cell viability of A549 cells which were incubated with PM2.5 dropped in a dose- and time- dependent manner (Fig. 1). After incubation with 100 μg/mL PM2.5 for 48 h, the cell viability of A549 cells approached 57.7% compared to the control group cells. In the following experiments, incubation with 100 μg/mL PM2.5 for 48 h was used to induce damage on A549 cells.

When A549 cells were incubated with more than 6 μmol/L SFN, the cell viability of A549 cells dropped in a time- and dose- dependent manner (Fig. 2). However, there were no discernible differences of cell viability between 0.5, 1, 2, 4 and 6 μmol/L SFN-treated cells and control cells (*p* > 0.05).

As indicated in Fig. 3, after SFN pre-treated for 24 h, the dose–response abolished profiles of SFN on PM2.5-caused cell damage were observed. The cell viability of 6 μmol/L SFN group increased 19.8% versus the positive control group (*p* < 0.01). There were no discernible differences between 6 μmol/L SFN-treated cells and control cells.

**Fig. 1:**
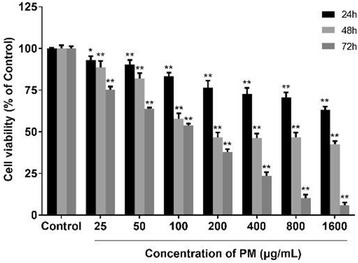
Effects of PM2.5 on the cell viability of A549 cells. Data are presented as mean ± SD of three experiments performed in triplicate. *p < 0.05, **p < 0.01, compared with the control group

**Fig. 2:**
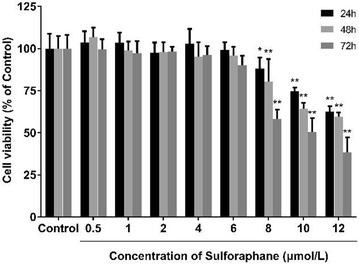
Effects of SFN on the cell viability of A549 cells. Data are presented as mean ± SD of three experiments performed in triplicate. *p < 0.05, **p < 0.01, compared with the control group

**Fig. 3:**
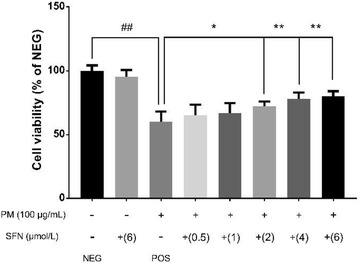
Effects of SFN on the cell viability of A549 cells exposed to PM2.5. Data are presented as mean ± SD of three experiments performed in triplicate. *p < 0.05, **p < 0.01, compared with the control group. (NEG: negative control group, cells without any treatment; POS: positive control group, cells treated with PM2.5 only)

**Conclusions:** In conclusion, our research demonstrated that the addition of SFN ameliorates the PM-induced oxidative damage in A549 cells. This provides the theoretical basis for the nutritional interventions to attenuate negative health effects of ambient particulate matter.

In recent years, the ambient particulate matter which is microscopic solid or liquid matter suspended in the Earth’s atmosphere continues to pose a threat to public health worldwide, especially in developing countries. Epidemiological and toxicological studies have clearly shown that ambient particulate matter not only induces respiratory and cardiovascular impairments, but also contributes to the initiation and progression of diabetes mellitus. Recent research has identified oxidative stress and inflammatory responses as potential features underlying the toxic effects of ambient particulate matter. Reported nutrients related to the reduction of oxidative stress and inflammation are summarized, along with potential nutrients which may ameliorate negative effects of ambient particulate matter. A control strategy in the form of nutritional interventions by adequate intake of functional micronutrients is raised, providing new insights into future studies of the amelioration of negative effects induced by particulate matter.

**References**

1. Kim KH, Kabir E, Kabir S. A review on the human health impact of airborne particulate matter. Environ Int. 2015; 74:136–43.
2. Péter S, Holguin F, Wood LG, Clougherty JE, Raederstorff D, Antal M, Weber P, Eggersdorfer M. Nutritional solutions to reduce risks of negative health impacts of air pollution. Nutrients. 2015; 7(12):10398–416.
3. Chen X, Liu J, Chen SY. Sulforaphane protects against ethanol-induced oxidative stress and apoptosis in neural crest cells by the induction of Nrf2-mediated antioxidant response. Br J Pharmacol. 2013; 169(2):437–448.
4. Greaney AJ, Maier NK, Leppla SH, Moayeri M. Sulforaphane inhibits multiple inflammasomes through an Nrf2-independent mechanism. J Leukoc Biol. 2016; 99(1):189–199.

## 23 Modern Industrial Preparative Chromatography Techniques and Their Applications in the Separation of Natural Products

### Jun Li

#### College of Food Science and Technology, Hebei Normal University of Science and Technology, Qinhuangdao, Hebei 066600, China

##### **Correspondence:** Jun Li - spgcx@163.com

*Journal of Chinese Medicine* 2018, **13(Suppl 1):**23

**Background:** The subject of industrial chemistry of natural product focuses on isolation, purification and fractionation of value-added products, e.g. fine chemicals, natural products, pharmaceuticals, biotechnical products, agrochemicals, aroma and food additives. The demand for products at higher and higher degrees of purity and the greater difficulty of the purification problems encountered (such as the resolution of optical isomers) is more and more forcing the pharmaceutical industry to use Industrial Preparative Chromatography (IPC).

**Results:** The present contribution discusses briefly the situation of the pharmaceutical industry today and its challenges. And the development of IPC separation was enabled by simultaneous developments in stationary phases, chromatography technologies, and computer simulation tools. With practical examples, it illustrates how IPC can compete efficiently with other purification processes like recrystallization and presents some applications.

**Conclusions:** Examples are also given to illustrate various techniques to make the chromatographic process more rugged and stable.

## 24 Dietary polyphenols in maintaining health: a focus on their mechanisms of action

### Maria Daglia^1^, Valeria Curti^1^, Arianna Di Lorenzo^1^, Arold Jorel Tsetegho Sokeng^1^, Seyed Fazel Nabavi^2^, Seyed Mohammad Nabavi^2^

#### ^1^Department of Drug Sciences, Medicinal Chemistry and Pharmaceutical Technology Section, University of Pavia, Italy; ^2^Applied Biotechnology Research Center, Baqiyatallah University of Medical Sciences, Tehran, Iran

##### **Correspondence:** Maria Daglia

*Journal of Chinese Medicine* 2018, **13(Suppl 1):**24

**Background:** A large body of evidence from the past two decades suggests that long-term consumption of dietary polyphenols holds a crucial role in the protection against pathologies induced by oxidative stress and chronic inflammation, such as some forms of cancer, obesity, metabolic syndrome, type 2 diabetes, cardiovascular and neurodegenerative diseases. Polyphenols are the most abundant antioxidants consumed by humans, with a total estimated intake ranging from 1 to 1.2 g/day. 40% of these consist of flavonoids [1–2]. The mechanisms of action at the basis of polyphenol protective activities are reported.

**Results:** Polyphenol protective properties have been initially ascribed to their antioxidant activity, free radical scavenging, and metal chelating properties. Polyphenols exert their antioxidant activity at the gastro-intestinal (GI) level, through their ability to quench free radicals formed in the GI tract and to chelate ions such as iron. Epidemiological and observational studies support the positive effects of polyphenols on many gastrointestinal diseases. For instance, the consumption of high amounts of green tea has been associated with the prevention of chronic gastritis and GI cancer [3]. Moreover, in vitro and in vivo studies have shown that some flavonoids inhibit the release of pro-inflammatory cytokines in human gastric adenocarcinoma cells infected with *Helicobacter pylori* [4] and significantly decrease lipid peroxidation and reactive oxygen species in gastric mucosa submitted to ketoprofen-induced oxidative damage [5].

As far as systemic effects of polyphenols are concerned, their poor bioavailability, mainly due to their low solubility in physiological conditions and extensive metabolism, has prompted recent consideration for other potential mechanisms of action, which may explain the protective effects of these dietary components at systemic levels. The main direct or indirect molecular targets playing important roles in the health maintaining properties of polyphenols include the expression of genes involved in key steps of oxidative stress and pro-inflammatory response, suggesting that nutrigenomic effects could participate in mediating the protective activities of polyphenols. Moreover, growing evidence suggests that polyphenols are able to modulate the expression of microRNA (miRNA) [6]. These are small non-coding RNAs containing about 22 nucleotides, which control various biological processes, such as cell development, differentiation, proliferation and apoptosis, through their capacity to bind to target mRNAs, especially in the 3′UTR region, and cause a negative regulation of gene expression by inhibiting target mRNA translation or inducing its degradation [7]. The deregulation of miRNA levels could lead to the development of oxidative stress and inflammation based-pathologies. In turn, the modulation of the expression levels of miRNAs involved in oxidative stress and inflammation, at least in part, could explain the protective antioxidant and anti-inflammatory activity of polyphenols [8].

**Conclusions:** The mechanisms of action at the basis of polyphenol protective activity seem to be different depending on the site of action. In fact, it may be assumed that they act through a direct antioxidant activity in the gastrointestinal system and through an indirect antioxidant mechanism at sistemic level, where, at least in part, polyphenols exert their protective properties via the interaction with cell signaling pathways as gene regulators.

**References**

1. Scalbert A, Johnson IT, Saltmarsh M. Polyphenols:antioxidants and beyond. Am J Clin Nutr. 2005; 81:215S–217S.
2. Perez-Jimenez J, Fezeu L, Touvier M, Arnault N, Manach C, Hercberg S, Galan P, Scalbert A. Dietary intake of 337 polyphenols in French adults. Am J Clin Nutr. 2011; 93:1220–1228.
3. Asfar S, Abdeen S, Dashti H, Asfar S. Green Tea Induced Cellular Proliferation and the Expression of Transforming Growth Factor-β1 in the Jejunal Mucosa of Fasting Rats. *Nutrition*, 2003. 19:536–40.
4. Skiba MA, Szendzielorz K, Mazur B, Król W. The inhibitory effect of flavonoids on interleukin-8 release by human gastric adenocarcinoma (AGS) cells infected with cag PAI (+) Helicobacter pylori. Cent Eur J Immunol. 2016; 41:229–35.
5. Cheng YT, Wu CH, Ho CY, Yen GC. Catechin protects against ketoprofen-induced oxidative damage of the gastric mucosa by up-regulating Nrf2 in vitro and in vivo. J Nutr Biochem. 2013; 24:475–83.
6. Blade C, Baselga-Escudero L, Arola-Arnal A. microRNAs as new targets of dietary polyphenols. Curr Pharm Biotechnol. 2014; 15:343–51.
7. Bartel DP. MicroRNAs: genomics, biogenesis, mechanism, and function. Cell. 2004; 116:281–97.
8. Tili E, Michaille JJ. Promiscuous effects of some phenolic natural products on inflammation at least in part arise from their ability to modulate the expression of global regulators, namely microRNAs. Molecules. 2016; 21:E1263.

## 25 Molecular regulation of tanshinone biosynthesis in *Salvia miltiorhiza*

### Shi Min^1,2^, Yao Wang^2^, Wei Zhou^2^,Guoyin Kai^2^

#### ^1^State Key Laboratory of Bioreactor Engineering, East China University of Science and Technology, Shanghai 200237, China; ^2^Institute of Plant Biotechnology, College of Life and Environment Sciences, Shanghai Normal University, Shanghai 200234, China

##### **Correspondence:** Guoyin Kai - gykai@hotmail.com

*Journal of Chinese Medicine* 2018, **13(Suppl 1):**25

**Background:** *Salvia miltiorrhiza* Bunge (also named danshen), is a famous traditional herb medicine widely used for the clinical treatment of cardiovascular and cerebrovascular diseases [1–2]. As one group of active constituents in *S. miltiorrhiza*, the abietane-type diterpenes tanshinones have been reported to own a variety of biological activities such as antitumor, anti-ischemic and antioxidant properties. However, the low contents of tanshinones in *S. miltiorrhiza* plants cannot meet the increasing clinical demand. In recent years, the genes involved in tanshinone biosynthetic pathway have been successfully isolated and characterized from *S. miltiorrhiza,* such as *SmGGPPS* (geranylgeranyl diphosphate synthase), *SmHMGR* (3-hydroxy-3-methylglutaryl CoA reductase), *SmDXR* (1-deoxy-D-xylulose-5-phosphate reductoisomerase), *SmDXS* (1-deoxy-D-xylulose-5-phosphate synthase).

**Results:** Pathway engineering has been an effective strategy to elevate the production of bioactive secondary metabolites [3]. Genetic engineering method was employed to enhance production of tanshinones significantly in transgenic hairy root lines than the control [1–5]. Co-expressing of *SmHMGR* and *SmGGPPS* can increase tanshinone production significantly in a transgenic *S. miltiorrhiza* hairy root line HG9 [3]. Also, when *SmHMGR* and *SmDXR* were co-expressed into *S. miltiorrhiza* hairy roots, the content of tanshinone was clearly enhanced, especially after elicitation by yeast extract and/or Ag+ [4]. Simultaneous introduction of *SmGGPPS* and *SmDXSII* can improve the production of tanshinones to 12.93 mg/g dry weight in line GDII10, and all the single-gene transformed lines showed higher content of tanshinones compared with the control. To elucidate the effect of *SmGGPPS* and *SmDXSII* on isoprenoids in plants, they were co-expressed in *Arabidopsis thaliana* plants. Transgenic lines harboring *GGPPS* and *DXSII* showed raised levels of chlorophyll, carotenoids, gibberellins, and indole acetic acid when compared with the wild lines [5].

**Conclusions:** The above results indicated that it is a promising method to improve the production of tanshinones and other natural bioactive compounds by metabolic engineering strategy.

**References**

1. Shi M, Zhou W, Zhang JL, Huang SX, Wang HZ, Kai GY. Methyl jasmonate induction of tanshinone biosynthesis in *Salvia miltiorrhiza* hairy roots is mediated by JASMONATE ZIM-DOMAIN repressor proteins. Sci Rep. 2016; 6:20919
2. Zhou W, Huang FF, Li S, Wang Y, Zhou CC, Shi M, Wang J, Chen YJ, Wang Y, Wang HZ, Kai GY. Molecular cloning and characterization of two 1-deoxy-Dxylulose-5-phosphate synthase genes involved in tanshinone biosynthesis in *Salvia miltiorrhiza*. Mol Breed. 2016; 36:124
3. Kai GY, Xu H, Zhou CC, Liao P, Xiao JB, Luo XQ, You LJ, Zhang L. Metabolic engineering tanshinone biosynthetic pathway in *Salvia miltiorrhiza* hairy root cultures. Metab Eng. 2011; 13:319–27
4. Shi M, Luo XQ, Ju GH, Yu XH, Hao XL, Huang Q, Xiao JB, Cui LJ, Kai GY. Increased accumulation of the cardiocerebrovascular disease treatment drug tanshinone in *Salvia miltiorrhiza* hairy roots by the enzymes 3-hydroxy-3-methylglutaryl CoA reductase and 1-deoxy-D-xylulose 5-phosphate reductoisomerase. Funct Integr Genomics. 2014; 14:603–15
5. Shi M, Luo XQ, Ju GH, Li LL, Huang SX, Zhang T, Wang HZ, Kai GY. Enhanced diterpene tanshinone accumulation and bioactivity of transgenic *Salvia miltiorrhiza* hairy roots by pathway engineering. J Agric Food Chem. 2016; 64:2523–30

## 26 Reducing the antinutritional factor phytic acid in staple food crops

### Søren K. Rasmussen, Weiyao Fan, Anna Maria Torp

#### Molecular Plant Breeding, Plant and Soil Science Section, Department of Plant and Environmental Sciences, University of Copenhagen, Copenhagen, Denmark

##### **Correspondence:** Søren K. Rasmussen

*Journal of Chinese Medicine* 2018, **13(Suppl 1):**26

**Background:** Phytic acid (*myo*-inositol 1,2,3,4,5,6 hexakisphosphate; PhA) can take the form of phytate and is the major phosphorus (P) storage molecule present in plant seeds [1]. PhA has several negative consequences in human and animal health as well as in agriculture. It is not digestible to humans and non-ruminants because of the absence of the degrading enzyme phytase in the gastro-intestinal tract. PhA also chelates to minerals such as zinc and iron, rendering them bio-unavailable to monogastrics like pigs and humans resulting in major mineral deficiencies in especially the populations in developing countries as their diets mainly consist of grains without access to phytase enzyme from other food sources. A better understanding of crop plant PhA biosynthetic pathway and the genetic improvement of varieties with reduced phytate content are important objectives in plant breeding. The identification of the precise mechanism, enzymes and regulatory genes involved in this pathway is needed to adequately manipulate the PhA amount in the seed.

**Case report:** There is whole array of technologies and methods that can reduce the antinutritional factor: household food preparation, production of complementary food [3], exploring the grain phytase, GMO strategies in plants as overexpression of phytase in planta, blocking the biosynthetic partway by mutations in planta by employing new breeding technologies like crispr/cas9 or simply exploring existing variation and biodiversity.

**Conclusion:** Although individual enzymes have been characterized in several plants and LPA mutants have been identified, the genetic variation, gene expression and function, and a definitive biosynthetic pathway for PhA in crop plants remains largely unknown. Alternative to removing the PhA crops can be biofortified by foliar application of zinc in the field [2].

**References**

1. Raboy V, Johnson A, Bilyeu K, et al. J Am Oil Chem Soc. 2017; 94:353–62
2. White PJ, Thompson JA, Wright G, et al. Plant and Soil. 2017; 411:151–65
3. Roos N, Sørensen JC, Sørensen H, et al. Maternal and Child Nutrition. 2013; 9:47–71.

## 27 What are essential to elucidate action mechanism of multi-ingredient natural drugs?

### Haruki Yamada^1,2^

#### ^1^School of Pharmacy, Tokyo University of Pharmacy and Life Sciences 1432-1, Horiniuchi, Hachioji-shi, Tokyo 192-0392, Japan; ^2^Kitasato Institute for Life Sciences, Kitasato University, 5-9-1, Shirokane, Minato-ku, Tokyo 108-8641, Japan

##### **Correspondence:** Haruki Yamada

*Journal of Chinese Medicine* 2018, **13(Suppl 1):**27

**Background:** With the progress of modern science and technology, modern medicine has greatly improved. However, disease structure has turned to see a marked increase in non-specific, constitutional or psychosomatic and multifactorial diseases under increasing populations of aged persons and changing life style and circumstances etc. Natural products are very important resources for the drug discovery and it was proved that 2015 Nobel Prize in Physiology or Medicine was awarded to the discovery of useful anti-parasitic drugs derived from the natural products, and their great contribution for global health. As another drugs derived from natural resource, traditional herbal medicines such as traditional Chinese medicines and Kampo (Japanese traditional) medicines are suitable for the treatment of multifactorial diseases because these medicines have been used as the traditional herbal formula, in which these multi-ingredients drugs due to component herbs are relatively effective to recover complicated symptoms caused by disturbance of the body systems such as immune, neural and endocrine systems. In Japan, 148 standardized Kampo pharmaceutical preparations have been developed as mostly granule type, and covered by national health insurance system. Now, more than 80% practicing physicians have experience of using the Kampo medicines for the treatment depending on the patient’s clinical situation, either separately or to complement modern western medicine in modern-day therapy of Japan. Present paper introduces our pharmacological studies of Kampo medicines to discuss what are essential to elucidate action mechanism of multi-ingredient natural drugs.

**Results:** Each Kampo formula seems to be the symptoms and constitution-dependent compounds library containing the set of essential low-molecular weight ingredients and bioactive polysaccharides, and these multi-ingredients may attack to multiple target sites to show multilateral actions. Shoseiryuto (SST) showed to recover bronchial airway inflammation but also airway infection in the animal model. SST had the oral adjuvant activity through the synergic effect of two stereo-isomers in the constituents. Hochuekkito showed to act activation of upper respiratory mucosal immune system and to repair epithelial tissue injury in local mucosal sites through the control of intestinal mucosal immune systems by the overall combination effects of the low-molecular weight ingredients and bioactive polysaccharides. Kososan showed an anti-depressive-like effect in the model mice via the normalization of hypotharamic-pituitary-adrenal axis, ameliorating effect of the stress-induced decreases in the number of neural progenitor cells, and increase of orexin-A-positive cells in lateral hypothalamic area.

**Conclusion:** Accumulated comprehensive results may contribute for more evidence-based clinical applications and further drug discovery.

## 28 Synergic effect of glucan particles from *Saccharomyces serevisiae* and curcumin on lipopolysaccharide-induced inflammatory response in vitro

### Jan Hošek^1^, Zuzana Plavcová^1^, Ivan Saloň^2^, Petra Šalamúnová^2^, František Štěpánek^2^, Jaroslav Hanuš^2^

#### ^1^Department of Molecular Biology and Pharmaceutical Biotechnology, Faculty of Pharmacy, University of Veterinary and Pharmaceutical Sciences Brno, Palackého 1946/1, CZ-612 42 Brno, Czech Republic; ^2^Department of Chemical Engineering, University of Chemistry and Technology Prague, Technicka 3, 166 28 Prague 6, Czech Republic

##### **Correspondence:** Jan Hošek - hosekj@vfu.cz

*Journal of Chinese Medicine* 2018, **13(Suppl 1):**28

**Background:** Glucan particles (GPs) from *Saccharomyces cerevisiae* are hollow cell walls that are composed mainly of β-1,3-d-glucans. β-Glucans have demonstrated immunomodulatory and anti-inflammatory potential both in vitro and in vivo. Several studies confirmed the ability of β-glucans to attenuate the lipopolysaccharide (LPS)-induced inflammatory response in vitro [1]. Curcumin is natural hydrophobic phenolic compound, which possesses significant anti-inflammatory effect [2]. The aim of this study is to elucidate whether it is possible to reach synergic effect or other benefit by the co-application of GPs and curcumin.

**Materials and methods:** The GPs were prepared by the partial extraction of yeast cell components as described in our recent work [3]. As a model for determination of anti-inflammatory potential, the THP1-XBlue™-MD2-CD14 cell line (Invivogen, USA) was used. It expresses an NF-κB/AP-1-inducible SEAP (secreted embryonic alkaline phosphatase) reporter gene. Cells were pre-treated by GPs, curcumin, or their mixture in different concentrations and ratios. After 1 h, the NF-κB/AP-1 signalling pathway was triggered by the addition of LPS. The NF-κB/AP-1 activity was detected 24 h later.

**Results:** GPs and curcumin alone did not induce NF-κB/AP-1 activity. When only GPs were used before LPS addition, they significantly (*p* < 0.05) attenuated the effect of LPS. Interestingly, a simple dose dependence has not been observed. Application of curcumin together with GPs strengthened the anti-inflammatory potential of pure GPs and reduced the NF-κB/AP-1 activity in dose-depend manner.

**Conclusions:** Obtained results indicated the strong potential of co-application of GPs from *Saccharomyces cerevisiae* with curcumin as promising therapeutic strategy for treatment of inflammatory diseases.

**Acknowledgements:** This work was supported by Ministry of Health of the Czech Republic, grant nr. 16-27522A. All rights reserved.

**References**

1. Du B, Lin C, Bian Z, Xu B. An insight into anti-inflammatory effects of fungal β-glucan. Trends Food Sci Technol. 2015; 41:49–59.
2. Pulido-Moran M, Moreno-Fernandez J, Ramirez-Tortosa C, Ramirez-Tortosa M. Curcumin and Health. Molecules. 2016; 21:264.
3. Saloň I, Hanuš J, Ulbrich P, Štěpánek F. Suspension stability and diffusion properties of yeast glucan microparticles. Food Bioprod Process. 2016; 99:128–35.

## 29 Chemical composition and antifungal activity of *Michelia alba* oil and linalool in vapor phase against *Aspergillus flavus*

### Sumethee Songsamoe^1^, Narumol Matan^1^, Nirundorn Matan^2^

#### ^1^Food Technology, School of Agricultural Technology, Walailak University, Nakhon Si Thammarat, Thailand; ^2^Walailak University, Nakhon Si Thammarat, Thailand

##### **Correspondence:** Narumol Matan - nnarumol@wu.ac.th

*Journal of Chinese Medicine* 2018, **13(Suppl 1):**29

**Background:** *Aspergillus flavus* is one of the most important of stored agricultural products. *A. flavus* is commonly known as a toxic moulds which pose a risk to human health [1] and can be caused postharvest losses and diseases in grains and grains products [2]. Consequently, safe method to control the *A. flavus* on agricultural products was needed to find. The natural compounds treatment especially the essential oils have been recognized as a harmless agent for food preservation [3]. Therefore, the objective of this study was to investigate the effect of *Michelia alba* oil and its main component (linalool) in the vapor phase on the growth of *A. flavus.*

**Materials and methods:** The components of the *M. alba* oil were identified using GC–MS analysis. Different volumes (150, 300, 450 μl) of *M. alba* oil and linalool were first absorbed by plant absorbent material (Φ ~ 20 mm) before being added into a closed airtight plastic box (1L). Next, Malt Extract Agar (MEA) containing *A. flavus* on a Petri dish (uncovered plate) was placed into the box and then, kept at 25 °C for 10 days. During the incubation, the diameter of the mold colony in the center was measured every day.

**Results:** The effect of vapor phase of *M. alba* oil and linalool are shown in Fig. 1. Results indicated that the vapor phase of *M. alba* oil at ≥ 300 μl L^−1^ air and linalool at ≥ 150 μl L^−1^ air could inhibit the growth of *A. flavus* on MEA by 100% for at least 10 days. Therefore, linalool vapor showed good inhibitor to inhibit growth of *A. flavus* than using *M. alba* vapor alone. Result from the GC–MS found that two main components of the *M. alba* leaf were L-linalool (73.74%) and *trans*-Caryophyllene (7.35%), respectively.

**Fig. 1:**
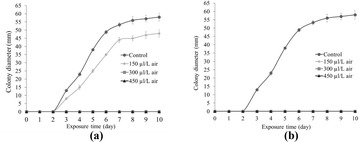
Effect of vapor phase of *M. alba* oil (**a**) and linalool (**b**) at 150, 300, and 450 µl L^−1^ air on the growth of *A. flavus*

**Conclusions:** The vapor phase of *M. alba* oil at ≥ 300 μl L^−1^ air and linalool at ≥ 150 μl L^−1^ air could completely inhibit the growth of *A. flavus* on MEA for at least 10 days. And Linalool is one of the main components of *M. alba* oil that showed a strongest antifungal activity in the vapor phase of this essential oil. Therefore, *M. alba* vapor demonstrated a good potential to control significant growth of *A. flavus*. This explore might be useful for grain storage industry.

**References**

1. Makun HA, Gbodi TA, Akanya OH, Salako, EA, Ogbadu, GH. Fungi and some mycotoxins contaminating rice (Oryza sativa) in Niger state, Nigeria. Afr J Biotechnol. 2007; 6.
2. Suhem K, Matan N, Nisoa M, Matan, N. Inhibition of *Aspergillus flavus* on agar media and brown rice cereal bars using cold atmospheric plasma treatment. Int J Food Microbiol. 2013; 161:107–11.
3. Burt S. Essential oils: their antibacterial properties and potential applications in foods-a review. Int J Food Microbiol. 2004; 94:223–53.

## 30 Secondary metabolites in different *Linaria* species

### T. V. Matveeva, S. V. Sokornova, D. A. Domoratskaya, O. D. Bogomaz

#### St. Petersburg State University, St. Petersburg, Russia

##### **Correspondence:** T. V. Matveeva

*Journal of Chinese Medicine* 2018, **13(Suppl 1):**30

**Background:** Naturally transgenic plants represent species, containing sequences, homologous to *Agrobacterium* T-DNA, in their genomes. Their ancestors were transformed by *Agrobacterium* and gave a rise to new species [1]. They are described in genera *Nicotiana, Linaria, Ipomoea.* All these plants are used in folk medicine, since they contain big amounts of secondary metabolites [2–3]. *Linaria* species are used as an anti-inflammatory and laxative. More than 200 secondary metabolites have been identified in 41 *Linaria* species so far [4]. They include iridoids [5], flavonoids [6], organic acids, saponins [7], diterpenoids and steroids [8]. The major fraction of them is iridoid glycosides. Although the spectrum of secondary metabolites is described in many *Linaria* species, nobody compared species in the aligned conditions. Here we identify and compare levels of iridoid glycosides in *Linaria* species, representing three sections and develop procedures to obtain plant tissue cultures, producing these compounds.

**Materials and methods:** Seeds of *L. vulgaris, L. genistifolia, L. cretaceae, L. maroccana* were sterilized with H_2_O_2_ and germinated on Murashige and Scoog [9] medium (MS). Then plants were propagated by cuttings. Shoots and roots were used for iridoid glycosides analysis. Internode explants were used for transformation with pRiA4 of *A. rhizogenes* and callus formation on MS media with 6-benzylaminopurine and α-naphthaleneacetic acid in different concentrations. Fresh aerial parts of plants *Linaria* were extracted with MeOH for 1 h in an ultra-sonic bath. The extract was filtered through a Minisart filter type RC-0.45 µm and analysed directly by HPLC on a Shimadzu SCL-6A System controller with a flow of 1 ml/min and the column C18.The system was an isocratic 3% acetonitrile in water [10]. The TLC solvent system used was ethyl acetate–methanol-water (38.5: 7.5: 4). Detection was done with UV light at 254 nm.

**Results:** Antirride and antirrhinoside were detected in shoots and roots of all investigated species. The amount of these compounds was higher in shoots, than in roots. Naturally transgenic *L. vulgaris, L. genistifolia, L. cretaceae* contain higher amounts of antirride and antirrhinoside, than non-transgenic *L. maroccana*. Transformation of *L. maroccana* with pRiA4 leads to the formation of hairy roots on the internode explants. Hairy roots are characterised by increasing of levels of the iridoids. All studied species effectively form calli, producing iridoid glycosides.

**Conclusion:** Investigated naturally transgenic species of Linaria are characterized by high levels of iridoid glycosides. This can be used in pharmacology.

This work was supported by RSF grant 16-16-10010.

**References**

1. Matveeva T, Lutova L. Horizontal gene transfer from *Agrobacterium* to plants. Front Plant Sci 2014; 5:326
2. Matveeva T, Sokornova S, Lutova L. Influence of *Agrobacterium* oncogenes on secondary metabolism of plants. Phytochem Rev 2015; 14:541.
3. Matveeva T, Sokornova S. *Agrobacterium rhizogenes*-mediated transformation of plants for improvement of yields of secondary metabolites. In: Pavlov A, Bley T, editors. Reference Series in Phytochemistry. Bioprocessing of plant in vitro systems. Springer; 2016:1–42
4. Venditti A, Serrilli A, Di Cecco M. Phytochemical analysis of *Plantago sempervirens* from Majella National Park. Nat Prod Res. 2012; 26:2035–9.
5. Boros C, Stermitz F. An updated review. Part II. J Nat Prod. 1990; 53:1055–147.
6. Harborne J, Valdés B. Identification of scutellarein 40-methyl ether in Linaria aeruginea. *Phytochem Rev*. 1971; 10:2850–1.
7. Ercil D, Sakar M. Chemical constituents of *Linaria aucheri* Linaridial, a new cis-clerodane-type diterpene dialdehyde, from *Linaria japonica* Miq. Turk J Chem. 2004; 28:133–9.
8. Kitagawa I, Yoshihara M, Kamigauchi T. Linarienone, a newcis-clerodane-type diteppene from the subterranean part of Linaria japonica Miq *Tetrahedron Lett.* 1977; 14:1221–4.
9. Murashige T, Skoog F. A revised medium for rapid growth and bioassay with tobacco tissue culture. Physiologia Plantarum. 1962; 15:473–97.
10. Drøhse Høgedal B., Mølgaard P. HPLC analysis of the seasonal and diurnal variation of iridoids in cultivars of *Antirrhinum majus*. Biochem Syst Ecol. 2000; 28:949–62

## 31 Metabolic engineering of the MVA pathway for isoprenoid production in plants

### Pan Liao^1,2^, Hui Wang^1^, Xinjian Chen^1^, Mingfu Wang^1^, Thomas J. Bach^3^, Mee-Len Chye^1,2^

#### ^1^School of Biological Sciences, The University of Hong Kong, Pokfulam Road, Hong Kong, China; ^2^Parner State Key Laboratory of Agrobiotechnology (CUHK); ^3^Centre National de la Recherche Scientifique, UPR 2357, Institut de Biologie Moléculaire des Plantes, 67083 Strasbourg, France

##### **Correspondence:** Pan Liao - mlchye@hku.hk

*Journal of Chinese Medicine* 2018, **13(Suppl 1):**31

**Background:** Isoprenoids consist of a large group of functional natural products. The second enzyme in the mevalonate (MVA) pathway, 3-hydroxy-3-methylglutaryl-coenzyme A synthase (HMGS), produces isoprenoids including phytosterols [1,2]. Dietary phytosterols are important because they are known to lower blood cholesterol levels. Previous studies on the enzyme kinetics of recombinant wild-type (wt) and mutant *Brassica juncea* HMGS1 have shown that the HMGS mutant enzyme S359A had a tenfold higher activity [3]. The overexpression of *B. juncea* wt and mutant (S359A) HMGS1 in *Arabidopsis* upregulated native *HMGR*, *SMT2*, *DWF1*, *CYP710A1* and *BR60X2*, thereby increasing sterol content [4]. Furthermore, tobacco HMGS overexpressors (OEs) enhanced native *NtHMGR1*, *NtIPI2*, *NtSQS*, *NtSMT1*-*2*, *NtSMT2*-*1*, *NtSMT2*-*2* and *NtCYP85A1* expression in seedlings, improving sterol content, plant growth and seed yield [5]. Enhanced growth and seed yield in tobacco OE-S359A in comparison to OE-wtBjHMGS1 corresponded to higher *NtSQS* expression and sterol content in OE-S359A [5].

**Results and conclusions:** In the present study, wt and mutant (S359A) BjHMGS1 were overexpressed in a crop plant, tomato (*Solanum lycopersicum*). The results obtained from this study will be presented and the feasibility in manipulating isoprenoid production using wt and mutant HMGS in an edible fruit will be discussed.

This work was supported by the Wilson and Amelia Wong Endowment Fund, Research Grants Council of Hong Kong (AoE/M-05/12), Innovation Technology Fund of the Innovation Technology Commission (Support to Partner State Key Laboratories in Hong Kong) and HKU CRCG awards (0910159039, 1007160002, 1511159010). PL was supported by a University Postgraduate Fellowship and a Postdoctoral Fellowship.

**References**

1. Liao P, Hemmerlin A, Bach TJ, Chye ML. The potential of the mevalonate pathway for enhanced isoprenoid production. Biotechnol Adv. 2016; 34:697–713.
2. Liao P, Wang H, Hemmerlin A, Nagegowda DA, Bach TJ, Wang M, Chye ML. Past achievements, current status and future perspectives of studies on 3-hydroxy-3-methylglutaryl-CoA synthase (HMGS) in the mevalonate (MVA) pathway. Plant Cell Rep., 2014; 33: 1005-1022.
3. Nagegowda DA, Bach TJ, Chye ML. *Brassica juncea* HMG-CoA synthase 1: expression and characterization of recombinant wild-type and mutant enzymes. Biochem. J., 2004; 383:517–27.
4. Wang H, Nagegowda DA, Rawat R, Bouvier-Navé P, Guo D, Bach TJ, Chye ML. Overexpression of *Brassica juncea* wild-type and mutant HMG-CoA synthase 1 in Arabidopsis up-regulates genes in sterol biosynthesis and enhances sterol production and stress tolerance. Plant Biotechnol J. 2012; 10:31–42.
5. Liao P, Wang H, Wang M, Hsiao AS, Bach TJ, Chye ML. Transgenic tobacco overexpressing *Brassica juncea* HMG-CoA synthase 1 shows increased plant growth, pod size and seed yield. PLoS ONE, 2014; 9:e98264.

## 32 Harnessing Lactic acid bacteria for riboflavin production

### Kiran Thakur

#### School of Food Science and Engineering, Hefei University of Technology, Hefei 230009, People’s Republic of China

##### **Correspondence:** Kiran Thakur - kumarikiran@hfut.edu.cn

*Journal of Chinese Medicine* 2018, **13(Suppl 1):**32

**Background:** Riboflavin been traditionally synthesized for food and feed fortification by chemicals means but past decade has witnessed a surge in information about commercial biotechnological processes.

**Materials and methods:** In this study, among the 55 isolates bioprospected from dairy and non-dairy sources, 16 isolates were found harbouring complete *rib* structural genes. The isolates harbouring both complete as well as incomplete operon were compared phenotypically for riboflavin production by chemical, fluorescence, microbiological based assays and HPLC. Among the screened isolates on agar diffusion assay, the maximum increase in growth of auxotroph was observed in the presence of KTLF1 and KTLF16 respectively. Expression pattern of *rib* genes was studied in selected isolates and MTCC8711. RNA was isolated at different intervals of time in MRS and riboflavin assay medium (RAM), milk and whey based medium. On the basis of fold increase in relative mRNA expression of all the *rib* genes, the isolate KTLF1 was selected for expression studies in milk and whey. The riboflavin producers were further screened for in vitro probiotic, safety aspects as well as technological properties.

**Results:** Three riboflavin producing isolates were able to show potential probiotic, safety attributes as well as appreciable adhesion on HT-29 cell lines as well as hold the promises to be used as novel starter cultures.

**Conclusion:** The study has generated the data for further exploration of these isolates endowed with appreciable starter as well as functional activities for industrial use as novel and native starter cultures to produce an essential vitamin in situ which would contribute significantly to the functional value of certain fermented foods.

**Keywords**: Riboflavin, Health, Probiotic and Milk

## 33 Bioactive metabolites from the jellyfish-derived fungus *Penicillium chrysogenum* J08NF-4

### Sen Liu^1^, Mingzhi Su^1^, Eun La Kim^1^, Tae Hwan Noh^1^, Zhiran Ju^1^, Haoran Feng^1^, Jongki Hong^2^, Jee H. Jung^1^

#### ^1^College of Pharmacy, Pusan National University, Busan, Republic of Korea, 609-735; ^2^College of Pharmacy, Kyung Hee University, Seoul, Republic of Korea, 130-701

##### **Correspondence:** Jee H. Jung - jhjung@pusan.ac.kr

*Journal of Chinese Medicine* 2018, **13(Suppl 1):**33

**Background:** Marine microorganisms are widely recognized to represent a new frontier for the discovery of pharmaceutical agents. In particular, endozoic fungi from diverse marine environments offer prolific sources of structurally unique and biologically active metabolites due to the complexities of fungal biosynthetic genes.

**Materials and methods:** The antibacterial activities against human pathogens, marine pathogens and multi-drug resistant strains were analysed using disc diffusion method. The planar structures, as well as the relative configurations of the compounds were elucidated on the basis of spectral analyses, including 1D, 2D NMR and MS experiments.

**Results:** Bioassay-guided fractionation of the fermentation broth of jellyfish-derived fungus *Penicillium chrysogenum* J08NF-4 resulted in the isolation of three bioactive meroterpenes, farnesylbenezenediol (**1**) [1], 7-deacetoxyyanuthone A (**2**) [2], farnesylquinone (**3**) [3]. Compounds **1-3** showed moderate activities against *Staphyococcus aureus* SG511*, Staphyococcus aureus* SG503, *Streptococcus iniae* FP3187, MDR *Pseudomonas aeruginosa* 2200 and MRSA 3089. Compound **1** also exhibited moderate cytotoxicities against KB, HepG2, and HCT 116 cells with IC_50_ values of 4.73, 20.02, and 18.10 μM, respectively.

**Conclusions:** This jellyfish-derived fungus strain was found as an excellent producer of biologically active meroterpenes. Further biological mechanisms of the meroterpens are underway.

**References**

1. Son B, Kim J, Choi H, Kang J. A radical scavenging Farnesylhydroquinone from a marine-derived fungus*Penicillium* sp. Arch Pharm Res. 2002; 25:77–9.
2. Li X, Choi H, Kang J, Lee C, Son B. New Polyoxygenated Farnesylcyclohexenones, Deacetoxyyanuthone A and Its Hydro Derivative from the Marine-Derived Fungus *Penicillium* sp. J Nat Prod. 2003; 66:1499–500.
3. Liu D, Yang A, Wu C, Wu C, Guo P, Proksch P, Lin W. Lipid-lowering effects of farnesylquinone and related analogues from the marine-derived *Streptomyces nitrosporeus.* Bioorg Med Chem Lett. 2014; 24:5288–93.

## 34 Platycodin D, a natural product isolated from *Platycodonis radix*, displays potential anticancer effects as single or combination therapy

### Ting Li, Zhenghai Tang, Xin Chen, Xiaohuang Xu, Jinjian Lu

#### State Key Laboratory of Quality Research in Chinese Medicine, Institute of Chinese Medical Sciences, University of Macau, Macao, China

##### **Correspondence:** Jinjian Lu - jinjianlu@umac.mo

*Journal of Chinese Medicine* 2018, **13(Suppl 1):**34

**Background:** Platycodin D (PD) is a triterpenoid saponin isolated from the Chinese herb Platycodonis Radix. It possesses various biological activities, such as immune stimulation, anti-nociception, anti-obesity and anti-cancer, etc.

**Materials and methods:** MTT, colony formation assay, flow cytometry, western blotting, transwell assay, immunoprecipitation, siRNA, real-time PCR and xenograft models were adopted to study the anticancer potential and mechanisms of PD.

**Results:** PD exhibited potential effects on the anti-adhesion, antimigration, anti-invasion as well as antiproliferation activities in cancer cells [1]. PD induced apoptosis and enhanced the anticancer properties of the chemotherapy drug doxorubicin via promotion of apoptotic activity [1,2]. In addition, PD triggered protective autophagy via ERK-JNK pathways in cancer cells [3,4]. We further demonstrated PD as a novel Hsp90 inhibitor by disrupting the protein–protein interaction of Hsp90 and its chaperone Cdc37 (unpublished data). This disruption by PD resulted in degradation of Hsp90 client proteins without feedback increase of Hsp70 (unpublished data). AKT and mTOR inhibitors were found to increase the expression of EGFR, HER-2 and IGF1R upon AKT/mTOR inhibition, while PD reduced the total levels of EGFR, HER-2 and IGF1R expression. Co-treatment of the AKT inhibitor or mTOR inhibitor with PD blocked this feedback activation and triggered enhanced antiproliferation activity and apoptotic effect ^[5]^.

**Conclusions:** These findings not only identified PD is a potential agent for cancer treatment, but also establish a novel mechanistic rationale for the combination approach with PD.

**Acknowledgments:** This study was funded by the grants from Science and Technology Development Fund, Macao S.A.R (FDCT) (024/2016/A1, 070/2013/A and 074/2012/A3), Research Fund of University of Macau (MYRG2015-00091-ICMS-QRCM and MYRG2015-00101-ICMS- QRCM).

**References**

1. Li T, Xu WS, Wu GS, et al. Asian Pac J Cancer P. 2014; 15:1745–9.
2. Tang ZH, Li T, Gao HW, et al. Lu JJ. Chin Med-Uk; 2014. p. 9.
3. Li T, Tang ZH, Xu WS, et al. Eur J Pharmacol. 2015; 749:81–8.
4. Li T, Xu X H, Tang Z H, et al. Acta Pharmacol Sin. 2015; 36:1503–13.
5. Li T, Chen X, Chen X, et al. Sci Rep. 2016; 6:37997.

## 35 Insights into anti-cancers mechanism of 10-gingerol from Tongling white ginger

### Fang Zhang, Jun-Jun Shi, Zhao-Jun Wei

#### School of Food Science and Engineering, Hefei University of Technology, Hefei 230009, People’s Republic of China

##### **Correspondence:** Zhao-Jun Wei - china_zhangfang@163.com

*Journal of Chinese Medicine* 2018, **13(Suppl 1):**35

**Background:** Antibacterial agents can inhibit bacterial contamination in food, which is necessary for human health. Therefore, investigate the effect of antibacterial agents with low toxicity is an important issue. Plant compounds extracted from different sources has gaining considerable interest due to its multiaspect in health benefits. However, in the current decade, plants and animals derived polysaccharide fractions have been studied for various biological properties but less attention is given so far on their antibacterial potential, and their exact antagonistic mechanism is still not well understood and needs further research. In our previous studies, the polysaccharides from peony possessed high anticancer, antioxidant, antiinflammatory activity. Thus, the present work with the aim of evaluating anti-bacterial potential of peony polysaccharides under different kinds of bacterial strains.

**Materials and methods:** For evaluating the antagonistic effect of four kinds of poney polysaccharides under different bacterial species (*Staphylococcus aureus*, *Escherichia coli*, *Bacillus subtilis* and *Salmonella typhimurium*). Antibacterial active ingredients extracted with water by reflux from penoy in different harvest periods, the agar diffusion method was used to measure the antimicrobial ability, inhibition zone diameter was used to ascertain the minimal concentration of polysaccharides to inhibit the growth of test organisms completely. The correlation between polysaccharides active ingredients and antibacterial activity was evaluated. The correlation between characteristic peaks and antibacterial activity was evaluated.

**Results:** After poney polysaccharides administration, regardless of the bacterial strain, DASS represented the strongest antibacterial activity on the basis of largest inhibition zone, the lowest minimal inhibitory concentration and maximum inhibition of bacterial growth in liquid medium.

**Conclusions:** Herein, we investigated the antimicrobial potential of sequentially extracted poney polysaccharide fractions namely HBSS, CHSS, DASS and CASS. Overall results indicated that poney polysaccharides as a kind of natural antimicrobial compounds to restrain the growth of bacteria which hold a promise as potential natural antimicrobial agent to formulate the functional foods with potential applications in the medical and food industries.

## 36 Antioxidant and antiproliferative properties of methanol extract of *Clinacanthus nutans* and identification of chemicals profile by UPLC-QTOF/MS

### Hazrulrizawati Abd Hamid

#### Faculty of Industrial Sciences & Technology, Universiti Malaysia Pahang, Pahang, Malaysia

##### **Correspondence:** Hazrulrizawati Abd Hamid - hazrulrizawati@ump.edu.my

*Journal of Chinese Medicine* 2018, **13(Suppl 1):**36

**Background:** The leaves extract of *C. nutans* have been used extensively as primary sources of complementary and alternative healthcare or as economical in-housing regimens for cancer patients [1]. Patients have claimed that they have recovered from cancer illness after consuming *C. nutans* leaves over a period of time. It has been proved that many antioxidant substances have anticancer or anticarcinogenic properties [2]. A study had pointed to the necessity of chemicals profiling and evaluation of antioxidant and antiproliferative properties of *C. nutans* extract.

**Materials and methods:** The whole plant of *C. nutans* were dried and extracted with methanol. The content of the active components in the extracts was determined by ultrahigh performance liquid chromatography-quadrupole time-of-flight/mass spectrometry (UPLC-QTOF/MS). The compounds were tested for antiproliferative property on HT29, HepG2 and MCF-7 cell lines while antioxidant activity was monitored by radical scavenging assay (DPPH) and ferric reducing power (FRAP).

**Results:** The content of the active components in the extracts were determined by UPLC-QTOF/MS. Six compounds were labelled as confirmed component from methanol extract of *C. nutans*. Peaks with retention times (RT; min) of 4.36, 4.94, 6.63, 7.60, 8.69 and 11.27 were identified as the following: 5,7-dihydroxychromone-7-β-d-glucoside, smiglanin, glabrol, corymboside, viscumneoside II and kushenol U (Fig. 1). Based on the component confirmed plot, the major compound found was corymboside. Methanol extract show significant antioxidant activities in DPPH (IC50 = 127.09 ± 0.042 µg/mL) and total reduction capability (IC50 469.13 ± 0.05 µg/mL) (Table 1) According to the IC_50_ obtained, the methanol extracts showed significant antiproliferative activity on HT29 (IC_50_ 36.19 ± 1.06 mg/mL), HepG2 (IC_50_ 48.37 ± 0.026 mg/mL) and MCF-7(IC_50_ 54.16 ± 0.99 mg/mL) after 72 h of treatment.

**Fig. 1:**
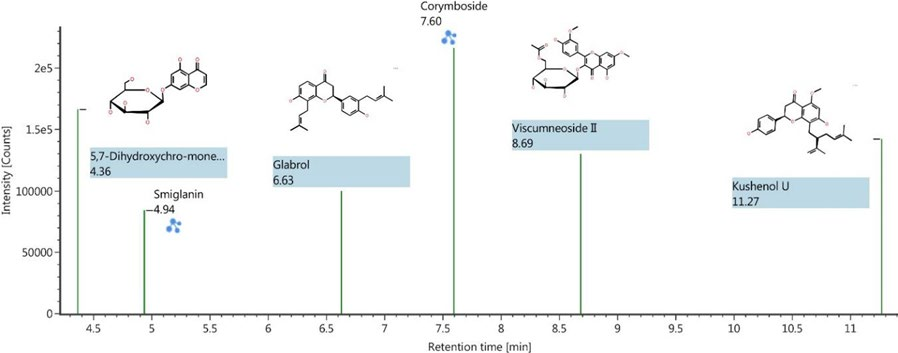
Confirmed component plot of *C. nutans* extract

**Table 1:** IC_*50*_ values of DPPH and FRAP assays of *C. nutans*

Extract	IC_*50*_ values (µg/mL)	
	DPPH	FRAP
Standard	135.02 ± 0.052	405.15 ± 0.16
Methanol	127.09 ± 0.042	469.13 ± 0.05

**Conclusion:** These results suggest antioxidant and antiproliferative properties of *C. nutans* extract might due to the presence of chromones, flavones and their glycosides as evidenced from UPLC-QTOF/MS.

**References**

1. P’ng XW, Akowuah GA, Chin JH. Int J Pharm Sci Res, 2012; 3:4202
2. Bennett LL, Rojas S. J Exp Clinic Med. 2012; 4:215–22

## 37 Structural properties and prebiotic effects of different molecular weight lotus seed resistant starch precipitated by stepwise ethanol

### Mingjing Zheng^1^, Hongliang Zeng^1^, Baodong Zheng^1,2^ and Yi Zhang^1,2^

#### ^1^College of Food Science, Fujian Agriculture and Forestry University, Fuzhou, Fujian, China; ^2^Fujian Provincial Key Laboratory of Quality Science and Processing Technology in Special Starch, Fuzhou, Fujian 350002, China

##### **Correspondence:** Baodong Zheng - zbdfst@163.com; Yi Zhang - zyifst@163.com

*Journal of Chinese Medicine* 2018, **13(Suppl 1):**37

**Background:** Lotus seed resistant starch (GP-LRS3) can escape digestion in the small intestine of healthy individuals and be completely or partially fermented in the colon, which has been reported to increase the proliferation of bifidobacterium. Generally, biopolymer with different molecular weight present a wide range of characteristics, which define its applications and influence the final performance of the products. Therefore, the objective of this study is to investigate the structural properties and prebiotic effects of different molecular weight lotus seed resistant starch precipitated by stepwise ethanol.

**Materials and methods:** In order to obtain LRS3 with different molecular weight,GP-LRS3 was precipitated by stepwise ethanol. Scanning electron microscopy (SEM) and Fourier transform infrared (FT-IR) were adopted to study the structural properties of different molecular weight LRS3. Furthermore, the prebiotic effects of different molecular weight LRS3 were also determined.

**Results:** The results showed two fractions of GP-LRS3 (GP-LRS3-1 and GP-LRS3-2) could be separated by 20 and 30% ethanol, respectively. The weight-average molar mass (*M*_*w*_*)* of GP-LRS3-1 and GP-LRS3-2 were 8.49 × 10^4^ and 2.23 × 10^4^ g/mol, respectively, and the polydispersity index(*M*_*w*_*/M*_*n*_) were 2.28 and 1.56, respectively. SEM micrographs of GP-LRS3-1 and GP-LRS3-2 showed an irregular shape with rough surface, similar to GP-LRS3. FT-IR for GP-LRS3, GP-LRS3-1 and GP-LRS3-2 showed almost identical characteristic bands. Indicating grading alcohol precipitation of LRS3 belong to physical separation without chemical changes. Moreover, there was a significant effect of lotus-seed resistant starch on promoting *bifidobacterium* proliferation and producing shot-chain fatty acids during fermentation in vitro. The effect of promoting *bifidobacterium* proliferation and short-chain fatty acids production were: GP-LRS3 > GP-LRS3-1 > GP-LRS3-2.

**Conclusions:** These results concluded the proliferation of *bifidobacterium* was affected by the molecular weight of lotus seed resistant starch, and the higher the molecular weight of lotus seed resistant starch, the greater the proliferation of *bifidobacterium*.

## 38 Composition profiles, bioavailability and benefit-risk assessment on cardiovascular health of phytosterols in Chinese diet

### Mengmeng Wang^1^, Weisu Huang^2^, Baiyi Lu^1^, Maiquan Li^1^, Liujuan Zhang^1^, Zhou Fei^1^

#### ^1^College of Biosystems Engineering and Food Science, Fuli Institute of Food Science, Zhejiang Key Laboratory for Agro-Food Processing, Zhejiang R&D Center for Food Technology and Equipment, Key Laboratory for Agro-Food Risk Assessment of Minstry of Agriculture, Zhejiang University, Hangzhou, 310058, China; ^2^Zhejiang Economic & Trade Polytechnic, Department of Applied Technology, Hangzhou 310018, China

##### **Correspondence:** Baiyi Lu - bylu@zju.edu.cn

*Journal of Chinese Medicine* 2018, **13(Suppl 1):**38

**Background:** Phytosterols are bioactive substances similar to cholesterol in structure, which could reduce total cholesterol and lower density lipoprotein cholesterol in serum to prevent cardiovascular disease. A daily dietary intake of 2 g phytosterols results in a reduction of low density lipoprotein cholesterol and total plasma cholesterol levels of approximately 10%, but no evidence show intakes of phytosterols higher than 3 g/day have additional cholesterol-lowering benefits and high intakes may induce undesirable effects. Phytosterols can be present in the free form and as esters or glycosides in plant-based foods, and the preparation have been approved as a novel food. The total intake of phytosterols in Chinese people was estimated to be 334.21 mg/day in 2007 which is much lower than the recommendation. However, the Chinese diet structure had changed a lot during the last 10 years and many researches had been done to figure out the physiological function and bioavailability of phytosterols under different processing.

**Conclusions:** This review summarized the content of total and individual phytosterols with free and conjugated forms in different food of the Chinese diet, discussed effect of different process on bioavailability of phytosterols, and evaluated the health benefit and potential adverse effects on cardiovascular health by estimating the total intake of phytosterols from Chinese diet. Finally, we outlined the knowledge gaps on research and presented suggestions on phytosterols intake to Chinese.

## 39 Nuciferine inhibits proinflammatory cytokines via PPARs in LPS-induced RAW264.7 cells

### Chao Zhang^1,2^, Dan Liu^1^, Qingqing Liu^3^, Jianjun Deng^3^, Haixia Yang^1,2^

#### ^1^Nutrition and Food Safety Engineering Research Center of Shaanxi Province, College of Public Health, School of Medicine, Xi’an Jiaotong University, Xi’an, 710061, P. R. China; ^2^Cardiovascular Research Center, Xi’an Jiaotong University, Xi’an, 710061, P. R. China; ^3^Shaanxi Key laboratory of Degradable Biomedical Materials, College of Chemical Engineering, Northwest University, Xi’an, 710069, P. R. China

##### **Correspondence:** Haixia Yang*Journal of Chinese Medicine* 2018, **13(Suppl 1):**39

**Background:** Inflammatory abnormalities are widely implicated in a vast variety of acute and chronic human disease processes. Regulation of inflammatory response relies on multiple potential mechanisms. Nuciferine is an aromatic ring-containing alkaloid found in the nelumbo nucifera leaves, showing potential anti-inflammation activity, but the molecular mechanism of anti-inflammatory effect of nuciferine is still unclear. Thus, in this study, we investigated the anti-inflammatory effect and possible mechanisms of nuciferine in the RAW264.7 cells.

**Materials and methods:** MTT assay was performed to detect nuciferine cytotoxicity. The inflammatory model in vitro was made using RAW264.7 cells stimulated by lipopolysaccharide (LPS). Interleukin 6 (IL-6) and tumor necrosis factor α (TNFα) production was detected using enzyme-linked immunosorbent assay. The real-time polymerase chain reaction was employed to assess the expression of IL-6 and TNFα mRNA. The peroxisome proliferator activated receptors (PPARs) activity was studied by luciferase reporter assay. PPARs specific pharmacological agonists (WY14643/GW501516/rosiglitazone) and antagonists (GW6417/GSK0660/GW9662) were applied for mechanism study. The expression of indicated proteins I-κB was examined by Western blotting.

**Results:** Nuciferine significantly suppressed inflammatory cytokines production such as IL-6 and TNF-α in LPS-induced RAW264.7 cells. In addition, the luciferase assay results of three PPAR subtypes showed that all of the activities are promoted by nuciferine in a dose-dependent manner. Specific inhibitors of PPARα and PPARγ markedly abolished LPS-induced IL-6 and TNF-α production, even I-κB degradation. However, the specific inhibitor of PPARδ did not.

**Conclusions:** Our finding suggested a potential mechanism of the protective effects of nuciferine in LPS-induced inflammation via activating PPARα and PPARγ in RAW 264.7 cells

**Keywords:** nuciferine, peroxisome proliferator-activated receptor, anti-inflammatory, macrophage

**Acknowledgements:** This study was supported by the National Natural Science Foundation of China (21676212 and 21476184).

## 40 Comparative study on composition, physicochemical and antioxidant characteristics of different varieties of kiwifruit seed oil in China

### Qingqing Liu^1^, Chao Zhang^2^, Dan Liu^2^, Haixia Yang^2^, Jianjun Deng^1^

#### ^1^Shaanxi Key laboratory of Degradable Biomedical Materials, College of Chemical Engineering, Northwest University, Xi’an, 710069, P. R. China; ^2^Nutrition and Food Safety Engineering Research Center of Shaanxi Province, College of Public Health, School of Medicine, Xi’an Jiaotong University, Xi’an, 710061, P. R. China

##### **Correspondence:** Jianjun Deng - dengjianjun@nwu.edu.cn

*Journal of Chinese Medicine* 2018, **13(Suppl 1):**40

**Background:** Kiwifruit (Actinidia chinensis Planch.) seed oil (KSO) as a by-product resource in food, pharmaceutical and cosmetics industries, exhibits various bioactivities such as antioxidant, anti-aging and regulating the blood lipids.

**Materials and methods:** Lipids, ash, moisture, protein and polyphenols of the kiwi fruit seeds were detected by the AOAC standard methods. The chemical properties of KSO such as density, refractive index, iodine value, peroxide value, and acidity value were determined by the AOCS methods, and the fatty acid compositions were determined by GC–MS analysis. The antioxidant capacity of KSO was assessed by 1,1-diphenyl-2-picrylhydrazyl (DPPH), oxygen radical absorbance capacity (ORAC), ferric reducing antioxidant power (FRAP) and hydroxyl radical scavenging capacity (HRSC) methods. The single cell gel electrophoresis was performed to evaluate the protective effects of KSO on DNA damage.

**Results:** Kuilv had the most oil content (34.84 ± 0.10 g/100 g) which is followed by Hort 16A (34.73 ± 0.3 g/100 g) and Xuxiang (34.84 ± 0.10 g/100 g). KSOs were rich in unsaturated fatty acids (UFA), accounting for 79.53-85.84% of the total lipid, and the linolenic acid content was the highest in UFA. UFA contents are in the follow order: Yate (85.84/100 g) > Hort 16A (82.65/100 g) > Xuxiang (82.35/100 g). Significantly, all varieties of KSO demonstrated the different levels of antioxidant activities that were evaluated by using in vitro methods. Yate had strong 1,1-diphenyl-2-picrylhydrazyl radical scavenging capacity (35.1 ± 2.06 mg/mL for IC50), whereas Xuxiang had high hydroxyl radical scavenging capacity (1.91 ± 0.01 mg/mL for IC50). Unexpected, Hongyang which had less oil content also have high activities in oxygen radical absorbance capacity (1.99 ± 0.21 mg Trolox) and ferric reducing antioxidant power (107.3 ± 0.3 mg Trolox/kg). The comet assay further verified that the different varieties of KSO obviously protected mice lymphocytes against oxidative DNA damage.

**Conclusion:** Our finding provided an evidence for the bioactivity of KSO as a potential source of dietary supplement and different varities of KSO had their own advantages.

**Acknowledgements:** This study was supported by the National Natural Science Foundation of China (21476184 and 21676212).

**Keywords:** kiwi seed oil, physiochemical properties, antioxidant activities, DNA damage

## 41 The glycosylation of anthocyanins strongly enhance the stabilities under cell culture condition

### Jiaojiao Zhang^1^, Jianbo Xiao^2^, Xiaodong Zheng^1^

#### ^1^Department of Food Science and Nutrition, Zhejiang University, Hangzhou 310058, China; ^2^State Key Laboratory of Quality Research in Chinese Medicine, Institute of Chinese Medical Sciences, University of Macau, Taipa, Macau

##### **Correspondence:** Jianbo Xiao; Xiaodong Zheng

*Journal of Chinese Medicine* 2018, **13(Suppl 1):**41

**Background:** The anthocyanins, belonging to a parent class of flavonoids, are neutral pH unstable chemical, which are easy to degrade in higher pH condition. Because of around-7-pH condition for most cell culture, the anthocyanins and their agylcons (anthocyanidins) shows weak stability in neutral cell-culture-condition. Herein, we characterized the stability of those ten anthocyanins (including anthocyanidins), which includes anthocyanins and their agylcons: cyanidin chloride and cyanidin 3-glucoside chloride; delphinidin chloride and delphinidin 3-glucoside chloride; malvidin chloride and malvidin 3-glucoside chloride; peonidin chloride and peonidin 3-glucoside chloride; petunidin chloride and petunidin 3-glucoside chloride, respectively, incubated with DMEM cell culture at 37 °C, 5% CO_2_.

**Result:** The anthocyanidins are instable and degraded within 10 min (^100^T_10_ < 1.0 min, ^100^T^50^ < 1.0 min), while cyanidin chloride (^100^T_10_ = 2.08 min, ^100^T_50_ = 6.19 min), delphinidin chloride (^100^T_10_ = 1.49 min, ^100^T_50_ = 3.13 min) and peonidin chloride (^100^T_10_ < 1 min, ^100^T_50_ = 8.13 min) are relatively more stable, but still degraded easily. However, on the other side anthocyanins with sugar moieties have high stability, whose values of ^100^T_50_ are almost larger than 180 min, except delphinidin 3-glucoside chloride (^100^T_50_ = 37.66 min).

**Conclusion:** In summary, the anthocyanidins were so instable and hardly to detect after first 10 min. The glycosylation at 3-position can largely enhance their stability. The methoxylation at ring B at 3′ and 5′ hydroxyl groups of anthocyanins can increase their stability. Compared to the methoxylation of 3′ hydroxyl group’s obvious enhancing the stability, the methoxylation of 5′ hydroxyl group just slightly increased the stability of anthocyanin. The hydroxylation at 3′ and 5′ positions on ring B of anthocyanins both decreased the stability. Compared to the hydroxylation of 3′ hydroxyl group’s significant weakening the stability, the hydroxylation of 5′ hydroxyl group just slightly decreased the stability of anthocyanin. The outcomes from the current experimental study could be utilized in further investigation on degradation or metabolism of anthocyanins and anthocyanidins under cell culture condition, and then might encourage further researches of actual bioactive compounds, which derived from anthocyanins and anthocyanidins.

**Funding:** This research was financially supported by the Start-up Research Grant from University of Macau (SRG2015-00061-ICMS-QRCM), and the opening fund of the State Key Laboratory of Quality Research in Chinese Medicine of University of Macau (No. SKL-QRCM-2014-2016).

## 42 Chromatographic fingerprints combined with chemometric methods reveal the chemical features of authentic Radix Polygalae

### Zhongquan Xin^1^, Dabing Ren^2^, Xiaojuan Zhang^3^, Zhibiao Yi^4^, LunZhaoYi^2^

#### ^1^Guangdong Provincial Key Laboratory of Animal Nutrition Control, Subtropical Institute of Animal Nutrition and Feed, College of Animal Science, South China Agricultural University, Guangdong, China; ^2^Yunnan Food Safety Research Institute, Kunming University of Science and Technology, Kunming, China; ^3^Central South University, College of Chemistry and Chemical Engineering, Changsha 410083, China; ^4^GuangZhou University of Chinese Medicine, Dongguan Mathematical and Engineering Academy of Chinese Medicine, Dongguan 523808, China

##### **Correspondence:** LunZhao Yi - yilunzhao@kmust.edu.cn

*Journal of Chinese Medicine* 2018, **13(Suppl 1):**42

**Background:** Radix Polygalae (RP) is the pharmaceutical name for the root of prescribed for the treatment of memory disorders and *Polygala tenuifolia* Willd. Traditionally, RP was mental diseases. In recent years, more bioactivities of RP have been published, such as its use as an anti-inflammatory [1] and antidepressant [2–4], and in the treatment of Parkinson disease[5] and Alzheimer disease[6]. Although RP is rich in biological activities, its chemical background has not been clear until now. Previous reports have focused on the research of saponins in RP [7]. Rarely, publications have related the research on another kind of bioactive component, i.e., volatiles, that may help to delay or reduce the insult to brain functions elicited by mental diseases [8].

**Materials and methods:** Thirty-two batches of RP samples included 20 batches of superior and 12 batches of inferior RP, were collected from Yunnan, Sichuan, and Changsha, China. The standards methyl eugenol (purity: 98%), undecane (purity, 99%), and α-asarone (purity, 98%) were purchased from Sigma. Alkane standard solution of C8–C20 was purchased from Fluka Chemika. Hexane and anhydrous sodium sulfate were of analytical grade.

Chromatographic fngerprints of essential oils from RP were obtained by GC–MS analysis. Two multivariate resolution methods, SFA and HELP [9, 10], were applied to resolve the qualitative and quantitative problem of overlapped peaks (Figs. 1, 2). Partial least-squares (PLS) discriminant analysis (DA) [11] coupled with regression coeffcient was applied to discriminate the superior RP samples from the inferior ones and to reveal the chemical features of authentic RP.

**Results:** A total of 88 volatile compounds were qualitatively and quantitatively analyzed (Table 1). The superior and inferior RP samples were separated well by the discriminant plane, with a correct rate of 100%, and the area under the receiver operating characteristic curve (AUC) value was 0.917. The data set was permutated for 5000 times and the frequency of correct rates for the permutated model is a normal distribution with the mean value about 45%, which guarantees the reliability of the recognition model. The four components, 1-octanol, shyobunone, isobornyl acetate, and α-asarone, were screened as the characteristic components to distinguish superior RP samples from the inferior ones (Fig. 3).

**Fig. 1:**
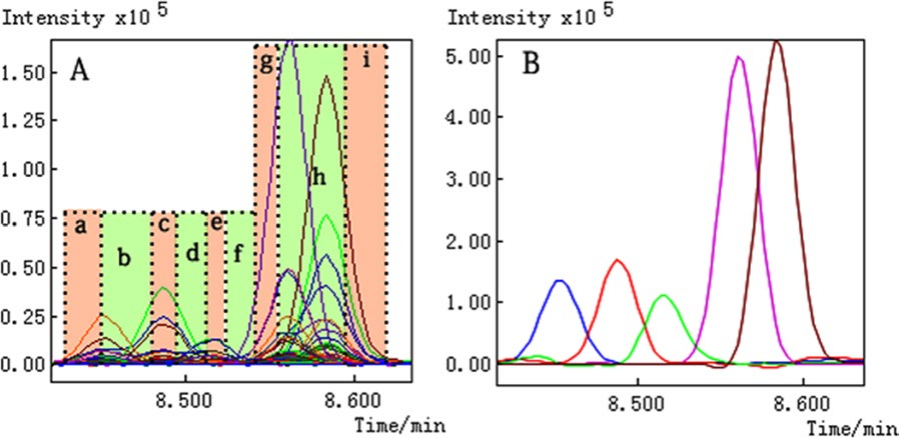
Resolution of the overlapped peaks by SFA

**Fig. 2:**
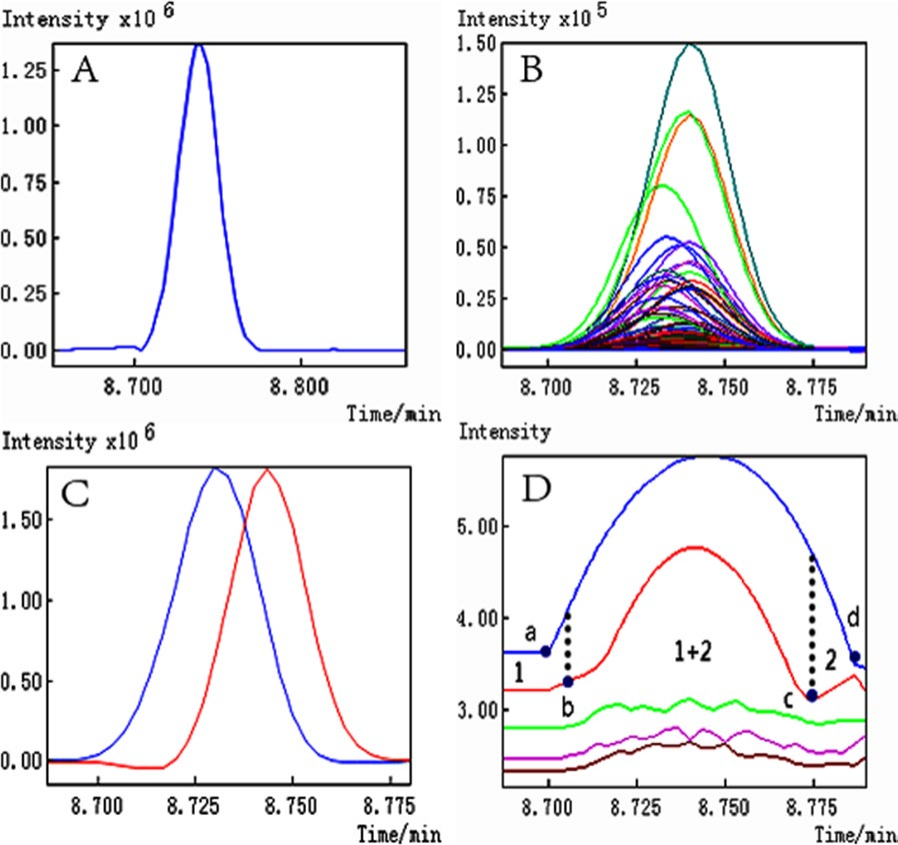
Resolution of the overlapped peaks by HELP

**Fig. 3:**
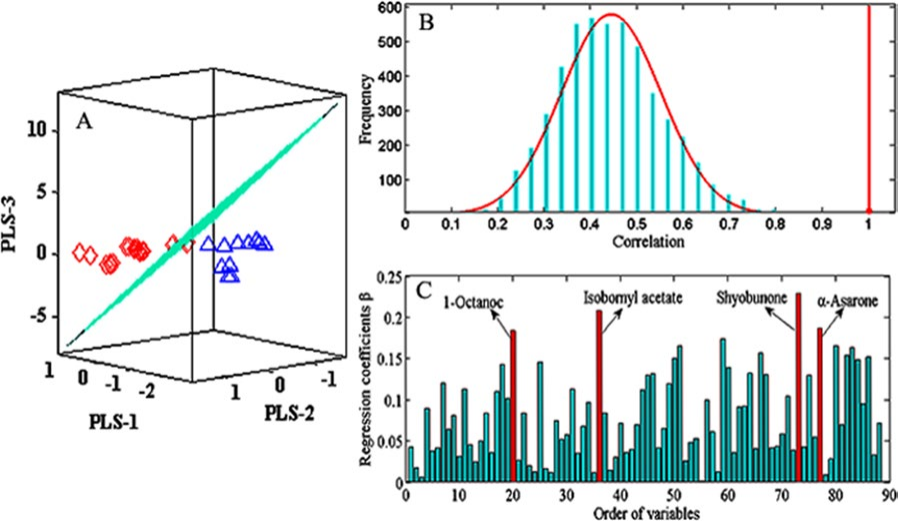
Discrimination analysis of the superior and inferior RP samples by PLS-DA

**Table 1:** Eighty-eight compounds of essential oils extracted from RPs

No.	RI	Name	Formula	Relative content (mean ± SD)		T
				Superior RPs	Inferior RPs	
1	–	Hexanal	C_6_H_12_O	3.888 ± 1.751	6.029 ± 6.369	0
2	801	2,4-Dimethylhexane	C_8_H_18_	0.516 ± 0.338	0.608 ± 0.537	0
3	818	1,3-Octadiene	C_8_H_14_	0.028 ± 0.013	0.034 ± 0.035	0
4	869	2-Heptanone	C_7_H_14_O	0.089 ± 0.037	0.134 ± 0.088	0
5	879	Heptanal	C_7_H_14_O	0.287 ± 0.085	0.365 ± 0.261	0
6	938	Benzaldehyde	C_7_H_6_O	0.241 ± 0.097	0.266 ± 0.134	0
7	942	2-Pinene	C_10_H_16_	0.154 ± 0.060	0.118 ± 0.073	0
8	958	Camphene	C_10_H_16_	0.593 ± 0.181	0.510 ± 0.317	0
9	966	1-Heptanol	C_7_H_16_O	0.043 ± 0.022	0.068 ± 0.051	0
10	970	1-Octen-3-one	C_8_H_14_O	0.047 ± 0.023	0.054 ± 0.024	0
11	975	2,3-Octadione	C_8_H_14_O_2_	0.055 ± 0.020	0.100 ± 0.081	1
12	979	1-Octen-3-ol	C_8_H_16_O	0.155 ± 0.066	0.148 ± 0.149	0
13	991	β-pinene	C_10_H_16_	0.100 ± 0.026	0.125 ± 0.104	0
14	999	2-Pentylfuran	C_9_H_14_O	1.116 ± 0.368	1.577 ± 1.293	0
15	1029	m-Cymene	C_10_H_14_	0.411 ± 0.105	0.391 ± 0.061	0
16	1037	1,8-Cineole	C_10_H_18_O	0.961 ± 0.202	0.976 ± 0.380	0
17	1040	(E)-β-Ocimene,	C_10_H_16_	0.407 ± 0.134	0.472 ± 0.253	0
18	1049	(Z)-β-Ocimene	C_10_H_16_	0.095 ± 0.057	0.048 ± 0.033	1
19	1060	g-Terpinene	C_10_H_16_	0.187 ± 0.034	0.163 ± 0.045	0
20	1063	1-Octanol	C_8_H_18_O	0.144 ± 0.063	0.209 ± 0.068	1
21	1069	Heptanoic acid	C_7_H_14_O_2_	0.069 ± 0.025	0.094 ± 0.099	0
22	1077	2-Nonanone	C_9_H_18_O	0.080 ± 0.031	0.109 ± 0.050	0
23	1088	Nonanal	C_9_H_18_O	2.414 ± 0.475	2.516 ± 0.923	0
24	1101	Undecane*	C_11_H_24_	2.062 ± 0.726	2.062 ± 1.008	0
25	1127	Camphor	C_10_H_16_O	0.846 ± 0.218	1.069 ± 0.621	0
26	1136	2-Nonenal	C_9_H_16_O	0.168 ± 0.058	0.192 ± 0.182	0
27	1159	Octanoic acid	C_8_H_16_0_2_	0.397 ± 0.234	0.507 ± 0.839	0
28	1173	Methyl salicylate	C_8_H_8_O_3_	2.352 ± 1.992	1.589 ± 1.681	0
29	1187	Methyl chavicol	C_10_H_12_O	22.858 ± 3.656	20.848 ± 6.880	0
30	1229	Anethole	C_10_H_12_O	0.113 ± 0.034	0.172 ± 0.206	0
31	1232	Cyclohexanone, 2-ethyl-	C_8_H_14_O	0.136 ± 0.079	0.339 ± 0.359	1
32	1236	2-Decenal	C_10_H_18_O	0.205 ± 0.142	0.330 ± 0.399	0
33	1244	1-(Trimethylsilyl)-1-propyne	C_6_H_12_Si	0.042 ± 0.016	0.082 ± 0.097	0
34	1261	Nonanoic acid	C_9_H_18_O_2_	1.224 ± 0.494	1.015 ± 1.142	0
35	1268	2,4-Decadienal^a^	C_10_H_16_O	0.203 ± 0.254	0.311 ± 0.374	0
36	1272	Isobornyl acetate	C_12_H_20_O_2_	0.068 ± 0.008	0.079 ± 0.019	1
37	1277	2-Methylnaphthalene	C_11_H_10_	0.077 ± 0.017	0.087 ± 0.027	0
38	1285	2-Methoxy-4-vinylphenol	C_9_H_10_O_2_	0.526 ± 0.202	0.535 ± 0.366	0
39	1292	2,4-Decadienal^a^	C_10_H_16_O	0.602 ± 0.713	1.043 ± 1.299	0
40	1300	8-Methyl-1-undecene	C_12_H_24_	0.088 ± 0.043	0.142 ± 0.138	0
41	1320	γ-Decalactone	C_10_H_18_O_2_	0.094 ± 0.064	0.093 ± 0.067	0
42	1330	Eugenol	C_10_H_12_O_2_	0.114 ± 0.051	0.084 ± 0.072	0
43	1341	2-Undecenal	C_11_H_20_0	0.147 ± 0.052	0.248 ± 0.249	0
44	1359	α-Longipinene	C_15_H_24_	0.230 ± 0.090	0.467 ± 0.840	0
45	1372	Methyl eugenol*	C_11_H_14_O_2_	2.187 ± 0.922	1.500 ± 1.061	0
46	1376	Ylangene	C_15_H_24_	0.063 ± 0.018	0.142 ± 0.230	0
47	1381	Longicyclene	C_15_H_24_	0.666 ± 0.253	0.615 ± 0.210	0
48	1393	β-Elemene	C_15_H_24_	0.620 ± 0.172	0.551 ± 0.239	0
49	1401	Tetradecane	C_14_H_30_I	0.061 ± 0.008	0.070 ± 0.019	0
50	1403	Paeonol	C_9_H_10_O_3_	0.217 ± 0.232	0.096 ± 0.040	0
51	1413	Longifolene	C_15_H_24_	0.155 ± 0.049	0.113 ± 0.039	1
52	1425	Methyl eugenol^a^	C_11_H_14_O_2_	5.482 ± 1.604	5.270 ± 2.608	0
53	1439	β-Gurjunene	C_15_H_24_	0.739 ± 0.271	0.776 ± 0.268	0
54	1449	β-Farnesene	C_15_H_24_	0.362 ± 0.135	0.322 ± 0.111	0
55	1457	Caryophyllene	C_15_H_24_	0.342 ± 0.108	0.334 ± 0.082	0
56	1462	Methyl eugenol^a^	C_11_H_14_O_2_	0.859 ± 0.379	0.566 ± 0.299	1
57	1468	β-Acoradiene	C_15_H_24_	0.184 ± 0.060	0.165 ± 0.042	0
58	1475	γ-Muurolene	C_15_H_24_	0.373 ± 0.124	0.350 ± 0.092	0
59	1483	shyobunone^a^	C_15_H_24_O	0.473 ± 0.129	0.589 ± 0.274	0
60	1488	β-Selinene	C_15_H_24_	0.775 ± 0.276	0.572 ± 0.244	1
61	1497	α-Guaiene	C_15_H_24_	1.146 ± 0.345	1.040 ± 0.320	0
62	1503	Shyobunone^a^	C_15_H_24_O	1.750 ± 0.419	1.852 ± 0.833	0
63	1511	γ-Muurolene	C_15_H_24_	0.758 ± 0.230	0.820 ± 0.251	0
64	1519	δ-Cadinene	C_15_H_24_	2.056 ± 0.566	2.338 ± 0.785	0
65	1529	Selina-3,7(11)-diene	C_15_H_24_	0.180 ± 0.088	0.202 ± 0.149	0
66	1533	α-Calacorene	C_15_H_20_	0.635 ± 0.180	0.753 ± 0.281	0
67	1537	Elemicin^a^	C_12_H_16_O_3_	0.896 ± 0.430	0.569 ± 0.329	1
68	1556	Germacrene B	C_15_H_24_	0.439 ± 0.123	0.388 ± 0.136	0
69	1561	Alloaromadendrene	C_15_H_24_	0.174 ± 0.069	0.181 ± 0.064	0
70	1568	Spathulenol	C_15_H_24_O	0.211 ± 0.078	0.226 ± 0.103	0
71	1575	Caryophyllene oxide	C_15_H_24_O	0.131 ± 0.062	0.165 ± 0.112	0
72	1585	Elemicin^a^	C_12_H_16_O_3_	6.446 ± 2.360	5.283 ± 3.081	0
73	1596	Shyobunone^a^	C_15_H_24_O	0.741 ± 0.282	1.156 ± 0.635	1
74	1608	Isolongifolene	C_15_H_24_	0.170 ± 0.054	0.164 ± 0.060	0
75	1620	Dehydroxy-isocalamendiol	C_15_H_24_O	0.340 ± 0.082	0.381 ± 0.137	0
76	1629	α-Muurolol	C_15_H_26_O	0.205 ± 0.066	0.179 ± 0.041	0
77	1639	α-Asarone*	C_12_H_16_O_3_	0.620 ± 0.314	0.270 ± 0.099	1
78	1651	Dehydroxy-isocalamendiol	C_15_H_24_O	0.135 ± 0.037	0.135 ± 0.026	0
79	1662	Aristol-9-en-8-one	C_15_H_22_O	0.848 ± 0.366	0.767 ± 0.426	0
80	1671	Spiro[4.5]dec-6-en-8-one, 1,7-dimethyl-4-(1-methylethyl)-	C_15_H_24_O	0.391 ± 0.109	0.483 ± 0.353	0
81	1726	Succinic anhydride, (2-dodecenyl)-	C_16_H_26_O_3_	0.185 ± 0.093	0.124 ± 0.081	0
82	1746	Phenanthrene	C_14_H_10_	0.118 ± 0.053	0.134 ± 0.033	0
83	1843	3,3′-Dimethoxy-1,1′-biphenyl	C_14_H_14_O_2_	0.155 ± 0.109	0.057 ± 0.031	1
84	1910	Hexadecanoic acid, methyl ester	C_17_H_34_O_2_	0.246 ± 0.153	0.177 ± 0.054	0
85	1954	Hexadecanoic acid	C_16_H_32_O_2_	4.857 ± 2.788	3.365 ± 1.613	0
86	–	Methyl linoleate	C_19_H_34_O_2_	0.675 ± 0.698	0.341 ± 0.144	0
87	–	Methyl oleate	C_19_H_34_O_2_	0.956 ± 0.748	0.912 ± 0.221	0
88	–	Oleic acid	C_18_H_34_O_2_	16.262 ± 5.442	18.974 ± 7.201	0

* Identified by the standard substances; ^a^ isomer is unidentified

**Conclusions:** In this study, a reliable and comprehensive strategy has been proposed to identify essential oils in RP by GC–MS combined with chemometrcics. This strategy shown effective mothed to revealing the chemical features of a complex analytical sample such as RP.

**References**

1. Cheong MH, Lee SR, Yoo HS, Jeong JW, Kim GY, Kim WJ, et al. Anti-inflammatory effects of Polygala tenuifolia root through inhibition of NF-kappaB activation in lipopolysaccharide-induced BV2 microglial cells. J Ethnopharmacol. 2011;137(3):1402–8.
2. Kawashima K, Miyako D, Ishino Y, Makino T, Saito K-i, Kano Y. Anti-stress effects of 3, 4, 5-trimethoxycinnamic acid, an active constituent of roots of Polygala tenuifolia (Onji). Biol Pharm Bull. 2004; 27(8):1317–9.
3. Liu P, Hu Y, Guo DH, Wang DX, Tu HH, Ma L, et al. Potential antidepressant properties of Radix Polygalae (Yuan Zhi). Phytomedicine. 2010; 17(10):794–9.
4. Jin ZL, Gao N, Zhang JR, Li XR, Chen HX, Xiong J, et al. The discovery of Yuanzhi-1, a triterpenoid saponin derived from the traditional Chinese medicine, has antidepressant-like activity. Prog Neuropsychopharmacol Biol Psychiatry. 2014; 53:9–14.
5. Choi JG, Kim HG, Kim MC, Yang WM, Huh Y, Kim SY, et al. Polygalae radix inhibits toxin-induced neuronal death in the Parkinson’s disease models. J Ethnopharmacol. 2011; 134(2):414–21.
6. Wu TY, Chen CP, Jinn TR. Traditional Chinese medicines and Alzheimer’s disease. Taiwan J Obstet Gynecol. 2011; 50(2):131–5.
7. Wen L, Xia N, Tang P, Hong Y, Wang Z, Liu Y, et al. The gastrointestinal irritation of polygala saponins and its potential mechanism in vitro and in vivo. Biomed Res Int. 2015; 2015:918048.
8. Youdim KA, Martin A, Joseph JA. Essential fatty acids and the brain: possible health implications. Int. J. Dev. Neurosci. 2000; 18(4–5):383–99.
9. Kvalheim OM, Liang YZ. Heuristic evolving latent projections: resolving two-way multicomponent data. 1. Selectivity, latent-projective graph, datascope, local rank, and unique resolution. Anal. Chem. 1992; 64(8):936–46.
10. Manne R, Shen H, Liang Y. Subwindow factor analysis. Chemometr Intell Lab. 1999; 45(1):171–6.
11. Yi L, Dong N, Yun Y, Deng B, Ren D, Liu S, et al. Chemometric methods in data processing of mass spectrometry-based metabolomics: A review. Anal Chim Acta. 2016; 914:17–34.

## 43 Preparation and Research of Konjac Glucomannan-Hairtail Iron Peptide by Microfluidic Technology

### Yongsheng Ni, Wanmei Lin, Lin Wang, Ruojun Mu, Yi Yuan, Chunhua Wu, Jie Pang

#### College of Food Science, Fujian Agriculture and Forestry University, Fuzhou 350002, China

##### **Correspondence:** Jie Pang - pang3721941@163.com

*Journal of Chinese Medicine* 2018, **13(Suppl 1):**43

**Background:** The exploration of methods allowing iron supplement to be prepared by Hairtail waste is of great scientific and technological interest. Microfluidic systems have provided an ideal microreactor platform to produce hairtail iron peptide (HPH) due to their simplified manipulation, high efficiency, flexible controllability, and environmental-friendly chemical process [1].

**Materials and methods:** Here a novel polysaccharide microfiber reactor based on a microfluidic spinning technique for in situ fabrication of an excellent biological activity iron supplement is demonstrated. A dense band or network structure could be formed by the konjac glucomannan (KGM) self-assembly of the molecules inner group or the cross-linking polymerization of other polymers. Macromolecule self-assembly reactions are carried out in coaxial flow-based microdevices with different microchannel. Bead structure was formed by hairtail protein hydrolysates and ferrous ions in T shape microchannel and then net structure was formed by KGM and polyvinyl pyrrolidone in Y shape microchannel. The ferrous ions was set in net structure tightly (Fig. 1). Moreover, the antioxidant activity was studied systematically by vitro chemical experiments and mice experiments.

**Results:** Results revealed that reticular konjac glucomannan hairtail iron peptide microfiber (KGM-Fe-HPH) had better reducing power, O^2−^ scavenging ability and DPPH scavenging than hairtail iron peptide. The activity of superoxide dismutase (SOD), glutathione peroxidase (GSH-Px) and catalase (CAT) were improved obviously. The negative performance and growth of MDA caused by D-Gal of mice were relieved by KGM-Fe-HPH. The structural deformation of hepatocytes, the outflow of fluid, the enlargement and aggregation of cells intercellular space were repaired by KGM-Fe-HPH (Fig. 2).

**Fig. 1:**
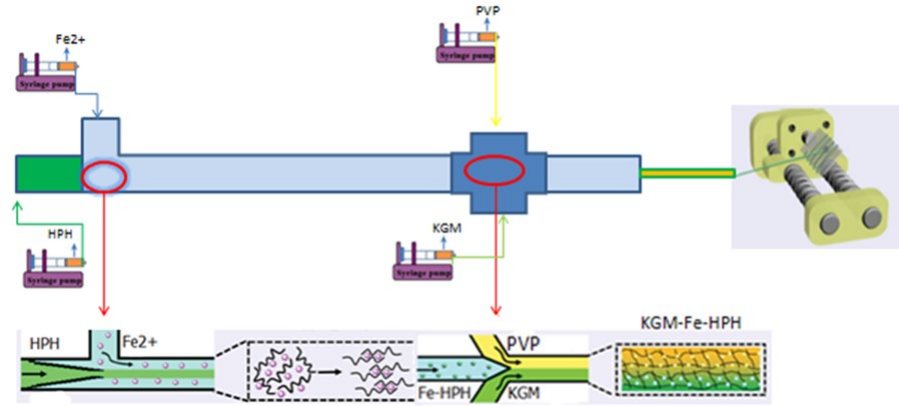
Preparation of konjac glucomannan hairtail iron peptide microfiber

**Fig. 2:**
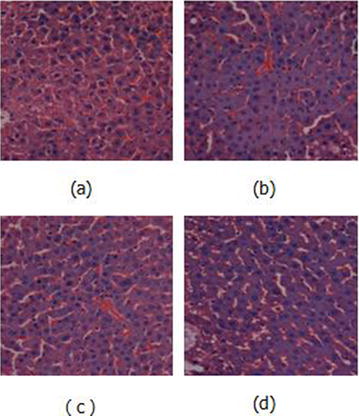
Effects of KGM-Fe-HPH on the hepatic tissue of ICR mice treated by D-Gal (**a** normal, **b** D-Gal, **c** KGM-Fe-HPH, **d** VE)

**Conclusions:** This strategy firstly contributes a facile and environmental-friendly route to fabricate polysaccharide microfiber. Moreover, an excellent biological activity iron supplement was fabricated by the HPH uniform dispersion and activity protection in microreactor platform, which opens a promising avenue to protect sensitive active substances.

**Reference**

1. Zhang Y, Wang C, Chen L, Chen S, Ryan A. Microfluidic-Spinning-Directed Microreactors Toward Generation of Multiple Nanocrystals Loaded Anisotropic Fluorescent Microfibers. Advanced Functional Materials. 2015; 25:7253–62.

## 44 C-type starch and its derivatives: Structure and function

### Xiangze Jia^1,2^, Zebin Guo^1,2^, Beibei Zhao^1,2^, Shaoxiao Zeng^1,2^, Baodong Zheng^1,2^, Jianbo Xiao^3^

#### ^1^College of Food Science, Fujian Agriculture and Forestry University, Fuzhou, Fujian 350002, China; ^2^Fujian Provincial Key Laboratory of Quality Science and Processing Technology in Special Starch, Fuzhou, Fujian 350002, China; ^3^Institute of Chinese Medical Sciences, State Key Laboratory of Quality Research in Chinese Medicine, University of Macau, Taipa, Macau

##### **Correspondence:** Baodong Zheng - zbdfst@163.com; Jianbo Xiao - jianboxiao@umac.mo

*Journal of Chinese Medicine* 2018, **13(Suppl 1):**44

The C-type starches are widely distributed in seeds or rhizomes of various legumes, traditional Chinese medicinal plants, and many potential medicinal crops. This carbohydrate polymer directly affects the application of starchy plant resources. Structure and crystallinity properties are crucial parameters of starch granules, which directly affect physicochemical and mechanical properties of starch materials and the performance of starch-based additives. The unique crystal structure consisting of both A- and B-type polymorphs endows C-type starches with specific crystal adjustability. Additionally, large proportions of resistant starch and slow digestible starch are found in C-type starch and contribute to benign glycemic response and proliferation of gut microflora. In this paper, we initially briefly review the distribution of C-type starch in various plant sources. We then introduce several structural models of C-type starch and its crystal properties. We also discuss the behavior and functionality relevant to modified C-type starch. Ultimately, we outline recent advances, potential applications, and limitations of C-type starch in industry, aiming to provide a theoretical basis for further research and to broaden the prospects of its applications.

## 45 Probiotic effect of lotus seed resistant starch prepared by different methods on *Bifidobacterium longum* and *Lactobacillus bulgaricus*

### Cancan Huang, Hongliang Zeng, Baodong Zheng, Yi Zhang

#### College of Food Science, Fujian Agriculture and Forestry University, Fuzhou, Fujian, 350002, China

##### **Correspondence:** Baodong Zheng; Yi Zhang

*Journal of Chinese Medicine* 2018, **13(Suppl 1):**45

**Structure abstracts:** Effect of lotus resistant starch on proliferation rate of *B. longum* and *L. bulgaricus*.

The determination of bifidobacterium and lactobacillus metabolites

**Background:** Resistant starch (RS) consists of starch and products of starch digestion that are poorly absorbed by the small intestine6 but are completely or partially fermented in the colon.7 RS is now categorized into ve RS types:8 RS1, which consists of starch that is difficult to digest due to its structural rigidity; RS2, which consists of starch granules in raw foods that are resistant to digestion; RS3, which consists of retrograded starch formed during the cooling process of gelatinized starch; RS4, which is chemically modified starch; and RS5, which is starch–lipid complexes. RS could not only reduce blood glucose and insulin levels but also increase fecal bulk and short chain fatty acid (SCFA) production during fermentation in the large intestine.

Therefore, the study of RS, especially RS3, has attracted a lot of interest within the food and nutrition scientific community.

**Materials and methods:** *B. longum* and *L. bulgaricus* were purchased from China’s industrial microbial preservation management center.

Lotus seed native starch and GP-LRS3 were prepared using the previous method reported by Zhang et al. (2014).

MP-LRS3: lotus seed native starch (150 g) was dispersed in 1000 ml distilled water (starch: water, 3:20), and the starch suspensions were heated under 640 W of microwave power for 2 min, cooled to room temperature, and stored at 4 C for 24 h. UP-LRS3: lotus seed native starch (450 g) was dispersed in 1000 ml distilled water (starch: water, 9:20), and the starch suspensions were packed with vacuum package machine, and then treated with an ultrasonic transducer at the ultrasonic power of 300 W for 55 min at 25 C. After that, the mixture was pressure-cooked in an autoclave at 115 C for 15 min, cooled to room temperature, and stored at 4 C for 24 h.

Lactic acid, acetic acid, propionic acid, butyric acid was purchased from Aladdin Reagent Co., LTD.

Glucose, potassium hydroxide and ice acetic acid, sodium hydroxide were obtained from Sinopharm Chemical Reagent Co., Ltd.

**Results:** The results showed that compared with the media containing GLU or HAMS, the media containing GP-LRS3, UP-LRS3 or MP-LRS3 displayed the significantly higher probiotic effect on *B. longum* and *L. bulgaricus*. *B. longum* and *L. bulgaricus* had an 8 h lag phase in the medium with GP-LRS3, UP-LRS3 or MP-LRS3, whereas the lag phase in the media with GLU or HAMS was 16 h. Besides, the content of butyric acid in the medium with GP-LRS3, UP-LRS3 and MP-LRS3 during the *B. longum* fermentation process was significantly higher than that in the media with GLU or HAMS, and the positive effect on the capability of butyric acid production from *B. longum* were: MP-LRS3 > UP-LRS3 > GP-LRS3. The content of butyric acid and propionic acid have no significant increase in all media during the *L. bulgaricus* fermentation process.

**Conclusions:** The conclusions from the above results were as follows: firstly, the proliferation of probiotics was significantly better than that of HAMS and GLU on *B. longum* and *L. bulgaricus*, but the probiotic effect of LRS3 has no significant difference between them on *L. bulgaricus*; then, *B. longum* had longer logarithmic growth phase in the medium with GP-LRS3; finally, two probiotics use LRS3 to produce short-chain fatty acids in different capacities, *B. longum* is superior to *L. bulgaricus*.

## 46 Comparison of total flavonoids content, antioxidant activity of extracts of *Woodwardia japonica* L. collected from 9 different regions

### Xin Wang^1,2^, Jianbo Xiao^2,3^, Jianguo Cao^2^, Quanxi Wang^2^

#### ^1^Insititute of Applied Ecology, Chinese Academy of Sciences, Shenyang 110016, China; ^2^Department of Biology, Shanghai Normal University, 100 Guilin Rd, Shanghai 200234, China; ^3^State Key Laboratory of Quality Research in Chinese Medicine, Institute of Chinese Medical Sciences, University of Macau, Taipa, Macau

##### **Correspondence:** Quanxi Wang

*Journal of Chinese Medicine* 2018, **13(Suppl 1):**46

**Background:** Flavonoid extracts of ferns were not only conductive to phylogeny and genesiology, but with significant pharmacological activities [1]. Flavonoids of higher plants could be altered by the changes of ecological factors. Nevertheless, the effects of ecological factors on the flavonoid content and antioxidant activity of ferns remain unclear. Here total flavonoid contents and antioxidant activities of the extracts of *Woodwardia japonica* from 9 different places were compared.

**Materials and methods:** *W. japonica* was chosen for the experiment because of the highest content in our previous reported [2]. Radical scavenging assay (DPPH, ABTS, O^2−^), FRAP, reducing power assay were adopted.

**Results:** The differences between flavonoid contents and bioactivities of *W. japonica* collected from 9 different regions are obvious (Fig. 1). Total flavonoid content of the species from Wuyi Mountain in Jiangxi province was the highest (34.54%), while that from Yandang Mountain in Zhejiang province was the lowest (11.45%). Species living under adequate sunshine in high altitude region were with higher total flavonoid content and antioxidant activity, and vice versa. The results showed that sunshine and altitude were positive correlation with total flavonoid contents. The extracts from *W. japonica* from 9 different regions all showed strong antioxidant activity (Fig. 2). Furthermore, antioxidant activity of species from Wuyi Mountain in Jiangxi province was obviously stronger than those from Yandang Mountain in Zhejiang province. Species living under adequate sunshine in high altitude region of *W. japonica* were with higher total flavonoid content and antioxidant activity.

**Fig. 1:**
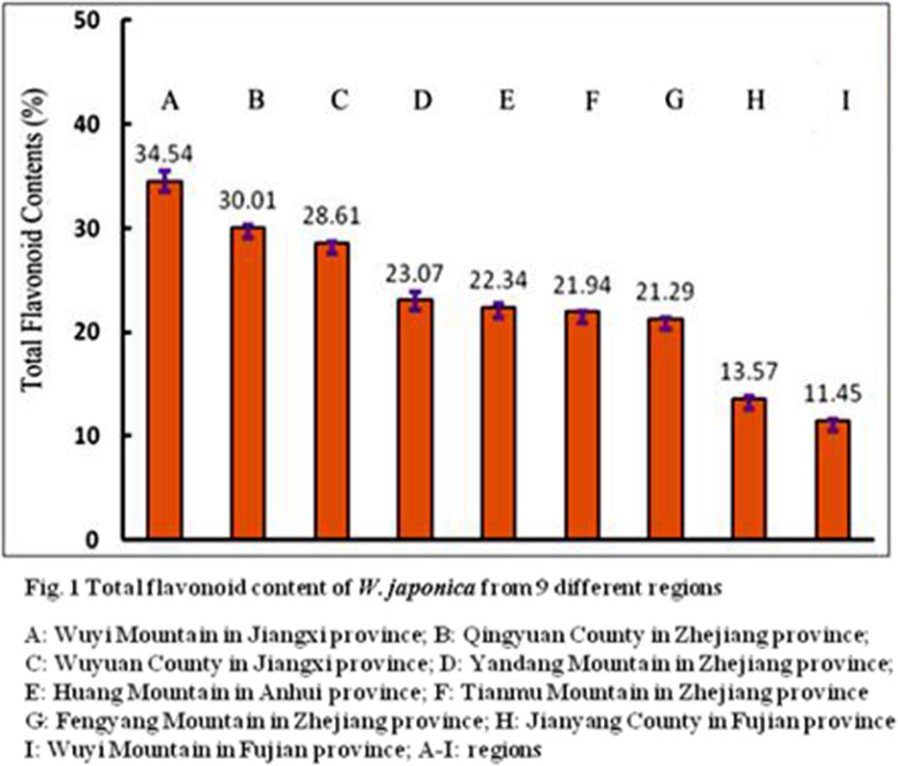
See text for description

**Fig. 2:**
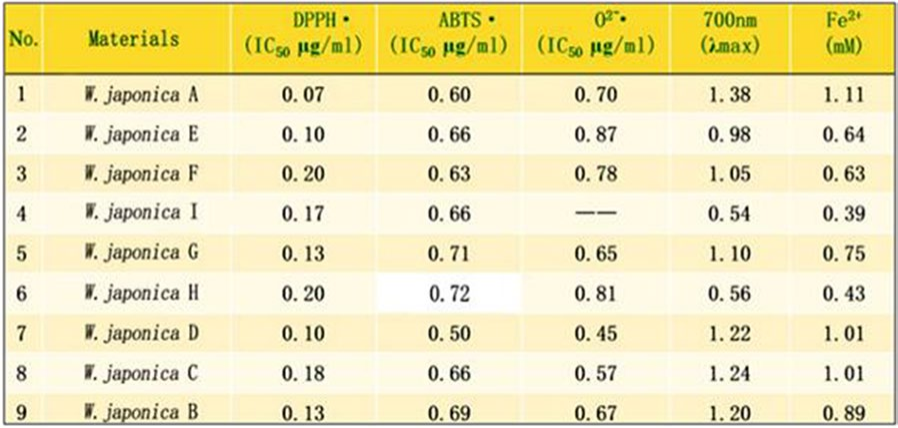
Antioxidant activity of extracts from *W. japonica* from 9 different regions

**Conclusion:** The total flavonoid contents and antioxidant activity of extracts from *W. japonica* were relative to ecological factors.

**References**

1. Cao H, Chai TT, Wang X, Morais-Braga MFB, Yang JH, Wong FC, Wang RB, Yao HK, Cao JG, Cornara L, Burlando B, Wang YT, Xiao JB. Coutinho HDM. Phytochemicals from fern species: potential for medicine applications. Phytochem Rev. 2017; 16:379–440.
2. Wang X, Wang Ml, Cao JG, Wu YH, Wang QX. Total flavonoid concentrations of bryophytes from Tianmu Mountain, Zhejiang Province (China): Phylogeny and ecological factors. Ind Crop Prod. 2017; 97:137–145.

## 47 Phenolic profiles in fruits and seeds, and their contribution to antioxidant activities of black goji (*Lycium ruthenicum* Murr.) and red goji (*Lycium barbarum* L.) grown in northwest of China

### Fengmei Zhu, Bin Du

#### Hebei Normal University of Science and Technology, Qinhuangdao, Hebei 066600, China

##### **Correspondence:** Fengmei Zhu - fmzhu@aliyun.com; Bin Du - bindufood@aliyun.com

*Journal of Chinese Medicine* 2018, **13(Suppl 1):**47

**Background:** As the fruits of Solanaceae family shrub plant, goji (also called as wolfberry) has been used as traditional medicinal foods in China and other Asian countries for centuries [1]. Red goji (*Lycium barbarum* L.), is a perennial, deciduous shrub growing northwest China and the Mediterranean region [2]. Black goji (heiguogouqi in Chinese) is a black color small berry fruit from (*L. ruthenicum* Murr.) natively growing in northwest part of China. Furthermore, the information on the effect of goji color and the effect of boiling on phytochemical distribution of goji is very few.

**Materials and methods:** The objective of this study was to investigate and compare the phytochemical profiles and health benefits between red and black goji, and discuss the distribution of phytochemicals in fruits and contribution of goji pulp and seeds to the antioxidants of whole goji. Pulp and seeds from four varieties of black goji and two varieties of red goji with different geographical origins were examined for their total phenolic content (TPC), total flavonoid content (TFC), condensed tannin content (CTT), monomeric anthocyanin content (MAC), 2,2-diphenyl-1-picrylhydrazyl (DPPH) scavenging capacity, and ferric reducing antioxidant power (FRAP) using colorimetric methods.

**Results:** Contribution rates of pulp and seed to phytochemical contents and overall antioxidant capacities of whole fruits were calculated for each parameter. Effects of boiling processing on these parameters were also evaluated. It was observed that most of the phytochemicals and antioxidant activities were predominantly contributed by the pulp in all six varieties.

**Conclusions:** Boiling led to significant (*p *< 0.05) losses in the phytochemical content and antioxidant capacity. The average MAC value in black goji was significantly (*p *< 0.05) higher than that observed for red goji.

**References**

1. Potterat O. Planta Med. 2010; 76:7–19.
2. Zhao J, Li H, Xi W, et al. Food Chem. 2015; 173:718–24.

## 48 Critical review in development of terpenoids as key enzyme inhibitors relevant for hyperglycemia

### Hui Teng^1^, Kang Li^1^, Benyao Yuan^1^, Young-Hwa Kang^2^, Lei Chen^1^

#### ^1^College of Food Science, Fujian Agriculture and Forestry University, Fuzhou, Fujian 350002, China; ^2^Division of Applied Biosciences, College of Agriculture & Life Sciences, Kyungpook National University, Daegu, Republic of Korea

##### **Correspondence:** Lei Chen - chenlei841114@hotmail.com

*Journal of Chinese Medicine* 2018, **13(Suppl 1):**48

**Background:** Nowadays, importance for terpenoids in food and pharmaceutical industries is mainly based on their potential effects on diabetes treatment. α-Amylase and α-glucosidase are key enzymes responsible for the hydroxylation of oligosaccharides and conversion of disaccharides into glucose which are suitable for absorption.

**Material and method:** In this review, we summarized the latest developments on the inhibitory activities of terpenoids as well as terpene-rich herbs against key enzymes relevant to hyperglycemia, and discussed their underlying molecular mechanisms of anti-diabetic potential.

**Results:** According to extensive comparison of relative tepenoid compounds, we suggested a better evaluation of the pharmacological profile of terpenes and their derivatives with a clear-cut choice of possible human pathologies.

**Conclusion:** Future studies aimed at enhancing the absorption of terpenoids or their metabolites are likely to be necessary for their ultimate uses for chemo-therapy effect.

## 49 Phytochemicals in hops in preventing and managing of metabolic syndrome

### Pavel Dostálek, Marcel Karabín, Lukáš Jelínek

#### Department of Biotechnology, University of Chemistry and Technology, Prague, Technická 5, 16628 Prague 6, Czech Republic

##### **Correspondence:** Pavel Dostálek - Pavel.Dostalek@vscht.cz

*Journal of Chinese Medicine* 2018, **13(Suppl 1):**49

**Background:** In many countries, the prevalence of metabolic disease has attained epidemic proportions because of cardiovascular complications and mortality. A metabolic syndrome is associated with risk from 5 factors: abdominal (central) obesity, elevated blood pressure, elevated plasma glucose, high serum triglycerides, and low high-density lipoprotein levels. Treatment is focused on reduction of the risk of heart disease by lowering LDL cholesterol and reducing high blood pressure, and then on treatment of diabetes. Very important is a reduction in weight by proper diet and exercise [1].

**Results:** Beneficial effects of hop phenolic compounds for diabetic patients consist of improving blood glucose and lipid profiles, and reducing insulin resistance. Phenolic compounds exert antiobesity effects that are closely linked with antioxidant effects, through their ability to modulate lipid and energy metabolism and thus enable weight loss and reduce obesity. Regulation of cholesterol metabolism by phenolic compounds may reduce certain factors linked with hypercholesterolemia and dyslipidemia. Prenylflavonoids such as xanthohumol have been demonstrated to have strong antiobesity activities including the ability to inhibit diacylglycerol acyltransferase in rat liver, to inhibit triglyceride transport using a HepG2 cell line, and to inhibit the secretion of apolipoprotein B, the main constituent of the cholesterol LDL fraction. Therapeutic potential of polyphenols as treatment for obesity is also associated with the ability to inhibit α-glucosidase, an enzyme that controls blood glucose levels [1, 2]. Iso-α-acids are able to improve health by influencing lipid metabolism, glucose tolerance, and body weight. Diabetic mice treated with iso-α-acids showed reduced plasma glucose, triglyceride, and free fatty acid levels by 65.3, 62.6, and 73.1%, respectively. When mice were fed hop iso-α-acids in their diet, with high levels of cholesterol, an increase in plasma HDL-cholesterol and a reduction in cholesterol and triacylglycerol content in the liver were observed. The modulatory effect of iso-α-acids on lipid metabolism may also be responsible for lost body weight [1].

**Conclusions:** For treatment of metabolic syndrome should be very effective hop substances from group of polyphenols and bitter acids.

**References**

1. Karabín M, Hudcová T, Jelínek L, et al. Compr Rev Food Sci Food Saf. 2016;15:542–67.
2. Karabín M, Hudcová T, Jelínek L, et al. Biotechnol Adv. 2015;33:1063–90.

## 50 Nuciferine ameliorates endothelial dysfunction via PPARgamma activation in high-fat diet/streptozocin induced type II diabetes mice

### Jianjun Deng^1^, Chao Zhang^2,3^, Dan Liu^2^, Qingqing Liu^1^, Haixia Yang^2,3^

#### ^1^Shaanxi Key laboratory of Degradable Biomedical Materials, College of Chemical Engineering, Northwest University, Xi’an, 710069, China; ^2^Cardiovascular Research Center, Xi’an Jiaotong University, Xi’an, 710061, China; ^3^Nutrition and Food Safety Engineering Research Center of Shaanxi Province, College of Public Health, School of Medicine, Xi’an Jiaotong University, Xi’an, 710061, China

##### **Correspondence:** Haixia Yang

*Journal of Chinese Medicine* 2018, **13(Suppl 1):**50

**Background:** Endothelial dysfunction has been suggested as a possible causal link between in cardiovascular complications diabetes mellitus. Nuciferine (Nuci), a major active component from lotus leaf, may elicit cardiovascular protection, however, the underlying mechanism remains unclear. Here, we investigated the ameliorative effect of Nuci with two doses (0.06 and 0.12%) on type II diabetes mice induced by high-fat diet (HFD) and multiple low doses intraperitoneal injection of streptozocin (STZ).

**Materials and methods:** Type 2 diabetic mice induced by high fat and low dose of streptozotocin (STZ, 35 mg/kg) and were administered Nuci for 13 weeks. The fasting blood glucose (FBG) and body weight were tested. Acetylcholine (Ach) induced endothelium-dependent relaxation was measured in aortas for estimating endothelial function. The level of serum insulin was measured by enzyme linked immunosorbent assay. The NO levels in aortas were determined according to the manufacturer’s instructions. In addition, endothelial NO synthase (eNOS), phosphorylated-eNOS (p-eNOS), Akt, and phosphorylated-Akt (p-Akt) levels were also evaluated in endothelial cells.

**Results:** Nuci significantly reduced blood glucose level by ~ 30% and attenuated insulin resistance by 22% compared with HFD/STZ group. For estimating endothelial function, aortas endothelium-dependent relaxation results indicated the aortas in Nuci treatment group (0.12% Nuci) mice had better relaxing ability than that in HFD/STZ group mice. Meanwhile, nuci (3-30 μmol/L) dose-dependently restored the endothelial dysfunction and increased Akt-Ser473 phosphorylation, accompanied by increasing eNOS-Ser1177 phosphorylation and NO production in human umbilical vein endothelial cells in vitro.

**Conclusions:** Our finding indicated that Nuci can prevent and improve hyperglycemic status in HFD/STZ-induced diabetic mice and the mechanism may depend on ameliorating the endothelial dysfunction via the Akt/eNOS pathway.

**Keywords**: Nuciferine, Endothelial dysfunction, PPARgamma, type II diabetes.

**Funding:** This study was supported by the National Natural Science Foundation of China (21476184 and 21676212).

## 51 Supramolecular encapsulation of pesticides

### Ruibing Wang, Xue Yang

#### State Key Laboratory of Quality Research in Chinese Medicine, Institute of Chinese Medical Sciences, University of Macau, Taipa, Macau SAR

##### **Correspondence:** Ruibing Wang - rwang@umac.mo

*Journal of Chinese Medicine* 2018, **13(Suppl 1):**51

**Background:** Pesticides have been used as a major means of pest control for nearly a century in agriculture for higher-yield and higher-quality crop. However, it has been clear that extended exposure to pesticides would harm to environment and human health. For instance, it was reported that extended exposed of anabasine (ANA) caused different degree of toxic signs, and even resulted in the fetal cleft palates in pregnant goats [1, 2]. In this study, we aimed to investigate the binding behaviors between cucurbit[7]uril (CB[7]) and ANA, and examine the influence of CB[7]’s encapsulation on the toxicity of the pesticide.

**Materials and methods:** The chemical behaviors between CB[7] and ANA were examined by ^1^H NMR, electrospray ionization mass spectrometry (ESI–MS), UV–visible absorbance spectroscopy, isothermal titration calorimetry (ITC), and molecular modeling. Zebrafish embryos were selected as in vivo models to investigate the toxicity.

**Results:** ^1^H NMR, ESI–MS, as well as ITC suggested that ANA formed 1:1 binding complexes with CB[7], with a relatively strong binding affinity (10^5^ M^−1^). Regarding the toxicity of the pesticide, as shown in Fig. 1A, compared with the control group, the viable hatching rate of embryos treated with 300 μM ANA was significantly lower, (ca. 20%), suggesting dramatic embryonic toxicity of ANA. In contrast, a much higher hatching rate (ca. 90%) was observed when ANA was encapsulated by CB[7]. Similar trend was observed in the survival rate of embryos treated with a variety of concentrations of ANA in the absence and in the presence of CB[7] (Fig. 1B).

**Fig. 1:**
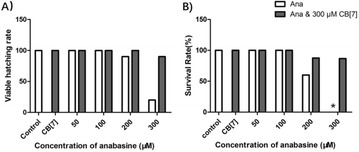
Viable hatching rate (**A**) and survival rate (**B**) of zebrafish exposed with various concentrations of ANA in the absence and in the presence of 300 μM CB[7]for 48 h. ‘*’ means zebrafish embryos in this group all died at the end of exposure

Moreover, relatively high concentrations (200–300 μM) of ANA induced serious physical malformations during the embryonic development, including retarded growth, yolk sac edema, spinal deformation, and yolk sac deformity and tail malformation. In contrast, in the presence of CB[7], the treated embryos didn’t show any obvious malformation compared with the free ANA group. The results demonstrated that CB[7] can decrease the teratogenic effect caused by ANA.

**Conclusions:** In summary, our results demonstrated with in vivo zebrafish models that supramolecular encapsulation of pesticides by CB[7] may significantly reduce the fetal and developmental toxicities of these pesticides.

**Acknowledgements:** Macau Science and Technology Development Fund (FDCT Grant No.: FDCT/020/2015/A1) is gratefully acknowledged for providing financial support.

**References**

1. Keeler RF, Crowe MW. Teratogenicity and toxicity of wild tree tobacco, Nicotiana glauca in sheep. Cornell Vet. 1984; 74:50–9.
2. Stein S, Dunsche A, Gellrich NC, Harle F, Jonas I. One- or two-stage palate closure in patients with unilateral cleft lip and palate: comparing cephalometric and occlusal outcomes. Cleft Palate Craniofac J. 2007; 44:13–22.

## 52 Isolation and identification of new chemical constituents from Chinese chive (*Allium tuberosum*) and toxicological evaluation of raw and cooked Chinese chive

### Quan Gao^1^, Xia-Bing Li^2^, Jia Sun^2^, Er-Dong Xia^2^, Feng Tang^2^, Hai-Qun Cao^1^, Hang Xun^2^

#### ^1^School of Plant Protection, Anhui Agricultural University, Hefei 230036, China; ^2^State Forestry Administration Key Open Laboratory, International Centre for Bamboo and Rattan, Beijing 100102, China

##### **Correspondence:** Jia Sun- sunjia@icbr.ac.cn

Quan Gao, Xia-Bing Li and Jia Sun contributed equally to this work

*Journal of Chinese Medicine* 2018, **13(Suppl 1):**52

**Background:** Chinese chive (*jiu cai*) is a popular vegetable in China and has a unique flavour and aroma [1]. The molecular basis of the characteristic fragrance and nutritional properties of Chinese chive has not been previously identified [2–4].

**Materials and methods:** Sequential extractions in a series of solvents and high-performance liquid chromatography were used to isolate 40 compounds from Chinese chive. The compounds were identified based on high-resolution electrospray ionization mass spectra, 1D and 2D nuclear magnetic resonance techniques, and circular dichroism spectra.

**Results:** Eight novel compounds were identified—four new pyrazines (Fig. 1), which have distinctive flavour; one new lignan; and three new flavonoids (Fig. 2)—together with 32 known compounds. Several of these compounds have potential applications as health-promoting dietary supplements, food additives, or seasonings. Additionally, the volatile organic compounds in fresh and steamed Chinese chive were compared, and the toxicological activity of extracts from fresh and steamed Chinese chive was tested in normal rat liver (IAR20) and kidney (NRK) cells.

**Fig. 1:**
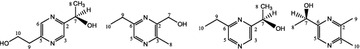
The structures of new pyrazines

**Fig. 2:**
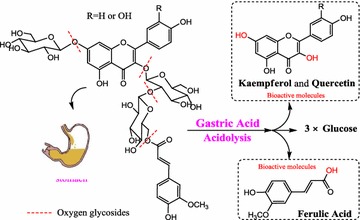
Acid hydrolysis process of the new flavonoids

**Conclusions:** The results showed that Chinese chive is toxic to liver and kidney cells when fresh, but is safe after heating. This could explain why it is traditional to eat cooked Chinese chive. A possible metabolic rule regarding pyrazines is postulated based on this data, and a human metabolic pathway is suggested for two of the novel compounds which have the highest amount of Chinese chive extracts.

References

1. China Flora Editorial Board. Sci Press, Beijing (in Chinese). 1980;14:249
2. Block E, Naganathan S. J Agr Food Chem. 1992;40:2418–30.
3. Block E, Putman D. J. Agr. Food Chem. 1992;40:2431–8.
4. Gao XL, Wu L. Chin Condi. 2004;6:18–20.

## 53 Screening of bromelain from different variants of *Ananas comosus*

### Aizi Nor Mazila Ramli, Nurul Hartini Ani, Siti Hajar Mohamad Nor

#### Faculty of Industrial Sciences & Technology, Universiti Malaysia Pahang, Lebuhraya Tun Razak, Gambang Kuantan, Pahang 26300, Malaysia

##### **Correspondence:** Aizi Nor Mazila Ramli - aizinor@ump.edu.my

*Journal of Chinese Medicine* 2018, **13(Suppl 1):**53

**Background:** Pineapple fruit, *Ananas comosus,* is widely known and distributed all over the countries. The consumption of pineapple fruit had been used for many purposes and interest. Many products produce from the pineapple source had been established in the market for the consumer use. The uses of it also dispersed in many areas that are mainly focus for the industrial and medicinal application. Bromelain is the enzyme extracted from the pineapple fruit or its stem. Bromelain is from the protease enzymes belonging to the *Bromeliaceae* family. The isolation of bromelain from the pineapple fruit and the study about its specialty had begun since 1894. This enzyme is rich with many benefits and help to treat in many problems. The content of this enzyme was found to be varied in the different type of pineapple cultivar. Therefore, this research study is aim to extract the fruit and stem bromelain from different type of cultivars in Malaysia. It was then followed by the enzymatic analysis of the enzyme followed by the protein quantification using SDS-PAGE analysis.

**Materials and methods:** The three different cultivars of *A. comosus*, Nanas Moris, Nanas Madu and Nanas Sarawak Kecil, are collected. The samples were prepared for bromelain extraction. The analysis of bromelain from fruit and stem were done using enzymatic assay and SDS PAGE.

**Results:** As a result, for fruit bromelain screening, Nanas Morris gave the highest enzymatic activity which is 0.8220 U/ml followed by Nanas Madu which is 0.7703 U/ml and Nanas Sarawak Kecil with the amount of 0.6925 U/ml. This is shown that, Nanas Morris have high amount of protein content in it and there are high amount of l-tyrosine released when the protease enzyme digest the casein substrate. On the other hand, for stem bromelain screening, the highest enzymatic activity was shown by Nanas Sarawak Kecil which is 0.672 U/ml followed by Nanas Morris and Nanas Madu with the value of 0.670 U/ml and 0.653 U/ml, respectively. The results were shown in Table 1 below.

**Table 1:** Enzymatic activity of fruit and stem brmelain from different cultivars of *A. comosus*

Cultivars	U/ml enzyme
Nanas Moris	0.8220
Nanas Madu	0.7703
Nanas Sarawak Kecil	0.6925
Stem bromelain	
Nanas Moris	0.672
Nanas Madu	0.670
Nanas Sarawak Kecil	0.653

It was reported that for the pineapple fruit in their crude state that is analyzed by using SDS-PAGE, The expected protein band of bromelain are observed within the range of 24–45 kDa [1]. In addition, the previous findings from the experiment conducted on crude extracts of the peel, pulp, leaves and stem of *A. comosus* showed the molecular weight ranging from 25 to 27 kDa for bromelain [2].

The result shows above is the formation of the protein band when undergo SDS PAGE analysis for all of the cultivar. The molecular weight for fruit bromelain is found approximately at 25 kDa based on the Fig. 1. However, other protein bands also visible in the SDS-PAGE because of the presence of other protein sample in the crude extract of the pineapple. This protein bands might indicate for the other proteases such as ananain and papain. Papain is one of the cysteine proteinase that is found in the pineapple fruit beside the bromelain [1, 2].

**Fig. 1:**
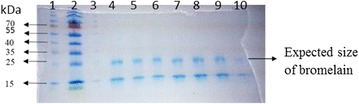
SDS PAGE of Crude Bromelain. Lane 1 and 2: protein marker, lane 4–6: stem bromelain, lane 7–10: fruit bromelain

**Conclusion:** The result indicated the presence of different concentration of bromelain from different variants of *A. comosus*. The research study can lead to the increase awareness of eating fresh pineapple among citizens.

**Acknowledgments:** This work was supported by UMP research grant under the Project No. RDU160321.

**References**

1. Sharma HK, Srivastava R, Shukla S. Isolation, purification and quantitative analysis of cysteine protease, bromelain from *Ananas Comosus* (pineapple). Int J Pharm Bio Sci. 2014;5(1):429–37.
2. Krishnan AV, Gokulakrishnan M. Extraction, purification of bromelain from pineapple and determination of its effect on bacteria causing periodontitis. Int J Pharm Sci Res. 2015;6(12):5284.

## 54 Study on the non-covalent weak interaction between natural stilbenoids and proteins

### Hui Cao^1^, Feng Tang^2^, Jianbo Xiao^1^, Xiaoqing Chen^2^

#### ^1^Institute of Chinese Medical Sciences, State Key Laboratory of Quality Research in Chinese Medicine, University of Macau, Taipa, Macau; ^2^College of Chemistry and Chemical Engineering, Central South University, 410083 Changsha, PR China

##### **Correspondence:** Jianbo Xiao, Xiaoqing Chen

*Journal of Chinese Medicine* 2018, **13(Suppl 1):**54

**Background:** On the molecular level, exploring the metabolism, distribution, and the interaction of natural bioactive substances of natural bioactivities, and confirming the effects of these small molecules on the metabolic diseases to develop natural medicines and nutritional food for treating metabolic diseases are the most important research areas in the field of biomedicine, pharmacy, and nutrition food sciences. This work studied the structure-affinity relationship of stilbenoids-HSA interaction and clarify the effects of stilbenoids-protein non-covalent weak interactions on the free radical scavenging activity and the stability of stilbenoids.

**Materials and methods:** Isorhapontigenin, oxyresveratrol, piceid, pterostilbene, pinostilbene, piceatannol and resveratrol were purchased from Tokyo Chemical Industry Co., Ltd (Shanghai, China). HPAC analysis was performed on a Waters HPLC with a 1525 binary HPLC pump, a 717plus auto sampler, and a model 2487 UV/VIS dual wavelength absorbance detector (MA, USA). The chromatography isolation was performed on a CHIRALPAK-HSA column (150 mm × 4 mm, I.D.,5 μm)(Chrom Tech Ltd., Congelton, Cheshire, UK). The DPPH and ABTS free radical scavenging activities of stilbenoids in the absence and presence of HSA were measured according to the literature with minor modifications [1,2]. The stability of stilbenoids in DMEM cell culture, human plasma, Milli Q water and HSA solution were detected [3,4].

**Results:** (1) The structure-affinity relationship shows that the methylation, glycosylation and methoxylation of resveratrol will reduce binding affinity with HSA. (2) The structure-free radical scavenging activity relationships of stilbenoids showed that the free radical scavenging activity of stilbenoids depends on their structure: the hydroxyl number on the ring A and B ring of stilbenoids significantly influences the free radical scavenging potential, more the hydroxyl group on stilbenoids, stronger free radical scavenging activity. The ortho-hydroxyl group substituted shows stronger free radical scavenging activity than the meta-hydroxyl group substituted. The methylation of the hydroxyl moiety on stilbenoids will weaken the free radical scavenging capacity; however, an additional methoxyl group on resveratrol will enhance the free radical scavenging ability. (3) The stability of stilbenoids in DMEM cell culture, human plasma, Milli Q water and HSA solution are compared. It was found that stilbenoids showed different stability in different solutions, and their stability is as follows: MilliQ water > HSA > human plasma > DMEM cell culture. The structure-stability relationship of stilbenoids in DMEM cell culture is determined as follows: (i) An additional hydroxyl group on ring B will reduce the stablity; (ii) The stability of resorcinol-type stilbenoids is higher than that of catechol-type stilbenoids; (iii) The methoxylation and glycosylation on of resveratrol improves the stability.

**Conclusions:** HSA masks the DPPH scavenging ability of stilbenoids, but it increases ABTS scavenging ability. The interaction between stilbenoids with plasma proteins is beneficial to enhance the stability.

**Acknowledgments:** This research was financially supported by the Start-up Research Grant from University of Macau (SRG2015-00061-ICMS-QRCM), and the opening fund of the State Key Laboratory of Quality Research in Chinese Medicine of University of Macau (No. SKL-QRCM-2014-2016).

**References**

1. Cao H, Chen XQ, Yamamoto K. Anti-cancer agent me. 2012;12:940–8.
2. Cao H, Xie YX, Chen XQ. Food Chem. 2015;186:106–12.
3. Cao H, Shi J, Jia XP, et al. Food Chem. 2016;202:383–8.
4. Tang F, Xie YX, Cao H, et al. Food Chem. 2017;219:321–8.

## 55 *Ganoderma* triterpene compounds ameliorates lipid metabolism based on the intestinal flora

### Bin Liu^1,2^

#### ^1^College of Food Science, Fujian Agriculture and Forestry University, Fuzhou, Fujian 350002, China; ^2^National Engineering Research Center of JUNCAO Technology, Fujian Agriculture and Forestry University, Fuzhou, Fujian 350002, China

##### **Correspondence:** Bin Liu - liubin618@hotmail.com

*Journal of Chinese Medicine* 2018, **13(Suppl 1):**55

**Background:** Among the many biologically active constituents of *Ganoderma*, the triterpenoids have been shown to have hypolipidemic effects, enhanced immunity, liver protection, anti-cancer and health.

**Materials and methods:** The effects of *Ganoderma* triterpene compounds (GP) on lipid metabolism in wistar rats induced by high-fat diet were studied in this study. The effects of GP on gut microbiota composition by performing a high-throughput sequencing-based analysis of bacterial 16S rRNA (V3–V4 region) in caecal feces and the liver tissue transcriptase sequencing analysis based on the Illumina high-throughput sequencing platform.

**Results:** The weight of Wistar rats fed with 8 weeks of high-fat diet was significantly higher than the control group (NFD) (p < 0.05), The weight gain of different doses of GP group (GP50, GP100, GP150) were significantly slower than model group (HFD) (P < 0.01). The animal serum levels of TG, TC, ALT, AST and FFA in high-dose test sample group (GP150) was significantly lower than those the HFD, and (HDL-C) was higher than that in HFD. Antioxidant enzymes in liver tissue of experimental rats were analyzed, the levels of MDA of silymarin group (Sym), GP50, GP100 group and GP150 group were significantly decreased respectively compared with the HFD (P < 0.01), GSH-PX and SOD in GP150 were significantly increased (P < 0.01). High-fat diet can lead to significant increased in the abundance of *Akkermansia*, *Lactobacillus, Nosocomiicoccus, Odoribacter, Oligella* and *Anaeroplasma*. On the other hand, decreased in the abundance of *Collinsella, Enterococcus, Desulfovibrio* and so on. The liver tissue of each experimental group was subjected to transcriptome sequencing analysis based on Illumina high-throughput sequencing platform, and the differentially expressed genes were analyzed by GO enrichment and KEGG enrichment. It was found that the differentially expressed genes were mainly enriched in PPAR signaling pathway, Biosynthesis of amino acids, Non-alcoholic fatty liver disease, Type II diabetes mellitus and AMPK signaling pathway compared with the model group. The differentially expressed genes closely related to lipid metabolism mainly include Gk2, MEF, Scd1, IRS1IRM, SREBP-1c, PKL, Jun, IRS1IRM, Tkfc, PKL, Gadd45, enoyl -CaA hydratase, Hadhsc, Cyp2c70, Acaa2, Cyp4a11, RT1-A, Ins1, HRSL3, Phlpb, Fadsd6, Muscpho, Xbp1 and pe-CoA compared with the model group, these differentially expressed genes play a vital role in the corresponding signaling pathways, and regulate the body’s lipid synthesis transport.

**Conclusions:** The *Ganoderma* triterpenes can effective in reducing the serum levels, the body weight, gut microbiota composition and genes expression, which closely related to lipid metabolism. The results provided the theoretical basis for the application of GP in functional food and drugs of the regulation of lipid metabolism.

## 56 Structural characteristics and prebiotic effects of lotus seed resistant starch

### Hongliang Zeng, Cancan Huang, Chuanjie Chen, Yi Zhang, Baodong Zheng

#### College of Food Science, Fujian Agriculture and Forestry University, China

##### **Correspondence:** Yi Zhang - zyifst@163.com; Baodong Zheng - zbdfst@163.com

*Journal of Chinese Medicine* 2018, **13(Suppl 1):**56

**Background:** Lotus seed resistant starch (LRS) is a type of retrograded starch that is commonly known as resistant starch type 3 (LRS3) [1, 2].

**Materials and methods:** The structural and crystalline properties of unpurified LRS (NP-LRS3), enzyme purified LRS after drying (GP-LRS3), and enzyme purified LRS (ZP-LRS3) were described. The in vitro effects of NP-LRS3 and GP-LRS3 on the proliferation of bifidobacteria were evaluated by assessing the changes in optical density (OD), pH values, short chain fatty acid (SCFA) production, and tolerance to gastrointestinal conditions compared with glucose (GLU) and high amylose maize starch (HAMS).

**Result:** The result showed that the molecular weights of NP-LRS3, GP-LRS3, and ZP-LRS3 were 0.102 × 10^6^, 0.014 × 10^6^, and 0.025 × 10^6^ Da, respectively. Compared with native starch and HAMS, LRS3 lacked the polarization cross and the irregularly shaped LRS3 granules had a rougher surface, B-type crystal structure, and greater level of molecular order. The FT-IR measurements indicated no differences in the chemical groups. Analysis by ^13^C NMR indicated an increased propensity for double helix formation and higher crystallinity in LRS3 than in the two other types of starch. Moreover, LRS3 was more effective than either glucose or HAMS in promoting the proliferation of bifidobacteria. LRS3 increased the number of bifidobacteria, achieved a brief lag phase, enhanced the bacterial tolerance to gastrointestinal conditions, and obtained a higher production of butyric acid.

**Conclusions:** The prebiotic effect of LRS3 was a result of a combination of multiple factors, including the surface microstructure, crystalline pattern, double helix structure and biological mechanisms. LRS3 as a prebiotic constituent could be applied widely in food industry.

**References**

1. Zhang Y, Zeng H, Wang Y, et al. Food Chem. 2014;155:311–8.
2. Zhang Y, Wang Y, Zheng B, et al. Food Funct. 2013;4:1609–16.

## 57 Effect of coconut shell based activated carbon containing peppermint oil and its major component on growth of *Aspergillus niger* and *Aspergillus flavus*

### Siriporn Chaemsanit^1^, Narumol Matan^1^, Nirundorn Matan^2^

#### ^1^Food Technology, School of Agricultural Technology, Walailak University, Nakhon Si Thammarat, Thailand; ^2^Walailak University, Nakhon Si Thammarat, Thailand

##### **Correspondence:** Narumol Matan - nnarumol@wu.ac.th

*Journal of Chinese Medicine* 2018, **13(Suppl 1):**57

**Background:** The used of essential oil as the natural antifungal in food and agricultural products have been increasing. Essential oil vapor, have been proved to be more effective than liquid phase [1]. The antifungal activity of peppermint oil vapor has been confirmed from many studied that has potential to apply in foods due to its pleasant sensory qualities and it traditionally used against a number of diseases [2]. However, the application of essential oil vapor has some drawback about the degradation and unstable concentration during exposure. To overcome this problem, activated carbon (AC) is needed to act as an essential oil carrier. This experimental aim is to prove that AC could release peppermint oil and menthol (as its major component) in vapor phase and the released vapor can cause the antifungal activity against postharvest pathogenic fungi isolated from brown rice which are *Aspergillus niger*, *Aspergillus flavus*.

**Materials and methods:** Peppermint oil was analyzed for its main component using GC/MS. The *A. flavus* and *A. niger* were isolated from brown rice. The modified disc volatilization was used to determine the antifungal activity. The 20 μL of fungal suspensions (10^7^ CFU mL^−1^) was added on MEA media in the air tight glass jar (1L) before the peppermint oil (100–1000 μL L^−1^air) and menthol (40–400 μL L^−1^air) treated activated carbon was being hung in the middle of the jar. Control was done in the same way but without essential oil. After 5 days, mycelia growth was measured. The lowest concentration that produced no visible colony was reported to be the minimum inhibitory concentration (MIC).

**Results:** The result shows that AC has potential to release both peppermint oil and menthol vapor against *A. niger* and *A. flavus*. The antifungal activity could be correlated to the presence of peppermint oil and menthol as its major components (38.6%) since the increasing of peppermint oil and menthol concentration have effect on the growth of both fungal species. The MIC of peppermint oil adsorbed activated carbon on *A. niger* and *A. flavus* was shown in Table 1.

**Table 1:** Minimum inhibitory concentration of *A. flavus* and *A. niger*

	Menthol		Peppermint	
	*A. niger*	*A. flavus*	*A. niger*	*A. flavus*
MIC (uL L^−1^ air)	500	800	250	700

**Conclusions:** This experiment confirmed that both peppermint oil and menthol could release from the activated carbon at room temperature and those compounds still have antifungal ability to inhibit *A. niger* and *A. flavus* growth for more than 5 days. Further experiment should be performed in order to investigate the effect of the minor component and the synergic effect between each component. However, this experiment prove that peppermint oil gives higher antifungal activity than the purified major component. This gives a lot of benefit for the local application since the price of essential oil is lower than the purified component.

**References**

1. Songsamoe S, Matan N, Matan N. Effect of UV-C radiation and vapor released from a water hyacinth root absorbent containing bergamot oil to control mold on storage of brown rice. J Food Sci Tech. 2016;53:1445–53.
2. Chamsai P, Tapnarong G, Junlapak D, Matan N. Development of hand sanitizing spray using peppermint oil. As J Food Ag-Ind. 2010;3:178–83.

## 58 Targeting HSF1 leads to anti-tumor effect in human epithelial ovarian cancer

### Ting Liu^1^, You-Hui Yang^1^, Yi-Fei Chen^2^

#### ^**1**^Guizhou Provincial Key Laboratory of Pharmaceutics, Guizhou Medical University, Beijing Road 9, Guiyang, 550004, Guizhou, People’s Republic of China; ^2^The International Peace Maternity and Child Health Hospital, School of medicine, Shanghai Jiaotong University, Hengshan Road 910, 200030, Shanghai, People’s Republic of China

##### **Correspondence:** Yi-Fei Chen

*Journal of Chinese Medicine* 2018, **13(Suppl 1):**58

**Background:** Late diagnosis and lack of specific therapy target contribute to the low survival rate of human epithelial ovarian cancer (EOC), the most lethal gynecologic malignancy [1]. Therefore, screening diagnostic markers and identifying therapy targets are urgently required. Heat shock factor 1 (HSF1) has been demonstrated to be over-expressed in certain malignancies and to be involved in tumor initiation, development, transformation and metastasis. It is believed that HSF1 is a promising candidate for anti-tumor therapy [2, 3]. However, its expression pattern and function in ovarian cancer is far from illumined.

**Methods:** We examined the HSF1 expression in human epithelial ovarian cancer tissues, and evaluated its carcinogenesis promoting activity in a xenograft tumor model.

**Results:** In normal ovarian tissues, HSF1 was barely detected, whereas, high expression of HSF1 was found in malignant EOC tissues. Suppressed proliferative activity and intensified apoptosis were observed in HSF1 knocked-down SKOV3 cells. In nude mouse xenografts, down-regulation of HSF1 was found to cause reduced carinogenesis, indicating the anti-tumor effect induced by modulation of HSF1 against EOC.

**Conclusions:** Our findings suggest that HSF1 may be considered as a potential candidate for a diagnostic marker of human EOC, and that modulation of HSF1 could be a promising therapy strategy against human EOC.

**Acknowledgements:** Supported by National Natural Science Foundation of China (No. 81401216 and 81603189)

**References**

1. Coward JI, Middleton K, Murphy F. Int J Womens Health. 2015;7: 89–203.
2. Vihervaara A, Sistonen L. J Cell Sci. 2014;127:261–6.
3. Chen Y, Chen J, Loo A, et al. Oncotarget. 2013;4:816–29.

## 59 Protective effects of cyanidin-3-*O*-β-glucoside on carbon tetrachloride-induced liver fibrosis and the underlying mechanism in mice

### Xinwei Jiang^1,2^, Tianran Shen^2^, Xilan Tang^2^, Wenqi Yang^2^, Yan Yang^2^, Honghui Guo^3^, Wenhua Ling^2,4^

#### ^1^Department of Food Science and Engineering, Institute of Science and Technology, Jinan University, Guangzhou 510632, China; ^2^Department of Nutrition, School of Public Health, Sun Yat-Sen University, Guangzhou 510080, China; ^3^Department of Nutrition, Henry Fok School of Food Science and Engineering, Shaoguan University, Shaoguan 512005, China; ^4^Guangdong Provincial Key Laboratory of Food, Nutrition and Health, Guangzhou 510080, China

##### **Correspondence:** Honghui Guo - guohh1999@hotmail.com; Wenhua Ling - lingwh@mail.sysu.edu.cn

*Journal of Chinese Medicine* 2018, **13(Suppl 1):**59

**Background:** Previous studies indicated that Cyanidin-3-O-β-glucoside (C3G) as a classical anthocyanin exerted liver protective effect, but the effect on liver fibrosis was not fully explored [1]. In addition, the bioavailability of anthocyanins are quite low, thus the mechanism of anti-fibrosis effect of C3G still need to be systematically investigated [2].

**Materials and methods:** In the present study, carbon tetrachloride (CCl_4_)-treated liver fibrosis animal model and primary hepatic stellate cells (HSCs) were adopted to explore the restraining effect of C3G and its metabolite protocatechuic acid (PCA) on liver fibrosis and the activation of HSCs.

**Results:** Our data demonstrated that the treatment of C3G on CCl_4_-treated mice model inhibited liver fibrosis and HSCs activation. Both C3G and PCA reserved the lipid droplet as well as retinol in primary HSCs in vitro. C3G and PCA separately inhibited the mRNA expression of α-smooth muscle actin and collagen I, but elevated the level of matrix metalloproteinase-2 and liver X receptors. Only PCA suppressed the levels of tumor necrosis factor alpha (TNF-α) and interleukin-6 (IL-6) secreted from HSCs significantly. In addition, C3G and PCA inhibited the proliferation and migration of HSCs.

**Conclusions:** In conclusion, daily intake of Cy-3-G could prevent liver fibrosis progression in mice induced by CCl_4_ through inhibiting HSC activation. PCA mainly explained the inhibiting effect, which provides a basis for clinical practice of liver fibrosis prevention.

**Funding:** This work was funded by grants from the National Basic Research Program (973 Program, 2012CB517506) and the National Nature Science Foundation (81372994).

**Acknowledgments:** Lili Yang, Dan Li and Dongliang Wang are acknowledged for supporting the research work with suggestions.

**References**

1. Tang X, Shen T, Jiang X et al. Purified anthocyanins from bilberry and black currant attenuate hepatic mitochondrial dysfunction and steatohepatitis in mice with methionine and choline deficiency. J Agric Food Chem. 2015;63(2): 552–61.
2. Wang DL, Xia M, Yan X, et al. Gut Microbiota Metabolism of Anthocyanin Promotes Reverse Cholesterol Transport in Mice Via Repressing miRNA-10b. Circ Res. 2012;111(8): 967-+.

## 60 Contemplation on application of phytochemicals from food materials

### Xuqiao Feng, Chenghui Zhang, Yuxia Qi

#### College of Food Science and Engineering, Bohai University, Jinzhou Liaoning 121013, P.R.China

##### **Correspondence:** Xuqiao Feng

*Journal of Chinese Medicine* 2018, **13(Suppl 1):**60

**Background:** Researches carried out all over the world in recent decades have demonstrated that some phytochemicals extracted from food materials such as fruits and vegetables by modern technologies exert some health-keeping functions.

**Results:** However, practical application of these compounds is still impossible in most cases as crucial data like appropriate dosage have not yet precisely determined for human beings to benefit from them. Nevertheless, food technologists would not like to wait any longer to take the advantage of these compounds as food materials or additives for producing daily food with some desired effects.

**Conclusions:** Feasible suggestions regarding the present situation on the application of these compounds have been posed based on detail analysis and discussion on status quo of the phytochemical research results.

## 61 Flavonoids may weaken the toxicity of ZnO NPs to Caco-2 cells

### Yi Cao, Rui Geng, Liangliang Liu, Yixi Xie

#### College of Chemistry,Xiangtan University, 411105, Xiangtan, China

##### **Correspondence:** Yixi Xie - xieyixige@xtu.edu.cn

*Journal of Chinese Medicine* 2018, **13(Suppl 1):**61

**Background:** The interactions between phytochemicals and nanoparticles (NPs) may affect the toxicity of NPs [1]. Flavonoids are the most vital phytochemicals in diets and are of great general interest due to their diverse bioactivity [2]. Herein, baicalein (Ba) and its glycoside baicalin (Bn), were selected as models for phytochemicals and their interactions with ZnO NPs as well as the influences on the toxicity of ZnO NPs to Caco-2 cells were studied.

**Materials and methods:** The human colon epithelial Caco-2 cells (ATCC, HTB-37) and the human liver cells HepG2 (ATCC, HB-8065) were used for cytotoxicity assay. The interactions between Ba or Bn and ZnO NPs were indicated by the changes of hydrodynamic sizes, zeta potential and UV–Vis spectra. The cytotoxicity of ZnO NPs with or without the presence of Ba or Bn was investigated by CCK-8 assay, neutral red uptake and acridine organe. The uptake of ZnO NPs into Caco-2 cells was estimated by the increase of intracellular Zn ions. Oxidative stress was indicated by the measurement of superoxide using dihydroethidium (DHE), whereas inflammation was indicated by the measurement of release of interleukin 1beta (IL-1beta) and IL-6.

**Results:** Compared with Bn, Ba were more effective at changing the hydrodynamic sizes, zeta potential and UV–Vis spectra of ZnO NPs. With the presence of Ba, the cytotoxicity of ZnO NPs to Caco-2 cells was modestly decreased, whereas the cyto-protective effect was not observed in HepG2 cells (see Fig. 1). Intracellular superoxide or release of inflammatory cytokines were not affected by the exposure to ZnO NPs with or without the presence of Ba or Bn (p > 0.05). Exposure of Caco-2 cells to ZnO NPs significantly increased intracellular Zn ions (p < 0.01), which was modestly decreased by the presence of Ba but not Bn (p > 0.05).

**Fig. 1:**
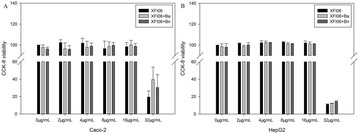
The cytotoxicity of ZnO NPs (XF106) with or without the presence of Ba or Bn

**Conclusions:** The presence of Ba may modestly protected Caco-2 cells from the exposure to ZnO NPs by stabilizing the NPs and accumulation of intracellular Zn ions.

**Acknowledgements:** The authors thank for the financial support from Natural Science Foundation of Hunan Province (2017JJ3303) and Research Foundation of Education Bureau of Hunan Province (17C1521).

**References**

1. Cao Y, Li J, Liu F, Li X, Jiang Q, Cheng S, Gu Y. Environ Toxicol Pharmacol. 2016;46:206–10
2. Xiao J. Crit Rev Food Sci Nutr. 2017;57:1874–905

## 62 Modification of Rupestonic acid for development of drug candidates against influenza viruses

### Jiangyu Zhao^1^, Abdullah Yusuf^1,2^, Gen Li^1^, Haji Akber Aisa^1^, Guozheng Huang^1^

#### ^1^Xinjiang Technical Institute of Physics and Chemistry, Chinese Academy of Sciences, South Beijing Road 40-1, Urumqi, 830011, P. R. China; ^2^University of Chinese Academy of Sciences, Yuquan Rd 19 A, Beijing, 100049, P.R.China

##### **Correspondence:** Haji Akber Aisa; Guozheng Huang

*Journal of Chinese Medicine* 2018, **13(Suppl 1):**62

**Background:** Influenza is a highly contagious disease spreading around the world in seasonal epidemics, causing death up to half million annually. Although influenza vaccine and anti-influenza drugs are available, multi-drug-resistant variant viruses will render some of those drugs ineffective, and thus discovery of new anti-influenza agents is of great emergency. *Artemisia rupestris* L. (Compositae) is widely distributed in the Xinjiang, China and central Asia countries. Its multiple biological function including anti-influenza intrigued continuous phytochemical investigations. In 1988 Xu et al. isolated rupestonic acid, a guaiane type sesquiterpene from *A. rupestris* for the first time [1], although its structure was erroneously assigned as *1S, 7S, 10S*-**1** (Fig. 1). Later they proposed its structure as *1S, 7S, 10R*-**1** [2], but the absolute configuration was only recently confirmed as *1S, 7R, 10S* by us applying X-ray analysis. Our group also isolated 13 new sesquiterpene alkaloids from the flowers and the leaves of *A. rupestris* [3, 4].

**Fig. 1:**
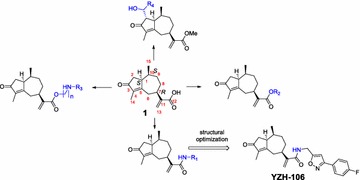
Chemical modification of rupestonic acid

**Results:** We systemically modified carboxyl group and cyclopentenone ring of rupestonic acid by condensation reaction (Fig. 1) [5–7]. Nearly 250 new compounds were synthesized and evaluated with regard their activities inhibiting influenza viruses A and B. Among them, several compounds especially compound, especially compound **YZH-106** [8] (Fig. 1), exhibited pronounced activities and deserved further investigation.

**Conclusions:** Structural-activity relationship of rupestonic acid derivatives has been systematically investigated. Several anti-influenza virus lead compounds have been discovered. Among them, compound **YZH-106** exhibited activity against influenza viruses as good as the positive drug (oseltamivir). Further investigation on the structural modification of rupestonic acid is ongoing and would contribute to the discovery of novel anti-influenza drug candidates from natural products.

**Acknowledgments:** This work was financially supported by National Natural Science Foundation of China (No. 81402808).

**References**

1. Xu GS, Chen XY, Yu DQ. Rupestonic acid, A new sesquiterpene from Artemisia Rupestris *L*. Acta Pharma Sinica. 1988; 23:122–5.
2. Xu GS, Zhao W, Wu D, Yu DQ, He CH, Yang JJ, Sun F. The structure and absolute configuration of isorupestonic acid from Artemisia Rupestris *L.* Acta Pharma Sinica. 1991; 26: 505–9.
3. Su Z, Wu HK, He F, Slukhan U, Aisa HA. New Guaipyridine Sesquiterpene Alkaloids from Artemisia rupestris *L*. Helv Chim Acta. 2010;93:33–8.
4. He F, Nugroho AE, Wong CP, Hirasawa Y, Shirota O, Morita H, Aisa HA. Rupestines F—M, New Guaipyridine Sesquiterpene Alkaloids from Artemisia rupestris. Chem Pharm Bull. 2012;60:213-218.
5. Zhao J, Aisa HA. Synthesis and anti-influenza activity of aminoalkyl rupestonates. Bioorg Med Chem Lett. 2012;22:2321–5.
6. Li G, Zhao JY, Niu C, Nie LF, Dong CZ, Aisa HA. Structure–activity relationship studies of 1-(10-hydroxyalkyl)rupestonic acid methyl esters against influenza viruses. Bioorg Med Chem Lett. 2017;27:1484–7.
7. He YW, Dong CZ, Zhao JY, Ma LL, Li YH, Aisa HA. 1,2,3-Triazole-containing derivatives of rupestonic acid: Click-chemical synthesis and antiviral activities against influenza viruses. Eur J Med Chem. 2014;76:245–255.
8. Ma LL, Wang HQ, Wu P, Hu J, Yin JQ, Wu S, Ge M, Sun WF, Zhao JY, Aisa HA, Li YH, Jiang JD. Rupestonic acid derivative YZH-106 suppresses influenza virus replication by activation of heme oxygenase-1-mediated interferon response. Free Radical Bio Med 2016;96:347–61.

## 63 Evaluation of the therapeutic potential of gyepnoside for retinal degeneration

### Reem Hasaballah A. Alhasani, Xinzhi Zhou, Jim Reilly and Xinhua Shu

#### Department of Life Sciences,Glasgow Caledonian University, Cowcaddens Road, Glasgow G4 0BA, Scotland, UK

##### **Correspondence:** Xinhua Shu - Xinhua.Shu@gcu.ac.uk

*Journal of Chinese Medicine* 2018, **13(Suppl 1):**63

**Background:** Inherited retinal degeneration (IRD) is a common cause of blindness in humans, characterized by the death of photoreceptor cells. Although the molecular disease mechanisms of IRD are not fully understood, oxidative stress and inflammation are believed to play a major role in the photoreceptor cell death. There is no effective therapy for patients with IRD, and development of new therapeutic strategies is urgently needed. The zebrafish has been increasingly used as a model to study a variety of human diseases, including visual disorders. We have characterized a zebrafish mutant line that carries a nonsense mutation in the *rpgrip1* gene. The *rpgrip1* mutant Zebrafish showed an absence of rod photoreceptor segments and early retinal degeneration. Gyepnoside (Gyp) is the predominant component of *Gynostemma pentaphyllum*, a traditional Chinese medicine. Gyp has been shown to have antioxidant and anti-inflammatory properties in non-retinal cells and organs. In this study we investigated the protective role of Gyp in retinal degeneration.

**Materials and methods:** *Rpgrip1* mutant zebrafish were treated with Gyp from 6 h post fertilization (hpf) to one-month old or from 2- to 6-months old. After the treatment, eye samples were collected and subjected to histological, histochemical and biochemical assays.

**Results:** To evaluate whether Gyp can protect against rod photoreceptor cell death, *rpgrip1* mutant zebrafish embryos were treated with Gyp from 6hpf till one-month old. Gyp-treated zebrafish showed significantly delayed rod photoreceptor cell death. To evaluate whether Gyp can reduce cone cell death, 2-months old *rpgrip1* mutant zebrafish were treated with Gyp for 4 months and exhibited significantly decreased death of double cone cells. *Rpgrip1* mutant zebrafish showed significantly decreased antioxidant capacity and increased inflammation and lipid peroxidation when compared to the wildtype siblings. Gyp-treated zebrafish demonstrated significantly increased expression of antioxidant genes (SOD, catalase, NQ1) and decreased inflammation genes (IL-1β, IL-6, TNF-α) when compared to untreated controls. The level of glutathione and the activities of catalase and superoxide dismutase were also significantly increased whereas the level of malondialdehyde was significantly decreased in Gyp-treated zebrafish when compared to untreated controls. The protection against photoreceptor cell death by Gyp is likely to be through NRF2-ARE and NF-kB signalling pathways. We found Gyp-treated *rpgrip1* mutant zebrafish had a high level of NRF2 and a low level of NF-kB when compared to untreated controls.

**Conclusion:** Gyp has the capacity to protect against oxidative damage and inflammation and offers therapeutic potential for patients with retinal degeneration.

## 64 Comprehensive characterization and identification of antioxidants in *Folium Artemisiae Argyi* using high resolution tandem mass spectrometry

### Binsong Han, Zhongquan Xin, Shasha Ma, Wenbin Liu, Bingyang Zhang, Lu Ran, Lunzhao Yi, Dabing Ren

#### Yunnan Food Safety Research Institute, Kunming University of Science and Technology, Kunming, 650500, P.R. China

##### **Correspondence:** Dabing Ren - rendabing425@163.com

*Journal of Chinese Medicine* 2018, **13(Suppl 1):**64

**Background:** *Folium Artemisia Argyi* (FAA) is the dried leaves of *Artemisia argyi* Levl. et Vant., which is widely distributed in China, Korea, Mongolia, and Japan [1]. As a traditional Chinese medicine and food supplement, FAA exhibits hemostatic, analgesic and antipruritic activities for treatment of various ailments, such as hemorrhage, pain, and skin itch; it is also consumed as a food ingredient because of its delicious flavor and distinctive smell [2, 3]. To date, most of the published works are focused on the volatile oil due to its high content in FAA [4, 5]. However, FAA contains many other nonvolatile compounds, such as phenolic acids and flavonoids. Unfortunately, only few systematic studies were performed to investigate the nonvolatile components of FAA. To study the nonvolatile phytochemical compounds in FAA, we developed a rapid and efficient UHPLC–high-resolution quadrupole Orbitrap mass spectrometry (Q–Orbitrap–MS) method in the current work.

**Materials and methods:** FAA powder (50.00 g) was extracted twice with 70% methanol under ultrasound, and extracts were successively extracted with *n*-hexane, ethyl acetate and *n*-butanol to yield four fractions, namely, n-hexane fraction (*n*-HexF), ethyl acetate fraction (EAF), *n*-butanol fraction (*n*BuF), and water fraction (WF). The total phenolic content (TPC), total flavonoid content (TFC) of all fractions were measured and compared, and antioxidant activities of all fractions were assessed by DPPH free radical, ABTS free radical, superoxide anion scavenging assay and Ferric-reducing antioxidant power (FRAP) assay [6–10]. DPPH–UHPLC–MS experiments were performed to screen the antioxidant constituents in each fraction [11].

**Results:** As shown in Fig. 1 EAF shows the highest TPC and TFC and highest antioxidant capability with regard to DPPH, ABTS, superoxide anion free radical scavenging ability, and ferric reducing antioxidant power. In addition, the potential antioxidant components were screened by DPPH–UHPLC–MS experiments and subsequently characterized by using high-resolution tandem mass spectrometry (shown in Fig. 2).

**Fig. 1:**
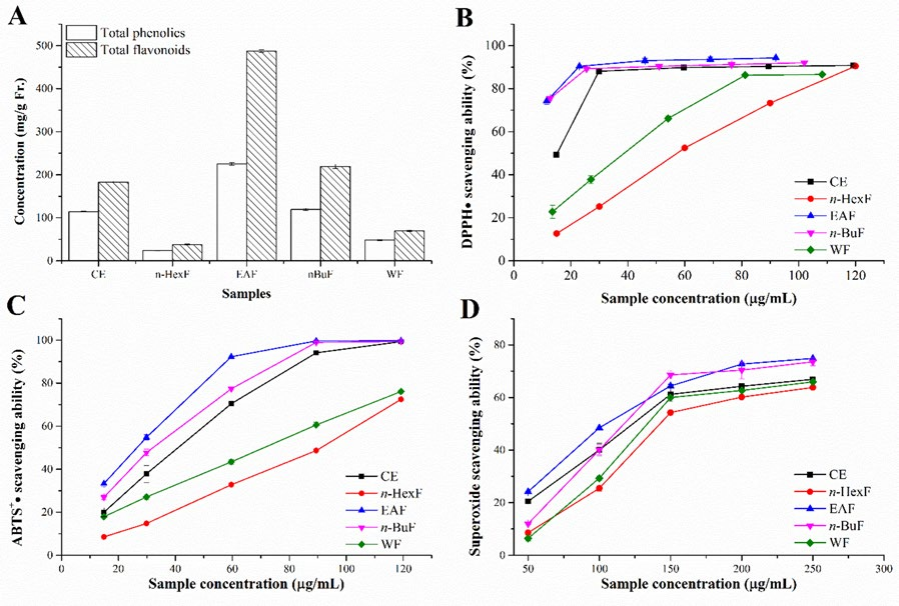
Content of total phenolics and total flavonoids (**A**), DPPH• scavenging ability (**B**), ABTS^+^• scavenging ability (**C**), Superoxide anion scavenging ability (**D**) of all fractions

**Fig. 2:**
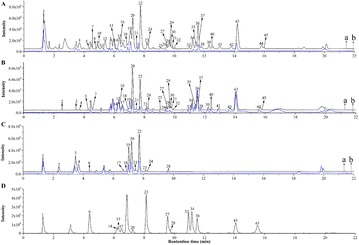
Base peak chromatogram (BPC) (a) and DPPH•-BPC (b) of crude extract (**A**); BPC (a) and DPPH•-BPC (b) of ethyl acetate fraction (**B**); BPC (a) and DPPH•-BPC (b) of *n*-butanol fraction (**C**) of FAA, BPC of 15 standards (**D**) obtained by UHPLC-Q-Orbitrap-MS^2^

**Conclusion:** This work further demonstrated that UHPLC–HRMS is a powerful tool for characterization and identification of compounds in complex samples, such as FAA. In addition, this work revealed the potential of FAA as an inexpensive resource of natural antioxidants.

**Acknowledgements:** This work was supported financially by National Nature Foundation Committee of P.R. China (No. 21465016).

**References**

1. Abad MJ, Bedoya LM, Apaza L, Bermejo P. The artemisia L. Genus: a review of bioactive essential oils. Molecules. 2012;17(3):2542–66. PubMed PMID: 22388966.
2. Zhao QC, Kiyohara H, Yamada H. Anti-complementary neutral polysaccharides from leaves of Artemisia princeps. Phytochemistry. 1994;35(1):73–7.
3. Techen N, Parveen I, Pan Z, Khan IA. DNA barcoding of medicinal plant material for identification. Current opinion in biotechnology. 2014;25:103–10. PubMed PMID: 24484887.
4. Wenqiang G, Shufen L, Ruixiang Y, Yanfeng H. Comparison of composition and antifungal activity of Artemisia argyi Levl. et Vant inflorescence essential oil extracted by hydrodistillation and supercritical carbon dioxide. Natural product research. 2006;20(11):992–8. PubMed PMID: 17032625.
5. Li N, Yu M, Zhang X. Separation and Identification of Volatile Constituents in Artemisia argyi Flowers by GC–MS with SPME and Steam Distillation. J Chromatogr Sc. 2008;46(5):401.
6. Stratil P, Klejdus B, Kubán V. Determination of total content of phenolic compounds and their antioxidant activity in vegetables–evaluation of spectrophotometric methods. J Agric Food Chem. 2006;54(3):607.
7. Hu C, Yuan YV, Kitts DD. Antioxidant activities of the flaxseed lignan secoisolariciresinol diglucoside, its aglycone secoisolariciresinol and the mammalian lignans enterodiol and enterolactone in vitro. Food and chemical toxicology: an international journal published for the British Industrial Biological Research Association. 2007;45(11):2219–27. PubMed PMID: 17624649.
8. Yang L, Gong J, Wang F, Zhang Y, Wang Y, Hao X, et al. Synthesis and antioxidant evaluation of novel silybin analogues. J Enzyme Inhib Med Chem. 2008;21(4):399–404.
9. Hosu A, Cristea VM, Cimpoiu C. Analysis of total phenolic, flavonoids, anthocyanins and tannins content in Romanian red wines: prediction of antioxidant activities and classification of wines using artificial neural networks. Food Chem. 2014;150:113–8. PubMed PMID: 24360427.
10. Shafiee S, Minaei S, Moghaddam-Charkari N, Barzegar M. Honey characterization using computer vision system and artificial neural networks. Food chemistry. 2014;159:143–50. PubMed PMID: 24767037.
11. Hu X, Chen L, Shi S, Cai P, Liang X, Zhang S. Antioxidant capacity and phenolic compounds of Lonicerae macranthoides by HPLC–DAD-QTOF-MS/MS. Journal of pharmaceutical and biomedical analysis. 2016;124:254–60. PubMed PMID: WOS:000374202000028.

## 65 Overexpression of the global regulator DegU controls the biosynthesis of bacillomycin D-like lipopeptides in *Bacillus amyloliquefaciens*

### Eun La Kim^1^, Soo-Kyung Kim^1^, Sen Liu^1^, Jongki Hong^2^, Joon-Hee Lee^1^, Jee H. Jung^1^

#### ^1^College of Pharmacy, Pusan National University, Busan, Republic of Korea, 609-735; ^2^College of Pharmacy, Kyoung Hee University, Seoul, Republic of Korea, 130-701

##### **Correspondence:** Jee H. Jung - jhjung@pusan.ac.kr

*Journal of Chinese Medicine* 2018, **13(Suppl 1):**65

**Background:** In a continuing search for novel and biologically active natural products from microorganisms associated with marine invertebrate, an endobiotic bacterium J05B-2-F′-4 was isolated from the tissue of the sponge *Suberites japonicus* and identified as *Bacillus amyloliquefaciens*. The crude extract of the bacterial culture exhibited antibacterial activity against methicillin-resistant *Staphylococcus aureus,* MRSA 3089. Bioassay-guided fractionation led to isolation of new nonribosomal cyclic lipopeptides **1**–**4**, which were found to exist as two inseparable conformers in DMSO-*d*_*6*_. It was revealed that sponge-derived strain, *B. amyloquefaciens* J05B-2-F′-4, biosynthesize new nonribosomal cyclic lipopeptides, which is similar to bacillomycin D [1]. In order to improve bacillomycin D-like peptides production, the strain *Bacillus amyloliquefaciens* J05B-2-F′-4 was engineered by the global two-component response regulator DegU [2, 3].

**Materials and methods:** The global regulator DegU gene found in the genome of *B. amyloquefaciens* J05B-2-F′-4, was cloned into pHY300PLK vector. The plasmid was introduced into the naturally competent *B. amyloquefaciens* J05B-2-F′-4 to create strains DBAJ05B-2-F′-4. The lipopeptides were purified from extracts of *B. amyloquefaciens* J05B-2-F′-4 fermentation.

**Results:** Compared to those of wild strain, *B*. *amyloquefaciens* J05B-2-F′-4, the bacillomycin D-like peptides production of the strain DBAJ05B-2-F′-4 was reduced by the overexpression of DegU. This study led to additional isolation of new nonribosomal cyclic lipopeptides (**5**).

**Conclusions:** The overexpression of DegU in *B. amyloliquefaciens* J05B-2-F′-4 down-regulated expression of their gene cluster encoding bacillomycin D-like peptides.

**References**

1. Peypoux F, Pommier M, Das BC, Besson F, Delcambe L, Michel G. Structures of bacillomycin D and bacillomycin L peptidolipid antibiotics from *Bacillus subtilis*. *J Antibiotics*, 1984;37:1600–4.
2. Koumoutsi A, Chen X, Vater J, Borriss R. DegU and YczE Positively Regulate the Synthesis of Bacillomycin D by *Bacillus amyloliquefaciens* Strain FZB42. *Appl Environ Microbiol*. 2007;73: 6953–64.
3. Xu Z, Zhang R, Wang D, Qiu M, Feng H, Zhang N, Shen Q. Enhanced control of cucumber wilt disease by *Bacillus amyloliquefaciens* SQR9 by altering the regulation of its DegU phosphorylation. *Appl Environ Microbiol*. 2014; 80: 2941–50.

## 66 Photooxidation degradation of phytochemicals in food: A review

### Baiyi Lu, Yajing Zhao

#### National Engineering Laboratory of Intelligent Food Technology and Equipment, Key Laboratory for Agro-Products Postharvest Handling of Ministry of Agriculture, Key Laboratory for Agro-Products Nutritional Evaluation of Ministry of Agriculture,Zhejiang Key Laboratory for Agro-Food Processing, Fuli Institute of Food Science, College of Biosystems Engineering and Food Science, Zhejiang University, Hangzhou 310058, China

##### **Correspondence:** Baiyi Lu - bylu@zju.edu.cn

*Journal of Chinese Medicine* 2018, **13(Suppl 1):**66

**Background:** Phytochemicals are widely present in food and have been confirmed to be bioactive contributing to human health. However, some of them are sensitive to light due to their structures and may suffer from photooxidation degradation especially when sensitizers such as riboflavin exist. Phytochemicals under light exposure may lose their bioactivities, resulting in sensory quality change and nutrition loss of food, even producing toxic components.

**Results:** The photooxidation degradations react through three different ways: (1) by directly absorbing luminous energy (type I photooxidation); (2) with triplet-excited state sensitizers through electron transfer or proton transfer (type I photooxidation); (3) with singlet oxygen produced by O_2_. Which pathway predominates in the food is determined by the substrate and reaction conditions, including pH, solvent, concentrations and the availability of molecular oxygen. Type I photoreaction often occurs under low oxygen pressure conditions, while type II prefers a high oxygen atmosphere. On the basis of the above mechanisms, in addition to physical control, effective quenchers can be used to quench triple-excited state sensitizers or singlet oxygen against photooxidation.

**Conclusions:** The deterioration of food due to the light-induced degradation of phytochemicals has become a common phenomenon in daily life. Light exposure is an important factor that cannot be ignored. The pathways of phytochemicals photooxidation are determined by the environment and structure characteristics. Considering the protection effectivity of quenchers and adverse quality change of food due to photooxidation, we need to choose the most suitable quencher through more experiments.

**Keywords**: Phytochemicals; photooxidation; reaction pathway; control measures

## 67 Discovery of oleanolic acid and ursolic acid derivatives as potent and highly selective inhibitors against human carboxylesterase 1

### LiWei Zou^1,2^, Ping Wang^1,2^, Dandan Wang^1,2^, Guangbo Ge^2^, Ling Yang^1^

#### ^1^Institute of Interdisciplinary Integrative Medicine Research, Shanghai University of Traditional Chinese Medicine, Shanghai, China; ^2^Dalian Institute of Chemical Physics, Chinese Academy of Sciences, Dalian, China

##### **Correspondence:** Guangbo Ge - geguangbo@dicp.ac.cn

*Journal of Chinese Medicine* 2018, **13(Suppl 1):**67

**Background:** Human carboxylesterase 1 (hCE1), one of the most important serine hydrolases distributed in liver and adipocytes, plays key roles in endobiotic homeostasis and xenobiotic metabolism [1]. Recent studies have revealed that the activities of hCE1 are markedly elevated in obese individuals and patients with type 2 diabetes, and the treatment of hCE1 inhibitors displayed multiple beneficial effects in both lipid and glucose homeostasis in genetic and diet-induced mouse models of obesity, insulin resistance and type 2 diabetes.

**Results and conclusions:** In summary, a series of natural triterpenoids were collected and their inhibitory effects against human carboxylesterases were assayed. Two natural pentacyclic triterpenoids including OA and UA were found to display strong inhibitory effects on hCE1 (Fig. 1). Our results demonstrated that the carbonyl group at the C-28 site is essential for hCE1 inhibition, any modifications of OA or UA at this site including esters, amides and alcohols are unbeneficial for hCE1 inhibition. In contrast, the structural modifications on OA and UA at other sites, such as converting the C-3 hydroxy group to 3-O-β-carboxypropionyl (compounds 20 and 22), led to a dramatically increase of the inhibitory effects against hCE1 (IC_50_, 17 and 12 nM respectively) and the selectivity over hCE2 (3296- and 6919-fold against hCE2 respectively). Furthermore, both inhibition kinetic analyses and docking simulations demonstrated that compounds 20 and 22 were potent competitive inhibitors against hCE1-mediated DME hydrolysis. All these findings are very helpful for medical chemists to design and develop potent and highly selective hCE1 inhibitors for biomedical applications.

**Fig. 1:**
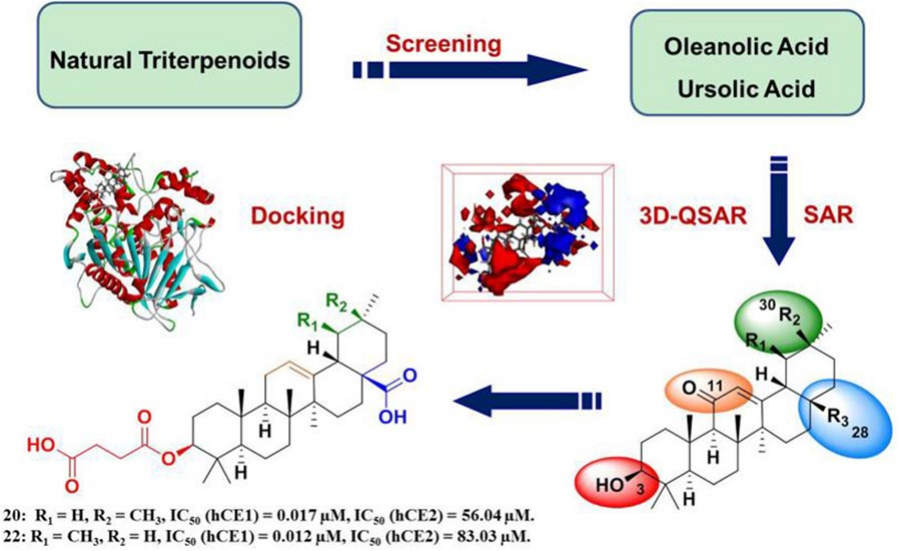
Graphical abstract

**Reference**

1. Redinbo MR, Potter PM. Drug Discov Today. 2005;10:313–25.

## 68 Structure–activity relationships of flavonoids as natural inhibitors against *E. coli* β-glucuronidase

### Zimiao Weng^1^, Ping Wang^2^, Pingkun Liu^1^, Ziru Dai^2^, Guangbo Ge^2^, Jie Hou^1^

#### ^1^Dalian Medical University, Dalian, LiaoNing Province, China, 116044; ^2^Dalian Institute of Chemical Physics, Chinese Academy of Sciences, Dalian, LiaoNing Province, China, 116023

##### **Correspondence:** Jie Hou - houjie@dlmedu.edu.cn

*Journal of Chinese Medicine* 2018, **13(Suppl 1):**68

**Background:** Bacterial β-glucuronidases (GUS) play key roles in the deconjugation of a variety of endogenous and drug glucuronides, thus have been recognized as important targets to modulate the enterohepatic circulation of various glucuronides [1]. It is increasingly clear that bacterial GUS are closely related with the toxicity or intestinal disorders caused by the hydrolysis of β-d-glucuronides. Thus, it is highly desirable to find safe and potent inhibitors from friendly phytochemicals or herbals against GUS for alleviating the intestine disorders by the hydrolysis of drug glucuronides [2]. Natural flavonoids are the most abundant friendly phytochemicals in plants and famous for its antioxidant, anti-inflammatory effects and anti-diabetic activity [3].

**Materials and methods:** In this study, more than 30 natural flavonoids were collected and their inhibitory effects on *E. coli* β-glucuronidase (EcGUS) were assayed in vitro. Recombinant EcGUS was used as enzyme source, while p-Nitrophenyl-β-d-glucuronide acid (pNPG) was used as the substrate for EcGUS. The IC_50_ values were used to evaluate the inhibition capability of various flavonoids. The inhibition kinetic analysis was conducted to obtain the inhibition contant (*K*_*i*_) and to determine the inhibition kinetic types. Docking simulations were performed to reveal the interactions between the flavonoid-type EcGUS inhibitor and EcGUS.

**Results:** Among all tested flavonoids, the polyphenolic flavonoids including scutellarein, luteolin, baicalein, quercetin and scutellarin displayed strong to moderate inhibitory effects against EcGUS, with the IC_50_ values ranging from 5.09 to 29.64 μM, while isoflavones and dihydroflavones displayed weak inhibitory effects against EcGUS. Further investigation on inhibition kinetics revealed that scutellarein and luteolin functioned as potent competitive inhibitors against EcGUS-mediated PNPG hydrolysis, with the *K*_*i*_ values less than 3.0 μM. Molecular docking simulations demonstrated that scutellarein and luteolin could be well-docked into the catalytic site of EcGUS, while the binding areas of these two natural inhibitors on EcGUS were identical to that of the substrate PNPG. Additionally, as shown in Fig. 1, the structure-inhibition relationships of natural flavonoids against EcGUS are also summarized.

**Fig. 1:**
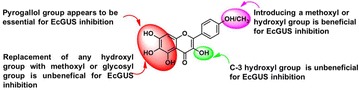
The structure-inhibition relationships of natural flavonoids against EcGUS

**Conclusions:** All these findings are very helpful for deep understanding the interactions between natural flavonoids and EcGUS, as well as for medicinal chemist to design and develop more potent flavonoid-type inhibitors against EcGUS via chemical modifications.

**References**

1. Wallace BD, Wang H, Lane KT, et al. Alleviating cancer drug toxicity by inhibiting a bacterial enzyme. Science. 2010;330:831–5.
2. Xin H, Qi XY, Wu JJ, et al. Assessment of the inhibition potential of Licochalcone A against human UDP-glucuronosyltransferases. Food Chem Toxicol. 2016;90:112–22.
3. Xiao JB, Kai GY, Yamamoto K, et al. Advance in dietary polyphenols as α-glucosidases inhibitors: a review on structure–activity relationship aspect. Crit Rev Food Sci Nutr. 2013;53:818-36.

## 69 Chemical characterization and anti-benign prostatic hyperplasia effect of total flavonoid extract of *Pteris multifida*

### Guangcheng Dai^1,2^, Fang Peng^1^, Boxin Xue^2^, Jiangyun Liu^1^, Yuxi Shan^2^

#### ^1^College of Pharmaceutical Sciences, Soochow University, Suzhou 215123, China; ^2^The Second Affiliated Hospital of Soochow University, Suzhou 215004, China

##### **Correspondence:** Jiangyun Liu - liujiangyun@suda.edu.cn; Boxin Xue - xbxcp@hotmail.com

*Journal of Chinese Medicine* 2018, **13(Suppl 1):**69

**Background:** The decoction of *Pteris multifida* had been applied to attenuate symptoms of benign prostatic hyperplasia (BPH) in Chinese folk medicine. To investigate the functional ingredients of *P. multifida* for its potential medicinal applications, the total flavonoid extract of *P. multifida* (PME) was processed and characterized, as well as evaluation of anti-BPH effect of PME.

**Results and conclusions:** High performance liquor chromatography and mass spectrometer assay revealed 10 flavonoids, including apigenin-7-*O*-β-d-glucose- 4′-*O*-α-l-rhamnoside (**1**), luteolin-7-*O*-β-d-glucose (**2**) and apigenin-7-*O*-β-d-glucose (**3**) as key constituents of PME. Biological evaluation of PME against BPH was then conducted on a testosterone-induced BPH mice model, and the results were listed in Table 1. After 60-day administration, PME (180 mg/kg, i. g.) decreased the prostate index in BPH mice apparently. Immunohistochemical assay revealed inhibition of vascular endothelial growth factor (VEGF) expression, together with activation of transforming growth factor-beta 1 (TGF-β_1_) expression in the prostatic samples administrated by PME. A 90-day subchronic toxicity test was further undertaken in male Sprague–Dawley rats, and the no-observed-adverse-effect level for PME was 200 mg/kg BW/day. These results revealed that PME exhibited anti-BPH potential with safe, which might be applied in treatment of BPH [1].

**Table 1:** Effects of PME in testosterone-induced mice model (X ± SD, n = 12)

Group	Dose (mg/kg)	Weight (g)	Prostate wet weight (g)	Prostate index (%)	VEGF	b-FGF	TGF-β_1_
Sham		40.5 ± 5.4	0.100 ± 0.016	0.25 ± 0.04*	22.1 ± 5.94**	36.1 ± 13.56*	15.6 ± 5.65
BPH Model		38.0 ± 5.2	0.123 ± 0.056	0.32 ± 0.12	31.1 ± 9.92	48.1 ± 16.61	12.2 ± 4.84
PME	20	39.2 ± 3.9	0.100 ± 0.028	0.25 ± 0.06	26.3 ± 6.23	50.9 ± 14.7	13.9 ± 4.61
	60	36.0 ± 4.0	0.102 ± 0.021	0.28 ± 0.05	27.5 ± 9.68	44.1 ± 13.5	17.4 ± 5.97*
	180	39.2 ± 3.9	0.089 ± 0.029*	0.23 ± 0.03*	21.7 ± 7.63**	38.5 ± 11.2	22.5 ± 6.63**
Prostat	29.6	39.0 ± 5.7	0.088 ± 0.028*	0.23 ± 0.06*	22.3 ± 6.54**	42.9 ± 11.15	18.3 ± 6.87**

Significantly different (* p < 0.05; ** p < 0.01) as compared with BPH model group

**Acknowledgements:** This work was supported by National Natural Science Foundation of China (Nos. U1603124 and 81460634), and Jiangsu Provincial Science and Technology Commission (BE2011671).

**Reference**

1. Dai GC, Hu B, Zhang WF, et al. Chemical characterization, anti-benign prostatic hyperplasia effect and subchronic toxicity study of total flavonoid extract of *Pteris multifida*. Food Chem Toxicol. 2017;108(B): 524–31.

## 70 Structure–inhibition relationship of flavonoids against UDP-glucuronosyltransferase 1A1

### Xin-Yu Liu^1,2^, Xia Lv^2^, Ping Wang^2^, Li-Wei Zou^2^, Guang-Bo Ge^2^, Hui Tang^1^, Ling Yang^2^

#### ^1^Key Laboratory of Xinjiang Phytomedicine Resource and Utilization, Ministry of Education, Pharmacy School of ShiHezi University, Xinjiang 832000, China; ^2^ Laboratory of Pharmaceutical Resource Discovery, Dalian Institute of Chemical Physics, Chinese Academy of Sciences, Dalian 116023, China

##### **Correspondence:** Hui Tang - Th_pha@shzu.edu.cn; Guang-Bo Ge - geguangbo@dicp.ac.cn

*Journal of Chinese Medicine* 2018, **13(Suppl 1):**70

**Background:** Uridine-disphosphate glucuronosyltransferase 1A1 (UGT1A1), one of the most important phase II conjugative enzymes, plays key role in the elimination and detoxification of a host of potentially harmful compounds (such as bilirubin) and clinical drugs (such as etoposide and diethylstilbestrol). Therefore, it is of great significance to systematically evaluate the inhibitory effects of natural products in dietary supplements (such as flavonoids) against human UGT1A1 [1,2]. A previous study by us has developed a specific fluorescent probe (**NCHN**) for UGT1A1, This study aimed to explore the structure–inhibition relationships of flavonoids against human UDP-glucuronosyltransferase UGT1A1 using a high-throughput screening method.

**Methods:** More than thirty natural flavonoids have been collected and assayed with the probe **NCHN** which can be used for high-throughput screening (HTS) and characterization of UGT1A1 inhibitors by using human liver microsomes (HLM) and UGT1A1 as the enzyme source in this paper [3]. To research the effect of inhibition against UGT1A1 mediated NCHN-*O*-glucuronidation in HLM, and select the suitable concentration of inhibitor (flavonoids) to determine the IC_50_ value; According to the IC_50_ value, the compounds which has the strongest inhibitory effect (IC_50_ <5 μmol L^−1^) would be the chosen to proceed the next study; The single enzymes and HLM were used as enzyme sources, respectively. With the IC_50_ value and the suitable concentration of substrate which determined by enzyme kinetics, the compound inhibited glucuronyl transferase enzyme inhibition kinetics experiment was studied to determine the inhibition constants *K*_*i*_ of the compound and its inhibit competitive type, respectively.

**Results:** The results demonstrated that kaempferol which with multiple phenolic groups displayed strong inhibition against UGT1A1 mediated NCHN-O-glucuronidation in HLM and UGT1A1(IC_50_<5 μM)in these flavoids, the IC_50_ values of kaempferol was determined as 3.34 and 2.44 μM, respectively. Further investigation on the inhibitory behaviors of kaempferol demonstrated that tested nature flavonoids are non-competitive inhibitors against UGT1A1 mediated NCHN-*O*-glucuronidation, at the same time, that is competitive inhibitors against HLM, UGT1A1 mediated NCHN-O-glucuronidation, with the *K*_*i*_ values are 1.74 and 0.90 μM, respectively. While, the glycosyl flavonoids are hardly to inhibit UGT1A1 (IC_50_ > 100 μM) in this study. What’s more, the saturated flavonoids displayed weaker inhibition against UGT1A1 mediated NCHN-*O*-glucuronidation in HLM than that of unsaturated flavonoids.

**Conclusion:** Different types of flavonoids and flavonoids with different structure expressed different levels inhibition against UGT1A1 mediated NCHN-*O*-glucuronidation in HLM. At the same time it seems to be more inclined to develop flavonone as novel flavonoid drugs for the pharmaceutical chemists. All these findings were extraordinary helpful for us to explore the potential structure-inhibition relationships about flavonoids as UGT1A1 inhibitors, which was also very useful to designing and developing more potent flavonoid-type inhibitors against UGT1A1 for the pharmaceutical chemists, as well as for the development of novel flavonoid drugs with improved safety profiles.

**Keywords:** Flavonoids; UGT1A1; Structure–inhibition relationship; High-throughput screening.

**References**

1. Xin–Xin Wang, Guang-Bo Ge, et al. Toxicol Appl Pharmacol. 2015;289:70–8.
2. Xia Lv, Xin–Xin Wang et al. Toxicol Appl Pharmacol. 2016;301:42–9.
3. Xia Lv, Guang-Bo Ge, et al. Biosens Bioelectr. 2015;72:261–7.

## 71 Impact of *Debaryomyces hansenii* treatment on intestinal microorganism in mice with antibiotic-induced diarrhea

### Lu He^1^, Chengxing Long^1,2^, Youjia Liu^1^, Yanfang Guo^1^, Nenqun Xiao^1^, Zhoujin Tan^1^

#### ^1^Hunan University of Chinese Medicine, Changsha, Hunan Province, China; ^2^Hunan University of Humanities Science and Technology, LouDi, Hunan Province, China

##### **Correspondence:** Zhoujin Tan - 18229725249@163.com; Nenqun Xiao - xiaonenqun@sohu.com; tanzhjin@sohu.com

*Journal of Chinese Medicine* 2018, **13(Suppl 1):**71

**Background:** *Debaryomyces hansenii*, which was isolated from food or intestinal contents of experimental mice, was identified as a halotolerant yeast with a high biotechnological potential, particularly in food industry. It has been demonstrated that *Debaryomyces hansenii* adjusts the microecosystem balance and has curative effect on antibiotic-induced diarrhea. Our objective was to investigate the influence of *Debaryomyces hansenii* treatment on intestinal microorganisms of mice with symptom of antibiotics-induced diarrhea.

**Materials and methods:** Eighteen specific pathogen free (SPF) mice were randomly selected into three groups: healthy control group, diarrhea control group and diarrhea treated group. Mice model with antibiotic-induced diarrhea was constructed by gavaging mixed antibiotics (23.33 mL kg^−1^ day^−1^) composed of gentamycin sulfate and cefradine for 5 days. After the success of modeling, mice were treated with *D. hansenii* by intragastric administration. In the control group, mice were given steriled water. Four days after treatment, total DNA of intestinal microflora of treated and control mice was extracted, and their quantities were measured by sequencing the V4 region of 16S rDNA.

**Results:** When compared with the control, treatment with *D. hansenii* enhanced the operational taxonomic units (OTUs) of intestinal bacteria. The density and diversity of intestinal bacterial population were recovered in treated mice. While, there was no significant impact on theirs at genus level. Interestingly, the treatment recovered the population density of certain bacterium species, such as *Bacteroidaceae* (in family level).

**Conclusion:** Our results indicate that the ecosystem of intestinal bacteria is adaptable and adjustable with treatment of *D. hansenii.*

**Acknowledgements:** This work was supported by grants from the National Natural Science Foundation of China (No. 81573951).

## 72 MYC2 have an important role in biosynthesis of active compounds in *Salvia miltiorrhiza Bunge*

### Wenzhi Cao, Fenfen Huang, Min Shi, Yao Wang and Guoyin Kai

#### Institute of Plant Biotechnology, College of Life and Environment Sciences, Shanghai Normal University, Shanghai 200234, China

##### **Correspondence:** Guoyin Kai - gykai@hotmail.com

*Journal of Chinese Medicine* 2018, **13(Suppl 1):**72

**Background:** MYCs are a key transcription factors in the JA signaling pathway and are important members of the family of bHLH transcription factors. Previous studies have shown that MYC2 is a very important positive regulator in the MYCs family and plays a vital role in JA, ABA signaling pathway and has positive effects on the biosynthesis of flavonoids (such as anthocyanins). *Salvia miltiorrhiza Bunge* is a kind of traditional Chinese medicine, its roots and stems have a high medicinal value which can which plays a crucial role in curing cerebrovascular diseases, irregular period, blood circulation and anti-cancer. The active ingredient of *S. miltiorrhiza* is mainly hydrosoluble phenolic acids and liposoluble tanshinone. However, the effects of MYC2 on the secondary metabolism of *S. miltiorrhiza* is rarely known.

**Results:** We firstly introduced the transcription factor *AtMYC2* from *Arabidopsis thaliana* into *S. miltiorrhiza* hairy roots, the results showed that *AtMYC2* could not only improve the tanshinones content, but also improve the salvanic acid content. Subsequently, based on the sequence of *AtMYC2*, we cloned a sequence from *S. miltiorrhiza* which was high homology with *AtMYC2* and named it *SmMYC2*. Then we constructed the *SmMYC2* overexpressing vector and obtained the transgenic hairy roots. The results showed that *SmMYC2* could significantly increase the content of tanshinone compared with the control group.

**Conclusion:** Overexpression of *AtMYC2* and *SmMYC2* can significantly induce the accumulation of active compounds in *S. miltiorrhiza* which indicates that *MYC2* play a positive role in the secondary metabolism of *Salvia miltiorrhiza.*

**Keywords:** *Salvia miltiorrhiza*, tanshinones, Salvanic acid, *MYC2,* secondary metabolism.

## 73 Assessment of the inhibition potential of Gambogenic acid against human UDP-glucuronosyltransferases

### Xiaoya Sun^1,2^, Shiyang Li^2^, Xia Lv^2^, Hui Tang^1^, Guangbo Ge^2^

#### Key Laboratory of Xinjiang Phytomedicine Resource and Utilization, Ministry of Education, Pharmacy School of Shihezi, Xinjiang 832000, China; ^2^Laboratory of Pharmaceutical Resource Discovery, Dalian Institute of Chemical Physics, Chinese Academy of Sciences, Dalian 116023, China

##### **Correspondence:** Hui Tang - Th_pha@shzu.edu.cn; Guangbo Ge - geguangbo@dicp.ac.cn

*Journal of Chinese Medicine* 2018, **13(Suppl 1):**73

**Background:** Gambogenic acid (GNA) is one of the major bioactive compounds in Gamboge. Recently, increasing evidence has indicated that GNA exerts promising anti-tumor effects [1]. Previous LC-UV fingerprint and UGT1A1 inhibition profile studies have indicated that the extract of gamboge exhibited evident inhibitory effects against UGT1A1 in human liver microsomes (HLMs) and GNA showed most potent inhibition on UGT1A1. However, there is no reported evidence currently on the inhibitory effects of GNA against common phase II drug metabolizing enzymes. In this study, we investigated GNA inhibition against 4-methylumbelliferone (4-MU) glucuronidation and N-3-carboxypropyl-4-hydroxy-1,8-naphthalimide(NCHN) glucuronidation.

**Materials and methods:** Recombinant UGTs-catalyzed 4-MU-glucuronidation reaction was preliminary employed to evaluate the inhibition potential of GNA towards various UGTs. Meanwhile, a novel applicable ratiometric fluorescent probe for highly selective sensing the enzyme activity of UGT1A1 [2], NCHN, was utilized to determine the inhibitory effects induced by GNA.

**Results:** The results showed that GNA exhibited strong inhibitions on six major human recombinant UGTs, including UGT1A1, UGT1A3, UGT1A6, UGT1A8, UGT1A10, and UGT2B7 with IC_50_ values of 4.83 ± 0.64, 2.50 ± 0.44, 9.13 ± 2.10, 5.65 ± 0.41, 2.37 ± 0.21, and 4.71 ± 1.19 μM respectively. The inhibition kinetic parameters (*K*_*i*_) were determined as 2.28, 1.17, 10.01, 5.04, 5.08, and 6.46 μM for UGT1A1, 1A3, 1A6, 1A8, 1A10 and 2B7. The experiments also found that GNA presented potent noncompetitive inhibition against NCHN-*O*-glucuronidation in both HLM and recombinant UGT1A1 as enzyme source, and the *K*_i_ values was determined as 7.64 μM in HLM and 0.48 μM in recombinant UGT1A1.

**Conclusion:** All these findings are not only useful for guiding reasonable applications of GNA and its related medical preparations, but also important in laying out the foundation for further in vivo investigations on the HDIs risks caused by GNA-associated inhibition against human UGTs. GNA is an Broad-spectrum inhibitor against most UGTs (Fig. 1).

**Fig. 1:**
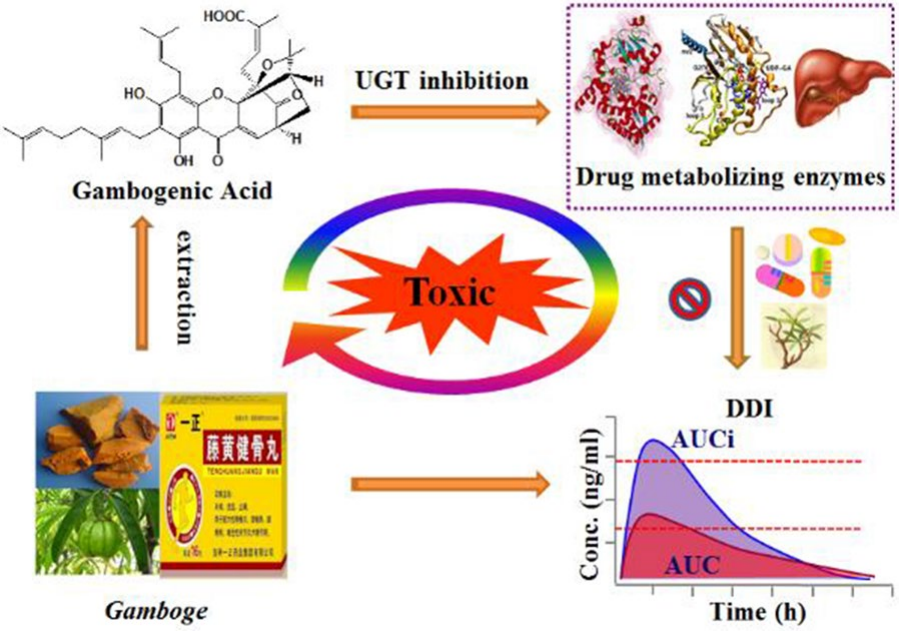
Assessment of the inhibition potential of GNA towards human UGTs

**References**

1. Xia L, Xin–Xin W, Jin–Jin W, et al. Toxicol Appl Pharmacol. 2016;301:42–9.
2. Xia L, Guang-Bo G, Lei F, et al. Biosens Bioelectron. 2015;72:261–7.

## 74 Effects of different polarities from *Ganoderma lucidum* on glucolipid metabolism and gut microbiota of rats fed with high fat diet

### Weiling Guo^1^, Tiantian Li^1^, Ruibo Jia^1^, Zirui Huang^1^, Yuyang Pan^1^, Xucong Lv^1,2^, Bin Liu^1,2^

#### ^1^College of Food Science, Fujian Agriculture and Forestry University, Fuzhou, Fujian 350002, China; ^2^National Engineering Research Center of JUNCAO Technology, Fujian Agriculture and Forestry University, Fuzhou, Fujian 350002, China

##### **Correspondence:** Bin Liu - liubin618@hotmail.com

*Journal of Chinese Medicine* 2018, **13(Suppl 1):**74

**Background:** Lipid metabolic is a cluster of metabolic disorders that leads to type 2 diabetes and mortality. Among the many biologically active constituents of *Ganoderma lucidum*, polysaccharides, proteoglycans, proteins and triterpenoids have been shown to have hypoglycemic effects, enhanced immunity, anti-aging, liver protection, anti-cancer, hypolipidemic and health.

**Materials and methods:** The aim of the present study was to determine if *G. lucidum*’s extracts (95% ethanol, 55% ethanol, water and enzymatic method) that have an effect lipid metabolic in the high-fat-diet fed rats. The effects of *G. lucidum*’s extracts on gut microbiota composition was analyzed by 16S rRNA(V3–V4 region) high-throughput sequencing (HTS) in caecal faeces.

**Results:** The results showed, as compared with the weight of Wistar rats in the control group, there are significant (p < 0.05) increase in model group. The biochemical indexes and the changes of the faecal and intestinal contents flora in experimental rats were analyzed. The results showed that the animal serum levels of triglyceride (TG), total cholesterol (TC), low density lipoprotein cholesterol (LDL-C), alanine aminotransferase (ALT), aspartate aminotransferase (AST) in the control group was significantly lower than those in the model group (HFD), and (HDL-C) was higher than that in HFD; *Lachnospiraceae, Porphyromonadaceae* and *Barnesiella* in the experimental group were significantly lower than the model groud; *Prevotella, Ruminococcaceae* and *Clostridiales* in the experimental group were significantly higher than the model group.

**Conclusions:** Our data confirmed that extracts of *G. lucidum* can effective in reducing the serum levels and the body weight. The results provided a reference for the exploitation of *G. lucidum* which would be significant to sustainable development of industry and agriculture, full utilization of resources.

## 75 Platycodin D, a natural product isolated from Platycodonis Radix, displays potential anticancer effects as single or combination therapy

### Ting Li, Zhenghai Tang, Xin Chen, Xiaohuang Xu, Jinjian Lu

#### State Key Laboratory of Quality Research in Chinese Medicine, Institute of Chinese Medical Sciences, University of Macau, Macao, China

##### **Correspondence:** Jinjian Lu - jinjianlu@umac.mo

*Journal of Chinese Medicine* 2018, **13(Suppl 1):**75

**Background:** Platycodin D (PD) is a triterpenoid saponin isolated from the Chinese herb Platycodonis Radix. It possesses various biological activities, such as immune stimulation, anti-nociception, anti-obesity and anti-cancer, etc.

**Materials and methods:** MTT, colony formation assay, flow cytometry, western blotting, transwell assay, immunoprecipitation, siRNA, real-time PCR and xenograft models were adopted to study the anticancer potential and mechanisms of PD.

**Results:** PD exhibited potential effects on the anti-adhesion, antimigration, anti-invasion as well as antiproliferation activities in cancer cells [1]. PD induced apoptosis and enhanced the anticancer properties of the chemotherapy drug doxorubicin via promotion of apoptotic activity [1,2]. In addition, PD triggered protective autophagy via ERK-JNK pathways in cancer cells [3,4]. We further demonstrated PD as a novel Hsp90 inhibitor by disrupting the protein–protein interaction of Hsp90 and its chaperone Cdc37 (unpublished data). This disruption by PD resulted in degradation of Hsp90 client proteins without feedback increase of Hsp70 (unpublished data). AKT and mTOR inhibitors were found to increase the expression of EGFR, HER-2 and IGF1R upon AKT/mTOR inhibition, while PD reduced the total levels of EGFR, HER-2 and IGF1R expression. Co-treatment of the AKT inhibitor or mTOR inhibitor with PD blocked this feedback activation and triggered enhanced antiproliferation activity and apoptotic effect ^[5]^.

**Conclusions:** These findings not only identified PD is a potential agent for cancer treatment, but also establish a novel mechanistic rationale for the combination approach with PD.

**Acknowledgments:** This study was funded by the grants from Science and Technology Development Fund, Macao S.A.R (FDCT) (024/2016/A1, 070/2013/A and 074/2012/A3), Research Fund of University of Macau (MYRG2015-00091-ICMS-QRCM and MYRG2015-00101-ICMS- QRCM).

**References**

1. Li T, Xu WS, Wu GS, et al. *A*sian Pac J Cancer P. 2014;15:1745–9.
2. Tang ZH, Li T, Gao HW, Lu JJ, et al. Chin Med-Uk. 2014;9.
3. Li T, Tang ZH, Xu WS, et al. Eur J Pharmacol. 2015;749:81–8.
4. Li T, Xu XH, Tang ZH, et al. Acta Pharmacol Sin. 2015;36:1503–13.
5. Li T, Chen X, Chen X, et al. Sci Rep. 2016;6:37997.

## 76 Supramolecular encapsulation of pesticides

### Ruibing Wang, Xue Yang

#### State Key Laboratory of Quality Research in Chinese Medicine, Institute of Chinese Medical Sciences, University of Macau, Taipa, Macau SAR

##### **Correspondence:** Ruibing Wang - rwang@umac.mo

*Journal of Chinese Medicine* 2018, **13(Suppl 1):**76

**Background:** Pesticides have been used as a major means of pest control for nearly a century in agriculture for higher-yield and higher-quality crop. However, it has been clear that extended exposure to pesticides would harm to environment and human health. For instance, it was reported that extended exposed of anabasine (ANA) caused different degree of toxic signs, and even resulted in the fetal cleft palates in pregnant goats [1, 2]. In this study, we aimed to investigate the binding behaviors between cucurbit[7]uril (CB[7]) and ANA, and examine the influence of CB[7]’s encapsulation on the toxicity of the pesticide.

**Materials and methods:** The chemical behaviors between CB[7] and ANA were examined by ^1^H NMR, electrospray ionization mass spectrometry (ESI–MS), UV–visible absorbance spectroscopy, isothermal titration calorimetry (ITC), and molecular modeling. Zebrafish embryos were selected as in vivo models to investigate the toxicity.

**Results:** ^1^H NMR, ESI–MS, as well as ITC suggested that ANA formed 1:1 binding complexes with CB[7], with a relatively strong binding affinity (10^5^ M^−1^). Regarding the toxicity of the pesticide, as shown in Fig. 1A, compared with the control group, the viable hatching rate of embryos treated with 300 μM ANA was significantly lower, (ca. 20%), suggesting dramatic embryonic toxicity of ANA. In contrast, a much higher hatching rate (ca. 90%) was observed when ANA was encapsulated by CB[7]. Similar trend was observed in the survival rate of embryos treated with a variety of concentrations of ANA in the absence and in the presence of CB[7] (Fig. 1B).

Moreover, relatively high concentrations (200–300 μM) of ANA induced serious physical malformations during the embryonic development, including retarded growth, yolk sac edema, spinal deformation, and yolk sac deformity and tail malformation. In contrast, in the presence of CB[7], the treated embryos didn’t show any obvious malformation compared with the free ANA group. The results demonstrated that CB[7] can decrease the teratogenic effect caused by ANA.

**Fig. 1:**
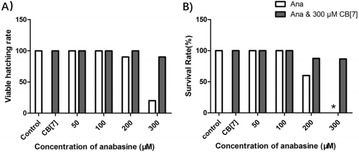
Viable hatching rate (**A**) and survival rate (**B**) of zebrafish exposed with various concentrations of ANA in the absence and in the presence of 300 μM CB[7]for 48 h. ‘*’ means zebrafish embryos in this group all died at the end of exposure

**Conclusions:** In summary, our results demonstrated with in vivo zebrafish models that supramolecular encapsulation of pesticides by CB[7] may significantly reduce the fetal and developmental toxicities of these pesticides.

**Acknowledgements:** Macau Science and Technology Development Fund (FDCT Grant No.: FDCT/020/2015/A1) is gratefully acknowledged for providing financial support.

**References**

1. Keeler RF, Crowe MW. Teratogenicity and toxicity of wild tree tobacco, Nicotiana glauca in sheep. Cornell Vet. 1984;74:50–9.
2. Stein S, Dunsche A, Gellrich NC, Harle F, Jonas I. One- or two-stage palate closure in patients with unilateral cleft lip and palate: comparing cephalometric and occlusal outcomes. Cleft Palate Craniofac J. 2007;44:13–22.

## 77 Microcosamines D and E, two new piperidine alkaloids from the leaves of *Microcos paniculata* L.

### Gang Zhang, Yunghusan Chen, Li Xu, Dan Chen, Zhongwei Chen, Yayuan Yu, Qihuang Lin, Lianzhong Luo

#### Technology and Engineering Center for Marine Biomedical Resource Utilization, Fujian Province University, Xiamen Medical College, Xiamen 361008, China

##### **Correspondence:** Lianzhong Luo - lzluo@xmu.edu.cn

*Journal of Chinese Medicine* 2018, **13(Suppl 1):**77

*Microcos paniculata* L., a member of the family Malvaceae, is mainly distributed in tropical and subtropical areas of South and Southeast Asia [1]. In China, the leaves of *M. paniculata* L. are commonly used in the treatment of colds, sore throat, abdominal distention, jaundice, and so on [2]. Previous phytochemical studies on *M. paniculata* L. led to the isolation of triterpenoids, flavonoids and minor components of 2, 3, 6-trisubstituted piperidine alkaloids with a variety of biological activities [3–5]. As part of an ongoing investigation on the discovery of naturally occurring bioactive compounds from this plant, two new new piperidine alkaloids, microcosamine D (**1**) and microcosamine E (**2**) were obtained from the leaves of *M. paniculata* L. Their structures were elucidated by extensive spectroscopic analyses (1D and 2D NMR, UV, and HR-ESI–MS). The isolates were found to be inactive (IC_50_ > 100 μM) against four selected tumor cell lines (HeLa, HepG2, A549 and MCF-7) (Fig. 1).

**Fig. 1:**
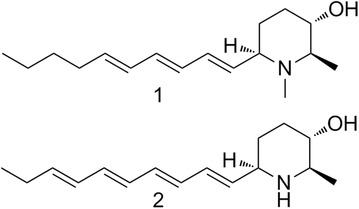
Structures of microcosamines D (**1**) and E (**2**)

**References**

1. Zhang G, Zhang N, Xu L, et al. Nat Prod Res. 2017;31:169–74.
2. Luo JP, Zhang LP, Yang SL, et al. Acta Pharm Sin. 2009;44:150–3.
3. Feng SX, Lin LD, Xu HH, et al. Nat Prod Res. 2008; 10:1155–8.
4. Bandara KANP, Kumar V, Jacobsson U, et al. Phytochemistry. 2000;54:29–32.
5. Still PC, Yi B, González-Cestari TF, et al. J Nat Prod. 2013;76:243–9.

## 78 In Vitro xanthine oxidase inhibition by *Chrysanthemum morifolium* and its mode of inhibition

### Ng Teng Lit^1^, Loh Khye Er^1^, Tan Hui Yin^1^, Intan Safinar Ismail^2^

#### ^1^Department of Bioscience and Sport Science, Faculty of Applied Sciences and Computing, Tunku Abdul Rahman University College, 53300 Kuala Lumpur, Malaysia; ^2^Laboratory of Natural Products, Institute of Bioscience, University of Putra Malaysia, 43400 Serdang, Selangor, Malaysia

##### **Correspondence:** Loh Khye Er - lohke@tarc.edu.my

*Journal of Chinese Medicine* 2018, **13(Suppl 1):**78

In the present study, *Chrysanthemum morifolium* was extracted with hydro-methanol solution to obtain ethyl acetate (EA), petroleum ether (PE) and residual fractions (RF). The Xanthine Oxidase (XO) inhibition potential of various fractions was further determined by enzyme kinetics. Lineweaver–Burk plots showed that EA fraction exhibited the highest potency of 49.55 ± 0.92%; IC_50_ = 10.86 ± 0.86 µg/mL, followed by the RF fraction, 28.55 ± 5.58%; IC_50_ = 101.77 ± 13.44 µg/mL, PE fraction, 22.79 ± 8.22%; IC_50_ = 58.07 ± 10.79 μg/mL and crude fractions, 16.58 ± 3.35%; IC_50_ value = 150.11 ± 12.30 µg/mL. The IC_50_ value for allopurinol (positive control) was 9.85 ± 0.15 µg/mL. Enzyme inhibited mode indicated that RF fraction can produce competitive inhibition, whereas crude, EA and PE fractions were of non- competitive inhibitions. *C. morifolium* can serve as potential XO inhibitors that applied in treatment herbal remedies for gout, arthritis and other XO-related disorders.

**Acknowledgements:** The authors wish to thank the Ministry of Higher Education (MOHE) for financial support trough FRGS, Project Number FRGS/2/2014/SG05/TARUC/02/2.

**References**

1. Milind P, Sushila K, Neeraj S. Understanding gout beyond doubt. IRJP. 2013;4:9.
2. Newman DJ, Cragg GM. Natural products as sources of new drugs over the last 25 years. J Nat Prod. 2007;70:461–77.
3. Sweeney AP, Wyllie SG, Shalliker RA, Markham JL. Xanthine oxidase inhibitory activity of selected Australian native plants. J Ethnopharmacol. 2001;75:273–7.

## 79 Phytochemical screening and antimicrobial effect of *Rubus ellipticus* from Nepal

### Rishav Acharya, Prakash Subedi, Ajaya Bhattarai

#### Department of Chemistry, Mahendra Morang Adarsh Multiple Campus, Tribhuvan University, Biratnagar, Nepal

##### **Correspondence:** Ajaya Bhattarai - bkajaya@yahoo.com

*Journal of Chinese Medicine* 2018, **13(Suppl 1):**79

**Background:** Plants containing bioactive compounds which are used for curing different human diseases are termed as Medicinal Plants. The main objective of our research work was to check the presence or absence of the phytochemical constituents in the selected medicinal plants and also to check their antimicrobial effect.

**Materials and methods:** This present study involves phytochemical analysis from aqueous leaf extract of medicinal plants of taxonomical identification *Rubus ellipticus*, which were collected from Dhankuta region of Nepal for the phytochemical screening.

**Results:** The different test was performed for the qualitative tests of leaf extract and the methanol extract was checked for antimicrobial effect against four bacterial strains: *Pseudomonas aerogens*, *Staphylococcus aureus*, *E. coli*, and *K. Pneumonia*.

*Rubus ellipticus* contain all the phytochemicals (Flavonoid, alkaloid, tannin, saponin, terpenoid) and it showed sensitivity towards gram-positive bacteria i.e. *S. aureus* and *Pseudomonas aerogens* and resistance to *E. coli* and *K. pneumonia*.

**Conclusions:** It is expected that the important phytochemical recognized in our medicinal plant will be helpful in curing different diseases and led a base to study on *R. ellipticus* leaves in food and pharmaceutical application.

## 80 Effect of ultrafine grinding on physicochemical and antioxidant properties of dietary fiber from wine grape pomace

### Fengmei Zhu^1^, Bin Du^2^, Jun Li^1^

#### ^1^College of Food Science and Technology, Hebei Normal University of Science and Technology, Qinhuangdao 066600, China; ^2^Analysis and Testing Center, Hebei Normal University of Science and Technology, Qinhuangdao 066600, China

##### **Correspondence:** Jun Li

*Journal of Chinese Medicine* 2018, **13(Suppl 1):**80

**Background:** Dietary fiber (DF), defined as “edible parts of plants or analogous carbohydrate that are resistant to digestion and absorption in the human small intestine with complete or partial fermentation in the large intestine” [1], is abundant in plant products such as fruits, vegetables, and grains. DF has attracted increasing interests in recent years as many studies have revealed that it might be involved in disease preventive and health promotive activities, including attenuation of blood cholesterol and glucose, laxative effect and reduction of risk of colon cancer, heart disease and obesity [2, 3].

**Materials and methods:** Wine grape pomace dietary fiber (DF) powders was prepared by superfine grinding, whose effects were investigated on the composition, functional and antioxidant properties of the wine grape pomace DF products. The antioxidant activities of wine grape pomace and DF before and after grinding were in terms of 1,1-diphenyl-2-picrylhydrazyl (DPPH) radical scavenging activity, 2,2′-azinobis(3-ethylbenzothiozoline-6-sulfonic acid) diammonium salt (ABTS) radical scavenging activity, ferric reducing antioxidant power (FRAP) and total phenolic content (TPC).

**Results:** The results showed that superfine grinding could effectively pulverize the fiber particles to submicron scale. As particle size decrease, the functional properties (water holding capacity, water retention capacity, swelling capacity, oil binding capacity and nitrite ion absorption capacity) of wine grape pomace DF were significantly (*p* < 0.05) decreased and a redistribution of fiber components from insoluble to soluble fractions was observed.

**Conclusions:** Compared with DF before and after grinding, micronized insoluble DF showed increased ABTS radical scavenging activity, ferric reducing antioxidant power and TPC yet decreased DPPH radical scavenging activity. Positive correlations were detected between ABTS radical scavenging activity, FRAP and TPC.

**References**

1. AACC report. American Association of Cereal Chemists. 2001. 46;112–26.
2. Zhu KX, Huang S, Peng W, et al. Food Res Int. 2010;43:943–8.
3. Huang YL, Sheu F, Lee MH, et al. J Sci Food Agr. 2007;88:435–41.

## 81 Nutrients, phytochemicals and antioxidant activities of 26 kidney bean cultivars

### Lijiao Kan^1^, Shaoping Nie^1^, Jielun Hu^1^, Sunan Wang^2^, Steve W. Cui^3^, Yawen Li^1^, Sifan Xu^1^, Yue Wu^1^, Junqiao Wang^1^, Zhouya Bai^1^, Mingyong Xie^1^

#### ^1^State Key Laboratory of Food Science and Technology, Nanchang University, Nanchang 330047, China; ^2^Canadian Food and Wine Institute, Niagara College, 135 Taylor Road, Niagara-on-the-Lake, Ontario L0S 1J0, Canada; ^3^Agriculture and Agri-Food Canada, Guelph Food Research Centre, 93 Stone Road West, Guelph, Ontario NIG 5C9, Canada

##### **Correspondence:** Shaoping Nie - spnie@ncu.edu.cn

*Journal of Chinese Medicine* 2018, **13(Suppl 1):**81

**Background:** There was a positive association between the consumption of kidney beans and the reduction of the risk of cardiovascular diseases, obesity, and certain types of cancer.

**Materials and methods:** In this study, 26 kidney beans varying in cultivars and growth conditions were assessed for their nutritional and phytochemical compositions, and antioxidant activities. The moisture, ash, fat, protein, dietary fiber and starch were determined using AOAC methods (AOAC, 2005). Sugars were analyzed by HPLC equipped with a refraction index (RI) detector. Anthocyanins were identified by UHPLC-MS. Tocopherols were analyzed by UPLC-FLD (fluorescence detector). Total phenolics, total flavonoids and antioxidant activities (DPPH radical scavenging activities and ferric reducing antioxidant power (FRAP) assay) were measured by the microplate reader (Thermo Varioskan Flash).

**Results:** The kidney beans contained high levels of dietary fiber (29.32–46.77%), resistant starch (9.16–18.09%) and protein (22.06–32.63%) but low levels of lipid (1.05–2.83%) and sugars (1.55–9.07%). 16 anthocyanins were identified including delphinidin-3-galactoside, delphinidin-3-glucoside, cyanidin-3-galactoside, cyanidin-3-glucoside, petunidin-3-galactoside, petunidin-3-glucoside, pelargonidin-3-galactoside, pelargonidin-3-glucoside, malvidin-3-galactoside, malvidin-3-glucosidem, cyanidin-3-sambubiose, 3, 5-diglucoside of cyanidin, pelargonidin, petunidin and malvidin, and malvidin-3, 5-digalactoside. The total tocopherol content was in the range of 12.83−68.35 μg/g, predominantly γ-tocopherol, followed by δ-tocopherol. In addition, certain levels of total phenolics and flavonoids with respective values of 0.25–3.79 mg gallic acid equivalent/g dry weight and 0.19–7.05 mg rutin equivalent/g dry weight resulted in significant antioxidant activities. And a good correlation was observed between TPC and FRAP values (R^2^ = 0.8030).

**Conclusions:** The results suggested that kidney beans could be a potential dietary source of certain health-promoting compounds including protein, dietary fiber, resistant starch, anthocyanins and antioxidants.

Notes: This article named “Nutrients, phytochemicals and antioxidant activities of 26 kidney bean cultivars” has been included by the *Food Chemical and Toxicology* on its special issue on phytochemicals in medicine and food.

## 82 Network pharmacology studies on the bioactive compounds and action mechanisms of natural products for the treatment of diabetes mellitus

### Weiwei Li, Guoqi Yuan, Yuxiang Pan, Cong Wang, Haixia Chen

#### School of Pharmaceutical Science and Technology, Tianjin University, Tianjin, P. R. China

##### **Correspondence:** Haixia Chen - chenhx@tju.edu.cn

*Journal of Chinese Medicine* 2018, **13(Suppl 1):**82

**Background:** Diabetes mellitus (DM) is a kind of chronic and metabolic disease, which can cause a number of diseases and severe complications. It is caused by either the body can’t produce enough insulin or the body can’t effectively respond to the produced insulin. Natural products have played an important role in DM treatment due to multi-components and multi-targets to produce combined or synergistic effects. Network pharmacology approach, integrating network biology and pharmacology, is introduced to study DM, which can combine the drugs, target proteins and disease and form drug-target-disease networks [1]. It has been widely used in the studies of the bioactive compounds and action mechanisms of natural products for the treatment of DM.

**Results:** Natural extracts, polysaccharides and polyphenols are the main components used to DM therapy. Super Natural II, NAPRALERT, Chemical Entities of Biological Interest and DrugBank are the related databases. The action mechanisms related to type 2 DM (T2DM) include α-amylase and α-glucosidase inhibitory, targeting β cell dysfunction, targeting signal pathways [2–8] (AMPK, PI3K/Akt, mTOR, JAK-STAT, ROS-ERK-NF-κB, Wnt and IGF-1 signal pathways) (Fig. 1) and modulation of gut microbiota. T2D-Db, T2DGADB and T2D@ZJU, the databases related to DM, were developed to integrate these information. The high availability of databases provides new opportunities for data integration and can be used to predict the target network of the bioactive constituents. Network pharmacology has been widely applied on the treatment of DM. Based on network pharmacology, the mechanisms of Ge-Gen-Qin-Lian decoction and Tangminling Pills used to the treatment of T2DM were elucidated.

**Fig. 1:**
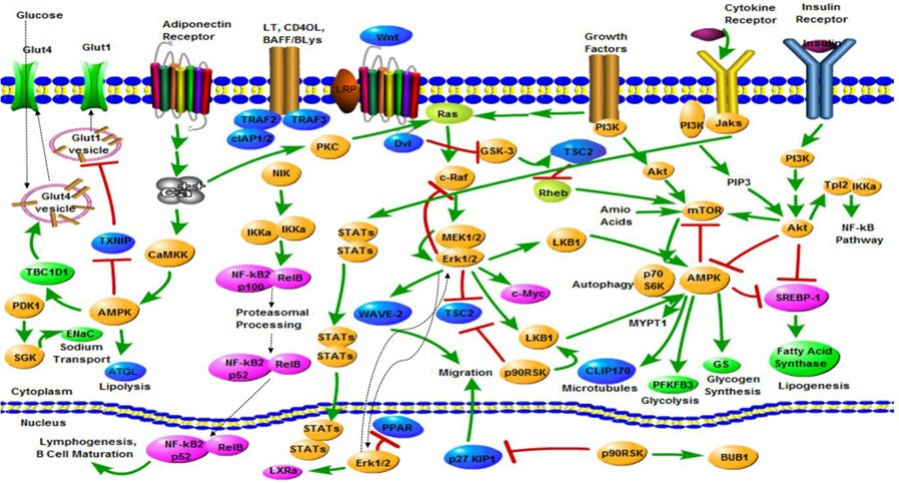
Summary of signal pathways related to diabetes mellitus

**Conclusions:** The appropriate use of network pharmacology may initiate new directions, overcome the disadvantages of current antidiabetic therapies as well as contribute new insights into the discovery of novel antidiabetic drugs.

**Acknowledgements:** Our work was financially supported by the grant from the Natural Science Foundation of China (NSFC 31371879) and National High Technology Research and Development Program (“863” Program) of China (Grant No. SS2013AA100207).

**References**

1. Poornima P, Kumar JD, Zhao Q, Blunder M, Efferth T. Network pharmacology of cancer: from understanding of complex interactomes to the design of multi-target specific therapeutics from nature. Pharmacol. Res. 2016;111:290–302.
2. Kurimoto Y, Shibayama Y, Inoue S, Soga M, Takikawa M, Ito C, et al. Black soybean seed coat extract ameliorates hyperglycemia and insulin sensitivity via the activation of AMP-activated protein kinase in diabetic mice. J Agric Food Chem. 2013;61:5558–64.
3. Li S, Chen H, Wang J, Wang X, Hu B, Lv F. Involvement of the PI3K/Akt signal pathway in the hypoglycemic effects of tea polysaccharides on diabetic mice. Int J Biol Macromol. 2015;81:967–74.
4. Siegel N. The mTOR pathway and its role in human genetic diseases. Mutat Res. 2008;659:284–92.
5. Gurzov EN, Stanley WJ, Pappas EG, Thomas HE, Gough DJ. The JAK-STAT pathway in obesity and diabetes. FEBS J. 2016;283:3002–15.
6. Liu M, Qin J, Hao Y, Liu M, Luo J, Luo T, et al. Astragalus polysaccharide suppresses skeletal muscle myostatin expression in diabetes: involvement of ROS-ERK and NF-kB pathways. Oxid Med Cell Longev. 2013;12:782497.
7. Chiang YT, Ip W, Jin T. The role of the Wnt signaling pathway in incretin hormone production and function. Front Physiol. 2012;3:1–14.
8. Siddle K. Signalling by insulin and IGF receptors: supporting acts and new players. J Mol Endocrinol. 2011;47:1–10.

## 83 Yunnaneic acid B is a bioactive component of a medicinal plant—*Pulmonaria officinalis*

### Joanna Kolodziejczyk-Czepas^1^, Łukasz Pecio^2^, Anna Stochmal^2^, Pawel Nowak^1^, Justyna Krzyżanowska-Kowalczyk^2^

#### ^1^Department of General Biochemistry, Faculty of Biology and Environmental Protection, University of Lodz, Pomorska 141/143, 90-236 Lodz, Poland; ^2^Department of Biochemistry, Institute of Soil Science and Plant Cultivation, State Research Institute, Czartoryskich 8, 24-100 Pulawy, Poland

##### **Correspondence:**Joanna Kolodziejczyk-Czepas - joanna.kolodziejczyk@biol.uni.lodz.pl

*Journal of Chinese Medicine* 2018, **13(Suppl 1):**83

**Background:** The work was based on in vitro evaluation of radical scavenging ability and antioxidant action of yunnaneic acid B, isolated from *Pulmonaria officinalis* L. The term “yunnaneic acids” is a collective name of several oligomeric derivatives of caffeic and rosmarinic acids, originally identified in *Salvia yunnanesis*. Yunnaneic acid B (C_54_H_46_O_25_) is a dimer of yunnaneic acid C, (being a complex of caffeic acid and the Diels–Alder adduct of rosmarinic acid). Contrary to numerous studies on biological activities of caffeic and rosmarinic acids, physiological effects of yunnaneic acid(s), including their activity in the cardiovascular system, have not been described yet.

**Materials and methods:** The methanol extract from aerial parts of *P. officinalis* L. was investigated for phytochemical constituents. It was purified in a stepwise manner by different chromatographic methods. The extract was first applied to preconditioned RP-C18 column (80 × 100 mm, Cosmosil 140 C_18_-PREP, 140 µm), followed by removal of polar constituents (like sugars and simple organic acids), while flavonoids and other phenolic compounds were eluted with 50% methanol. This fraction was directed to further purification by low-pressure chromatography on Sephadex LH-20 column (48 × 400 mm), as well as reversed phase column (32 × 300 mm, Cosmosil 40 C_18_-PREP, 40 µm), followed by semi-preparative RP-HPLC (10 × 250 mm, Atlantis T3 Prep OBD, 5 µm). The chromatographic separation yielded a cream-colored substance, subsequently identified by means of HR-QTOF-MS/MS and NMR techniques and comparison with literature data [1] as yunnaneic acid B. Measurements of 1,1-diphenyl-2-picrylhydrazyl (DPPH^•^) reduction and H_2_O_2_-scavenging efficacy indicated on considerable antioxidant potency of yunnaneic acid B. At its concentrations ≤10 µg/ml, about 69% of the radical reduction and 35% of H_2_O_2_ scavenging were observed. Antioxidant action of yunnaneic acid B (1-50 µg/mL) was also evaluated using a biological experimental system of blood plasma in vitro, exposed to 100–200 μM peroxynitrite-induced oxidative stress.

**Results:** Yunnaneic acid B effectively diminished oxidative damage of blood plasma lipids (up to 60 and 90% inhibition of lipid hydroperoxides and thiobarbituric acid-reactive substances generation, respectively). Furthermore, under oxidative stress, it was able to prevent the peroxynitrite-induced decrease of non-enzymatic antioxidant capacity of blood plasma (also measured with the use of DPPH^•^ radical), and even, to strengthen the antioxidant potential of blood plasma (by about 25%, when compared to control/untreated blood plasma).

**Conclusions:** This is the first study confirming the presence of yunnaneic acid B in *P. officinalis* as well as in the Boraginaceae family. The examined acid significantly reduced effects of peroxynitrite-induced oxidant stress and enhanced antioxidant potential of blood plasma in vitro.

**Acknowledgements:** The work was supported by grants from the national Science Centre (UMO-2013/11/D/NZ9/02771) and University of Lodz, Poland (506/1136).

**Reference**

1. Tanaka T, Nishimura A, Kouno I, et al. J Nat Prod. 1996;59:843–9.

## 84 Hypoglycemic and prebiotic properties of a polysaccharide from *Sargassum thunbergii*

### Beibei Ren^1^, Xiong Fu^1,2^, Chun Chen^1^, Chao Li^1,2^

#### ^1^School of Food Science and Engineering, South China University of Technology, Guangzhou 510640, China; ^2^Guangdong Province Key Laboratory for Green Processing of Natural Products and Product Safety, Guangzhou 510640, China

##### **Correspondence:** Chao Li - felichao@scut.edu.cn

*Journal of Chinese Medicine* 2018, **13(Suppl 1):**84

**Background:** *Sargassum thunbergii*, which belongs to the family of *Sargassum*, has been traditionally used as an edible and medicinal seaweed in China for a long time [1]. The polysaccharides extracted from *S. thunbergii* have been found to exhibit versatile bioactivities, such as antioxidant, immunomodulatory, and antitumor activities [2]. In recent years, considerable research has shown that non-digestible polysaccharides conferred prebiotic effects by modulating the intestinal health [3]. Up to now, little information is available on the hypoglycemic and prebiotic functions of polysaccharides from *S. pallidum* (SPP). Therefore, the aim of the study was to investigate the preliminary structure, hypoglycemic and prebiotic properties of SPP.

**Results:** A novel polysaccharide named ST-P2 was isolated from *S. pallidum* by hot water extraction and DEAE Sepharose fast flow column purification. Chemical composition analysis indicated that ST-P2 contained 48.3% of total carbohydrate, 0.33% of protein, and 3.47% of sulfates. Structural analysis showed that ST-P2 was homogeneous with molecular weight (Mw) of 48788 Da, and consisted of Ara, Gal, Glc, Xyl, Man, GalA, and GlcA with molar percentages of 3.93, 6.21, 3.19, 15.6, 14.8, 40.6, and 15.6%, respectively. The main glycosidic linkages included (1 → 6) or (1 →) glycosidic linkages (37.8%), (1 → 2) or (1 → 4) glycosidic linkages (25.7%) and (1 → 3) glycosidic linkages (36.5%). AFM analysis showed that ST-P2 exhibited a short chain conformation with branched structure. The results of in vitro hypoglycemic assay showed that ST-P2 exhibited effective inhibitory effect on α-glucosidase α-glucosidase activity, and could significantly promote pancreatic cell proliferation and insulin secretion of RIN-m5f cells [4]. In addition, in vitro fermentation assay showed that ST-P2 resulted in pH decline of fecal culture and a significant increase in the production of total short chain fatty acids (SCFAs), and especially acetic, propionic, n-butyric and n-valeric acids. Furthermore, ST-P2 significantly enhanced beneficial *Bacteroidetes* population, and inhibited the harmful *Firmicutes* population [5].

**Conclusions:** Overall, these results suggest that ST-P2 could potentially be exploixed as a novel hypoglycemic and prebiotic ingredient in functional food and pharmacological fields.

**References**

1. Luo D, Yuan X, Zeng Y, Nie K, Li Z, Wang Z. Structure elucidation of a major fucopyranose-rich heteropolysaccharide (STP-II) from *Sargassum thunbergii*. Carbohyd Polym. 2016;143:1–8.
2. Ren B, Chen C, Li C, Fu X, You L, Liu RH. Optimization of microwave-assisted extraction of *Sargassum thunbergii* polysaccharides and its antioxidant and hypoglycemic activities. Carbohyd Polym. 2016;173:192–201.
3. Shang Q, Shan X, Cai C, Hao J, Li G, Yu G. Dietary fucoidan modulates the gut microbiota in mice by increasing the abundance of *Lactobacillus* and *Ruminococcaceae*. Food Funct. 2016;7:3224–32.
4. Zhu J, Liu W, Yu J, Zou S, Wang J, Yao W, Gao X. Characterization and hypoglycemic effect of a polysaccharide extracted from the fruit of *Lycium barbarum* L. Carbohyd Polym. 2016;98(1):8–16.
5. Ma G, Kimatu B, Zhao L, Yang W, Pei F, Hu Q. In vivo fermentation of a *Pleurotus eryngii* polysaccharide and its effects on fecal microbiota composition and immune response. Food Funct. 2017;8:1810-21.

## 85 Optimizing conditions of extracting tannin and protein from grape seed

### Jun Li

#### College of Food Science and Technology, Hebei Normal University of Science and Technology, Qinhuangdao 066600, China

##### **Correspondence:** Jun Li

*Journal of Chinese Medicine* 2018, **13(Suppl 1):**85

**Background:** The wine-making industries produce millions of tons of residues (grape pomace) after fermentation, which represents a waste management issue both ecologically and economically [1]. In the process of grape juicing and brewing red wine will generate a lot of byproducts such as grape seeds and grape skins [2]. These byproducts are rich in bioactive phytochemicals and dietary fibers. The tannin and protein of byproducts contains a very high nutritional value and keeping health function.

**Materials and methods:** This study discusses the method of the organic solvent extraction to extract tannins and alkali fusion protein extraction process.

**Results:** The experimental results showed that: the best conditions of extracting tannins from grape seed are: the volume fraction of ethanol is 51.70%, the extraction time is 3.08 h, the extraction temperature 61.88 °C. Under this conditions the extraction rate of tannin is up to 6.15%; the best conditions of extracting protein from grape seed are: the extraction time is 48.02 min, the extraction temperature is 60.89 °C, and the solid–liquid ratio is 1:32. Under this conditions the extraction rate of protein is up to 3.24%.

**Conclusions:** This method could be useful to the development of industrial extraction processes.

**References**

1. Fontana AR, Antoniolli A, Bottini R. J Agric Food Chem. 2013;61:8987–9003
2. Zhu FM, Du B, Zheng LH, et al. Food Chem. 2015;186:207–12.

## 86 Isolation and extraction of compounds from *Stenoloma chusanum* (*Linn*.) *Ching*

### Tianyun Li^1^, Jianguo Cao^1^, Guozheng Huang^1,2^

#### ^1^College of Life and Environmental Sciences, Shanghai Normal University, Shanghai 201418, China; ^2^Key Laboratory of Plant Resources and Chemistry of Arid Zone and State Key Laboratory Basis of Xinjiang Indigenous Medicinal Plants Resource Utilization, Xinjiang Technical Institute of Physics and Chemistry, Chinese Academy of Sciences, Urumqi 830011, China

##### **Correspondence:** Jianguo Cao - cao101@shnu.edu.cn

*Journal of Chinese Medicine* 2018, **13(Suppl 1):**86

**Background:** *Stenoloma chusanum* (L.) Ching is known as a traditional Chinese medicinal fern. It has been reported for its high flavonoid content, strong antioxidant potentials, and biological activities including detoxification, hemostasis, antibacterial and anti-cancer effects, etc [1]. However, the previous works mainly focus on the crude extract and there are few data on the exact components of *S. chusanum* and their intrinsical biological activities. Hence, the purpose of this work is to identify its chemical components and isolate the active compounds and explore their biological activities.

**Materials and methods:** The 80% ethanol extract of *S. chusanum* (L.) Ching (7.8 kg) was extracted sequentially using petroleum ether, ethyl acetate, dichloromethane and *n*-butyl alcohol. The ethyl acetate extract was isolated via chromatography eluting with hexane and ethyl acetate in a gradient concentration. Pure compounds were obtained by further purification of silica gel column chromatography, and/or crystallization, medium pressure preparative liquid chromatography (MPLC) and high-speed counter-current chromatography (HSCCC).

**Results:** Five compounds were obtained and their structures were identified by means of chemical evidence, spectral analysis (‘H-NMR, ^13^C-NMR). They are confirmed as vanillic acid, syringic acid, *p*-hydroxybenzoic acid, ethyl caffeate and protocatechualdehyde. Among the five compounds, cinnamic acid and ethyl caffeate were isolated from the plants of *S. chusanum* for the first time.

**Conclusions:** MPLC and HSCCC are effective methods to separate the active compounds from the medicinal fern *S. chusanum*.

**Funding:** Financial support from Natural Science Foundation of Shanghai Normal University (No. DYL201702) was appreciated.

**Reference**

1. Luo Y, Xiao X, Wang Z. J Chem Res Appl. 2009;1:20.

## 87 Structure-affinity relationship of flavonoids in lotus leaf (*Nelumbo nucifera* Gaertn.) on binding to serum albumin

### Jing Chen, Aiping Xiao, Liangliang Liu

#### Institute of Bast Fiber Crops, Chinese Academy of Agricultural Sciences, Changsha 410205, China

##### **Correspondence:** Liangliang Liu - liuliangliang@caas.cn

*Journal of Chinese Medicine* 2018, **13(Suppl 1):**87

**Background:** The structural differences of flavonoids significantly affect their absorption, metabolism and activity [1]. As a widely distributed plant, the lotus leaf was becoming popular as a kind of drink like tea especially in herbal formulations [2]. Flavonoids are main functional components of lotus leaf and many of them was identified including isoquercetin, hyperin, kaempferol, astragalin, myricetin and so on [3–5].

**Materials and methods:** The interactions between flavonoids in lotus leaf and two kinds of serum albumin (human serum albumin and bovine serum albumin, HSA and BSA) and the DPPH free radical scavenging activities were carried out by spectroscopic methods. Eleven flavonoids reported existing in lotus leaf were selected as the research samples. And the relationship between the molecular properties of flavonoids and their affinities for HSA and BSA were also analyzed.

**Results:** It showed that the hydroxylation might decrease or increase the affinities for HSA and BSA depending on the conjunction sites. The methoxylation on 3′ position might also decrease the affinities for HSA and BSA. The glycosylation decreases the DPPH free radical scavenging activities of flavonoids and lowers the affinities for HSA and BSA depending on the type of sugar moiety. The hydrogenation of the C_2_-C_3_ double bond of apigenin and quercetin decreases both the affinities for HSA and BSA and DPPH activities.

**Conclusion:** The molecular property–affinity relationship reveals that the hydrogen bond force plays an important role in binding flavonoids to HSA and BSA. The DPPH activity generally increase with the increasing affinities of flavonoids for serum albumins (Fig. 1).

**Fig. 1:**
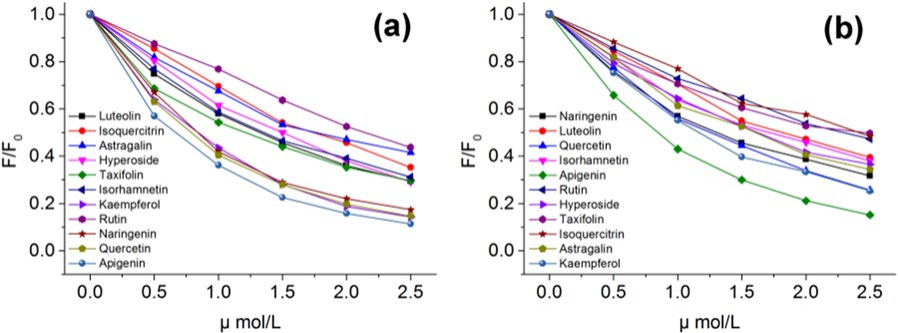
The quenching ratio (F/F_0_) of (**a**) HSA and (**b**) BSA fluorescences with addition of apigenin, naringenin, luteolin, quercetin, taxifolin, isorhamnetin, hyperoside, kaempferol, astragalin, isoquercitrin and rutin

**References**

1. Xiao JB, Cao H, Wang YF, et al. Mol Nutr Food Res. 2010;54:S253–60.
2. Zhu YT, Jia YW, Liu YM, et al. J Agric Food Chem. 2014;62:10679–86.
3. Tao Y, Zhang YF, Wang Y, et al. Anal Chim Acta. 2013;785:75–81.
4. Ye LH, He XX, Yan MZ, et al. Anal Methods. 2014;6:6088–9604.
5. Chen S, Wu BH, Fang JB, et al. J Chromatogr A. 2012;1227:145–53.

## 88 Grains of paradise intake improves antioxidant status of type 2 diabetes model of rats

### Aminu Mohammed^1,2^, Md. Shahidul Islam^1^

#### ^1^Department of Biochemistry, Faculty of Life Science, Ahmadu Bello University, Zaria, Nigeria; ^2^Department of Biochemistry, School of Life Sciences, University of KwaZulu-Natal, (Westville Campus), Durban, 4000, South Africa

##### **Correspondence:** Md. Shahidul Islam

*Journal of Chinese Medicine* 2018, **13(Suppl 1):**88

**Background:** Grains of paradise (*Aframomum melegueta* K. Schum) has been a popularly used spice in most of African food preparation. Our previous study showed that ethyl acetate fraction from crude ethanolic extract inhibited α-amylase and α-glucosidase actions, improved pancreatic β-cell damage and ameliorated insulin resistance in diabetic rats [1]. Additionally, 6-Gingerol, 6-shogaol, 6-paradol and oleanolic acid are shown to be the compounds responsible for the antidiabetic action of Grains of paradise [2]. However, detail antioxidant potential of this spice in diabetic animal model has not yet been reported. Thus, the present study investigates the effect of oral consumption of Grains of paradise fruit on the in vivo antioxidant status of type 2 diabetes (T2D) model of rats.

**Materials and methods:** The extraction and subsequent fractionation was carried out according to the method as reported previously [3]. T2D was induced in rats by feeding a 10% fructose solution ad libitum for 2 weeks followed by a single intraperitoneal injection of streptozotocin (40 mg/kg body weight (bw)). The animals were orally administered with 150 (DGPL) or 300 mg/kg bw (DGPH) of the fraction once daily for 4 weeks. Data were analyzed by using a statistical software package (SPSS for Win-dows, version 22, IBM Corporation, NY, USA) using Tukey’s-HSD multiple range post hoc test. Values were considered significantly different at p < 0.05.

**Results:** After 4 weeks of intervention, diabetic untreated animals showed significantly (p < 0.05) elevation of blood glucose levels (Fig. 1). The levels of thiobarbituric acid reactive substances (TBARS) were observed to increase with concomitant reduction of reduced glutathione (GSH) levels in the serum and organs (liver, kidney, heart and pancreas) of diabetic untreated animals. The activities of endogenous antioxidant enzymes (superoxide dismutase, catalase, glutathione peroxidase and reductase) were greatly reduced in the serum and organs of diabetic untreated animals compared to the normal animals (Fig. 2). These alterations were reverted to near-normal after the intake of Grains of paradise fruit in the treated groups (DGPL & DGPH) within the study period, especially at the dose of 300 mg/kg bw. This potent antioxidant action may partly be attributed to the presence of the 6-Gingerol, 6-shogaol and 6-paradol are known to be potent antioxidant [4].

**Fig. 1:**
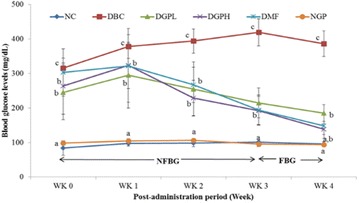
Weekly serum blood glucose levels

**Fig. 2:**
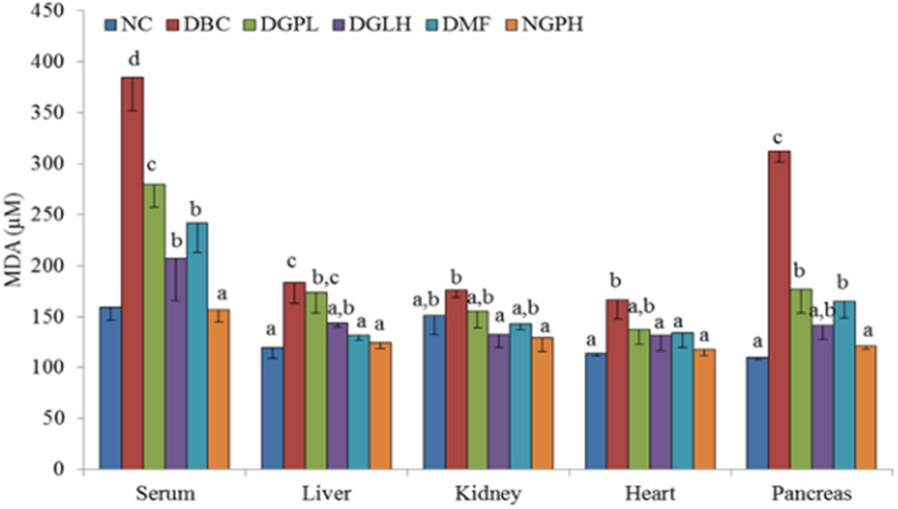
Levels of serum and tissues antioxidant parameters

**Conclusions:** The results of our study showed that Grains of paradise intake improved the antioxidant status of a T2D rats and therefore could be used to ameliorate diabetes-induced oxidative damage.

**References**

1. Mohammed A, Koorbanally NA, Islam MS. J Ethnopharmacol. 2015;175:518–27.
2. Mohammed A, Awolola GV, Koorbanally NA, et al. Pharm Biol. 2017, Accepted.
3. Ibrahim MA, Islam MS. J Ethnopharmacol. 2014;154:832–8.
4. Semwal RB, Semwal DK, Combrinck S, et al. Phytochemistry. 2015;117:554–68.

## 89 Plasma protein binding rates of dietary flavonoids to human serum albumin: a high performance affinity chromatography approach

### Xiaojuan Liu^1^, Hui Cao^2, 3^, Jianbo Xiao^2^

#### ^1^College of Life and Environment Sciences, Shanghai Normal University, Shanghai 200234, China; ^2^Institute of Chinese Medical Sciences, State Key Laboratory of Quality Research in Chinese Medicine, University of Macau, Taipa, Macau

##### **Correspondence:** Jianbo Xiao

*Journal of Chinese Medicine* 2018, **13(Suppl 1):**89

**Background:** The plasma protein binding (PPB) is an unavoidable process after a drug being distributed in circulating blood. PPB rate is a thermodynamic value which is measured the binding percentage in the steady state [1]. The structure-affinity relationship of polyphenols binding to human serum albumin (HSA) had been widely reported. Previous research mainly focused on the combining strength of the structural properties of selected dietary flavonoids and HSA. However, few articles have paid close attention to the relationship between flavonoids’ structure and their PPB affinity. Herein, we elucidated the protein binding of selected flavonoids and chose high performance affinity chromatography to determine the PPB affinities of flavonoids to HSA.

**Materials and methods:** All the flavonoids standard of different structures were dissolved with chromatographic grade of dimethyl sulfoxide. The molarity of each standard is 10^−3^ M. All the flavonoids standard were stored in low temperature refrigerator for use. The 50 mM potassium phosphate buffer (pH 7.0) consisted of 25 mM KH_2_PO_4_ and 25 mM K_2_HPO_4_. MilliQ water was used to dissolve the buffer and phosphoric acid was used to adjust the value of pH. Degassing the buffer 15 min for use. HPAC was performed by using a Thermo Fisher HPLC (Thermo Fisher Scientific, USA) with a 1525 binary pump, a 717 plus autosampler, a 2487 dual wavelength absorbance detector set at the detection wavelength of 210/270/280/360 nm respectively. Data collection and integration were accomplished by using Chameleon software version 7.1. Analysis was performed on a CHIRAL-HSA column (150 × 4 nm I.D., 5.0 μm particle size; Daicel chiral Tech Co., Ltd., Japan).

**Results:** The flavonoids with hydroxyl on ring A showed a higher PPB affinity compared those without hydroxyl on ring A. However, the hydroxylation of ring B lowered the PPB affinity. It was found that an additional methoxy group in ring A of flavones could decrease PPB affinity. Nevertheless, the methoxy group in ring A (position 6) of flavanone and ring B (position 4′) of isoflavone increased the PPB affinity. Methyl group at other positions of flavonoids slightly enhanced or little affected the PPB affinity. Hydrogenation of C_2_=C_3_ double bond and glycosylation decreased the PPB affinity.

**Conclusions:** In contrast, we found that the flavonoids with different structures their protein binding rates were also different.

**Reference**

1. Kurlbaum M, Hogger P. J Pharmaceut Biomed. 2011;54:127–32.

## 90 A PPAR-γ agonistic phthalimide analogue exerts in vitro and in vivo anti-inflammatory effects

### Mingzhi Su, Jiafu Cao, Jin Huang, Sen Liu, Dongsoon Im, JinwookYoo, Jee H. Jung

#### College of Pharmacy, Pusan National University, Busan, Republic of Korea

##### **Correspondence:** Jee H. Jung - jhjung@pusan.ac.kr

*Journal of Chinese Medicine* 2018, **13(Suppl 1):**90

**Background:** Previously, we have synthesized a phthalimide analogue 4-hydroxy-2-(4-hydroxyphenethyl) isoindoline-1,3-dione (PD1), which showed good PPAR-γ agonist activity [1, 2]. Since one of the functions of PPAR-γ is suppression of inflammatory responses, the present study aimed to investigate anti-inflammatory activity of PD1.

**Methods:** Transcriptions of mRNA were determined by reverse transcriptase-PCR. Inflammatory protein expressions were determined by ELISA and western blot method.

**Results:** In lipopolysaccharide(LPS)-stimulated marine macrophage RAW264.7 cells, PD1 suppressed the induction of pro-inflammatory factors including inducible nitric oxide synthase (iNOS), nitric oxide (NO), cyclooxygenase 2 (COX-2), tumor necrosis factor α (TNF-α), interleukine 1β (IL-1β), and interleukine 6 (IL-6) in both mRNA level and protein level. In parallel, PD1 enhanced expression of anti-inflammatory factors such as arginase-1 and interleukine 10 (IL-10). PD1 simultaneously suppressed LPS-evoked nuclear factor kappa B (NF-κB) p65 subunit phosphorylation in macrophages. The anti-inflammatory mechanism of PD1 is proposed to be the inhibition of NF-κB pathway. In subsequent in vivo animal experiment employing carrageenan-induced acute inflammatory paw edema model, PD1 showed significant reduction in paw swelling. Histological analysis of tissue sections revealed reduction of cellular infiltration of immune cells in PD1-treated groups.

**Conclusions:** These findings suggest that PD1 may serve as a lead for anti-inflammatory therapeutics.

**References**

1. Xiao B, Su M, Kim E L, Hong J, Chung H Y, Kim H S, Yin J, Jung J H. Synthesis of PPAR-γ activators inspired by the marine natural product, paecilocin A. Mar Drugs. 2014;12:926–39.
2. Eom SH, Liu S, Su M, Noh TH, Hong J, Kim ND, Chung HY, Yang MH, Jung JH. Synthesis of phthalimide derivatives as potential PPAR-γ Ligands. Mar Drugs. 2016;14:112–22.

## 91 Study of regulation mechanism of *Ganoderma* triterpenes on lipid metabolism in high-fat-diet induced Wistar rats

### Ruibo Jia^1^, Zirui Huang^1^, Yuyang Pan^1^, Tiantian Li^1^, Xucong Lv^1,2^, Bin Liu^1,2^

#### ^1^College of Food Science, Fujian Agriculture and Forestry University, Fuzhou, Fujian 350002, China; ^2^National Engineering Research Center of JUNCAO Technology, Fujian Agriculture and Forestry University, Fuzhou, Fujian 350002, China

##### **Correspondence:** Bin Liu - liubin618@hotmail.com

*Journal of Chinese Medicine* 2018, **13(Suppl 1):**91

**Background:** Among the many biologically active constituents of *Ganoderma*, the triterpenoids have been shown to have hypoglycemic effects, enhanced immunity, liver protection, anti-cancer, hypolipidemic and health.

**Materials and methods:** The regulation mechanism of *Ganoderma* triterpenes (GP) on lipid metabolism was studied in wistar rats with the disturbance in the lipid metabolism. The effects of GP on gut microbiota composition was analyzed by 16S rRNA(V3–V4 region) high-throughput sequencing (HTS) in caecal faeces and regulation mechanism on the lipid metabolism in rats with high-fat and high-carbohydrate diet was elucidated by the liver tissue transcriptase sequencing analysis based on the Illumina high-throughput sequencing platform.

**Results:** The weight of Wistar rats fed with 8 weeks of high-fat diet was significantly higher than the control group (NFD) (p < 0.05), The weight gain of different doses of GP group (GP50, GP100, GP150) were significantly slower than model group (HFD) (P < 0.01). the animal serum levels of total cholesterol (TC), triglyceride (TG), low density lipoprotein cholesterol (LDL-c), glutamate pyruvate transaminase (ALT), glutamic oxalacetic transaminase (AST) and free fatty acid (FFA) in GP150 was significantly lower than those the HFD, and high density lipoprotein cholesterol (HDL-C) was higher than that in HFD. Antioxidant enzymes in liver tissue of experimental rats were analyzed, the levels of methylenedioxyamphetamine (MDA) of GP50, GP100 and GP150 group were significantly decreased respectively compared with the HFD (P < 0.01), glutathion peroxidase (GSH-PX) and superoxide dismutase (SOD) in GP150 were significantly increased (P < 0.01). High-fat diet can lead to significantly increase the abundance of *Akkermansia*, *Lactobacillus, Nosocomiicoccus, Odoribacter, Oligella* and *Anaeroplasma*. On the other hand, decreased in the abundance of *Collinsella, Enterococcus, Desulfovibrio* and so on. The liver tissue of each experimental group was subjected to transcriptome sequencing analysis based on Illumina high-throughput sequencing platform, and the differentially expressed genes were analyzed by GO enrichment and KEGG enrichment. It was found that the differentially expressed genes were mainly enriched in PPAR signaling pathway, Biosynthesis of amino acids, Non-alcoholic fatty liver disease, Type II diabetes mellitus and AMPK signaling pathway compared with the model group. The differentially expressed genes closely related to lipid metabolism mainly include Gk2, MEF, Scd1, IRS1IRM, SREBP-1c, PKL, Jun, IRS1IRM, Tkfc, PKL, Gadd45, enoyl -CaA hydratase, Hadhsc, Cyp2c70, Acaa2, Cyp4a11, RT1-A, Ins1, HRSL3, Phlpb, Fadsd6, Muscpho, Xbp1 and pe-CoA compared with the model group, these differentially expressed genes play a vital role in the corresponding signaling pathways, and regulate the body’s lipid synthesis transport.

**Conclusions:** The *Ganoderma* triterpenes can effective in reducing the serum levels, the body weight, gut microbiota composition and genes expression, which closely related to lipid metabolism. The results provided the theoretical basis for the application of GP in functional food and drugs of the regulation of lipid metabolism.

## 92 Total active ingredients extracted from *Spirulina* and their effect on lipid metabolism in high-fat diet induced Wistar rats

### Zirui Huang^1^, Yuyang Pan^1^, Ruibo Jia^1^, Hongpei Chen^1^, Tiantian Li^1^, Bin Liu^1,2^

#### ^1^College of Food Science, Fujian Agriculture and Forestry University, Fuzhou, Fujian 350002, China; ^2^National Engineering Research Center of JUNCAO Technology, Fujian Agriculture and Forestry University, Fuzhou, Fujian 350002, China

##### **Correspondence:** Bin Liu - liubin618@hotmail.com

*Journal of Chinese Medicine* 2018, **13(Suppl 1):**92

**Background:** Among the many biologically active constituents of *spirulina*, the polysaccharides, proteoglycans, proteins and triterpenoids have been shown to have hypoglycemic effects, enhanced immunity, anti-aging and health.

**Materials and methods:** This study aimed to evaluate the lipid-lowering effect of extract of *spirulina* by 95% ethanol, 55% ethanol, water, enzymolysis and above mixture, called total active ingredients (TAI). The effects of TAI on lipid metabolism were studied on Wistar rats induced by high-fat diet, The effects of *spirulina*’s extracts on gut microbiota composition was analyzed by 16S rRNA (V3–V4 region) high-throughput sequencing (HTS) in caecal faeces.

**Results:** The weight growth of TAI groups were significantly slower than model (HFD) group(P < 0.01), which indicating that the TAI could effectively control the body weight of HFD rats. The animal serum (liver) levels of triglyceride (TG), total cholesterol (TC), glutamate pyruvate transaminase (ALT), glutamic oxalacetic transaminase (AST) and free fatty acid (FFA) in TAI groups were significantly lower than those in HFD group, and (HDL-C) was higher than that in HFD group. The effects of TAI on gut microbiota composition were analyzed on the faecal and intestinal contents flora in each group by 16S rRNA high-throughput sequencing (HTS), the results showed that *Firmicutes*-to-*Bacteroidetes* ratio of HFD group was significantly higher than normal-food diet (NFD) group, *Firmicutes*-to-*Bacteroidetes* ratio with gavage TAI were lower compared with HFD group. In Genus, HFD can lead to significantly increase the abundance of *Anaerofustis*, *Blautia*, *Clostridium_XVIII*, *Holdemania*; on the other hand, decreased in the abundance of *Nosocomiicoccus, Oligella* and so on.

**Conclusions:** The results showed that TAI had a certain effect on the balance of intestinal microecology in HFD rats. This study demonstrated that different polarity extracts of *spirulin*a could have beneficial effects on lipid metabolism in HFD induced Wistar rats. TAI might play an interesting role in the prevention of lipid metabolism disorders and could be used as a natural ingredient or supplement for functional food preparation.

## 93 Polysaccharide purified from *Ganoderma atrum* induced activation and maturation of murine myeloid-derived dendritic cells

### Hui Wang^1^, Qiang Yu^1^, Shaoping Nie^1^, Quandan Xiang^1^, Mingming Zhao^1^, Shiyu Liu^1^, Mingyong Xie^1^, Shunqi Wang^2^

#### ^1^State Key Laboratory of Food Science and Technology, Nanchang University, Nanchang 330047, China; ^2^Institute of Life Science & College of Life Sciences, Nanchang University, Nanchang 330047, China

##### **Correspondence:** Qiang Yu - yuqiang8612@163.com; Mingyong Xie - myxie@ncu.edu.cn

*Journal of Chinese Medicine* 2018, **13(Suppl 1):**93

**Background:** *Ganoderma atrum*, a member of the genus *Ganoderma*, is an edible and medicinal fungus. The polysaccharide is regarded as the major bioactive substances in *G. atrum*. *G. atrum* polysaccharide (PSG-1) has been discovered many bioactivities. However, the effect of PSG-1 on dendritic cells (DCs) is still not clear. It is necessary to understand the bioactivity of PSG-1 on DCs. This study aimed to reveal the direct stimulation activity and indirect effect of PSG-1 on DCs.

**Materials and methods:** DCs and T cells were generated from bone marrow cells and separated from spleen respectively. Flow cytometric, ELISA analysis and mixed lymphocyte reaction (MLR) were adopted to analysis cell surface molecule expression, cytokines production and the ability of DCs to promote proliferation of splenic T lymphocyte of mouse, respectively. Western blot analysis was employed to investigate the phosphorylation of p38, ERK and JNK. The indirect effect on DCs was studied by using a DCs-Caco-2 co-culture model in vitro.

**Results:** The direct stimulation studies indicated that PSG-1 could increase cell surface molecule expression of MHC-II, CD80 and CD86, enhance the production of IL-12 p70, IL-6, IL-10, RANTES, MIP-1α and MCP-1 in DCs, improve the ability of DCs promoting proliferation of splenic T lymphocyte of mouse and increase the phosphorylation of p38, ERK and JNK in DCs. In addition, p38, ERK and JNK are all crucial in PSG-1-induced expression of MHC-II, CD80 and CD86 and production of IL-6 and IL-10 by DCs. Furthermore, the indirect effect study showed that PSG-1 could increase expression of MHC-II of DCs in the DCs-Caco-2 co-culture model.

**Conclusions:** We conclude that PSG-1 directly induces expression of maturation markers and cytokines of DCs via MAPK pathways, and indirectly stimulates DCs separated by intestinal epithelial cells.

**Note:** This article named “Polysaccharide purified from *Ganoderma atrum* induced activation and maturation of murine myeloid-derived dendritic cells” has been included by the Food Chemical and Toxicology.

## 94 New polyacetylenes isolated from the roots of *Eurycoma longifolia*

### Minglong Wang^1^, Huijuan Zou^1^, Qibin Chen^2^, Jianguo Cao^1^*, Haji Abker Aisa^2^, Guozheng Huang^1,2^

#### ^1^College of Life and Environmental Sciences, Shanghai Normal University, Shanghai, 201418, P. R. China; ^2^Key Laboratory of Plant Resources and Chemistry of Arid Zone, Xinjiang Technical Institute of Physics and Chemistry, Chinese Academy of Sciences, Urumqi, 830011, P. R. China

##### **Correspondence:** Guozheng Huang

*Journal of Chinese Medicine* 2018, **13(Suppl 1):**94

**Background:** *Eurycoma longifolia* is a tropical plant widely distributed in Southeast Asia [1]. As a traditional herbal medicine, it is used to treat fever, fatigue, dysentery, etc. In the present study, we aimed to isolate natural products from the roots of *E. Longifolia* via high-speed counter-current chromatography (HSCCC), a highly efficient chromatographic technique widely used on isolation of phytochemicals [2].

**Materials and methods:** Around 30 g of methonalic extracts were obtained from 1 kg of dried root of *E. Longifolia*. The extracts were subjected to silica chromatography, eluting with petroleum ether with increased amount of ethyl acetate to provide 230 fractions. Among them, fractions 118–119 contained of polyacetylenes and were then subjected to HSCCC for further purification. A two-phase solvent system of hexane/ethyl acetate/methanol/water at a volume ratio of 5/2/5/2 was applied to separate a series of analogues. Then the whole mobile and stationary phase was blown out, concentrated, and subjected to second HSCCC purification with a solvent system at a volume ratio of 6/1/6/1.2.

**Results:** 19 mg of longifolione A, 9 mg of longifolione B and 317 mg of longifolione C were yielded in the first step. 5 mg of longifolione D and 33 mg of longifolione E were obtained in the second step. The structures of these five compounds were identified by 1D and 2D NMR, HRMS and specific rotation.

**Conclusions:** HSCCC method was applied to purify five new polysacetylenes from *E. longifolia*. All of these compounds are isolated from *E. longifolia* for the first time.

**Acknowledgements:** Financial support from Natural Science Foundation of Shanghai Normal University (No. DYL201702) and the Recruitment Program for Young Professionals of China were appreciated.

**References**

1. Rehman SU, Choe K, Yoo HH. Molecules. 2016;21:331.
2. Marston A, Hostettmann K. J Chromatogr A. 2006;1112:181.

## 95 Cyanidin-3-*O*-glucoside protects against 1,3-dichloro-2-propanol-induced reduction of progesterone by up-regulation of steroidogenic enzymes and cAMP level in Leydig cells

### Jianxia Sun^1,2^, Wei Xu^2^, Cuijuan Zhu^2^, Yunfeng Hu^2^, Xinwei Jiang^2^, Shiyi Ou^2^, Zhijian Su^2^, Yadong Huang^2^, Rui Jiao^2^, Weibin Bai^2^

#### ^1^Faculty of Chemical Engineering and Light Industry, Guangdong University of Technology, Guangzhou, China; ^2^Department of Food Science and Engineering, Institute of Food Safety and Nutrition, Jinan University, Guangzhou, China

##### **Correspondence:** Rui Jiao - tjiaorui@jnu.edu.cn; Weibin Bai - baiweibin@163.com

*Journal of Chinese Medicine* 2018, **13(Suppl 1):**95

**Background:** 1,3-Dichloro-2-propanol (1,3-DCP) is a food processing contaminant and has been shown to perturb male reproductive function [1]. Cyanidin-3-*O*-glucoside (C3G), a monotype of anthocyanins, is reported to reveal protective effects on various injuries [2]. However, it remains unclear whether C3G protects against 1,3-DCP-induced reproductive toxicity.

**Materials and methods:** C3G (HPLC, purity > 97%) was obtained from Biosynth AS (Sandnes, Norway). The present study was to investigate the intervention effect of C3G on 1,3-DCP-induced reproductive toxicity in R2C Leydig cells.

**Results:** C3G inhibited the 1,3-DCP-induced cytotoxicity and cell shape damage with the effective doses being ranging from 10 to 40 mmol/L. In addition, 1,3-DCP (2 mmol/L) exposure significantly increased the ROS level and mitochondrial membrane potential damage ratio, leading to a decrease in progesterone production, while C3G intervention reduced the ROS level, and increased the progesterone production after 24 h treatment. Most importantly, C3G intervention could up-regulate the cyclic adenosine monophosphate (cAMP) level and protein expression of steroidogenic acute regulatory protein and 3b-hydroxysteroid dehydrogenase.

**Conclusions:** C3G is effective to reduce 1,3-DCP-induced reproductive toxicity via activating steroidogenic enzymes and cAMP level.

**Funding:** This work is supported by National Natural Science Foundation of China (NSFC NO. 31471588). The authors also thank to the Program for New Century Excellent Talents in University (NCET) and Outstanding Young Teachers of the University in Guangdong Province (Yq2013024).

**Acknowledgements:** Chen Hongxia is appreciated for their help with western blotting, Xiao Xue for her help with SCGE, and Li Mingwei for the help with the statistical analysis.

**References**

1. Andres S, Appel KE, Lampen A. Toxicology, occurrence and risk characterisation of the chloropropanols in food: 2-monochloro-1,3-propanediol, 1,3-dichloro-2-propanol and 2,3-dichloro-1-propanol. Food Chem Toxicol. 2013;58:467–78.
2. He Y, et al. Cyanidin-3-*O*-glucoside inhibits the UVB-induced ROS/COX-2 pathway in HaCaT cells. J Photochem Photobiol B Biol. 2017;177(Supplement C):24–31.

## 96 Structure–activity relationship of dietary polyphenols as aldose reductase inhibitors

### Qianqian Yang^1^, Xiaojuan Liu^1^, Hui Cao^2^, Jianbo Xiao^2^, Guozheng Huang^1^

#### ^1^College of Life and Environmental Sciences, Shanghai Normal University, Shanghai, 201418, P. R. China; ^2^Institute of Chinese Medical Sciences, State Key Laboratory of Quality Research in Chinese Medicine, University of Macau, Avenida da Universidade, Taipa, Macau

##### **Correspondence:** Jianbo Xiao - jianboxiao@yahoo.com

*Journal of Chinese Medicine* 2018, **13(Suppl 1):**96

**Background:** Polyphenols are one of the most abundant antioxidants in human daily diets and are one of the most common, most universal second metabolites found in various tissues of plants [1]. Diabetes complications are mainly caused by the accumulation of sorbitol. Under the action of NADPH coenzyme, aldose reductase can catalyze the conversion of glucose to sorbitol [2]. Therefore, aldose reductase is a key enzyme in polyol metabolism, but also an important rate-limiting enzyme. Dietary polyphenols, as important aldose reductase inhibitors, have attracted the attention of scholars. Herein, the structure- activity relationship of dietary polyphenols as aldose reductase inhibitors was investigated [3].

**Materials and methods:** Human recombinant aldose reductase (HRAR) activity was measured following the method developed by Halder et al. [4]. The reaction mixture was mixed by the order as followed: 146 μL of 67 mM (pH 6.2) sodium phosphate buffer contained 0.4 M Li_2_SO_4_, 24 μL of different concentrations of DMSO and inhibitors, 20 μL of 3 mM NADPH, 25 μL of diluted HRAR. After it was incubated for 10 min in 37 °C, immediately, 10 μL of dl-glyceraldehyde as a substrate was added to start the reaction. The HRAR activity was examined by measuring the decrease of NADPH absorption at 340 nm, and the dynamic absorbance was recorded for 10 min at intervals of 30 s. The concentration of inhibitors represented by the half maximal inhibitory concentration (IC_50_) were calculated by the least-squares regression line that the concentration plotted against the residual activity.

**Conclusions:** (1) The methylation on C5, C3′, C4′ of flavones remarkably weakened the inhibition; the methylation on C6, C8 of flavones enhanced the inhibition. (2) The hydroxylation on C5, C6, C7 of flavones, notably at positions 5 and 6, observably enhanced the inhibition; the hydroxylation on C3′ of flavones remarkably weakened the inhibition. (3) The hydrogenation of the C2=C3 double bond of flavones weakened the inhibition. (4) The glycosylation of flavonoids at different positions show different influence on their inhibitory potential.

**References**

1. Xiao J, Ni X, Kai G, et al. Crit Rev Food Sci Nutr. 2015;55:16–31.
2. Yeonsil L, Seonha K, Sanghoon J, et al. Biol Pharmaceut Bull. 2010;33:917–21.
3. Park HY, Kim HK, Jeon SH, et al. App Biol Chem. 2009;52:493–7.
4. Halder N, Joshi S, Gupta SK. J Ethnopharmacol. 2003;86:113–6.

## 97 Structure–activity relationship of dietary polyphenols as aldose reductase inhibitors

### Qianqian Yang^1^, Xiaojuan Liu^1^, Hui Cao^2^, Jianbo Xiao^2^, Guozheng Huang^1^

#### ^1^College of Life and Environmental Sciences, Shanghai Normal University, Shanghai, 201418, P. R. China; ^2^Institute of Chinese Medical Sciences, State Key Laboratory of Quality Research in Chinese Medicine, University of Macau, Avenida da Universidade, Taipa, Macau

##### **Correspondence:** Jianbo Xiao - jianboxiao@yahoo.com

*Journal of Chinese Medicine* 2018, **13(Suppl 1):**97

**Background:** Polyphenols are one of the most abundant antioxidants in human daily diets and are one of the most common, most universal second metabolites found in various tissues of plants [1]. Diabetes complications are mainly caused by the accumulation of sorbitol. Under the action of NADPH coenzyme, aldose reductase can catalyze the conversion of glucose to sorbitol [2]. Therefore, aldose reductase is a key enzyme in polyol metabolism, but also an important rate-limiting enzyme. Dietary polyphenols, as important aldose reductase inhibitors, have attracted the attention of scholars. Herein, the structure- activity relationship of dietary polyphenols as aldose reductase inhibitors was investigated [3].

**Materials and methods:** Human recombinant aldose reductase (HRAR) activity was measured following the method developed by Halder et al. [4]. The reaction mixture was mixed by the order as followed: 146 μL of 67 mM (pH 6.2) sodium phosphate buffer contained 0.4 M Li_2_SO_4_, 24 μL of different concentrations of DMSO and inhibitors, 20 μL of 3 mM NADPH, 25 μL of diluted HRAR. After it was incubated for 10 min in 37 °C, immediately, 10 μL of DL-glyceraldehyde as a substrate was added to start the reaction. The HRAR activity was examined by measuring the decrease of NADPH absorption at 340 nm, and the dynamic absorbance was recorded for 10 min at intervals of 30 s. The concentration of inhibitors represented by the half maximal inhibitory concentration (IC_50_) was calculated by the least-squares regression line that the concentration plotted against the residual activity.

**Conclusions:** (1) The methylation on C5, C3′, C4′ of flavones remarkably weakened the inhibition; the methylation on C6, C8 of flavones enhanced the inhibition. (2) The hydroxylation on C5, C6, C7 of flavones, notably at positions 5 and 6, observably enhanced the inhibition; the hydroxylation on C3′ of flavones remarkably weakened the inhibition. (3) The hydrogenation of the C2=C3 double bond of flavones weakened the inhibition. (4) The glycosylation of flavonoids at different positions show different influence on their inhibitory potential.

**References**

1. Xiao J, Ni X, Kai G, et al. Crit Rev Food Sci Nutr. 2015;55:16–31.
2. Yeonsil L, Seonha K, Sanghoon J, et al. Biol Pharmaceut Bull. 2010;33:917–21.
3. Park HY, Kim HK, Jeon SH, et al. App Biol Chem. 2009;52:493–7.
4. Halder N, Joshi S, Gupta SK. J Ethnopharmacol. 2003;86:113–6.

## 98 Nuciferine inhibits proinflammatory cytokines via PPARs in LPS-induced RAW264.7 cells

### Chao Zhang^1,2^, Dan Liu^1^, Qingqing Liu^3^, Jianjun Deng^3^, Haixia Yang^1,2^

#### ^1^Nutrition and Food Safety Engineering Research Center of Shaanxi Province, College of Public Health, School of Medicine, Xi’an Jiaotong University, Xi’an, 710061, P. R. China; ^2^Cardiovascular Research Center, Xi’an Jiaotong University, Xi’an, 710061, P. R. China; ^3^Shaanxi Key laboratory of Degradable Biomedical Materials, College of Chemical Engineering, Northwest University, Xi’an, 710069, P. R. China

##### **Correspondence:** Haixia Yang

*Journal of Chinese Medicine* 2018, **13(Suppl 1):**98

**Background:** Inflammatory abnormalities are widely implicated in a vast variety of acute and chronic human disease processes. Regulation of inflammatory response relies on multiple potential mechanisms. Nuciferine is an aromatic ring-containing alkaloid found in the *Nelumbo nucifera* leaves, showing potential anti-inflammation activity, but the molecular mechanism of anti-inflammatory effect of nuciferine is still unclear. Thus, in this study, we investigated the anti-inflammatory effect and possible mechanisms of nuciferine in the RAW264.7 cells.

**Materials and methods:** MTT assay was performed to detect nuciferine cytotoxicity. The inflammatory model in vitro was made using RAW264.7 cells stimulated by lipopolysaccharide (LPS). Interleukin 6 (IL-6) and tumor necrosis factor α (TNFα) production was detected using enzyme-linked immunosorbent assay. The real-time polymerase chain reaction was employed to assess the expression of IL-6 and TNFα mRNA. The peroxisome proliferator activated receptors (PPARs) activity was studied by luciferase reporter assay. PPARs specific pharmacological agonists (WY14643/GW501516/rosiglitazone) and antagonists (GW6417/GSK0660/GW9662) were applied for mechanism study. The expression of indicated proteins I-κB was examined by Western blotting.

**Results:** Nuciferine significantly suppressed inflammatory cytokines production such as IL-6 and TNF-α in LPS-induced RAW264.7 cells. In addition, the luciferase assay results of three PPAR subtypes showed that all of the activities are promoted by nuciferine in a dose-dependent manner. Specific inhibitors of PPARα and PPARγ markedly abolished LPS-induced IL-6 and TNF-α production, even I-κB degradation. However, the specific inhibitor of PPARδ did not.

**Conclusions:** Our finding suggested a potential mechanism of the protective effects of nuciferine in LPS-induced inflammation via activating PPARα and PPARγ in RAW 264.7 cells

**Keywords:** Nuciferine, peroxisome proliferator-activated receptor, anti-inflammatory, macrophage

**Acknowledgements:** This study was supported by the National Natural Science Foundation of China (21676212 and 21476184).

## 99 A new cytotoxic biflavone from *Selaginella doederleinii*

### Zhen-Xing Zou^1^, Kang-Ping Xu^2^, Guo-Gang Zhang^1^, Ping-Sheng Xu^1^, Gui-Shan Tan^1^

#### ^1^Xiangya Hospital of Central South University, Changsha 410008, China; ^2^Xiangya School of Pharmaceutical Sciences, Central South University, Changsha 410013, China

##### **Correspondence:** Zhen-Xing Zou; Gui-Shan Tan

*Journal of Chinese Medicine* 2018, **13(Suppl 1):**99

**Background:** *Selaginella doederleinii*, belonging to the genus *Selaginella*, is widely distributed in Guangxi Zhuang Autonomous Region, Guizhou and Yunnan provinces of mainland China [1]. Traditionally, the whole plant has been used as a folk medicine to treat some kinds of cancers, sore throat and rheumatoid arthritis [2]. In our previous work, some unique flavonoids were reported in this plant [3–5]. As part of ongoing search for novel and bioactive flavones from this genus, 75% aqueous ethanol extract from the whole herbs of *Selaginella doederleinii* was isolated.

**Materials and methods:** The whole herbs of *S. doederleinii* were collected in the town of Wutong, Lingui district, Guangxi, China, in July 2013 and authenticated by Prof. ZhenJi Li (Xiamen University, Xiamen, China). A botanical specimen of this species (20130710) was deposited at the Xiangya School of Pharmaceutical Sciences, Central South University. Structures of **1**–**5** were determined by extensive spectroscopic methods including NMR and HRMS. All compounds were evaluated for their in vitro cytotoxicity against three human cancer cell lines A549, MCF-7, and SMMC-7721. The cytotoxicity assay was performed using MTT (3-(4, 5-dimethylthiazol-2-yl)-2, 5-diphenyltetrazolium bromide) method in 96-well microplates [6].

**Results**: A new biflavone, doederbiflavone A (**1**), together with four known ones (**2**–**5**) were isolated from *S. doederleinii* (Fig. 1). Compound **1** features a unique dimeric skeleton with apigenin and chrysin units, which was first discovered in nature, and exhibited significant cytotoxicity against A549 with IC_50_ value of 1.78 μM.

**Fig. 1:**
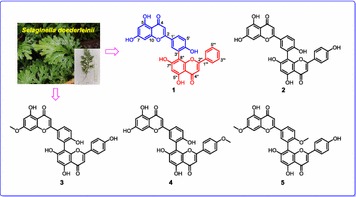
Structures of compounds **1**–**5**

**Conclusions:** Compound **1** may be a promising compound for treating lung cancer.

**References**

1. Nanjing University of Chinese Medicine, Zhong Yao Da Ci Dian. Shanghai: Shanghai Scientific & Technical Publishers; 2006. p. 831–2.
2. State Administration of Traditional Chinese Medicine, Zhong Hua Ben Cao. Shanghai: Shanghai Scientific & Technical Publishers; 1998. p. 44.
3. Zou ZX, Xu PS, Zhang GG, Cheng F, Chen K, Li J, Zhu WX, Cao DS, Xu KP, Tan GS. Selagintriflavonoids with BACE1 inhibitory activity from the fern *Selaginella doederleinii*. Phytochemistry. 2017;134:114–21.
4. Zou ZX, Xu KP, Xu PS, Li XM, Cheng F, Li J, Yu X, Cao DS, Li D, Zeng W, Zhang GG, Tan GS. Seladoeflavones A–F, six novel flavonoids from *Selaginella doederleinii*. Fitoterapia. 2017;116:66–71.
5. Zou ZX, Tan GS, Zhang GG, Yu X, Xu PS, Xu KP. New cytotoxic apigenin derivatives from *Selaginella doederleinii*. Chin Chem Lett. 2017;28:931–4.
6. Mosmann T. Rapid colorimetric assay for cellular growth and survival: application to proliferation and cytotoxicity assays. J Immunol Methods. 1983;65:55–63.

## 100 Anti-diabetic effects of *Inonotus obliquus* polysaccharides-chromium (III) complex in type 2 diabetic mice and its sub-acute toxicity evaluation in normal mice

### Cong Wang, Zhongqin Chen, Yuxiang Pan, Xudong Gao, Haixia Chen

#### School of Pharmaceutical Science and Technology, Tianjin University, Tianjin, China

##### **Correspondence:** Haixia Chen - chenhx@tju.edu.cn

*Journal of Chinese Medicine* 2018, **13(Suppl 1):**100

**Background:** The mushroom *Inonotus obliquus* (*I. obliquus*), a white rot fungus, has been used as a traditional folk remedy for centuries [1]. Polysaccharides from *I. obliquus* are known to be the main components with great biological activities [2]. Chromium is an essential mineral which is proved to overcome insulin resistance, ameliorate diabetes [3]. Thus, this study was aimed to synthesize a novel *I. obliquus* polysaccharides-chromium (III) complex (UIOPC) and characterize its physicochemical properties. The anti-diabetic effects of UIOPC in streptozotocin (STZ) induced type 2 diabetes mellitus (T2DM) mice and its sub-acute toxicity in normal mice were also well demonstrated.

**Materials and methods:** Polysaccharides were extracted and purified from sclerotia of *I. obliquus* by ultrafiltration method and then were used for the synthesis of UIOPC. Its physicochemical properties were characterized by HPGPC, GC, FTIR and CD, respectively. The anti-diabetic effects of UIOPC were investigated in high fat diet and STZ-induced T2DM mice. Besides, the sub-acute toxicity evaluation of UIOPC was conducted in normal mice.

Results: Results showed that the molecular weight of UIOPC was about 11.5 × 10^4^ Da with the chromium content of 13.01%. The chromium (III) was illustrated to link with polysaccharides through coordination bond, indicating the successful synthesis of UIOPC. After the successful establishment of T2DM model, the diabetic mice were treated with UIOPC for 4 weeks. It had been observed that the body weight, fasting blood glucose levels, plasma insulin levels of the diabetic mice were significantly reduced when compared with those of the un-treated diabetic mice (*P *< 0.05), suggesting the improved glucose homeostasis in T2DM mice [4]. The results on serum profiles and antioxidant enzymes activities revealed that UIOPC had a positive effect on hyperlipidemia and antioxidant ability. Histopathology results also showed that UIOPC could effectively alleviate the STZ-lesioned tissues in diabetic mice (Fig. 1A–C). Furthermore, high dose administration of UIOPC had no obvious influence on serum profiles levels and antioxidant ability of the normal mice. The organ tissues maintained organized and integrity in the sub-acute toxicity evaluation (Fig. 1D).

**Fig. 1:**
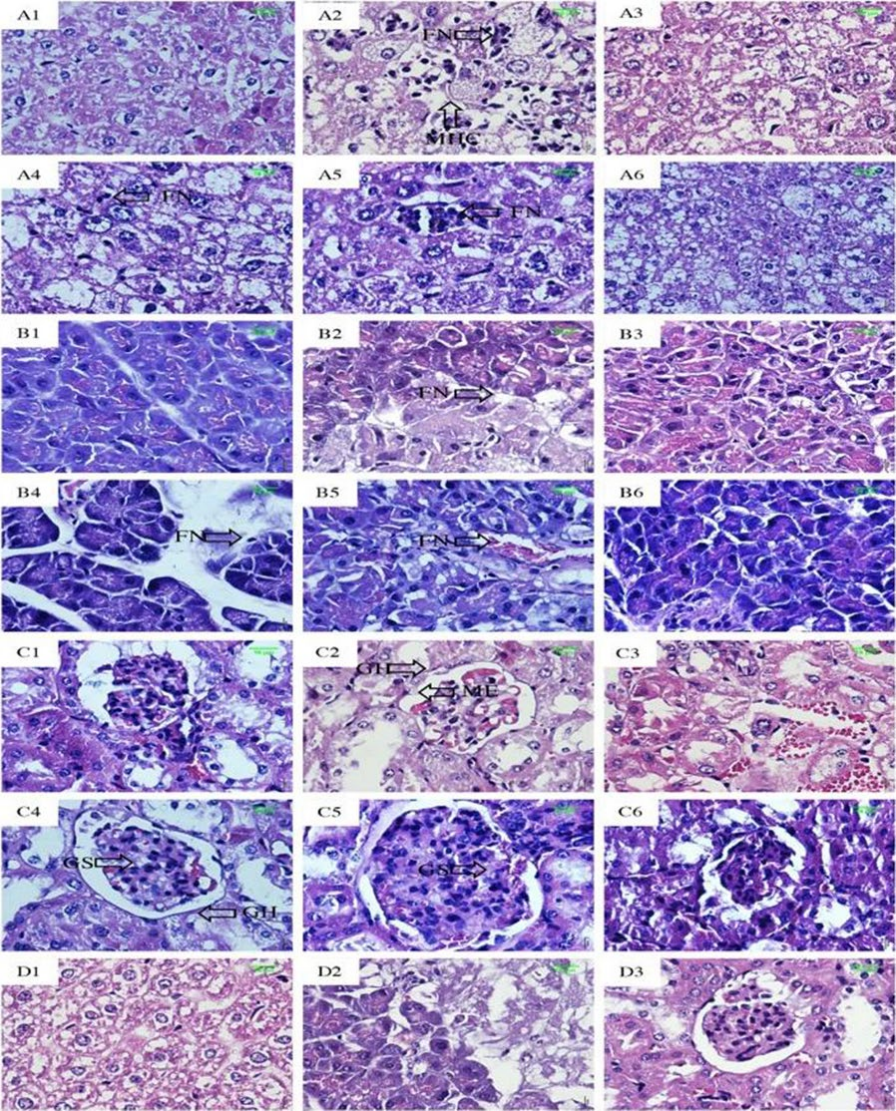
Effects of UIOPC on the treatment of liver, pancreas and kidney damages in STZ-induced diabetic mice and its toxicity evaluation in normal mice. **A** Liver; **B** Pancreas; **C** Kidney; **D** Toxicity evaluation

**Conclusions:** The antidiabetic and sub-acute toxicity evaluation results suggested that UIOPC might be a good candidate for the functional food or pharmaceutical in the treatment of T2DM.

**Acknowledgements:** This work was supported by the grant from the National Natural Science Foundation of China (NSFC 31371879) and National High Technology Research and Development Program (“863” Program) of China (Grant No. SS2013AA100207).

**References**

1. Zhang N, Chen H, Ma L, Zhang Y. Physical modifications of polysaccharide from *Inonotus obliquus* and the antioxidant properties. Int J Biol Macromol. 2013;54:209–15.
2. Ma L, Chen H, Dong P, Lu X. Anti-inflammatory and anticancer activities of extracts and compounds from the mushroom *Inonotus obliquus*. Food Chem. 2013;139:503–8.
3. Sushil KJ, Parag P, Kimberly R, et al. Trivalent chromium inhibits protein glycosylation and lipid peroxidation in high glucose-treated erythrocytes. Antioxid Redox Signal. 2006;8:238–41.
4. Wang J, Wang C, Li S, Li W, Yuan G, Pan Y, et al. Anti-diabetic effects of *Inonotus obliquus* polysaccharides in streptozotocin-induced type 2 diabetic mice and potential mechanism via PI3K-Akt signal pathway. Biomed Pharmacother. 2017;95:1669–77.

## 101 Phytochemicals in hops in preventing and managing of metabolic syndrome

### Pavel Dostálek, Marcel Karabín, Lukáš Jelínek

#### Department of Biotechnology, University of Chemistry and Technology, Prague, Technická 5, 16628 Prague, Czech Republic

##### **Correspondence:** Pavel Dostálek - Pavel.Dostalek@vscht.cz

*Journal of Chinese Medicine* 2018, **13(Suppl 1):**101

**Background:** In many countries, the prevalence of metabolic disease has attained epidemic proportions because of cardiovascular complications and mortality. A metabolic syndrome is associated with risk from 5 factors: abdominal (central) obesity, elevated blood pressure, elevated plasma glucose, high serum triglycerides, and low high-density lipoprotein levels. Treatment is focused on reduction of the risk of heart disease by lowering LDL cholesterol and reducing high blood pressure, and then on treatment of diabetes. Very important is a reduction in weight by proper diet and exercise [1].

**Results:** Beneficial effects of hop phenolic compounds for diabetic patients consist of improving blood glucose and lipid profiles, and reducing insulin resistance. Phenolic compounds exert antiobesity effects that are closely linked with antioxidant effects, through their ability to modulate lipid and energy metabolism and thus enable weight loss and reduce obesity. Regulation of cholesterol metabolism by phenolic compounds may reduce certain factors linked with hypercholesterolemia and dyslipidemia. Prenylflavonoids such as xanthohumol have been demonstrated to have strong antiobesity activities including the ability to inhibit diacylglycerol acyltransferase in rat liver, to inhibit triglyceride transport using a HepG2 cell line, and to inhibit the secretion of apolipoprotein B, the main constituent of the cholesterol LDL fraction. Therapeutic potential of polyphenols as treatment for obesity is also associated with the ability to inhibit α-glucosidase, an enzyme that controls blood glucose levels [1, 2]. Iso-α-acids are able to improve health by influencing lipid metabolism, glucose tolerance, and body weight. Diabetic mice treated with iso-α-acids showed reduced plasma glucose, triglyceride, and free fatty acid levels by 65.3, 62.6, and 73.1%, respectively. When mice were fed hop iso-α-acids in their diet, with high levels of cholesterol, an increase in plasma HDL-cholesterol and a reduction in cholesterol and triacylglycerol content in the liver were observed. The modulatory effect of iso-α-acids on lipid metabolism may also be responsible for lost body weight [1].

**Conclusions:** For treatment of metabolic syndrome should be very effective hop substances from group of polyphenols and bitter acids.

**References**

1. Karabín M, Hudcová T, Jelínek L, et al. Compr Rev Food Sci Food Saf. 2016;15:542–67.
2. Karabín M, Hudcová T, Jelínek L, et al. Biotechnol Adv. 2015;33:1063–90.

## 102 Structural analysis and antidiabetic activity of polysaccharide from G*rifola frondosa* in human HepG2 cells

### Yuqing Chen^1^, Chengfeng Yang^1,2^, Bin Liu^1^, Chao Zhao^1,3^

#### ^1^College of Food Science, Fujian Agriculture and Forestry University, Fuzhou 350002, China; ^2^College of Food Science and Nutritional Engineering, China Agricultural University, Beijing 100083, China; ^3^Department of Chemistry, University of California, Davis 95616, CA, USA

##### **Correspondence:** Chao Zhao - zhchao@live.cn

*Journal of Chinese Medicine* 2018, **13(Suppl 1):**102

**Background:** Polysaccharides isolated from mushroom have been widely studied in the medicine area due to their biological functions [1]. *Grifola frondosa* is one of the most valued traditional medicines and has been used as health food for centuries in China, Japan, and other Asian countries [2].

**Materials and methods:** A novel polysaccharide, coded as GFP-N, has been successfully isolated from mushroom. GFP-N was obtained by hot-water extraction and purified by DEAE-cellulose DE-52 and Sephadex G-100 column chromatography. Fourier transform infrared (FT-IR) spectroscopy, one-dimensional nuclear magnetic resonance spectroscopy (^1^H NMR and ^13^C NMR), and two-dimension NMR spectroscopy (^1^H-^1^H COSY, HMQC and HMBC) were used to display the structural characterization of GFP-N.

**Results:** Chemical and spectral analysis revealed that GFP-N possessed a 1,6-β-d-glucan backbone with a single 1,3-α-d-fucopyranosyl side-branching unit. GFP-N was was tested for its antidiabetic activity in insulin resistant human HepG-2 cells which induced by 1 μM dexamethasonin. The result indicated that GFP-N could increase the glucose consumption by 48.50% in insulin resistant HepG-2 cells. GFP-N orally administered at 75 and 100 mg/kg body weight/day could significantly reduce the blood glucose level by 46.02 and 57.58% in streptozotocin/high fat diet-induced type 2 diabetic mice, respectively. Oral administration of GFP-N caused no changes in feeding behavior and there were no harmful effects in mice. The effects of long-term lower GFP-N treatment increased the body weight. The results of the effect of GFP-N on oral glucose tolerance in diabetic mice were assessed. Two hours after sugar administration, the blood glucose concentrations had returned to normal or were lower than before the administration.

**Conclusions:** The result revealed that GFP-N from *G. frondosa* possessed potent antidiabetic properties. The polysaccharide may be useful as a functional food additive and a hypoglycemic agent.

**Acknowledgements:** This work was financially supported by Natural Science Foundation (2016J06009 & 2017N5003) of Fujian Province, China, Key Project of Fuzhou Municipal Bureau of Science and Technology (2017-N-36), and FAFU grants (KXb16011A & XJQ201608).

**References**

1. Zhao C, Gao LY, Wang CY, et al. Carbohydr Polym. 2016;144:382–9.
2. Meng M, Cheng D, Han LR, et al. Carbohydr Polym. 2017;157:1134–43.

## 103 Basic characteristics of konjac glucomannan-gum tragacanth hydrogels

### Jing-ni Gong, Yi Yuan, Lin Wang, Jia-yu Wu, Ruo-jun Mu, Xin Hong, Jie Pang

#### College of Food Science, Fujian Agriculture and Forestry University, Fuzhou, Fujian, China

##### **Correspondence:** Jie Pang

*Journal of Chinese Medicine* 2018, **13(Suppl 1):**103

**Background:** KGM/GT hydrogel was prepared by using Konjac Glucomannan (KGM) and gum tragacanth (GT) in order to develop a non-toxic hydrogel with high mechanical efficiency.

**Materials and methods:** Rheological and textural properties of KGM/GT hydrogels were evaluated using a rheometer and texture analyzer.

**Results:** The results showed that KGM/GT hydrogel is a thermoreversible hydrogel which dissolves into sol when the temperature exceeds 40 °C. The higher the proportion of GT, the higher the entanglement of KGM/GT hydrogel network, and the stronger the gel property. The KGM/GT hydrogel proportion at 3:7 revealed a tight connection between the hydrogels and the hardness reached its maximum. The KGM/GT hydrogel proportion at 5:5, showed the highest adhesiveness, chewiness and recovery. The KGM/GT hydrogel proportion at 9:1 showed a loose connection between the hydrogel chains (Figs. 1, 2).

**Fig. 1:**
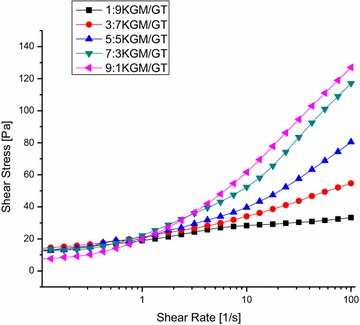
The relationship between the shear rate and shear stress of the KGM/GT

**Fig. 2:**
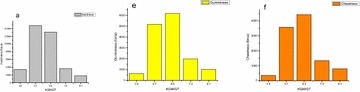
Texture properties of KGM-GT hydrogel for different concentration

**Conclusions:** This KGM/GT hydrogel improved the natural polymer hydrogel weakness of need additional cross-linking, insecurity, long preparation time and other shortcomings. The development and rational use of KMG/GT hydrogel has a certain guiding significance for the research of new hydrogels.

**References**

1. Ahmed EM. Hydrogel. J Adv Res. 2015;6(2):105–121.
2. Chan H, Mu RJ, Pang J, Tang XD. Chin J Struct Chem. 2016;01:166–8.
3. Wang L, Zhuang Y, Li J, et al. The textural properties and microstructure of konjacglucomannan–tungsten gels induced by DC electric fields. Food Chem. 2016;212:256–63.

## 104 Spectroscopic study on the interaction between biological active molecules and *S*-ovalbumin

### Qun Huang^1^, Hui Teng^1^, Meihu Ma^2^, Hongbo Song^1^, Meiyu Xu^1^, Xixi Wang^1^

#### ^1^College of Food Science, Fujian Agriculture and Forestry University, Fuzhou 350002, China; ^2^National R&D Center for Egg Processing, Food Science and Technology College, Huazhong Agricultural University, Wuhan, Hubei 430070, China

##### **Correspondence:** Meihu Ma - mameihuhn@163.com; Hongbo Song - sghgbode@163.com

*Journal of Chinese Medicine* 2018, **13(Suppl 1):**104

**Materials and methods:** The interaction between biological active molecules and *S*-ovalbumin was studied. The biological active molecules used in this work are resveratrol, riboflavin and curcumin.

**Results and conclusions:** Results showed that the quenching of intrinsic fluorescence of *S*-ovalbumin is produced by the formation of a complex. The value obtained for the binding constant, according to the Stern–Volmer equation, deduced the existence of static quenching mechanism. The distance between biological active molecules and *S*-ovalbumin was calculated to be less than 7 nm. The values obtained for the thermodynamic parameter ∆H and ΔS suggested the participation of vad der Waals force and hydrogen bonds in the binding of biological active molecules to *S*-ovalbumin, and the negative value of ∆G revealed the binding process is spontaneous. Finally, the conformational change of *S*-ovalbumin in the presence of biological active molecules was confirmed based on the information obtained from the evaluation of synchronous fluorescence spectra and UV–Vis spectra.

## 105 Ultrasonic-assisted extraction of polyphenols, flavonoids and anthocyanins from raspberry and its antioxidant activity evaluation

### Kang Li, Lei Chen, Qun Huang, Hongbo Song, Hui Teng

#### College of Food Science, Fujian Agriculture and Forestry University, Fuzhou, Fujian 350002, China

##### **Correspondence:** Kang Li

*Journal of Chinese Medicine* 2018, **13(Suppl 1):**105

**Background:** Raspberry is recognized as the third generation of golden fruit in the world, since it contains abundant flavonoid and phenolic contents, which are potentially effective on the prevention of cancer, diabetes, obesity and other diseases. However, extraction efficiency for raspberry using traditional technique is quite low. Thus, the present study employed ultrasonic-assisted extraction aiming at highly efficient extraction of functional compounds from raspberry, and overall evaluation of its antioxidant activity with multiple assays.

**Materials and methods**: The present study used frozen-dried raspberry powders for ultrasonic-assisted extraction. Total phenolic, flavonoid contents as well as anthocyanin content were determined with spectrophotometer. DPPH radical scavenging capacity and total antioxidant capacity (T-AOC) were used for antioxidant activity evaluation for raspberry extract.

**Results**: The results showed that characteristic wavelengths for flavonoid and phenolic compounds in raspberry were detected at 510 and 619 nm, respectively. And total phenolic and total flavonoid contents in raspberry were 26.0 and 45.6 mg/g dry weight basis, respectively. The total anthocyanin content was 2.58 mg/g dry weight basis. Besides, the DPPH radical scavenging activity of raspberry extract was 89.4%, and the total antioxidant capacity (T-AOC) was 56.61 units/mL.

**Conclusions**: Raspberry has a significant antioxidant activity which may relate with its polyphenol, flavonoid and anthocyanin.

## 106 Preparation and characterization of pH-sensitive polymer network hydrogels based on carboxymethyl konjac glucomannan and polydopamine for in vitro drug delivery

### Lin Wang^1^, Weihai Wang^1^, Yi Yuan^1^, Jingni Gong^1^, Ziqi Liu^2^, Weihao Wu^1^, Jie Pang^1^

#### ^1^College of food science, Fujian Agriculture & Forestry University, Fuzhou, China, 350002; ^2^College of materials and engineering, Fujian Agriculture & Forestry University, Fuzhou, China, 350002

##### **Correspondence:** Jie Pang - pang3721941@163.com

*Journal of Chinese Medicine* 2018, **13(Suppl 1):**106

**Background:** Konjac glucomannan (KGM) is a water-soluble non-ionic polysaccharide extracted from tubers of the Amorphophallus konjac plant, among the konjac glucomannan-based biomaterials, carboxymethyl konjac glucomannan-based hydrogels have been widely studied, inspired by the properties of adhesive proteins in marine mussels, dopamine (DA), was able to self-polymerize, we developed CMKGM/PDA hydrogels to exploit the properties of CMKGM and PDA for colon-targeting delivery.

**Materials and methods:** A novel pH-sensitive 3D polymer network hydrogel is prepared by using Carboxymethyl konjac glucomannan (CMKGM) and polydopamine (PDA), the structure of the 3D polymer network hydrogel was characterized by fourier transform infrared spectroscopy (FT-IR), scanning electron microscopy (SEM) and Differential Scanning Calorimeter (DSC). The equilibrium swelling property of the hydrogels were studied in different mediums, the drug loading efficiency and drug release behavior of hydrogels were also evaluated using Ofloxacin as the model drug.

**Results:** Results indicated that PDA interacted with CMKGM in the prepared hydrogels and obviously enhanced the mechanical properties, swelling abilities, thermal properties and drug loading efficiency of the CMKGM/PDA hydrogels, due to the hydrogen bond interaction. At pH 3, the release amount of Ofloxacin incorporated into the hydrogels was about 26% within 240 min, while this value approached to 87% at pH 7.2.

**Conclusions:** These results showed that the loading and release showed dependence on the 3D network structure of hydrogels, 3D hydrogels could be a suitable controlled release carrier for site-specific drug delivery in the intestine.

## 107 Effects of cross-pollination on physicochemical properties, phytochemicals and antioxidant capacities of ‘Fuju’ (*Citrus reticulata* Blanco)

### Qi Wang^1,2^, Huiying Gao^1^, Yafeng Zheng^3^, Chengchun Lai^1,2^, Xiangui Huang^1^

#### ^1^Institute of Agricultural Engineering, Fujian Academy of Agriculture Sciences, Fuzhou, China; ^2^Fujian Key Laboratory of Agricultural Product (Food) Processing, Fuzhou, China; ^3^College of Food Science, Fujian Agriculture and Forestry University, Fuzhou, China

##### **Correspondence:** Xiangui Huang - faashxg@163.com

*Journal of Chinese Medicine* 2018, **13(Suppl 1):**107

**Background:** ‘Fuju’ (*Citrus reticulata* Blanco) is a traditional Chinese citrus cultivar, which has been well-accepted by consumers due to its attractive peel colour, pleasant flavors and healthy benefits. The cultivation of ‘Fuju’ was reducing every year, due to the relatively poor fruit quality. Cross-pollination was reported to be an effective method to increase the fruit yield of citrus cultivars suffering from inadequate yield [1, 2]. In our previous study, cross-pollination by ‘Murcott’ tangor (*Citrus reticulata* Blanco × *Citrus sinensis* Osbeck) was found to have a potential of improving the fruit quality of ‘Fuju’. The present study aimed to investigate the effect of cross-pollination by ‘Murcott’ tangor on the ‘Fuju’ fruit qualities, including physicochemical properties, phytochemicals and antioxidant capacities.

**Materials and methods:** Fifteen fruit samples were randomly chosen from self-pollination (SP) and cross-pollination (CP) ‘Fuju’ trees. The physicochemical properties, including fruit weight, juice yield, total soluble solids (TSS), titratable acidity (TA) and Vitamin C, as well as the phytochemicals, including total polyphenolic and carotenoid contents, were determined. Antioxidant capacities, including the free radical-scavenging and total antioxidant capacities, were measured. High-performance liquid chromatographic (HPLC) method was used to identify and quantify three sugars (sucrose, glucose and fructose).

**Results:** Among the physicochemical properties, there were no significant differences on the fruit weight, juice yield and Vitamin C content between CP and SP fruits. However, TSS of CP fruits increased from 11.08 ± 1.12% (SP) to 13.70 ± 1.27%, while TA decreased from 0.81 ± 0.06% (SP) to 0.68 ± 0.03%. The results of HPLC analysis of three sugars indicated that the sum of sugars of CP fruits was significantly higher than that of SP fruits, which were mainly contributed by the increase of fructose and sucrose. The cross-pollination exhibit no effect on the carotenoid content, while the total polyphenolic content of CP fruits was 287.36 ± 16.21 mg/L, which was significantly higher than that of SP fruits (216.14 ± 13.45 mg/L). CP fruits showed higher free radical-scavenging and total antioxidant capacities with the similar trend as the total polyphenolic content.

**Conclusions:** In this study, the physicochemical properties, phytochemicals and antioxidant capacities of SP and CP ‘Fuju’ fruits have been examined. Higher levels of TSS, total sugars, polyphenolic and antioxidant capacities and lower levels of TA were detected in CP fruits. Therefore, ‘Murcott’ was proved to be a good pollenizer for ‘Fuju’ to enhance taste qualities and healthy benefits.

**Acknowledgements:** This study is funded by FAAS Scientific and Technological Innovation Team (Grant Number: STIT2017-1-10)

**References**

1. Schneider D, Goldway M, Rotman N, Adato I, Stern RA. Cross-pollination improves ‘Orri’ mandarin fruit yield. Sci Hortic. 2009;122(3):380–4.
2. Papadakis IE, Protopapadakis EE, Therios IN. Yield and fruit quality of ‘Nova’ hybrid [Citrus clementina hort. ex Tanaka × (C. reticulata Blanco × C. paradisi Macfad)] and two Clementine varieties (C. clementina hort. ex Tanaka) as affected by self- and cross-pollination. Sci Hortic. 2009;121(1):38–41.

## 108 Preparation of dietary fibers from bamboo shoot (*Leleba oldhami* Nakal) shell and their hypoglycemic and hypolipidemic effects

### Dongya Fang^1^, Qi Wang^1,2^, Xianliang Luo^1^, Yafeng Zheng^1^

#### ^1^College of Food Science, Fujian Agriculture and Forestry University, Fuzhou, China; ^2^Institute of Agricultural Engineering, Fujian Academy of Agriculture Sciences, Fuzhou, China

##### **Correspondence:** Yafeng Zheng - zyffst@163.com

*Journal of Chinese Medicine* 2018, **13(Suppl 1):**108

**Background:** Dietary fiber consumption has been proved to be strongly associated with the beneficial effects on lipid profile, blood sugar levels, bowel function and prevention of certain chronic diseases [1]. Bamboo shoots have been consumed as a nutritious and healthy food for a long history, especially in the Asia. Due to the short storage period, most of fresh bamboo shoots are processed to various kinds of food products, which generates huge quantity bamboo shoot shells (BSS) as by-products without any utilization. In our previous studies, BSS could not only be a natural source for the extraction of polysaccharides [2], but also a potential source for dietary fiber production with relatively low costs.

**Materials and methods:** BSS was decomposed by multiple enzymes to prepare dietary fiber samples, including insoluble dietary fiber (IDF), soluble dietary fiber (SDF) and total dietary fiber (TDF, the sum of IDF and SDF). To investigate the in vitro characteristics of resulting samples, swelling capacity, water holding capacity and binding capacities to fat, cholesterol, glucose, bile acids and nitrites, as well as the inhibition effects of alpha glucosidase and alpha amylase activities were determined and compared. Moreover, in vivo hypoglycemic and hypolipidemic effects were measured using the type 2 diabetic mice induced by streptozotocin (STZ) injection and high-fat diet.

**Results:** The in vitro experiment results revealed that all three fiber samples had favorable water holding capacity and swelling capacity, which contribute to the increase of the bulk volume and the decrease of calories of food containing fibers. The samples exhibited significant in vitro binding capacities to fat, cholesterol, glucose, bile acids and nitrites, as well as the inhibition effects of alpha glucosidase and alpha amylase activities, which might be partly mechanism for their hypolipidemic and hypoglycemic effects. The results of animal experiment showed that administration of three types of BSS fibers could significantly ameliorate the blood glucose and lipid metabolism disorderly situation of the diabetic mice. Especially, compared with model group, TDF supplement could exhibit the lowest body weight gain (2.84%), and decrease serum glucose, total cholesterol, triglyceride and low density lipoprotein-cholesterol by 40.42, 31.53, 21.35 and 31.53%, respectively, while increase high density lipoprotein-cholesterol by 37.6%.

**Conclusions:** These excellent physiological activities indicate that BSS fibers could be a potentially available dietary ingredient in functional food industries.

**References**

1. Mudgil D, Barak S. Composition, properties and health benefits of indigestible carbohydrate polymers as dietary fiber: a review. Int J Biol Macromol. 2013;61(10):1.
2. Zheng Y, Zhang S, Wang Q, et al. Characterization and hypoglycemic activity of a β-pyran polysaccharides from bamboo shoot (*Leleba oldhami* Nakal) shells. Carbohydr Polym. 2016;144:438–46.

## 109 The extraction of natural ursolic acid and its application in functional food

### Yuxia Qi, Chenghui Zhang, Xuqiao Feng

#### College of Food Science and Engineering, Bohai University, Jinzhou, Liaoning 121013, China

##### **Correspondence:** Xuqiao Feng

*Journal of Chinese Medicine* 2018, **13(Suppl 1):**109

**Background:** Ursolic acid has become a hot research in recent years because of its broad physiological functions such as antioxidation, anti-aging, anti-tumor and liver protection. Natural ursolic acid has a wide range of sources and various extraction methods, but the extraction rate is generally low, in addition ursolic acid insoluble in water, which is mainly used in cosmetics and medical industry, limits its application in food and drug industry, to find suitable methods to raise the extraction rate and to improve the solubility of ursolic acid can improve the utilization rate of the food and drug industry and is the main direction of the current.

**Results:** This paper mainly introduces the research status of ursolic acid health value, puts forward some methods of extracting ursolic acid with high efficiency, expatiates the applicability of ursolic acid in food industry, and its application prospect in food industry were summarized and been prospected.

**Conclusions:** To raise the extraction rate, high intensity pulsed electric field (HIPEF)-assisted enzymatic extraction will be employed in future research for the optimization of ursolic acid extraction. In addition, microcapsule embedding technology may provide some technical support for ursolic acid applications, embedding ursolic acid molecules with microcapsule technology, increasing its solubility, and promoting ursolic acid as a functional ingredient in food and drug.

## 110 Immuno-enhancing effect of a polysaccharide fraction PRM3 from *Rhynchosia minima* root

### Xuejing Jia, Ye’er Liang, Kai Wang, Chao Zhang, Chengwei He

#### State Key Laboratory of Quality Research in Chinese Medicine, Institute of Chinese Medical Sciences, University of Macau, Macao, China

##### **Correspondence:** Chengwei He - chengweihe@umac.mo

*Journal of Chinese Medicine* 2018, **13(Suppl 1):**110

**Background:** Polysaccharides, one of the active ingredients in traditional Chinese medicine, are proved to enhance innate immunity against infections. Macrophages, a kind of phagocytic cells, serve as sentry for the immune response. It is well known that macrophages play a key role in the removal of damaged cells and pathogens. In this study, we aimed to evaluate the immune-enhancement ability of polysaccharide fraction PRM3 from *Rhynchosia minima* root in an in vitro model of RAW 264.7 macrophage.

**Materials and methods:** PRM3 was obtained from *R. minima* root and purified by DEAE-52 cellulose column. The cytotoxicity and production of nitric oxide (NO) of PRM3 were determined to acquire optimal concentrations. RAW 264.7 macrophages were treated with PRM3 for 24 h, and lipopolysaccharide (1 μg/mL) was used as control. The reactive oxygen species (ROS) was quantitatively measured by flow cytometry and qualitatively observed by Incell 2000. The cytokines in the culture medium were tested, and proteins and RNA in cells were tested by Western blot and real time PCR, respectively. The translocation of p65 was observed by confocal laser scanning microscopy to test the activity of NF-κB.

**Results:** Three concentrations (1, 0.25, and 0.063 mg/mL) of PRM3, at which no cytotoxicity was observed, were used to treat the cells. The results showed that PRM3 remarkably stimulated the release of NO and ROS in RAW 264.7 cells. PRM3 enhanced phagocytic ability of RAW 264.7 macrophages (*P *< 0.05 vs. control). Simultaneously, PRM3 stimulated the productions of cytokines (interleukin-6, IL-6; tumor necrosis factor alpha, TNF-α; and monocyte chemoattractant protein-1, MCP-1) (*P *< 0.01 vs. control), and increased the expression of immune-related proteins (cyclooxygenase 2, COX-2; inducible nitric oxide synthase, iNOS; IkappaB kinase alpha/beta, IKK α/β; IkappaB alpha) (*P *< 0.05 vs. control) and genes (IL-6, TNF-α, MCP-1, COX-2, and INOS) in the cells (*P *< 0.05 vs. control). PRM3 was found to promote the nuclear translocation of NF-κB subunit p65. Co-treatment with Toll-like receptor (TLR4) inhibitor TAK-242 and NF-κB inhibitor PDTC attenuated the activities of PRM3, further suggested that PRM3 might exert immune-enhancing effect via TLR4/NF-κB signaling pathway.

**Conclusions:** In conclusion, PRM3 can enhance the immune function and is a promising candidate of immunopotentiator applied in functional foods or drugs.

**Acknowledgements:** This study is supported by the Research Fund of the University of Macau (MYRG2015-00081-ICMS-QRCM).

## 111 Research advances on inhibitory effects of mixed phytochemical polyphenols on α-glucosidase

### Chenghui Zhang, Xuqiao Feng

#### College of Food Science and Engineering, Bohai University, Jinzhou Liaoning 121013, China

##### **Correspondence:** Xuqiao Feng

*Journal of Chinese Medicine* 2018, **13(Suppl 1):**111

**Background:** α-Glucosidase is a kind of enzymes that catalyzes the hydrolysis of alpha glucosyl at the non-reducing end of the substrate containing the alpha glycosidic bond. It mainly affects the degradation of starch and other carbohydrates into glucose, and eventually affects the absorption and utilization of glucose by human blood. The inhibitor of α-glucosidase is a kind of novel substances which can inhibit the activity of α-glucosidase, and delay the decomposition and absorption of carbohydrates in human body. Among them, polyphenol compounds are a kind of natural products with high biological activity as an inhibitor of α-glucosidase, and can be potentially used as a new type of anti-diabetic health-keeping food or drugs.

**Results:** *Hippophae rhamnoides*, as one of the most valuable new plants, possesses high values of nutrition, health-keeping and medicine etc. Polyphenols is one of the most important bioactive components of *H. rhamnoides*. Research will be conducted that mainly aims to explore the inhibitory performance of mixed phytochemical polyphenols mainly composed of that extracted from *H. rhamnoides* on their inhibition effect on the activities of α-glucosidase both in vivo and in vitro experiments.

**Conclusions:** This paper mainly introduces the research status of functional value of *Hippophae rhamnoides*, points out modern extraction technologies of the leaves of *H. rhamnoides* polyphenols with high extract efficiency. Polyphenols extracted from *H. rhamnoides* will be combined with polyphenols from other sources to form the best formula for optimal α-glucosidase inhibition effect both in vitro and in vivo experiments. Microencapsulation of the polyphenols of the formula will be conducted to finalize a commercial product whose function to control blood glucose.

## 112 Rhinacanthins-rich extract: a potent synergistic α-glucosidase inhibitor and glucose uptake enhancer in muscles cells

### Muhammad Ajmal Shah^1^, Decha Sermwittayawong^2^, Ruqaiya Khalil^3^, Zaheer Ul-Haq^3^, Pharkphoom Panichayupakaranant^1,4^

#### ^1^Department of Pharmacognosy and Pharmaceutical Botany, Faculty of Pharmaceutical Sciences, Prince of Songkla University, Hat-Yai, Songkhla 90112, Thailand; ^2^Department of Biochemistry, Faculty of Science, Prince of Songkla University, Hat-Yai, Songkhla 90112, Thailand; ^3^Dr. Panjwani Center for Molecular Medicine and Drug Research, International Center for Chemical and Biological Sciences, University of Karachi, Karachi 75270, Pakistan; ^4^Phytomedicine and Pharmaceutical Biotechnology Excellence Center, Faculty of Pharmaceutical Sciences, Prince of Songkla University, Hat-Yai, Songkhla 90112, Thailand

##### **Correspondence:** Pharkphoom Panichayupakaranant - pharkphoom.p@psu.ac.th

*Journal of Chinese Medicine* 2018, **13(Suppl 1):**112

**Background:** Rhinacanthins-rich extract (RRE) is a semi purified leaf extract that contains 60% w/w of rhinacanthin-C (RC) obtained from *Rhinacanthus nasutus* leaf, a popular medicinal plant used in Thai traditional medicine and an herbal drink in Taiwan and China. RC has been reported for its antidiabetic activity but the commercial unavailability and high production cost of RC necessitate the preparation of RRE and its antidiabetic evaluation.

**Materials and methods:** In the present study RRE was prepared by microwave assisted green extraction method along with a simple step of fractionation using Amberlite^®^ column. RC was isolated from the RRE using silica gel column chromatography. Based on previous IC_50_ data, RRE and RC were evaluated for synergistic α-glucosidase inhibition with acarbose. Cell viability and glucose uptake stimulation assay was performed in L6 muscles cells of rat.

**Results:** RRE and RC, as noncompetitive α-glucosidase inhibitors showed synergistic inhibition at low concentrations (¼IC_50_, ½IC_50_ and IC_50_) in combinations with a competitive inhibitor acarbose. Furthermore, in silico studies explained the structure activity relationship of the RC as ligand with α-glucosidase. In glucose uptake assay, the L6 cells were incubated with various concentrations (0.32–2.5 µg/mL) of RRE and RC. The RRE and RC at a concentration of 2.5 µg/mL showed almost equal (> 80%) glucose uptake stimulation in comparison with standard drugs metformin (219.5 µg/mL) and insulin (2.90 µg/mL). On the basis of MTT assay, percentage viability of RRE and RC treated L6 cells (2.5 µg/mL) was almost 90%.

**Conclusions:** Based on the synergistic α-glucosidase inhibitory effect and glucose uptake stimulation in muscles cells, RRE with RC as a marker compound can be used as a natural glucose lowering agent in both post and pre-prandial hyperglycemia.

**References**

1. Panichayupakaranant P, Charoonratana T, Sirikatitham A. J Chromatogr Sci. 2009;47:705–8.
2. Shah MA, Panichyupakaranant P. 4th CDD, June 1–3 2016, Phuket Thailand.
3. Shah MA, Khalil R, Haq ZU, Panichyupakaranant P. J Func Foods. 2017 (submitted)

## 113 Eastern and homeopathic multi-ingredient antihypertensive remedies containing *Rauwolfia serpentina*: are they equivalent?

### Khalid Hussain, Nadeem Irfan Bukhari, Muhammad Islam, Amjad Hussain

#### University of the Punjab, Faculty of Pharmacy, Lahore, 54000, Pakistan

##### **Correspondence:** Khalid Hussain - khussain.pharmacy@pu.edu.pk

*Journal of Chinese Medicine* 2018, **13(Suppl 1):**113

**Background:** The remedies, prepared contrary to the classical eastern medicine (*Tibb*) and homeopathy practices, containing ingredients discontinued in the modern medicine are supposed to be an overlie to allopathy, hence may not devoid of claimed safety of these systems of treatment. To unearthing the fact, the present study describes reserpine-based equivalence of two most commonly used poly-ingredient antihypertensive herbal and homeopathic preparations containing roots of *Rauwolfia serpentina* L. (Benth.) ex kruz (family: *Apocynaceae*).

**Materials and methods:** A simple analytical method developed for the determination of reserpine using a combined TLC and HPLC approach was applied to investigate the remedies. The standards/samples drugs were applied band-wise on TLC plate, separately, and developed with mobile phase, chloroform: methanol (2:1, v/v). The air-dried plates were then placed in an oven at 100 °C for 10 min and viewed at 254 nm. The bands were scratched and silica gel was eluted with 5 mL methanol. The solvent was evaporated and the residue was reconstituted with 1 mL mobile phase. Each sample/standard solution (20 µL) was eluted through column—Eclipse X DB-C18 (5 µm, 4.6 × 150 mm)—using isocratic mobile phase comprising phosphate buffer (0.01 M): acetonitrile (65:35, v/v) adjusted to pH 3.0 with orthophosphoric acid, at flow rate of 1.0 mL/min. The temperature of the column was maintained at 25 °C and detection was carried out using FLD detector; 280 nm excitation and 360 nm emission. The peaks were identified by comparing retention time to that of the standard. Calibration curve was constructed between concentration and peak area for the quantification of the reserpine in samples.

**Results:** The extract of the roots and the two remedies were found to contain equivalent amount of reserpine.

**Conclusions:** The results of the present study indicate that such eastern and homeopathic remedies containing extracts and undiluted stocks need to be monitored like allopathic drugs to avoid toxic effects of reserpine.

## 114 Antioxidant effects of *Agrimonia pilosa* Ledeb: in vitro comparative activities of its various fractions

### Lei Chen^1,2^, Hui Teng, Benyao Yuan^1^, Young-Hwa Kang^2^, Ting Fang^1^

#### ^1^College of Food Science, Fujian Agriculture and Forestry University, Fuzhou, Fujian 350002, China; ^2^Department of Horticultural Sciences, College of Agriculture & Life Sciences Kyungpook National University, 1370 Sankyuk, Daegu 702-701, Republic of Korea

##### **Correspondence:** Young-Hwa Kang; Ting Fang

*Journal of Chinese Medicine* 2018, **13(Suppl 1):**114

**Background:** *Agrimonia pilosa* Ledeb is a traditional Chinese herb which has been used to treat or prevent a wide range of diseases such as cancer, obesity, diabetes and cardiovascular disorders. However, rare research can be found on its antioxidant evaluation, thus, the present study aims at in vitro antioxidant evaluation of *Agrimonia pilosa* Ledeb by using multiple assays.

**Materials and methods:** The antioxidant potentials of *Agrimonia pilosa* Ledeb extract were measured as DPPH, and 2.2′-azino-bis-(3-ethylbenzothiazoline-6-sulphonic acid) (ABTS) radical scavenging abilities as well as the reducing power decreasing NO in vitro. The methanol extract of *A. pilosa* Ledeb showed significantly scavenging effects on free radicals of DPPH, ABTS, and NO. Hexane fraction (HF), ethyl acetate fraction (EF) and *n*-butyl alcohol fraction (BF) were prepared by solvent fractionation.

**Results:** By comparison with BF and HF, EF with high polyphenol contents, showed the highest DPPH, ABTS, and NO inhibition with scavenging value of 50.2, 80.4, and 65.7%, respectively, at the highest tested dose. As a result, total phenol content was strongly correlated to flavonoids contents (r = 0.78, *p *= 0.002).

**Conclusion:** There were meaningful positive correlations between antioxidant activity and total phenols and flavonoids content.

## 115 Effects of α-glucosidase inhibitors from *Agrimonia pilosa* Ledeb on hepatic glucose release and glycaemia

### Hui Teng, Lei Chen, Qiyang Lin, Benyao Yuan, Ting Fang

#### College of Food Science, Fujian Agriculture and Forestry University, Fuzhou, Fujian 350002, China

##### **Correspondence:** Lei Chen

*Journal of Chinese Medicine* 2018, **13(Suppl 1):**115

**Background:** The present study is aimed to investigate the effect of several active compounds isolated from *Agrimononia pilosa* Ledeb on the improvement of insulin resistance and action mechanism in insulin-responsive HepG2 cell lines.

**Materials and methods:** Six compounds, agrimonolide (k1), desmethylagrimonolide (k2), tormentic acid (k3), ursolic acid (k4), belamcandonep (k5), and quercetin (k6) were successfully isolated as α-glucosidase inhibitors by column chromatography.

**Results:** Among them, ursolic acid and belamcandonep had the strongest activity compared to other compounds with the lowering value of 71.5% (1.24 mM glucose in DMEM) and 71.7% (1.23 mM), respectively. Agrimonolide effectively increased insulin-mediated glycogen synthesis in HepG2 cells. At a concentration of 20 μM, desmethylagrimonolide showed the strongest glucokinase (GK) activity (3.2 U/min/mg protein), followed by agrimonolide (3.0 U/min/mg protein), both of them significantly (p < 0.05) increased the GK activity compared to the control. In the same line, desmethylagrimonolide and agrimonolide caused a significant reduction of glucose-6-phosphatase (G6Pase) activity and a meaningful change in phosphoenol pyruvate carboxykinase (PEPCK) activity.

**Conclusion:** These compounds isolated from *A. pilosa* Ledeb exhibited anti-diabetic properties and were able to reduce certain diabetic complications related to hyper-lipidemia.

## 116 Anti-hyperglycemic effects of *Agrimonia pilosa* Ledeb

### Hui Teng, Qiyang Lin, Benyao Yuan, Ting Fang, Lei Chen

#### College of Food Science, Fujian Agriculture and Forestry University, Fuzhou, Fujian 350002, China

##### **Correspondence:** Lei Chen - chenlei841114@hotmail.com

*Journal of Chinese Medicine* 2018, **13(Suppl 1):**116

**Background:** This research aims at investigating enzyme inhibitory activities and nutraceutical properties of *Agrimonia pilosa* Ledeb against α-amylase and α-glucosidase.

**Materials and methods:** The crude MeOH extract (ME)of *A. pilosa* Ledeb was obtained, suspended in hot water and partitioned with *n*-hexane, EtOAc, and BuOH successively, to afford *n*-hexane-(HF), EtOAc-(EF), and BuOH-(BF) soluble fractions.

**Results:** The methanol extract of *A. pilosa* Ledeb showed α-amylase and α-glucosidase inhibitory potentials with inhibition value of 73.5 and 55.4%, respectively, at the concentration of 200 μg/mL. Furthermore, α-amylase inhibitory effect of the ethyl acetate fraction (EF) of *A. pilosa* Ledeb exhibited a higher inhibitory effect than n-butyl alcohol fraction (BF) and hexane fraction (HF). EF was found to have higher content of phenolics such as hyperside (5), quercetin (7), and apigenin (8) than other fractions. Interestingly, hexane fraction containing low-polarity compounds showed a significant inhibitory effect against α-glucosidase.

**Conclusion:** Correlation analysis revealed that quercitrin was strongly correlated with α-amylase inhibition. Additionally, there were high correlation coefficient between phenolics such as quercitrin (r = 0.58, p < 0.05) or apigenin (r = 0.64, p < 0.64) and α-glucosidase inhibition.

## 117 Identification and characterization of α-glucosidase inhibitors from *Agrimonia pilosa* Ledeb

### Lei Chen, Hui Teng, Qiyang Lin, Benyao Yuan, Ting Fang

#### College of Food Science, Fujian Agriculture and Forestry University, Fuzhou, Fujian 350002, China

##### **Correspondence:** Lei Chen

*Journal of Chinese Medicine* 2018, **13(Suppl 1):**117

**Background:** The complex biochemical composition of *Agrimonia pilosa* Ledeb has been studied as a source of biological components with health-related properties. The present study was interested in its α-glucosidase inhibition effect, and identifying the inhibitors.

**Materials and methods:** *Agrimonia pilosa* Ledeb sample was extracted and separated with hexane fraction (HF), ethyl acetate fraction (EtOAc) and *n*-butyl alcohol fraction (BF). And each fraction was evaluated for its inhibition effect on α-glucosidase and inhibitors were identified using bioactivity-guided isolation.

**Results:** The ethyl acetate (EtOAc) fraction of *A. pilosa* Ledeb showed strong α-glucosidase inhibitory effect. Especially, due to its selectivity towards α-glucosidase, the ethylacetate (EtOAc) fraction was chosen for further study on *A. pilosa* Ledeb. Ten compounds as main active constituents, K1–K10 were isolated and identified as agrimonolide (K1), desmethylagrimonolide (K2), tormentic acid (K3), ursolic acid (K4), belamcandone p (K5), and quercetin (k6), luteolin (K7), luteolin-7-*O*-glucoside (K8), kaempferol (K9), and apigenin (K10). Agrimonolide (K1) and desmethylagrimonolide (K2) showed the strongest inhibitory effects on α-glucosidase (IC_50_ 2.5 and 2.8 μM, respectively).

**Conclusion:** Agrimonolide (K1) and desmethylagrimonolide (K2) were the main inhibitors in *Agrimonia pilosa* Ledeb for α-glucosidase inhibition effect.

## 118 Preparation and characterization of octopus scraps protein hydrolysate-calcium chelate

### Mengru Tang, Shaoyun Wang

#### College of Biological Science and Engineering, Fuzhou University, Fuzhou 350108, China

##### **Correspondence:** Shaoyun Wang - shywang@fzu.edu.cn

*Journal of Chinese Medicine* 2018, **13(Suppl 1):**118

**Background:** Mollusk octopus contains a variety of nutrients, while octopus scraps have caused a large number of marine pollution and resource waste [1]. It is reported that peptide-calcium chelate can probably be a suitable candidate as a supplement to improve calcium absorption in human body [2]. In this work, we investigated the interaction between marine octopus peptides and calcium.

**Materials and methods:** The octopus scraps protein hydrolysate (OSPH)-Ca chelate was prepared and the possible chelating mechanism was investigated by UV spectroscopy, Fluorescence spectroscopy, FTIR spectroscopy and ^1^H NMR. The calcium bioavailability of OSPH-Ca was determined by Caco-2 cell monolayer model

**Results:** According to the optimal enzymolysis condition and chelation condition, the calcium binding capacity of OSPH reached 186.57 calcium ions per milligram peptide and the degree of hydrolysis was 19.78%. The structural properties indicated that amido and carboxy groups could be the reaction sites for chelation and calcium ions might link with oxygen atoms of carboxy group, nitrogen atoms of amido group by coordinate linkage (Fig. 1). In addition, calcium ions chelated with OSPH would cause intramolecular and intermolecular folding and aggregating (Fig. 2). Moreover, the amount of calcium uptake increased by 41% when compared with the CaCl_2_ which was determined by Caco-2 cell lines. Particularly, OSPH-Ca could protect calcium ions from precipitation caused by dietary inhibitors tannic acid and phytate, and calcium uptake efficiency remained 3.35 and 1.68 times higher than that of CaCl_2_, respectively (Fig. 3).

**Fig. 1:**
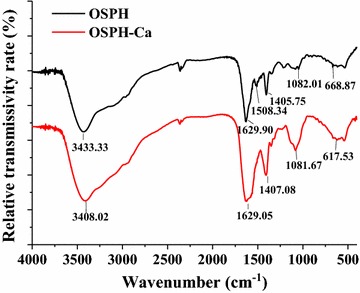
FTIR spectra of OSPH and OSPH-Ca chelate in the regions from 4000 to 400 cm^−1^

**Fig. 2:**
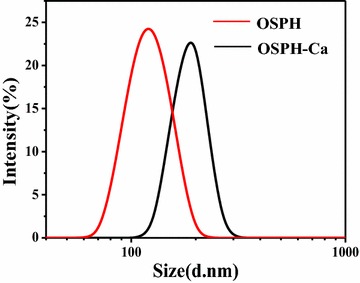
Particle size distributions of OSPH and OSPH-Ca chelate

**Fig. 3:**
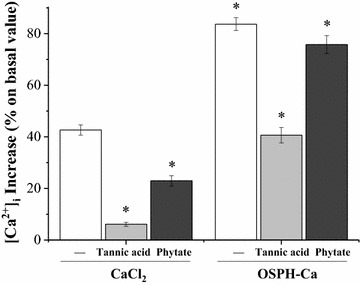
Effect of OSPH–Ca chelate on calcium bioavailability under the action of dietary inhibition factors. *Statistical significance p < 0.05, compared with CaCl_2_ control group

**Conclusions:** These findings further the progress in the research of turning marine waste into food ingredients, suggesting the potential in making marine peptide–calcium chelate as a functional supplement.

**References**

1. Nurdiani R, Dissanayake M, Street WE, Donkor ON, Singh TK, Vasiljevic T. Sustainable use of marine resources-turning waste into food ingredients. Int J Food Sci Tech. 2015;50:2329–39.
2. Perego S, Del Favero E, De Luca P, Dal Piaz F, Fiorilli A, Cantu L, Ferraretto A. Calcium bioaccessibility and uptake by human intestinal like cells following in vitro digestion of casein phosphopeptide-calcium aggregates. Food Funct. 2015;6:1796–1807.

## 119 Periodate oxidation of xanthan gum and its crosslinking effects on gelatin-based hydrogels

### Qingyan He, Shaoyun Wang

#### College of Biological Science and Engineering, Fuzhou University, Fuzhou 350108, China

##### **Correspondence:** Shaoyun Wang - shywang@fzu.edu.cn

*Journal of Chinese Medicine* 2018, **13(Suppl 1):**119

**Background:** Oxidized xanthan gum with different aldehyde content was successfully prepared by periodate oxidization and used as a crosslinking agent for gelatin hydrogels. The effect of xanthan gum with different degree of oxidation and different gelatin/oxidized xanthan gum ratio on the structural and properties of hydrogel was investigated.

**Materials and methods:** Oxidized xanthan gum was prepared by periodate oxidization and then crossed link with gelatin to form chemical cross-linked hydrogels. The swelling degree of different oxidized xanthan gum and different mass ratio of oxidized xanthan gum-gelatin hydrogels were determined gravimetrically. And the chemical composition of the samples was verified by FTIR.

**Results:** The swelling degree of all the samples were enhanced with the degree of oxidation of xanthan gum increased, and reached maximum value at the ratio of 1:1 (Fig. 1). The results of FTIR revealed that Schiff-base formation promoted the crosslink between oxidized xanthan gum and gelatin (Fig. 2). When the mass ratio was 1:1, the characteristic peak of Schiff-base was the strongest.

**Fig. 1:**
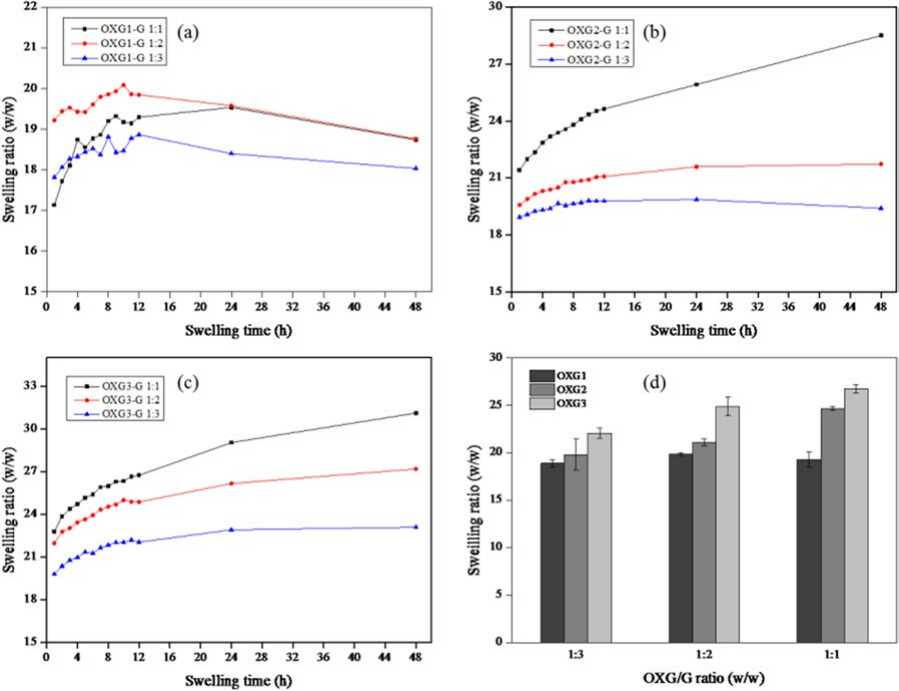
Swelling capacity of OXG-G hydrogels with different mass ratio and degree of oxidation

**Fig. 2:**
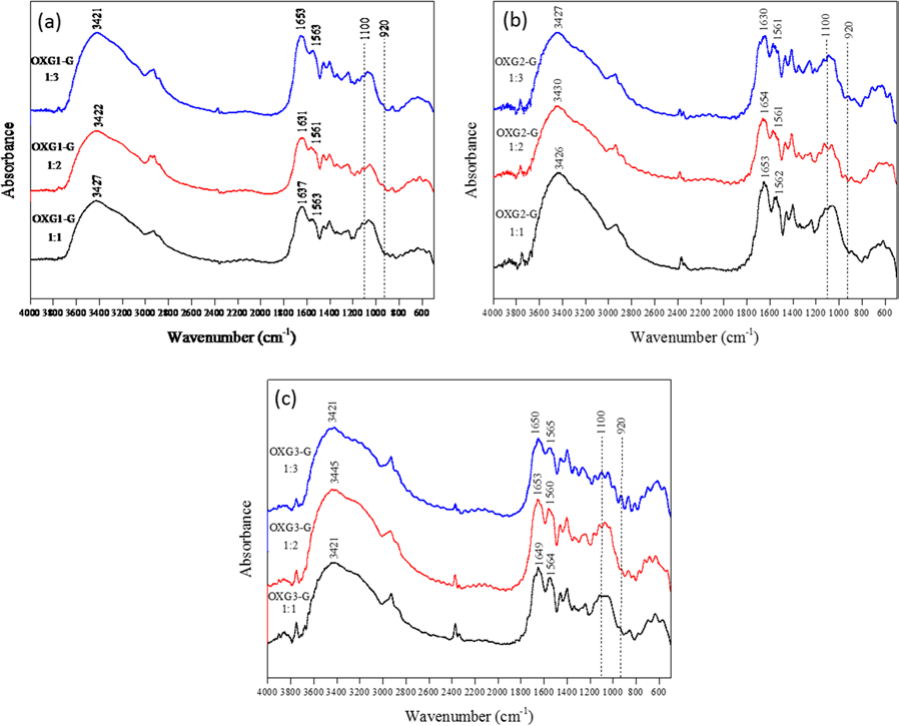
FTIR spectra of OXG-G hydrogels with different mass ratio and degree of oxidation

**Conclusion:** The swelling degree of oxidized xanthan gum/gelatin hydrogels and the amount of Schiff-base reached maximum value when the mass ratio of oxidized xanthan gum/gelatin was 1:1.

**References**

1. Guo JM, Ge LM, Li XY, Mu CD, Li DF. Periodate oxidation of xanthan gum and its crosslinking effects on gelatin-based edible films. Food Hydrocolloid. 2014;39:243–50.
2. Potthast A, Rosenau T, Kosma P. Analysis of oxidized functionalities in cellulose, vol. 205. Berlin: Springer; 2006. p. 1–48.

## 120 Effect of heat-drying process in protein glycation and antioxidant activity of *Radix pseudostellariae*

### Xixi Cai, Shaoyun Wang

#### College of Biological Science and Engineering, Fuzhou University, Fuzhou 350108, China

##### **Correspondence:** Shaoyun Wang - shywang@fzu.edu.cn

*Journal of Chinese Medicine* 2018, **13(Suppl 1):**120

**Background:** Heating is the most common process of traditional Chinese medicine (TCM), which has been confirmed to impact not only on the physical properties of raw materials but also the biological activities. Maillard reaction has been introduced into the study of heat processing of TCM in recent years [1, 2]. In this study, the Maillard reaction and antioxidant activity of the heat-processed *Radix pseudostellariae* (RP) were investigated.

**Materials and methods:** The raw materials were heat-dried at 40, 50 and 60 °C for different time. The Maillard reaction levels were determined. The changes of proteins were analysed by SDS PAGE and FTIR spectroscopy. Besides, the antioxidant activities in vitro were investigated.

**Results:** Increased browning degrees of the extracts were observed temperature- and time-dependently (Fig. 1). The contents of free amino acids and reducing sugar decreased after heat-dried for 24 h at different temperature, indicating the occurring of Maillard reaction. Arginine, the predominant free amino acids of raw RP, decreased from 1.2422 to 0.2388% solid content by heat-dried at 60 °C for 12 h, which contributed to the significant reduction of total free amino acids contents. The smeared bands with high molecular weight in SDS PAGE indicated the conjugation of proteins and saccharides in the Maillard reaction (Fig. 2A), and the glycation was further confirmed by FTIR (Fig. 2B). The DPPH radical scavenging activity, hydroxyl radical scavenging activity and reducing power all increased with thermal treatment (Fig. 3), which was accordance with the changes of browning degree.

**Fig. 1:**
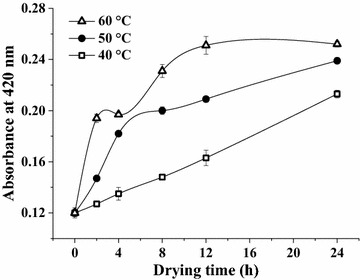
Absorbance of heat-dried RP extract at 420 nm

**Fig. 2:**
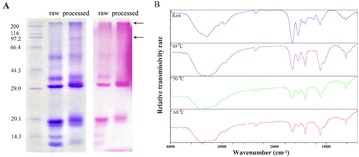
Glycation of RP protein. **A** SDS PAGE, **B** FTIR spectra

**Fig. 3:**
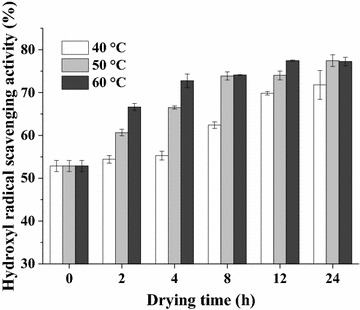
Hydroxyl radical scavenging activity of RP extract

**Conclusions:** The heat-drying process of RP could bring about Maillard reaction and the enhanced antioxidant activities may be attributed to the Maillard reaction products of free amino acids/proteins and saccharides.

**References**

1. Cao ZY, Chen XZ, Chang ET, Du J. Effective components of Chinese herbal compound decoction and Maillard reaction. Chin J Integr Med. 2009;15:224–8.
2. Kang KS, Yamabe N, Kim HY, Okamoto T, Sei Y, Yokozawa T. Increase in the free radical scavenging activities of American ginseng by heat processing and its safety evaluation. J. Ethnopharmacol. 2007;113:225–32.

## 121 Preparation and characterisation of glycopeptide analogues prepared by nonenzymatic glycation

### Xu Chen, Shaoyun Wang

#### College of Biological Science and Engineering, Fuzhou University, Fuzhou 350108, China

##### **Correspondence:** Shaoyun Wang - shywang@fzu.edu.cn

*Journal of Chinese Medicine* 2018, **13(Suppl 1):**121

**Background:** Antifreeze glycoprotein (AFGP) could be used for improving storage of blood, organs and tissues, preserving the textural quality of frozen foods, and protecting crops from freezing in a freeze–thaw cycle to prevent or minimize damage to cells and tissues [1, 2]. This study aimed to investigate the cryoprotective activities of antifreeze glycopeptide analogues (GoAPP) obtained by nonenzymatic glycation and possible action mechanism of GoAPP on Streptococcus thermophiles during cold stress.

**Materials and methods:** To prepare the glycopeptide analogues, various reaction time, temperature, pH and the mass ratio were used under various operating conditions. In order to understand the protective mechanism of GoAPP protectant, the impact on integrity permeability and microstructure of bacterial membrane, the glass transition temperature (Tg) and Thermal Hysteresis Activity (THA) of GoAPP were studied.

**Results:** The results indicated that under the best conditions, 7.3% (w/v) of antifreeze peptide, 2.7% (w/v) of dextran at 77 °C, pH 8.74 and 67 min of reaction time, a glycopeptide analogues was purified and submitted to characterization. Treatment of *S. thermophiles* with 5 mg/ml synthetic glycopeptide analogues led to 2.3-fold increased survival (p < 0.05). The leakage concentration of intracellular protein and nucleic acid were lesser in the GoAPP-treated group than those in the groups treated by glycerol, this indicated that GoAPP protectant maintained better membrance characteristics, prevented the leakage of intracellular proteins and nucleic acid (Fig. 1). Furthermore, the activities of lactate dehydrogenase and β-galactosidase were both higher in the GoAPP-treated group than those in control groups. Under the investigation of SEM, cells of GoAPP-treated group were significantly full and integral, while cells in control group were shrinking (Fig. 2). DSC curves of the freezing and melting processes for GoAPP and APP (original antifreeze peptide) indicated that the GoAPP group produce less ice nuclei than APP group in the equilibrium sample at the same holding temperature. Moreover, the addition of synthetic glycopeptide analogues (20 mg/ml) in deionized water increased the glass transition temperature (Tg) from − 27.6 to − 21.4 °C.

**Fig. 1:**
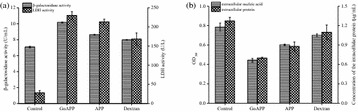
The interaction between GoAPP and cell membrane. **a** Effect of GoAPP on the activity of β-galactosidase and lactate dehydrogenase (LDH) during frozen. **b** Effect of GoAPP on the leakage of intracellular protein and nucleic acid during frozen

**Fig. 2:**
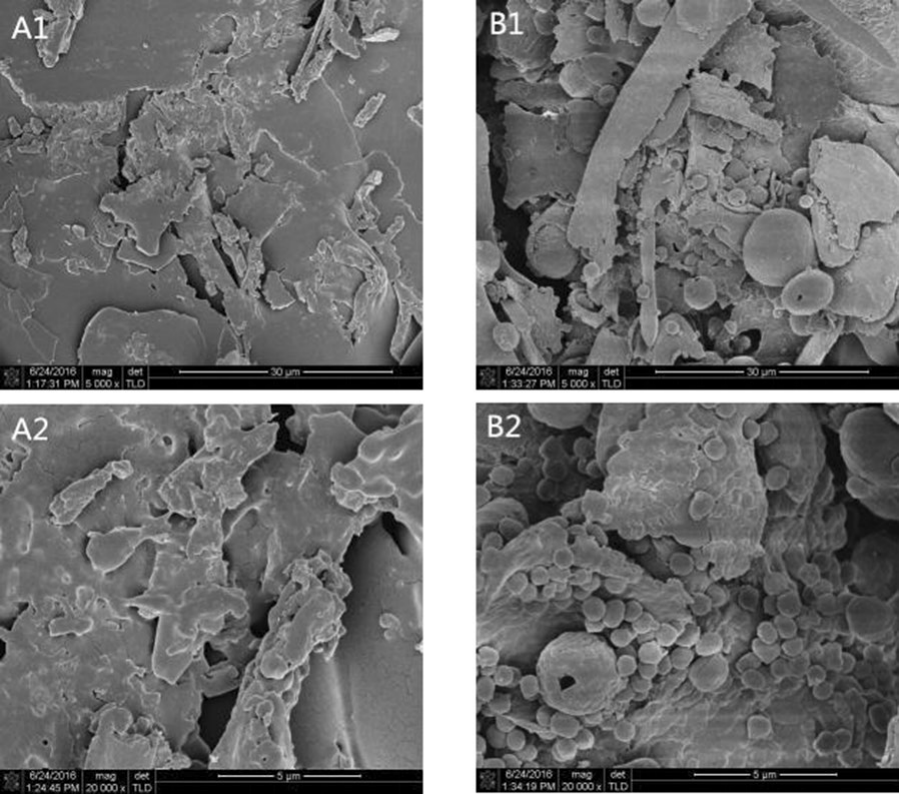
Scanning electron microscopy (SEM) images of freeze-dried *S. thermophiles*. Untreated cells show collapsed morphology at 5000-fold (A1) and 20,000-fold (A2) magnifications, respectively. GoAPP-treated cells show plump cell profile at 5000-fold (B1) and 20,000-fold (B2), respectively

**Conclusion:** The GoAPP were synthesized by modifying both the structure of the dextran moieties and the antifreeze peptide backbone, which could improve cell viability and maintaining the cell integrity. The results suggest that GoAPP could be potentially developed as a new and effective probiotics cryoprotectant that require low temperature storage.

**References**

1. Knight CA. Adding to the antifreeze agenda. Nature. 2000;406:249–51.
2. Wang SY, Damodaran S. Ice-structuring peptides derived from bovine collagen. J Agric Food Chem. 2009;57:5501–9.

## 122 A study on the potential of water extract of ginseng in the treatment of cancer cachexia

### Shuai Lu, Yubo Zhang, Yingqian Ci, Huajun Li, Mei Han

#### College of Chemistry, Beijing Normal University, Beijing, P. R. China

##### **Correspondence:** Mei Han - hanmei@bnu.edu.cn

*Journal of Chinese Medicine* 2018, **13(Suppl 1):**122

**Background:** Panax ginseng is one of the most commonly used herbal medicines around the world. It has potent anti-tumor, anti-oxidation, anti-inflammation, immune modulation properties [1]. Cancer cachexia is characterized by body weight loss (skeletal muscle mass loss), and cannot be completely reversed by common nutritional support [2]. More than 70–80% of advanced cancer patients develop cachexia, and nearly 20% of cancer deaths are directly caused by cachexia [3]. This study aimed to improve the physical condition and reduce the expression of inflammatory cytokine in experimental cancer cachexia mice by using water extract of ginseng (WEG).

**Materials and methods:** Male BALB/c mice implanted with colon-26 adenocarcinoma cells were selected to establish cancer cachexia model for assessing the effect of WEG on experimental cancer cachexia mice.

**Results:** The results indicated that gastrocnemius muscle from colon-26 bearing mice treated with WEG had significantly higher than untreated tumor-bearing mice (*p *< 0.01). In addition, colon-26 bearing mice treated with WEG show little side effect on heart, liver, spleen, lung, and kidney than untreated tumor-bearing mice. Further study will focus on inflammatory cytokine expression and NF-κB pathway activation.

**Conclusions:** In conclusion, WEG may be an important medicine in treatment of cancer cachexia.

**References**

1. Fearon K, Strasser F, Anker SD, Bosaeus I, Bruera E, Fainsinger RL, et al. Definition and classification of cancer cachexia: an international consensus. Lancet Oncol. 2011;12(5):489–95.
2. Gu C, Qiao J, Zhu M, Du J, Shang W, Yin W, et al. Preliminary evaluation of the interactions of Panax ginseng and Salvia miltiorrhiza bunge with 5-fluorouracil on pharmacokinetics in rats and pharmacodynamics in human cells. Am J Chinese Med. 2013;41(2):443–58.
3. Cong M, Zou B, Yu L. Mechanisms of anorexia cancer cachexia syndrome and potential benefits of traditional medicine and natural herbs. Curr Pharm Biotechnol. 2016;17(13):1147–52.

## 123 Natural fungicides obtained from *Linaria* species

### Sophie V. Sokornova, Tatiana V. Matveeva

#### Department of Genetics and Biotechnology, St. Petersburg State University, St. Petersburg, Russia

##### **Correspondence:** Sophie V. Sokornova - sokornova@bio.spbu.ru

*Journal of Chinese Medicine* 2018, **13(Suppl 1):**123

**Background:** In natural conditions, some of *Linaria* species are more resistant to fungal infection than other plants from Antirrhineae tribe [1]. Toadflax plants from sections *Linaria* and *Speciosae* contain DNA sequences similar to *Agrobacterium rhizogenes* T-DNA in their genomes [2]. One of the possible advantages of the T-DNA-containing plants is enhanced secondary metabolite production [3]. This feature may play role in the resistance of plants to phytopathogens [4]. Plants possess a wide range of protective mechanisms against disease, including several types of pre-formed and infection-induced compounds include phenols/polyphenols (47%), terpenoids (29%), alkaloids (11%) [5]. The most of *Linaria* species appeared to have high levels of iridoid glycosides, that perform the plant protective function [6–8]. In addition, *L.* *vulgaris* shows high content of flavonoids with fungicide activity. The aim of this work is to identify biological activity of major secondary metabolites in *Linaria* species, representing three sections, growing in vitro conditions.

**Materials and methods:** Seeds of *L. vulgaris, L. genistifolia, L. maroccana* were sterilized with H_2_O_2_ and placed on Murashige and Scoog [9] medium for germination. Then plants were propagated by cuttings and cultivated at 24-h photoperiod. Fresh aerial parts of plants were extracted with MeOH for 1 h in an ultra-sonic bath. The crude extract was fractionated with chloroform: methanol in silica gel filled column under vacuum. The fraction was filtered through a Minisart filter type RC-0.45 µm, dried and analyzed on GC–MS. The TLC solvent system used was ethyl acetate–methanol–water (38.5:7.5:4). Fungicide activity of extracts was detected by classical agar disk-diffusion method (500 mg/ml crude extract, 50 mg/ml fraction) and TLC–bioautography method [10].

**Results:** Crude aqueous/alcoholic extract from *L.* *vulgaris* demonstrated higher fungicide activity than extracts from other species. The highest activity demonstrated antirride enriched fraction (chloroform:methanol 4:1). Additionally, TLC–bioautography test have shown the spot with fungicide activity. In case of detection by anisaldehyde the spot had yellow color indicating the presence of flavonoids. The crude extract of *L.* *genistifolia* has also demonstrated fungicide activity. However, fraction with fungicide activity was blue and had another retention factor. Perhaps this substance is genestifolioside, but further clarification is required. Thus, the spectrum of the secondary metabolites that provide resistance to plant diseases has individual features in different *Linaria* species.

**Conclusion:** *Linaria* species produced high levels of secondary metabolites with fungicide activity, that can be used in pharmacology.

This work was supported by RSF grant 16-16-10010.

**References**

1. Sokornova SV, Gasich EL, Matveeva TV, Afonin NA. Micromycetes of plants *Linaria* containing DNA sequences of Agrobacterial origin in their genomes. Mikologia i Fitopatologia. 2015;49(3):188–93.
2. Matveeva TV, Bogomaz DI, Pavlova OA Nester EW, Lutova LA. Horizontal gene transfer from genus *Agrobacterium* to the plant *Linaria* in nature. Mol Plant Microbe Interact. 2012;25:1542–51.
3. Chandra S. Natural plant genetic engineer *Agrobacterium rhizogenes*: role of T-DNA in plant secondary metabolism. Biotechnol Lett. 2012;34(3):407–15.
4. Matveeva TV, Lutova LA. Horizontal gene transfer from *Agrobacterium* to plants. Front Plant Sci. 2014;5:326.
5. Boulogne E, Petit P, Ozier-Lafontaine H, Desfontaines L, Loranger-Merciris G. Insecticidal and antifungal chemicals produced by plants: a review. Environ Chem Lett. 2012;10:325–47.
6. Matveeva TV, Sokornova SV, Lutova LA. Influence of *Agrobacterium* oncogenes on secondary metabolism of plants. Phytochem Rev. 2015;14:541–54.
7. Ahmad VU, Kousar F, Zubair M, Desfontaines L, Loranger-Merciris G. A new iridoid glycoside from *Linaria genestifolia.* Fitoterapia. 2006;77:12–14.
8. Otsuka H. Iridoid glucosides from *Linaria japonica*. Phytochem. 1993;33(3):617–22.
9. Murashige T, Skoog F. A revised medium for rapid growth and bio assays with Tobacco tissue cultures. Physiologia Plantarum. 1962;15:473–97.
10. Kagan IA, Flythe MD. Thin-layer chromatographic (TLC) separations and bioassays of plant extracts to identify antimicrobial compounds. Compounds J Vis Exp. 2014;85:e51411.

## 124 In vitro antioxidant activity and in vivo anti-fatigue effect of sea horse (*Hippocampus*) peptides

### Zebin Guo^1,2^, Duanquan Lin^1,2^, Juanjuan Guo^1,2^, Yi Zhang^1,2^, Baodong Zheng^1,2^

#### ^1^College of Food Science, Fujian Agriculture and Forestry University, Fuzhou, Fujian, 350002, China; ^2^Engineering Research Center of Marine Living Resources Integrated Processing and Safety Risk Assessment, Fuzhou, Fujian 350002, China

##### **Correspondence:** Baodong Zheng

*Journal of Chinese Medicine* 2018, **13(Suppl 1):**124

**Background:** *Hippocampus* belongs to the *Syngnathidae* family and provides a source of traditional Chinese medicine materials. *Hippocampus* is rich in proteins and essential amino acids. It is a high-quality material for preparation of proteins and related products. Moreover, previous studies have reported that a high ratio of heterocyclic or aromatic (i.e. His, Pro, Tyr, and Phe) and acidic (i.e. Glu and Asp) amino acids accounted for 16.14 and 20.09% of the total amino groups in *Hippocampus*, respectively. However, there is further study about the use of *Hippocampus* polypeptide for anti-fatigue treatment. In this study, the optimal enzymatic hydrolysis conditions for preparation of *Hippocampus* polypeptide and its anti-fatigue activity were investigated.

**Materials and methods:** This study investigated changes the in vitro antioxidant activity of *Hippocampus* polypeptides during enzymatic hydrolysis, including the effects of enzyme species, enzyme concentration, material–liquid ratio, hydrolysis time, pH, and temperature of the reaction system. Its in vivo anti-fatigue activity was also studied.

**Results and conclusions:** *Hippocampus* peptide prepared by papain digestion exhibited the highest 1,1-diphenyl-2-picryl-hydrazyl free radical scavenging rate (71.89 ± 1.50%) and strong hydroxyl radical scavenging rate (75.53 ± 0.98%), compared to those prepared by five other commonly used enzymes (i.e., trypsin, neutral protease, compound protease, flavorzyme, and alkaline protease). Additionally, maximum antioxidant activity of *Hippocampus* polypeptide prepared by papain digestion was reached after hydrolysis for 40 min at pH 6.0 and 60 °C of the reaction system by using 2000 U/g enzyme and a material–liquid ratio of 1:15. Moreover, compared with the control group, *Hippocampus* peptide prolonged the swimming time by 33–40%, stabilized the blood glucose concentration, increased liver glycogen levels, and decreased blood lactate levels and blood urea nitrogen levels in mice (*p* < 0.01). In conclusion, these results indicated that *Hippocampus* polypeptide prepared by papain digestion under optimal conditions exhibited high degrees of antioxidant and anti-fatigue activity.

## 125 A new cytotoxic biflavone from *Selaginella doederleinii*

### Zhen-Xing Zou^1^, Kang-Ping Xu^2^, Guo-Gang Zhang^1^, Ping-Sheng Xu^1^, Gui-Shan Tan^1^

#### ^1^Xiangya Hospital of Central South University, Changsha 410008, China; ^2^Xiangya School of Pharmaceutical Sciences, Central South University, Changsha 410013, China

##### **Correspondence:** Zhen-Xing Zou; *Gui*-*Shan Tan*

*Journal of Chinese Medicine* 2018, **13(Suppl 1):**125

**Background:** *Selaginella doederleinii*, belonging to the genus *Selaginella*, is widely distributed in Guangxi Zhuang Autonomous Region, Guizhou and Yunnan provinces of mainland China [1]. Traditionally, the whole plant has been used as a folk medicine to treat some kinds of cancers, sore throat and rheumatoid arthritis [2]. In our previous work, some unique flavonoids were reported in this plant [3–5]. As part of ongoing search for novel and bioactive flavones from this genus, 75% aqueous ethanol extract from the whole herbs of *Selaginella doederleinii* was isolated.

**Materials and methods:** The whole herbs of *S. doederleinii* were collected in the town of Wutong, Lingui district, Guangxi, China, in July 2013 and authenticated by Prof. ZhenJi Li (Xiamen University, Xiamen, China). A botanical specimen of this species (20130710) was deposited at the Xiangya School of Pharmaceutical Sciences, Central South University. Structures of **1**–**5** were determined by extensive spectroscopic methods including NMR and HRMS. All compounds were evaluated for their in vitro cytotoxicity against three human cancer cell lines A549, MCF-7, and SMMC-7721. The cytotoxicity assay was performed using MTT (3-(4, 5-dimethylthiazol-2-yl)-2, 5-diphenyltetrazolium bromide) method in 96-well microplates [6].

**Results:** A new biflavone, doederbiflavone A (**1**), together with four known ones (**2**–**5**) were isolated from *S. doederleinii* (Fig. 1). Compound **1** features a unique dimeric skeleton with apigenin and chrysin units, which was first discovered in nature, and exhibited significant cytotoxicity against A549 with IC_50_ value of 1.78 μM.

**Fig. 1:**
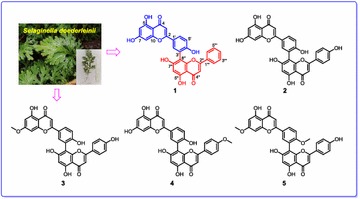
Structures of compounds **1**–**5**

**Conclusions:** Compound **1** may be a promising compound for treating lung cancer.

**References**

1. Nanjing University of Chinese Medicine, Zhong Yao Da Ci Dian. Shanghai: Shanghai Scientific & Technical Publishers; 2006. p. 831–2.
2. State Administration of Traditional Chinese Medicine, Zhong Hua Ben Cao. Shanghai: Shanghai Scientific & Technical Publishers; 1998. p. 44.
3. Zou ZX, Xu PS, Zhang GG, Cheng F, Chen K, Li J, Zhu WX, Cao DS, Xu KP, Tan GS. Selagintriflavonoids with BACE1 inhibitory activity from the fern *Selaginella doederleinii*, Phytochemistry. 2017;134:114–21.
4. Zou ZX, Xu KP, Xu PS, Li XM, Cheng F, Li J, Yu X, Cao DS, Li D, Zeng W, Zhang GG, Tan GS. Seladoeflavones A–F, six novel flavonoids from *Selaginella doederleinii*. Fitoterapia. 2017;116:66–71.
5. Zou ZX, Tan GS, Zhang GG, Yu X, Xu PS, Xu KP. New cytotoxic apigenin derivatives from *Selaginella doederleinii*. Chin Chem Lett. 2017;28:931–4.
6. Mosmann T. Rapid colorimetric assay for cellular growth and survival: application to proliferation and cytotoxicity assays. J Immunol Methods. 1983;65:55–63.

## 126 Fucoidans from *Enteromorpha prolifera* for their anti-diabetic effect in streptozotocin/high fat diet-induced diabetic mice

### Xin Yan^1^, Yuqing Chen^1,3^, Sinan Yuan^1^, Yangcheng Feng^1,3^, Luan Lin^1,2^, Bin Liu^1^, Chao Zhao^1,2^

#### ^1^College of Food Science, Fujian Agriculture and Forestry University, Fuzhou 350002, China; ^2^Fujian Province Key Laboratory for the Development of Bioactive Material from Marine Algae, Quanzhou Normal University, Quanzhou 362000, China; ^3^College of Food Science and Nutritional Engineering, China Agricultural University, Beijing 100083, China; ^4^Department of Chemistry, University of California, Davis 95616, CA, USA

##### **Correspondence:** Chao Zhao - zhchao@live.cn

*Journal of Chinese Medicine* 2018, **13(Suppl 1):**126

**Background:** The green algae belonging to *Enteromorpha* species are widely distributed in China and have been used as drug in traditional Chinese medicine for hundreds of years [1, 2]. A detailed investigation was carried out to examine the antidiabetic activities of fucoidans from *Enteromorpha prolifera* (EPOs) in streptozotocin/high fat diet-induced diabetic mice.

**Materials and methods:** *E. prolifera* collected in the coast of Qingdao, China that was chosen. EPOs was prepared from polysaccharides by ultrasonic hydrolysis at 60 °C and then filtrated by 3 kDa Millipore for use. The hypoglycemic activity of EPOs were evaluated in diabetic mice in vivo. Thirty Kunming mice (40–43 g) were divided randomly into three groups of ten mice each: diabetic control group, nondiabetic control group, and EPOs-treated group. Mice were intraperitoneally injected with 15 mg/mL streptozotocin (300 mg/kg). All animals received oral dosing daily for 28 days.

**Results:** Body weights of EPOs group mice were lower than that of normal mice. The blood sugar was reduced by 28.03% in EPOs group. Effects of EPOs on oral glucose tolerance in diabetic mice were determined. Two hours after sugar administration, the blood glucose concentrations had returned to normal or were lower than before the administration. Photomicrographs of liver sections stained by hematoxylin–eosin staining in different experimental groups were examined. The nondiabetic control group revealed normal hepatic structure and lobules. Each lobule was made up of radiating plates, strands of cells forming a net-work around a central vein. By contrast, diabetic group showed distended cells, loose cytoplasm, and loss of normal architecture such as disarrangement of hepatocytes in the liver. Morevoer, there were fat vacuoles in some hepatocytes of diabetic group. EPOs group showed normal strands of cells forming a net-work, some hydropic hepatocytes and there is lesser fat vacuoles and distended cells than model group.

**Conclusions:** These results indicated an effective relationship between low molecular weight fucoidans from *E. prolifera* and hypoglycemia in patients with diabetes mellitus. *E. prolifera* oligosaccharides can be developed into hypoglycemic drugs or health food.

**Acknowledgements:** This work was financially supported by Natural Science Foundation (2016J06009) of Fujian Province, China and Fujian Province Key Laboratory for the Development of Bioactive Material from Marine Algae grant (2017FZSK05). The project was supported by FAFU Grants KXb16011A and 612014043.

**References**

1. Zhao C. Res J Biotechnol. 2014;9(1):30–6.
2. Zhao C, Wu YJ, Yang CF, Liu B. Int J Food Sci Tech. 2015;50(8):1705–17.

## 127 Extraction and isolation of polyphenol from *Lepidium meyenii* leaves and its antioxidant activity

### Jicheng Chen^1^, Jianbo Xiao^3^, Shun Gao^2^

#### ^1^College of Food Science, Fujian Agriculture and Forest University, Fuzhou, 350002, China; ^2^Institute of Ecological Forestry, Faculty of Forestry, Sichuan Agricultural University, 611130, Chengdu, China; ^3^Institute of Chinese Medical Sciences, State Key Laboratory of Quality Research in Chinese Medicine, University of Macau, Avenida da Universidade, Taipa, Macau

##### **Correspondence:** Shun Gao - shun1220@yahoo.com

*Journal of Chinese Medicine* 2018, **13(Suppl 1):**127

**Background:** The utilization of wastes of agricultural and industrial residues as a source of functional ingredients is of great interest. *Lepidium meyenii* leaves are a byproduct of *L. meyenii* and contain good-quality extractable polyphenols.

**Materials and methods:** The effects of ethanol concentration, mixing time, solvent/meal ratio and temperature were studied on the extraction of polyphenols from *L. meyenii* leaves. Moreover, the isolation steps and antioxidant activity of *L. meyenii* leaves polyphenols were also designed.

**Results:** Results suggested that polyphenols yield was significantly affected by ethanol concentration, mixing time, and solvent/meal ratio. Based on the screening results of extraction parameters, a central composite design was used with three variables: ethanol concentration (20, 40, and 60%), extraction time (30, 90 and 150 min); and solvent/meal ratio (10:1, 25:1 and 40:1). Selected response which evaluates the extraction process was polyphenol yield and the second-order model obtained for polyphenol yield revealed coefficient of determination of 98.8%. Polyphenol yield obtained ranged from 27.83 to 30.65 mg/g. Maximum yield (30.86 mg/g) was obtained when ethanol concentration, extraction time and solvent/meal ratio were 47.78%, 76.99 min and 1: 21.6, respectively. Experiments at optimum conditions gave a polyphenol yield of 30.36 mg/g, suggesting that the relative error of predictive value is only 1.62%. According to the optimum extraction parameters, the isolation and purification steps consisted of extracting polyphenol in 47.78% ethanol, followed by centrifugation, plate filtration, ultrafiltration, and vacuum concentration. 2.58 g of polyphenols may be obtained from 100 g leaves, and the recovery rate reached 85.1%. Results from antioxidant activity tests showed that the polyphenol may effectively scavenge DPPH free radical, ·OH and O_2_^−·^, and the dose–effect relation is notable. The free radical scavenging ability of *L. Meyenii* leaves polyphenol was higher than that of BHT. Moreover, the scavenging ability are different, and the capacities from strong to weak are DPPH free radical, O_2_^−·^and ·OH.

**Conclusions:** These findings will help to design the process of optimal polyphenol extraction and isolation from *L. meyenii* leaves.

## 128 Determination and evolution of sesame lignans in *Monascus* aged vinegar employing microwave irradiation

### Yazhen Chen, Jicheng Chen, Jingjing Tian, Huifang Ge, Hetong Lin

#### College of Food Science, Fujian Agriculture and Forestry University, Fuzhou, Fujian, 350002, China

##### **Correspondence:** Jicheng Chen - newtaicjc@163.com; Hetong Lin - hetonglin@126.com

*Journal of Chinese Medicine* 2018, **13(Suppl 1):**128

**Background:** To establish a HPLC method for the simultaneous analysis of three sesame lignans including sesamol, sesamin and sesamolin in *Monascus* aged vinegar. In addition, the developed method was applied for investigating the conversion from sesamolin to sesamol over microwave irradiation.

**Materials and methods:** The chromatography was performed on Waters XBridge C18 reversed phase column (250 mm × 4.6 mm, 5 μm) with methanol–water (gradient elution) as the mobile phase at 40 °C. The detection wavelength were 295 nm for sesamol, 287 nm for sesamin and sesamolin, and the flow rate was 0.8 mL/min.

**Results:** The limits of quantification (LOQ) were 0.09 µg/mL for sesamol, 0.25 µg/mL for sesamin and 0.27 µg/mL for sesamolin. The calibration curves showed a good linearity ranges in the range of 0.5–200 µg/mL and the correlation coefficients were greater than 0.999 for the three compounds. The average recoveries of sesamol, sesamin and sesamolin were 103.03, 101.63 and 97.94%, with an average RSD of 1.24, 1.60 and 1.56%, respectively.

**Conclusions:** The method was tested to be simple, accurate and reproducible. The results suggested that the content of sesamol was significantly correlated with that of sesamolin in vinegar. There is probable a transformation from sesamolin to sesamol in vinegars.

## 129 Determination and evolution of sesame lignans in *Monascus* aged vinegar employing microwave irradiation

### Yazhen Chen, Jicheng Chen, Jingjing Tian, Huifang Ge, Hetong Lin

#### College of Food Science, Fujian Agriculture and Forestry University, Fuzhou, Fujian, 350002, China

##### **Correspondence:** Jicheng Chen - newtaicjc@163.com; Hetong Lin - hetonglin@126.com

*Journal of Chinese Medicine* 2018, **13(Suppl 1):**129

**Background:** To establish a HPLC method for the simultaneous analysis of three sesame lignans including sesamol, sesamin and sesamolin in *Monascus* aged vinegar. In addition, the developed method was applied for investigating the conversion from sesamolin to sesamol over microwave irradiation.

**Materials and methods:** The chromatography was performed on Waters XBridge C18 reversed phase column (250 mm × 4.6 mm, 5 μm) with methanol–water (gradient elution) as the mobile phase at 40 °C. The detection wavelength was 295 nm for sesamol, 287 nm for sesamin and sesamolin, and the flow rate was 0.8 mL/min.

**Results:** The limits of quantification (LOQ) were 0.09 µg/mL for sesamol, 0.25 µg/mL for sesamin and 0.27 µg/mL for sesamolin. The calibration curves showed a good linearity ranges in the range of 0.5–200 µg/mL and the correlation coefficients were greater than 0.999 for the three compounds. The average recoveries of sesamol, sesamin and sesamolin were 103.03, 101.63 and 97.94%, with an average RSD of 1.24, 1.60 and 1.56%, respectively.

**Conclusions:** The method was tested to be simple, accurate and reproducible. The results suggested that the content of sesamol was significantly correlated with that of sesamolin in vinegar. There is probable a transformation from sesamolin to sesamol in vinegars.

## 130 Enhancement of tetramethylpyrazine in traditional vinegar under different accelerated transformation approaches

### Jicheng Chen, Jingjing Tian, Yazhen Chen, Huifang Ge

#### College of Food Science, Fujian Agriculture and Forest University, No.15 Shangxiadian Rd, Fuzhou, 350002, China

##### **Correspondence:** Jicheng Chen - newtaicjc@163.com

*Journal of Chinese Medicine* 2018, **13(Suppl 1):**130

**Background:** Addressing the enhancement of characteristic flavors have attracted a great deal of scientific and industrial attention. Tetramethylpyrazine (TMP) is a crucial flavor and bioactive compound found in Chinese black vinegar. In order to enrich TMP and improve the quality of the vinegars, the different accelerated approaches were employed for transformation.

**Methods:** The effects of temperature, time, pH value of the conversion conditions on TMP were investigated, meanwhile the improvement of different nitrogen substrates and treatment by high voltage pulsed electric field (PEF) were involved.

**Results:** The results demonstrated that the formation of TMP was significantly affected by temperature, time, pH value of the conversion, and especially by substrates like ammonium acetate and Lysine. The content of TMP in vinegars under a elevated temperature were maximum increased by 13.15 μg/mL at 75 °C. Similarly, TMP also demonstrated in a time-dependent manner. The effect of pH value on TMP content was less significant, increased within 0.27–5.72 μg/mL. The TMP content was increased by 7.19 and 11.21 μg/mL, respectively, while strengthening nitrogen substrates by Lysine and ammonium acetate.

**Conclusions:** Application of PEF in accelerated ageing is a novel vinegar processing method. The content of TMP in PEF-processed vinegars was significantly increased in several times. The formation mechanism of TMP during the PEF processing need further investigate. However, there is no doubt that PEF processing is a potential application in the vinegar industry.

## 131 Evaluation of antioxidant activities of ethanol extract from *Ligusticum* subjected to in vitro gastrointestinal digestion

### Huifang Ge, Jicheng Chen, Yazhen Chen, Jingjing Tian, Xiaofeng Liang, Lei Chen

#### College of Food Science, Fujian Agriculture and Forestry University, Fuzhou, Fujian 350002, China

##### **Correspondence:** Jicheng Chen - newtaicjc@163.com

*Journal of Chinese Medicine* 2018, **13(Suppl 1):**131

**Background:** The Rhizome of *Ligusticum chuanxiong* Hort,a traditional Chinese medicine widely used to treat cardiovascular diseases and attenuate oxidative stress. The main bioactive compounds including tetramethylpyrazine (TMP), polyphenols, ferulic acid have been reported to be responsible for these effects. This paper was to evaluate the influence of *Ligusticum chuanxiong* extraction (LCE) in mimic gastrointestinal tract on antioxidant activity.

**Materials and methods:** Antioxidant activities of LCE in mimic gastrointestinal were performed by free radical-scavenging capacity, T-SOD and the cellular antioxidant activity (CAA). TMP, ferulic acid and its esters were determined by HPLC method, meanwhile the total phenolic acid, free and bound phenolics were also analyzed.

**Results:** The effects of gastric digestion group metabolic liquid on free radical scavenging followed as DPPH > ·O^2−^ > ·OH, while the clearance effects of intestine digestion group expressed as ·O^2−^ > ·OH > DPPH. Furthermore, the digested extraction promoted lower cellular antioxidant activity (CAA) with dose–response correlations. Gastrointestinal digestion increased the release of bound ferulic acids and polyphenols. Content of ferulic acid in gastric and intestinal metabolic solution increased from 6.07 to 9.33 and 14.17 mg/g. The free phenolic before and after digestion were 177.38, 179.69 and 194.99 mg/g, respectively.

**Conclusions:** The simulated gastrointestinal digestion of LCE promoted a significant increase in the free phenolic acids content, antioxidant activity and CAA. This study develops a way for the systematic scientific efficacy of traditional Chinese medicine, and also provides a References for investigation of possible mechanisms of multiple components.

## 132 Optimization of extraction technology conditions of *Enteromorpha prolifera* polysaccharides using response surface methodology and its antiviral activity against enterovirus 71

### Luying Gao^1^, Jingxue Zhou^2^, Yuqing Chen^2^, Chengfeng Yang^2^, Luan Lin^2,3^, Bin Liu^2^, Li Zhang^1^, Chao Zhao^2,3,4^

#### ^1^Department of Paediatrics, Nanjing First Hospital, Nanjing Medical University, Nanjing 211166, China; ^2^College of Food Science, Fujian Agriculture and Forestry University, Fuzhou 350002, China; ^3^Fujian Province Key Laboratory for the Development of Bioactive Material from Marine Algae, Quanzhou Normal University, Quanzhou 362000, China; ^4^Department of Chemistry, University of California, Davis 95616, CA, USA

##### **Correspondence:** Chao Zhao - zhchao@live.cn; Li Zhang - Jiangsunjzhangli2003@sina.com

*Journal of Chinese Medicine* 2018, **13(Suppl 1):**132

**Background:** Enterovirus 71 (EV71), a member of the Enterovirus genus in the family *Picornaviridae*, is the most frequently detected pathogen in patients suffering from the hand-foot-and-mouth disease [1]. Green algae *Enteromorpha* species have long been used not only as a source of food but medicinal resource [2]. However, it has not been reported on its antiviral activity against EV71.

**Materials and methods:** *Enteromorpha prolifera* polysaccharides (EPS) were obtained through water extraction and ethanol precipitation. Based on the effect of single factor solid–liquid ratio, temperature, time and concentration ratio on yield of EPS, response surface methodology was used to optimize the extraction process. MTT colorimetric assay was conduct to study the effect of EPS on the Vero cells infected by EV71 virus.

**Results:** The results showed that the optimal EPS extraction conditions were as follows: extraction temperature of 90 °C, extraction time of 3 h, solid–liquid ratio of 1:30 and concentration ratio of 3:1, under which the yield of EPS was 8.05%. EPS contained 31.4% of total sugar, 11.9% of uronic acid, 5.5% of protein, and 10.2% of sulfates. MTT assay indicated that EPS can inhibit the replication of EV71 and lead to decreased content of EV71 in Vero cells. In vitro cytotoxicity was also determined. The in vitro antiviral activity was determined via crystal violet staining assay. At the same time, time-of-drug addition assay were used to find that EPS was more effective when added during EV71 infection. EPS inhibited EV71 replication and showed that it might block viral polyprotein expression and genomic RNA synthesis.

**Conclusions:** The results demonstrated that EPS could effectively protect Vero cells from infecting and it has antiviral function during EV71-early-stage replication. *E. prolifera* polysaccharides could be valuable as the potentially anti-EV71 therapeutic compounds.

**Acknowledgements:** This work was financially supported by Natural Science Foundation (2016J06009) and Marine High-tech Industrial Development Project (MIN2014-17) of Fujian Province, China. The project was also supported by Fujian Province Key Laboratory for the Development of Bioactive Material from Marine Algae grant (2017FZSK05) and FAFU grant KXb16011A.

**References**

1. Zhao C, Gao LY, Wang CY, Liu B, Jin Y, Xing Z. Carbohydr Polym. 2016;144:382–9.
2. Zhao C, Wu YJ, Yang CF, Liu B, Int J Food Sci Tech. 2015;50(8):1705–17.

## 133 Phytochemicals in hops in preventing and managing of metabolic syndrome

### Pavel Dostálek, Marcel Karabín, Lukáš Jelínek

#### Department of Biotechnology, University of Chemistry and Technology, Prague, Technická 5, 16628 Prague 6, Czech Republic

##### **Correspondence:** Pavel Dostálek - Pavel.Dostalek@vscht.cz

*Journal of Chinese Medicine* 2018, **13(Suppl 1):**133

**Background:** In many countries, the prevalence of metabolic disease has attained epidemic proportions because of cardiovascular complications and mortality. A metabolic syndrome is associated with risk from 5 factors: abdominal (central) obesity, elevated blood pressure, elevated plasma glucose, high serum triglycerides, and low high-density lipoprotein levels. Treatment is focused on reduction of the risk of heart disease by lowering LDL cholesterol and reducing high blood pressure, and then on treatment of diabetes. Very important is a reduction in weight by proper diet and exercise [1].

**Results:** Beneficial effects of hop phenolic compounds for diabetic patients consist of improving blood glucose and lipid profiles, and reducing insulin resistance. Phenolic compounds exert antiobesity effects that are closely linked with antioxidant effects, through their ability to modulate lipid and energy metabolism and thus enable weight loss and reduce obesity. Regulation of cholesterol metabolism by phenolic compounds may reduce certain factors linked with hypercholesterolemia and dyslipidemia. Prenylflavonoids such as xanthohumol have been demonstrated to have strong antiobesity activities including the ability to inhibit diacylglycerol acyltransferase in rat liver, to inhibit triglyceride transport using a HepG2 cell line, and to inhibit the secretion of apolipoprotein B, the main constituent of the cholesterol LDL fraction. Therapeutic potential of polyphenols as treatment for obesity is also associated with the ability to inhibit α-glucosidase, an enzyme that controls blood glucose levels [1, 2]. Iso-α-acids are able to improve health by influencing lipid metabolism, glucose tolerance, and body weight. Diabetic mice treated with iso-α-acids showed reduced plasma glucose, triglyceride, and free fatty acid levels by 65.3, 62.6, and 73.1%, respectively. When mice were fed hop iso-α-acids in their diet, with high levels of cholesterol, an increase in plasma HDL-cholesterol and a reduction in cholesterol and triacylglycerol content in the liver were observed. The modulatory effect of iso-α-acids on lipid metabolism may also be responsible for lost body weight [1].

**Conclusions:** For treatment of metabolic syndrome should be very effective hop substances from group of polyphenols and bitter acids.

**References**

1. Karabín M, Hudcová T, Jelínek L, et al. Compr Rev Food Sci Food Saf. 2016;15:542–67.
2. Karabín M, Hudcová T, Jelínek L, et al. Biotechnol Adv. 2015;33:1063–90.

## 134 Efficacy of chitosan-phytochemicas coating on shelf-life extension of refrigerated hairtail (*Trichiurus japonicus*) fillets

### Wu Xianhui^1,2^, Ma Tengfeng^1,2^, Pang Jie^1^, Wu Chunhua^1^

#### ^1^College of Food Science, Fujian Agriculture and Forestry University, Fuzhou 350002, China; ^2^Ningde Vocational and technical College, Ningde 355000, China

##### **Correspondence:** Pang Jie - pang3721941@163.com; Wu Chunhua - chwu0283@163.com

*Journal of Chinese Medicine* 2018, **13(Suppl 1):**134

**Background:** As one of the most popular fish species to consumers, the hairtail *(Trichiurus japonicus)* is full of high quality protein and unsaturated fatty acids with high nourishment value. However, just because of its abundant nutrition, its flesh is highly perishable during postmortem aging. Thus, the development of effective preservation technologies to extend the shelf life and maintain the nutritional value as well as the flavor of hairtail products has become increasingly necessary. Biopolymer-based edible coating materials have several functions as barriers against water loss, gas exchange, flavor loss and microbial growth, thereby improving food quality and enhancing the shelf-life of food products. Among these biopolymers, chitosan is a good candidate for edible coating materials. The incorporation of phytochemicas into chitosan coatings may not only enhance the antimicrobial properties of the coating but also reduce its’ water vapor permeability and slow lipid oxidation of the product.

**Materials and methods:** Three different preservatives were applied to handle the refrigerated hairtail by chitosan and chitosan mixed with gallic acid in this paper. The preservation effect was investigated.

**Results:** According to several physical and chemical indexes such as the content of TVBN, total numbers of colony, the content of TBA and pH value, the freshness preservation effect of hairtail was evaluated. The result shows that the POV value, TBA value, K value, TVBN content and numbers of colony of samples with preservatives is obvious lower than that of the control group. Also, after processing with compound preservatives 6d storage, all physical and chemical indexes of hairtail is lower than that of the chitosan group.

**Conclusions:** The application of chitosan mixed with phytochemicas preservatives is effective and showed great potential in the fish storage.

**Acknowledgements**: The authors would like to thank the support of Natural Science Foundation of Fujian Province of China (2014J01384 and 2017J01155).

## 135 Pretreatment of *Siegesbeckia orientalis* L. extract effectively prevents and alleviates the postoperative systemic inflammation in mice

### John M. T. Chu^4,5^, Wei Sang^1^, Hongxun Tao^1^, Zhangfeng Zong^1^, Wei Xiong^1^, Yitao Wang^1^, Gordon T. C. Wong^4,5^, Hua Yu^1,2,3^

#### ^1^Institute of Chinese Medical Sciences, State Key Laboratory of Quality Research in Chinese Medicine, University of Macau, Macao, China; ^2^HKBU Shenzhen Research Center, Shenzhen, Guang Dong, China; ^3^School of Chinese Medicine, Hong Kong Baptist University, Kowloon Tong, Hong Kong, China; ^4^Department of Anaesthesiology, LKS Faculty of Medicine, The University of Hong Kong, Hong Kong, China; ^5^Research Centre of Heart, Brain, Hormone and Healthy Aging, LKS Faculty of Medicine, The University of Hong Kong, Hong Kong, China

##### **Correspondence:** Hua Yu - bcalecyu@umac.mo; Gordon T. C. Wong - gordon@hku.hk

*Journal of Chinese Medicine* 2018, **13(Suppl 1):**135

**Background:** Systemic inflammation is a physiological defense in response to trauma or infection to the body, as may occur in the post operative period. An appropriate inflammatory response is necessary for the organism’s healing potential and facilitates tissue repair. However, an excessive or prolonged response might induce to critical complications and even to organ failure. Therefore an effective means of modulating postoperative inflammation would be most useful and beneficial for clinicians and patients.

**Methods:** *Siegesbeckia orientalis* L. (SO), also called “*Xixiancao*” in China, is a traditional herbal medicine and has been widely used for the treatment and management of various chronic inflammatory diseases (such as arthritis, and ulcerative colitis, etc.) in China. Previously, the extract of SO (SOE) has been reported to present potential anti-inflammatory activities both in vitro and in vivo. In the current study, the preventive and alleviative effects of SOE on postoperative systemic inflammation in male C57BL/6 mice (12 weeks age) were investigated and evaluated. After pretreated with SOE (equaled to 10 g herb/kg/day, p.o.) for 2 weeks, the mice were underwent laparotomy (which mimics the major surgery clinically), and then allowed to recovery for 3 days. Subsequently, the mice were sacrificed and the levels of pro-inflammatory cytokines in liver and serum were examined by Multiplex ELISA, respectively.

**Results:** The results indicated that the surgery triggered a pro-inflammatory response in mice and up-regulated the levels of inflammatory cytokines in mice liver and serum. However, pretreatment of SOE significantly attentuated the surgical-induced increase of Interleukin-6 (IL-6) and IL-8 levels both in mice liver and serum.

**Conclusions:** Our pilot data suggested that SO might be beneficial in postoperative model by improving postoperative inflammation and prevent subsequent complications.

**Acknowledgements:** This study was financially supported by the opening fund of the State Key Laboratory of Quality Research in Chinese Medicine of University of Macau (No. SKL-QRCM-2014-2016), the Research Committee of the University of Macau (SRG2015-00060-ICMS-QRCM) and the National Natural Science Foundation of China (NSFC, No. 81470170).

## 136 Preparation of chitosan-epigallocatechin gallate nanoparticles and their application in the preservation of aquatic products

### Ma Tengfeng^1,2^, Wu Xianhui^1,2^, Pang Jie^1^, Wu Chunhua^1^

#### ^1^College of Food Science, Fujian Agriculture and Forestry University, Fuzhou 350002, China; ^2^Ningde Vocational and technical College, Ningde 355000, China

##### **Correspondence:** Pang Jie - pang3721941@163.com; Wu Chunhua - chwu0283@163.com

*Journal of Chinese Medicine* 2018, **13(Suppl 1):**136

**Background:** Epigallocatechin gallate (EGCG) found in green tea have attracted considerable attention due to their favourable biological properties. However, their potential application is limited by their low stability. To improve its efficiency, the EGCG was encapsulated on chitosan nanoparticles (NPs) in this study.

**Materials and methods:** The NPs were prepared by using self-assembly methods. The size and zeta potential (ζ-potential) of NPs were measured using a Zetasizer Nano ZS90 (Malvern Instruments, Worcestershire, UK). The morphology and structures of the NPs were characterized by transmission electron microscopy (TEM) and Fourier transform infrared (FT-IR). The preservation effect of NPs on the quality of silvery pomfret (*Pampus argenteus*) during refrigerated storage is also discussed.

**Results:** Compared to treated with EGCG, NPs coating is significantly more effective in inhibiting the growth of microorganisms and the changes of biochemistry (POV, TBARS, K value, TVBN content and numbers of colony) for silvery pomfret samples during refrigerated storage, thereby extending the pomfret’s shelf life by 6d. Meanwhile, this approach also helps to maintain a better WHC and sensory characteristics by reducing the hardness changes for a longer duration compared to other groups.

**Conclusions:** These results suggest NPs could be a stronger antioxidant than EGCG alone for the preservation of fresh fish during chilled storage and have a broad potential application in the seafood industry as a biopreservative.

**Acknowledgements:** The authors would like to thank the support of Natural Science Foundation of Fujian Province of China (2014J01384 and 2017J01155).

## 137 Enhancing the bioavailability of (−)-epigallocatechin-3-gallate (EGCG) by using chitosan based nanoparticles

### Wu Chunhua^1,2^, Hu Yaqin^2^, Pang Jie^1^

#### ^1^College of Food Science, Fujian Agriculture and Forestry University, Fuzhou 350002, China; ^2^College of Biosystems Engineering and Food Science, Zhejiang University, Hangzhou 310058, China

##### **Correspondence:** Hu Yaqin - yqhu@zju.edu.cn; Pang Jie - pang3721941@163.com

*Journal of Chinese Medicine* 2018, **13(Suppl 1):**137

**Background:** Epigallocatechin gallate (EGCG) found in green tea have attracted considerable attention due to their favourable biological properties. However, their therapeutic potential is limited by their low oral bioavailability. To improve its efficiency, the EGCG was encapsulated on poly-γ-glutamic acid/chitosan nanoparticles (NPs) in this study.

**Materials and methods:** The NPs were prepared by using self-assembly methods. The particle size, zeta potential, loading capacity and structural properties of the NPs are investigated using a Zetasizer Nano, HPLC, TEM and FT-IR. The release behavior and transport mechanism of EGCG from nanoparticles (NPs) were also examined at simulated gastrointestinal tract media.

**Results:** Results showed that loading content (LC), mean particle size increased with increasing EGCG concentration, but encapsulation efficiency (EE) declined. FT-IR spectroscopy suggested that the formation mechanism of NPs is main attributed to interactions among the functional groups of CS, γ-PGA and EGCG by hydrogen bonds and electrostatic interactions. The controlled release of EGCG from NPs presented a pH-dependent pattern, and the intestinal absorption of EGCG NPs was significantly enhanced (p < 0.05) by NPs encapsulation.

**Conclusions:** The results demonstrate that encapsulation of EGCG in NPs enhances their intestinal absorption and is a promising strategy for improving their bioavailability.

## 138 The synergistic interactions between natural compound MC and chemotherapy drug Epirubicin on pharmacodynamics in vitro

### Yubo Zhang, Yingqian Ci, Huajun Li, Shuai Lu, Mei Han

#### College of Chemistry, Beijing Normal University, Beijing, China

##### **Correspondence:** Mei Han - hanmei@bnu.edu.cn

*Journal of Chinese Medicine* 2018, **13(Suppl 1):**138

**Background:** In recent years, significant emphasis has been placed on combination chemotherapy with natural compounds and chemotherapy drugs in cancer [1]. In addition, natural polyphenols play an important role in the prevention of breast cancer [2]. MC is a kind of natural polyphenols which has anti-cancer activities. Epirubicin (EPC) is an antibiotic chemotherapy drug as first-line drugs for breast cancer treatment. This study aimed to preliminarily investigate the interactions between natural compound MC and chemotherapy drug EPC on pharmacodynamics.

**Materials and methods:** For in vitro study, cytotoxicity analysis was performed by ATP assay or MTT assay.

**Results:** A low concentration of MC (1 µM) significantly increased the inhibition rates of EPC from 27.00 to 34.26% in human breast cancer brain metastasis cell lines MDA-Mb-231Br. The cell viability decreased in a dose-dependent manner with increasing doses of different treatments. Furthermore, MC enhanced EBC cytotoxicity compared to that of EBC treatment alone. Each group had a significant difference (*p *< 0.05, *p *< 0.01, *p *< 0.01, *p *< 0.01) while the combination index (CI) was less than 1.

**Conclusions:** In conclusion, MC has the potential to enhance efficacy of chemotherapy drug EPC for human breast cancer brain metastasis.

**References**

1. Shang W, Lu W, Han M, Qiao J. The interactions of anticancer agents with tea catechins current evidence from preclinical studies. Anti-Cancer Agent Me. 2014;14(10):1343–50.
2. Ci Y, Qiao J, Han M. Molecular mechanisms and metabolomics of natural polyphenols interfering with breast cancer metastasis. Molecules. 2016;21(12).

## 139 The herbal drug-pair extracts of *Paeoniae Radix Alba* and *Atractylodis Macrocephalae Rhizoma* inhibits the inflammatory responses in activated macrophages

### Yangyang Zhou, Hongxun Tao, Hua Yu, Yitao Wang

#### Institute of Chinese Medical Sciences, State Key Laboratory of Quality Research in Chinese Medicine, University of Macau, Taipa, Macao

##### **Correspondence:** Yitao Wang - ytwang@umac.mo; Hua Yu - bcalecyu@umac.mo

*Journal of Chinese Medicine* 2018, **13(Suppl 1):**139

**Background:** *Paeoniae Radix Alba* (PRA) and *Atractylodis Macrocephalae Rhizoma* (AMR) have long been used in traditional medicine for the treatment of gynecologic and gastrointestinal diseases. Previous pharmacological studies have shown that they possess anti-inflammatory, immuno-modulatory, antioxidant and anti-tumor activities, etc. In the present study, we investigated the effects of extracts from PRA and AMR (PAEs) on the suppression of pro-inflammatory responses in murine RAW264.7 macrophages with a focus on NF-κB signaling pathway.

**Materials and methods:** The PAEs were prepared using different solvents and with various ratios of the drug pair, followed by screening in terms of anti-inflammatory effects on lipopolysaccharide (LPS)-stimulated RAW264.7 cells. The cell viability was measured using MTT assay. Nitric oxide release, as a marker of anti-inflammatory effects, was determined with Griess reagent. The protein expression of inducible nitric oxide synthase (iNOS) and cyclooxygenase 2 (COX-2) in cells were measured by Western blot.

**Results:** The MTT assay indicated that the PAEs were not cytotoxic when the concentrations were lower than 600 µg/mL. The 25% ethanolic extract of PAE (PRA:AMR/1:1, w/w) dose-dependently decreased the NO production in LPS-stimulated RAW 264.7 cells and with the most potency than other PAEs. The western blot analysis indicated that the optimized PAE could effectively inhibit the LPS-induced iNOS expression but not affect the expression of COX-2. Moreover, PAE dramatically inhibited LPS-induced activation of NF-κB signalling but did not impact on the LPS-induced activation of MAPK signaling.

**Conclusions:** PAE selectively inhibits the expression of iNOS via suppression of NF-κB pathways in inflamed macrophages. Such a preferential suppression cascade by PAEs might have therapeutic potential for inflammatory diseases with over activation of iNOS.

**Acknowledgements:** This study was financially supported by the opening fund of the State Key Laboratory of Quality Research in Chinese Medicine of University of Macau (No. SKL-QRCM-2014-2016) and the Research Committee of the University of Macau (SRG2015-00060-ICMS-QRCM).

## 140 Comparative comprehension of the chemical constituents in three species of Siegesbeckiae plants: a literature review

### Hongxun Tao^1^, Yangyang Zhou^1^, Wei Sang^1^, Zhixin Li^1^, Wei Xiong^1^, Yitao Wang^1^, Hua Yu^1,2,3^

#### ^1^Institute of Chinese Medical Sciences, State Key Laboratory of Quality Research in Chinese Medicine, University of Macau, Macao, China; ^2^HKBU Shenzhen Research Center, Shenzhen, Guang Dong, China; ^3^School of Chinese Medicine, Hong Kong Baptist University, Kowloon Tong, Hong Kong, China

##### **Correspondence:** Hua Yu - bcalecyu@umac.mo; Yitao Wang - ytwang@umac.mo

*Journal of Chinese Medicine* 2018, **13(Suppl 1):**140

**Background:** Herba Siegesbeckiae (HS) is a traditional Chinese medicine which has been reported to present various bioactivities including anti-inflammation [1], anti-allergy [2], anti-thrombotic, anti-atherosclerosis, improving microcirculation [3], promoting skin wound healing [4], and anti-cancer [5]. Recommended by the Chinese Pharmacopoeia 2015 Edition [6], the plant sources of HS include *Siegesbeckiae orientalis* L. (SO), *S. pubescens* Makino (SP) and *S. glabrescens* Makino (SG). In this review, the phytochemical studies for the three HS plants were summarized and compared.

**Results:** Reviewed from the database of SciFinder, a total amount of 228 components were found to be related to HS species. Among them, 9 components were reported for all three species, as well as 6 components for SP and SG, 10 components for SO and SP, and 1 component for SO and SG. Furthermore, other 58, 130 and 14 compounds were reported to be specific for SO, SP and SG, respectively. The distribution of these compounds in SO, SP and SG was illustrated in Fig. 1. Chemical-structurally, the HS-related components could be divided into 6 groups, including flavonoids, sesquiterpenoids, diterpenoids, triterpenoids, sterols and others. Two most important groups of the chemicals related to the pharmacological activities to SHs should be diterpenoids and sesquiterpenoids. Kaurane-type and pimarane-type carbon-skeletons (Fig. 2) were the main chemical structures for SH-related diterpenoids. The key differences of chemical distributions might to be related to the sesquiterpenoids. Most germacranolides were found in SO, while a cluster of cadinane sesquiterpenoids were only found in SP.

**Fig. 1:**
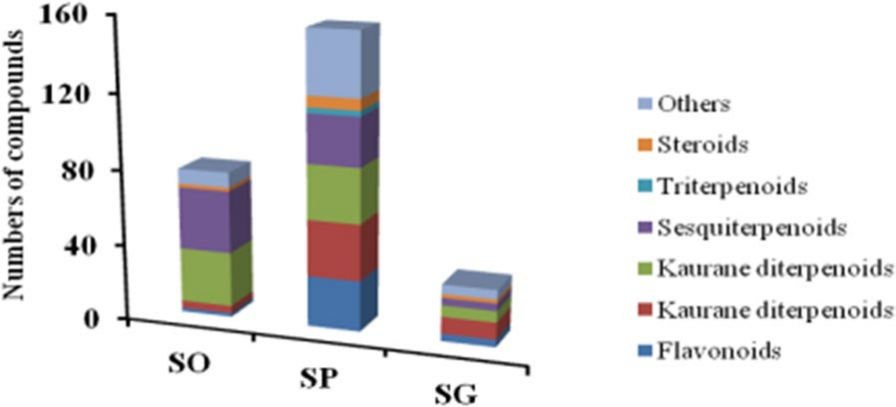
Distribution of different type of compounds in HS species. SO: *S. orientalis* L., SP: *S. pubescens* Makino, SG: *S. glabrescens* Makino

**Fig. 2:**
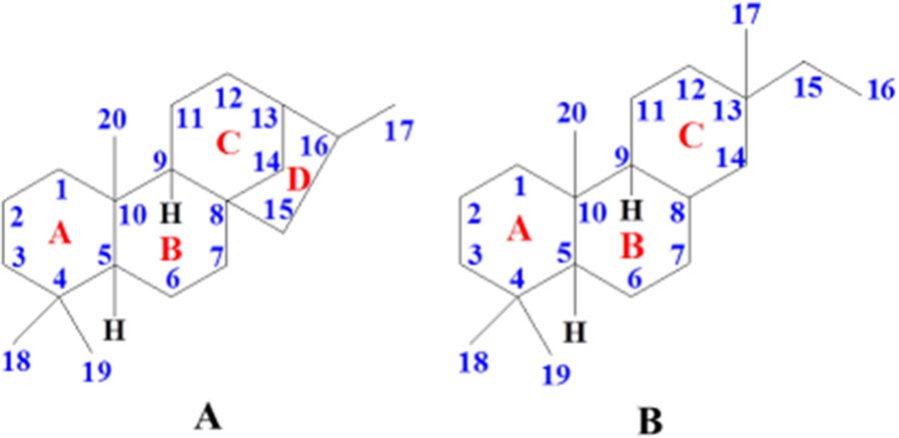
Main carbon-skeleton of kaurane-type (A) and pimarane-type (B) diterpenoids in SHs

**Acknowledgements:** This study was financially supported by the opening fund of the State Key Laboratory of Quality Research in Chinese Medicine of University of Macau (No. SKL-QRCM-2014-2016), the Research Committee of the University of Macau (SRG2015-00060-ICMS-QRCM) and the National Natural Science Foundation of China (NSFC, No. 81470170).

**References**

1. Jeon CM, Shin IS, Shin NR, Hong JM, Kwon OK, Kim HS, Oh SR, Myung PK, Ahn KS. Int Immunopharmacol. 2014;22(2):414–9.
2. Kim HM, Lee JH, Won JH, Park EJ, Chae HJ, Kim HR, Kim CH, Baek SH. Phytother Res. 2001;15(7):572–6.
3. Lee K, Jung J, Yang G, Ham I, Bu Y, Kim H, Choi HY. Phytother Res. 2013;27(9):1308–12.
4. Wang JP, Ruan JL, Cai YL, Luo Q, Xu H X, Wu YX. J Ethnopharmacol. 2011;134(3):1033–8.
5. Chang CC, Hsu HF, Huang KH, Wu JM, Kuo SM, Ling XH, Houng JY. Molecules. 2014;19(12):19980–94.
6. Commission CP. Pharmacopoeia of People’s Republic of China. 2015.

## 141 Dietary polyphenols and diabetes

### Hui Cao, Yitao Wang, Jianbo Xiao

#### Institute of Chinese Medical Sciences, State Key Laboratory of Quality Research in Chinese Medicine, University of Macau, Taipa, Macau, China

##### **Correspondence:** Jianbo Xiao - jianboxiao@yahoo.com

*Journal of Chinese Medicine* 2018, **13(Suppl 1):**141

**Background:** Diabetes mellitus is one of the most significant public health problems in the world. WHO has reported that there are about 347 million people worldwide have diabetes in 2013. The majority is seen in developing countries (South America, China, and India) undergoing westernization. For example, more than 100 million Chinese developed to type 2 diabetes in 2012. It accounts for about 9.3% of total population of China. Significant evidence has shown that the polyphenol-rich diets have the ability to protect against diabetes. Since the last several reviews focused on the nutrition and health effects including type 2 diabetes of polyphenols in 2007–2008, a number of related original publications have appeared in this area.

**Case report:** Here we summarized important advances related to influence of dietary polyphenols and polyphenol-rich diets on preventing and managing type 2 diabetes, as well as diabetes-mediated changes in pharmacokinetics and bioactivities of dietary polyphenols. It looks like that anthocyanins or anthocyanin-rich foods intakes are related to the risk of type 2 diabetes, but there is no association for other polyphenol subclasses. It illustrated that procyanidins are more active when administered individually than when mixed with food. The benefits of dietary polyphenols for type 2 diabetes can be summarized as: protection of pancreatic β-cells against glucose toxicity, anti-inflammatory, antioxidant, inhibition of starch digestion, inhibition of α-amylases or α-glucosidases, and inhibition of advanced glycation end products formation. Moreover, type 2 diabetes also significantly influence the benefits of dietary polyphenols. Although there is very limited information available so far, it is proposed that type 2 diabetes influences the pharmacokinetic behavior of dietary polyphenols including: (i) competition of glucose with polyphenols regarding binding to plasma proteins; (ii) weakened non-covalent interaction affinities of plasma proteins for natural polyphenols due to protein glycation in type II diabetes; (iii) the enhanced clearance of polyphenols in type 2 diabetes [1–4].

**Conclusion:** Although how type 2 diabetes impact the pharmacology of dietary polyphenols are not well understood. An understanding of type 2 diabetes-mediated changes in pharmacokinetics and bioactivity of dietary polyphenols will lead to improve the benefits of these phytochemicals and clinical outcomes for type 2 diabetics.

**Acknowledgements:** This research was financially supported by the Start-up Research Grant from University of Macau (SRG2015-00061-ICMS-QRCM), and the opening fund of the State Key Laboratory of Quality Research in Chinese Medicine of University of Macau (No. SKL-QRCM-2014-2016).

**References**

1. Xiao JB, Ni XL, Kai GY, et al. Crit Rev Food Sci Nutr. 2015;55:16–31.
2. Xiao JB, Högger P. Curr Med Chem. 2015;22:23–38.
3. Xiao JB, Ni XL, Kai GY, et al. Crit Rev Food Sci Nutr. 2013;53:497–506.
4. Xiao JB, Kai GY, Yamamoto K, et al. Crit Rev Food Sci Nutr. 2013;53:818–36.

## 142 Flavonoid glycosylation and human health

### Hui Cao, Yitao Wang, Jianbo Xiao

#### Institute of Chinese Medical Sciences, State Key Laboratory of Quality Research in Chinese Medicine, University of Macau, Taipa, Macau, China

##### **Correspondence:** Jianbo Xiao - jianboxiao@yahoo.com

*Journal of Chinese Medicine* 2018, **13(Suppl 1):**142

**Background:** The dietary flavonoids, especially their glycosides, are the most abundant polyphenols in diets and are of great general interest due to their diverse bioactivity. The natural flavonoids almost all exist as their O-glycoside or C-glycoside forms in plants. Here we review the advances of influence of glycosylation of flavonoid aglycones on their biological benefits, as well as the different pharmacokinetic behaviors between flavonoid aglycones and glycosides.

**Case report:** Overall, it is very difficult to draw general or universally applicable comments regarding the impact of glycosylation on flavonoids’ bioactivity and capacity for affecting human health. It seems as though O-glycosylation generally reduces the bioactivity of these compounds—this has been observed for diverse properties including antioxidant activity, antidiabetes activity, antiinflammation activity, antibacterial, antifungal activity, antitumor activity, anticoagulant activity, antiplatelet activity, antidegranulating activity, antitrypanosomal activity, influenza virus neuraminidase inhibition, aldehyde oxidase inhibition, immunomodulatory and antitubercular activity. However, O-glycosylation can enhance certain types of bioactivity including anti-HIV activity, tyrosinase inhibition, antirotavirus activity, anti-stress activity, anti-obesity activity, anticholinesterase potential anti-adipogenic activity, anti-allergic activity, and treatment for chronic kidney disease. However, there is a lack of data for most flavonoids, and their structures vary widely. There is also a profound lack of data on the impact of C-glycosylation on flavonoid bioactivity, although it has been demonstrated that in at least some cases C-glycosylation has positive effects on properties that may be useful in human healthcare such as antioxidant and antidiabetes activity. Furthermore, there is a lack of in vivo data that would make it possible to make broad generalizations concerning the influence of glycosylation on the benefits of flavonoids for human health. In spite of exhibiting diverse bioactivity, flavonoids are yet to achieve the status of promising drug candidates, and only very few these compounds have been approved for clinical application. The reason for this could be the lack of sufficient clinical or in vivo data.

**Conclusion:** Most bioactivity of flavonoid aglycones and glycosides is reported within “tubes” or “plates” and there are very few data from in vivo experiment or clinical practice. The flavonoid glycosylation on their benefit is believed to provide different outcomes between in vitro and in vivo. Flavonoids glycosides maintain higher plasma concentrations and have a longer mean residence time in the blood than aglycones. Although the attached sugar moiety on flavonoid molecules may influence their absorption and metabolic rates, flavonoid aglycones and glycosides show similar absorption and metabolism profiles in vivo.

**Acknowledgements:** This research was financially supported by the Start-up Research Grant from University of Macau (SRG2015-00061-ICMS-QRCM), and the opening fund of the State Key Laboratory of Quality Research in Chinese Medicine of University of Macau (No. SKL-QRCM-2014-2016).

## 143 Structural characterization and antiviral activity of a novel heteropolysaccharide isolated from *Grifola frondosa* against enterovirus 71

### Chao Zhao^1,4^, Luying Gao^2,3^, Bin Liu^1^, Yu Jin^2,3^, Zheng Xing^2,5^

#### ^1^College of Food Science, Fujian Agriculture and Forestry University, Fuzhou 350002, China; ^2^Medical School and Jiangsu Key Laboratory of Molecular Medicine, Nanjing University, Nanjing 210093, China; ^3^Nanjing Children’s Hospital, Nanjing Medical University, Nanjing 210008, China; ^4^Department of Chemistry, University of California, Davis, CA 95616, USA; ^5^Department of Veterinary Biomedical Sciences, College of Veterinary Medicine, University of Minnesota, Twin Cities, Saint Paul, MN 55108, USA

##### **Correspondence:** Chao Zhao - zhchao@live.cn

*Journal of Chinese Medicine* 2018, **13(Suppl 1):**143

**Background:** Enterovirus 71 (EV71), a member of the Enterovirus genus in the family Picornaviridae, is the most frequently detected pathogen in patients suffering from the handfoot-and-mouth disease. Mushrooms have long been used not only as a source of food but medicinal resource. The objective of this study is to elucidate the chemical structure and properties of *G. frondosa* polysaccharides (GFP) and investigate the effectiveness of a novel water-soluble polysaccharide fraction prepared from *G. frondosa* mycelium in for its suppression against EV71 replication [1, 2].

**Materials and methods:** A novel heteropolysaccharide from *Grifola frondosa* mycelia was extracted and purified using DEAE Sephadex A-50 and Sephadex G-200 chromatography. Fourier transform infrared (FT-IR) spectroscopy and nuclear magnetic resonance (^1^H NMR and ^13^C NMR) spectroscopy were used to decipher the structure of the purified *G*. *frondosa* polysaccharide (GFP1).

**Results:** Chemical and spectral analysis revealed that GFP1, with an average molecular weight of 40.5 kDa, possessed a 1,6-β-d-glucan backbone with a single 1,3-α-d-fucopyranosyl side-branching unit. Enterovirus 71 (EV71) is the causative pathogen of hand-foot-and-mouth disease. GFP1 was tested for its anti-EV71 activity in cultured cells, which showed that EV71 viral replication was blocked and viral VP1 protein expression and genomic RNA synthesis were suppressed. Moreover, GFP1 exhibited apoptotic and other activities by suppressing the EV71-induced caspase-3 cleavage and IκBα down regulation.

**Conclusions:** Our results demonstrate that the novel *G*. *frondosa* polysaccharide has antiviral activity, which could be valuable as a potentially new anti-EV71 therapeutic compound.

**Acknowledgements:** This work was financially supported by Natural Science Foundation (2016J06009 & 2017N5003) of Fujian Province, China, Key Project of Fuzhou Municipal Bureau of Science and Technology (2017-N-36), and FAFU grants (KXb16011A & XJQ201608).

**References**

1. Zhao C, Gao LY, Wang CY, et al. Carbohydr Polym. 2016;144:382–9.
2. Meng M, Cheng D, Han LR, et al. Carbohydr Polym. 2017;157:1134–43.

